# Universal Global Imprints of Genome Growth and Evolution – Equivalent Length and Cumulative Mutation Density

**DOI:** 10.1371/journal.pone.0009844

**Published:** 2010-04-14

**Authors:** Hong-Da Chen, Wen-Lang Fan, Sing-Guan Kong, Hoong-Chien Lee

**Affiliations:** 1 Graduate Institute of Systems Biology and Bioinformatics, National Central University, Chungli, Taiwan; 2 Department of Physics, National Central University, Chungli, Taiwan; 3 Genomic Research Center, Academia Sinaca, Taipei, Taiwan; 4 Cathay Medical Research Institute, Cathay General Hospital, Taipei, Taiwan; 5 National Center for Theoretical Science, Shinchu, Taiwan; University of Vermont, United States of America

## Abstract

**Background:**

Segmental duplication is widely held to be an important mode of genome growth and evolution. Yet how this would affect the global structure of genomes has been little discussed.

**Methods/Principal Findings:**

Here, we show that *equivalent length*, or 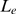, a quantity determined by the variance of fluctuating part of the distribution of the 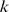-mer frequencies in a genome, characterizes the latter's global structure. We computed the 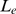
s of 865 complete chromosomes and found that they have nearly universal but (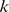-dependent) values. The differences among the 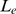 of a chromosome and those of its coding and non-coding parts were found to be slight.

**Conclusions:**

We verified that these non-trivial results are natural consequences of a genome growth model characterized by random segmental duplication and random point mutation, but not of any model whose dominant growth mechanism is not segmental duplication. Our study also indicates that genomes have a nearly universal cumulative “point” mutation density of about 0.73 mutations per site that is compatible with the relatively low mutation rates of (1
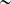
5)
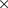
10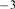/site/Mya previously determined by sequence comparison for the human and *E. coli* genomes.

## Introduction

Evolution has many facets, and one that is particularly accessible to quantitative analysis is the evolution of genomic sequences. In particular, the study of point mutations (here used in the sense that includes relatively small insertions and deletions, or indels) on genes has led to deep understandings of many aspects of genome evolution [1], [2]. Point mutation however cannot be the main force driving genome growth, because it does not give rise to gene duplication [3]–[8], and because the pace of evolution based on point mutation alone would be too slow. Gene duplication is a product of segmental duplication (SD). In fact, genomes are replete with vestiges of duplication [9]–[11], not only in the form of homologous genes, but also as transposons [12]–[14], pseudogenes [15]–[18], and many other types of coding and non-coding repeats [19]–[22]. There is also evidence of large-scale genomic rearrangements [23]–[27] and whole genome duplications [3], [28]–[30]. This has led to the generally held view that SD is an important mode of genome growth and evolution.

If products of SD are so prevalent in genomes, we expect the SD's in a genome, collectively, to leave a large imprint on the global structure of its host, one that is detectable using means not relying on sequence alignment, which in any case is not suitable for global studies. One may reasonably expect a study to understand the formation of such an imprint to yield useful insights into the global pattern of genome growth and evolution, yet no such effort has been made.

Here, we study the statistical properties of genomes by analyzing the distribution of the frequency of occurrence, or FD, of 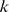-letter words, or 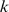-mers, in the sequence. Although genomic FDs have been much studied before [31]–[36], the method and focus of the present study are both distinct from all previous studies. A novel approach we use, crucial to our ability to extract results presented here, is the separation of the contributions to the variance from the fluctuating part of an FD (FFD), and the non-fluctuaing part (NFFD). We show that NFFD is entirely understood; it carries no statistical information other than the base composition of a sequence. A genomic sequence and its matching random sequence have essentially the same NFFD. The contribution from NFFD overwhelmingly dominates the variance (of an FD) of a random sequence in all cases and dominates the variance of a genome except when its base composition is approximately even. As a consequence, if the separation mentioned above is not carried out, then it is sometimes easy to distinguish genomic from random sequences and sometimes not, a situation that has confounded many previous studies. We will demonstrate that the very special characteristics of genomic FFDs sharply distinguishes them from their random counterparts under all circumstances.

In this study we used the FFD to define the *equivalent lengths* (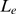
's; one for each 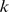) of a sequence and discovered a universality in these quantities. We then identify these 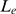
's and their small values, as a clear and distinct global imprints of genome growth and evolution. (The 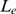 of a sequence is inversely proportional to the FFD part of the variance and is defined such that the 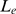 of a random sequence is its own true length. Therefore, a sequence whose equivalent length is 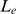 has the characteristic randomness of a random sequence of length 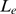.) We computed the 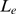 of about 900 complete chromosomes, all the complete sequences at the time of download from GenBank, for 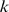 = 2 to 10, and found some unexpected and useful results: Roughly, the complete set of about 7400 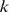-dependent whole-chromosome 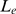
's is well represented by the universal formula 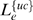
(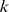) = 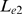 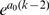 where 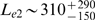 b (base pair) and 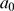 = 0.92. The formula means that, for the smaller 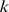
's, the universal genomic 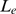 is only a small fraction of the genome length even for the shortest genomes. Another unexpected result is the small difference between the 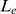
's of coding and non-coding parts. In our successful attempt to describe these results in a simple genome growth model driven by random segmental duplication, we obtained a universal cumulative point mutation density of 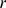 = 0.73
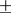
0.07/site for genomes. This value is compatible with the relatively low mutation rates previously determined by sequence comparison for the human and *E. coli* genomes [37]–[39].

## Results

### Only FFD contains non-trivial information

**Figure 1:**
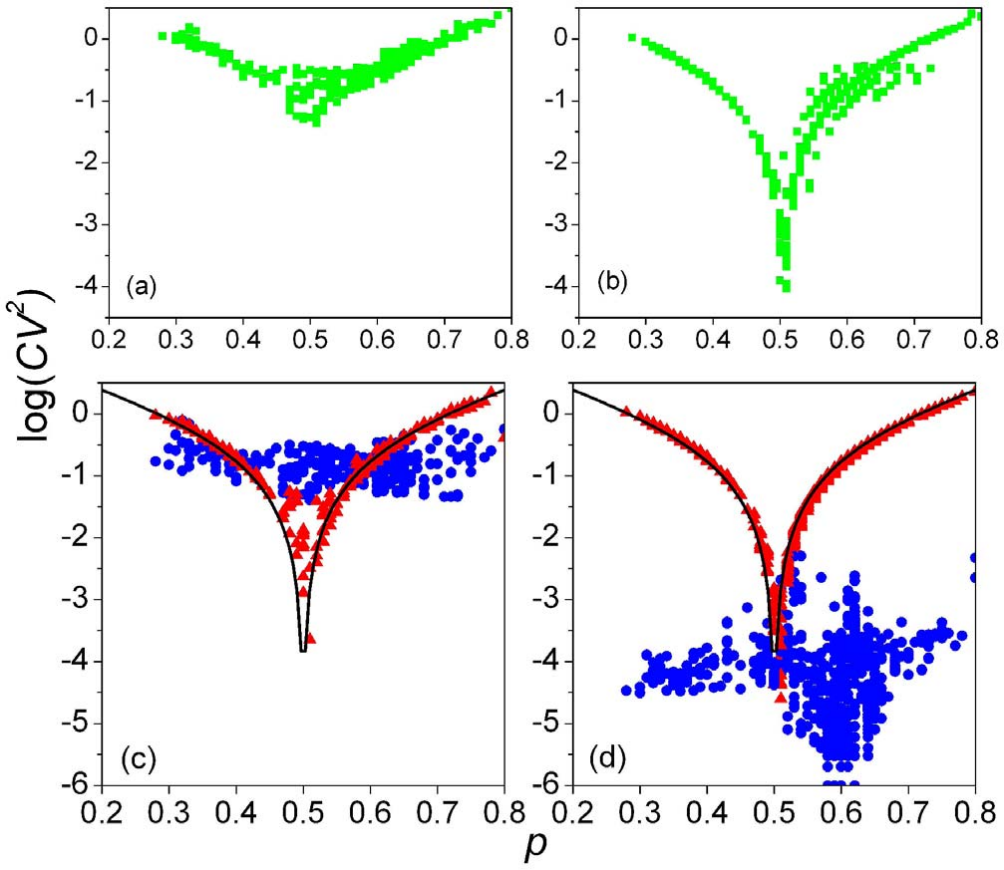
Fluctuating and non-fluctuating parts of variance. (a) Variances of 2-mer frequency distribution of 865 complete sequences. (b) Same as (a) but for for 865 matching random sequences. Bottom: same data as in top plots, but with each variance split into non-fluctuating (triangles) and fluctuating (bullets) parts, for (c) genomes and (d) matching random sequences. The “volcanic” curves through the non-fluctuating data in (c) and (d) plot theoretical values given by Eq. (12).

A key to our approach to the analysis of genomic sequences is the decomposition of 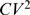 – 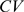 is the coefficient of variation of an FD – into FFD and NFFD components (Methods). This is illustrated in Fig. 1, which shows the values of 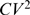 for 2-mers; results for other 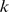
's are similar. The full 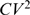 of genomic sequences (Fig. 1(a)) differs from that of their matching random sequences (Fig. 1(b)) clearly only when 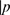 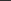 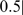 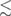
0.1, where 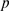 is the fractional A/T-content. (A genome and its matching random sequence have the same length and base composition.) The situation becomes much clearer when 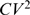 is decomposed into its FFD and NFFD parts, 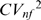 and 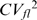, respectively. While the values of 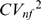 for the two type of sequences are almost indistinguishable ((red) triangles, Fig. 1(c,d); the two “volcano” curves are identical, being both given by the theoretical prediction, Eq. (12)), the values of 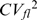 for genomes and random sequences are drastically different ((blue) bullets, Fig. 1(c,d)). The genomic 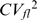 span a narrow band ranging from 0.01 to 0.1, while the random 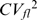 are several orders of magnitude smaller. In fact for random sequences the value of 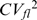 is well understood to be inversely proportional to sequence length (Eq. (13), and below). Clearly, if random sequences are used as controls to discuss the non-random properties of genomic sequences when the distinction between FFD and NFFD is not made, then it is possible that conflicting conclusions [32], [40]–[43] may be drawn.

### Genomic 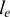 is approximately a constant of sequence length

**Figure 2:**
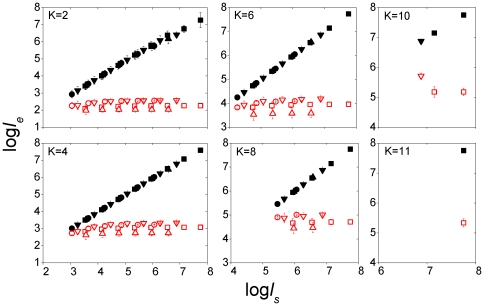
Segmental equivalent lengths from four model organisms. Equivalent length 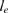 versus sequence length 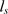 for genomic (hollow symbols) and matching random (solid symbols) sequences. Genomic segments are from *E. coli* (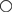), worm (*C. elegans* (*chromosome*) *I*, 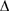), mustard (*A. thaliana I*, 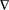), and human (*H. sapiens I*, 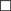). Each 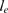 in the form of mean
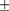
SD is averaged over the maximum number of non-overlapping segments (of length 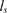) in the chromosome or, if the chromosome is longer than 20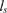, 20 randomly selected segments.

Throughout this paper we use 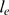 to denote generically the equivalent length of any sequence (Eq. (14), Methods), and reserve 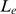 for denoting entire sequences such as a complete chromosomes. Fig. 2 shows 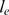 versus segment length 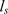 for segments taken from the chromosomes of four model organisms: *E. coli* 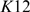; *C. elegans*, Chr. (chromosome) 1; *A. thaliana*, Chr. 1; *H. sapiens*, Chr. 1, and matching random sequences. The computation is carried out only when 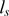 is at least four times 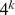, since for shorter lengths the systematic error becomes too large. It is seen that whereas the 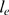 of random sequences closely tracks 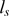, as expected, the 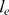 of genomic sequences quickly levels off to a saturation value 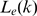. These results for 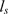 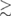
5 kb may be summarized in terms of the scaling relation 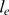 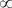 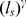. Then we have the two distinct classes 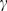 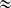
1 for random sequences and 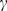 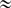
0 for genomic sequences. This scaling relation is not the same as the long-range correlation and scale-invariance observed in binary analyses of long genomic sequences [44]–[46]. In Fig. 2 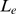 is seen not to depend strongly on organism. For small 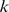, 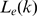 is diminutive relative to genome length: 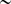
0.35 and 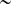
1.0 kb when 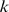 = 2 and 4, respectively, growing to 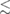
600 kb when 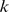 = 10. Within a genome, the apparent invariance of 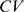 (not 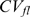) with respect to segment length was noted in [47]–[49] and the relation between Shannon information and a quantity similar to 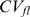 was discussed in [50].

### Whole chromosomes have nearly universal 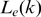


**Figure 3:**
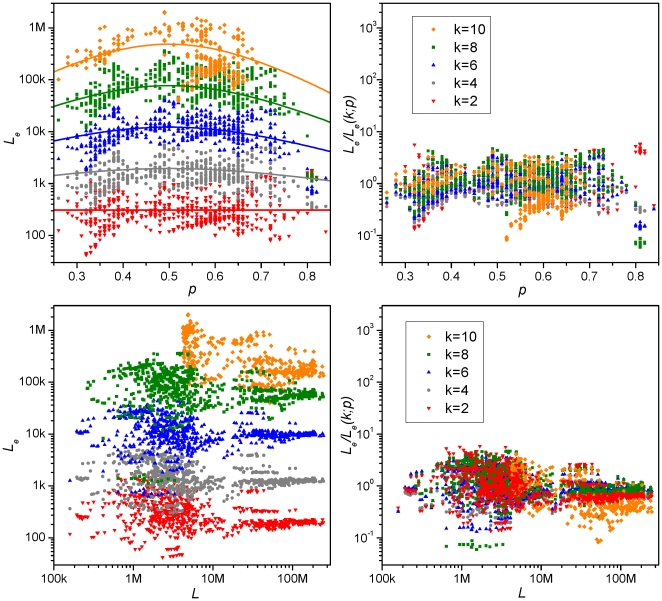
Chromosomal equivalent length (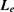) versus 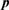 and 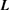. Top panels: 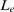 versus 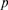; bottom panels: 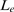 versus 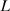. Each piece of data gives the 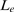 from a complete chromosome: 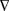 (red), 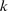 = 2; 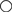 (gray), 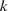 = 4; 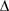 (blue), 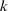 = 6, 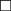 (green), 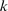 = 8, 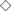 (orange), 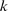 = 10. Lines in top-left panel represent the “universality class” 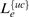 (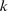;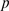) (Eq. (1)). The right panels show the collapse of genomic data to around unity when the genomic 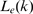 is divided by 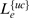 (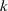;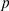).

**Figure 4:**
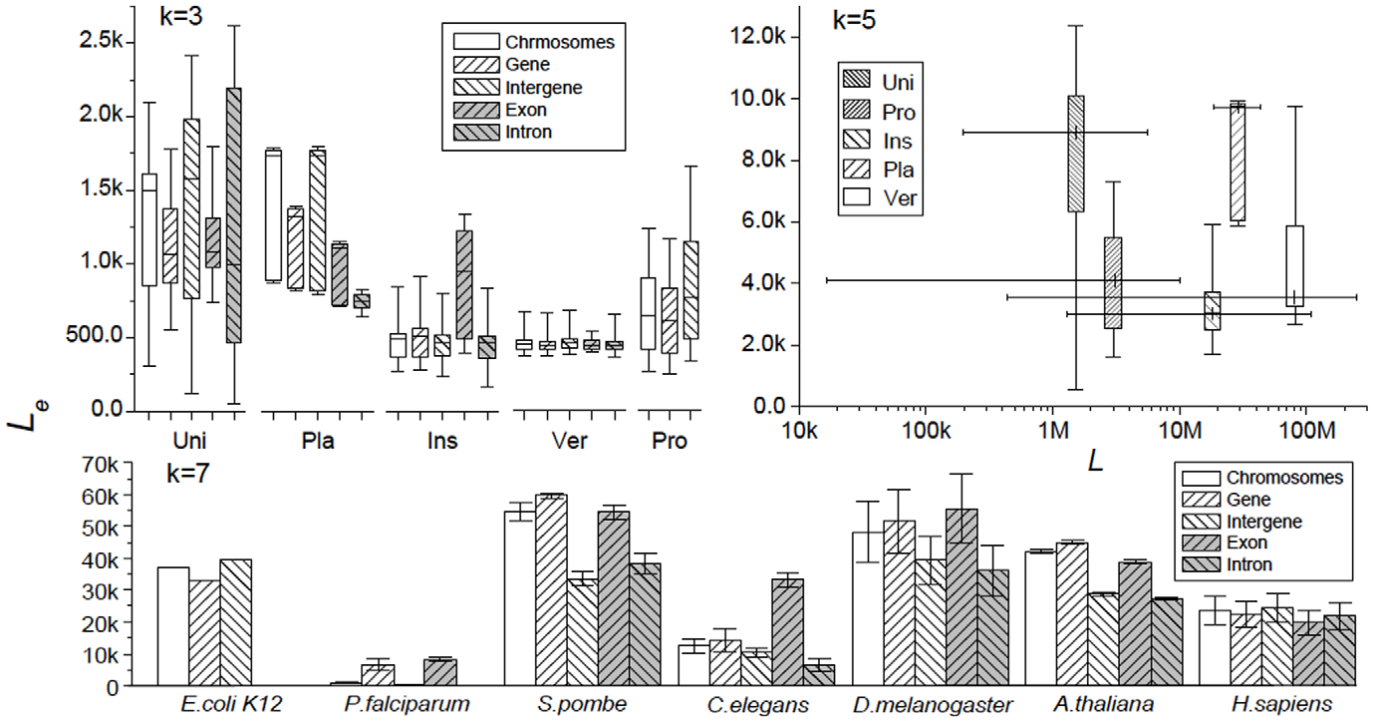
Averaged equivalent lengths for complete chromosomes and concatenates. The concatenates are: “gene” (*gn* in main text), coding regions; “intergene” (*ig*), non-coding or intergenic regions; “exon” (*ex*), exons in *gn* (for eukaryotes); “intron” (*in*), introns in *gn*. Top left, 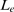 (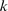 = 3) averaged over phylogenetic categories (Uni, unicellulars; Pla, plants; Ins, insects; Ver, vertebrayes; Pro, prokaryotes); top right, 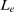 (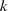 = 5) versus chromosome length average over categories; bottom, 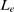 (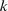 = 7) for seven model organisms averaged over chromosomes. Boxes indicate data in the 10, 25, 50, 75 and 90% range.

A list of the 865 complete chromosomes studied here is given in Table S1, and a list of 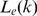
's, 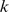 = 2 to 10, for the chromosomes is give in Table S2. Fig. 3 shows 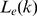, as a function of 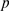 (top panels) and chromosome length 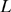 (bottom panels), computed from the complete chromosomes for even 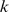
's up to 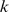 = 10. Table 1 gives the 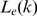, 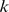 = 2 to 10, of chromosomes of seven model organisms. It is seen that 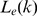 has a clear dependence on 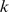, is essentially independent of sequence length, and has a weak dependence on 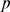. Fig. 4 gives 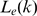 for odd 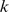
's averaged over categories of organisms and over chromosomes in model organisms (for more detailed results see Table S3). The 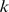 = 5 data reconfirms the absence in 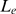 of a systematic dependence on chromosome length (similarly for other 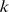
's). In the 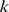 = 3 and 7 plots 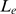
's are given separately for the whole chromosome, and genic (*gn*), and inter-genic (*ig*), exon (*ex*) and intron (*in*, when applicable) concatenates (Methods). The unicellulars are seen to have the largest variation in 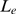, especially for the *ig* and *in* regions. This partly reflects the fact that this category includes two phylogenetically remote groups, protists and fungi. In contrast, the relatively small variation in the vertebrate 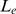 reflects the fact that, compared to organisms in other categories, vertebrates are phylogenetically very close. Two examples in opposite extremes are shown in the bottom panel of Fig. 4 (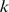 = 7): the malaria causing parasite *P. falciparum* with especially small 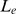
's, and the fungus *S. pombe* with relatively large 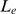
's. This indicates that the chromosomes of *P. falciparum* and *S. pombe* are much less and much more random, respectively, than the genomic norm. Although such inter-category, inter-species and inter-regional differences are significant, they pale when compared with the difference between 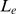 and true chromosome lengths. Table 2 lists 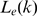, 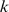 = 2, 5, 7 and 10, averaged over all 865 sequences, for whole chromosome and the four types of concatenates.

**Table 1:** Genomic equivalent lengths for model organisms.

	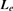 (kb) 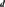								
Organism 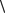 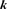	2	3	4	5	6	7	8	9	10
*H. sapiens* (24) 	.188 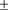 .021	.448 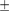 .046	1.22 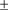 .13	3.39 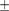 .41	9.34 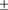 1.36	23.8 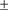 4.4	53.9 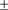 12.6	103 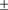 29	170 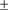 54
*H. sapiens* (*gn*; 43.2%) 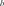	.185 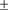 .022	.440 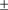 .048	1.20 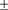 .14	3.31 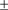 .42	9.02 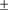 1.33	22.4 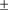 4.0	49.2 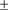 10.9	90.5 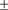 23.7	144 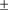 42
*H. sapiens* (*ig*; 63.6%) 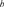	.190 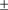 .021	.452 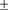 .045	1.24 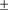 .13	3.44 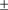 .41	9.51 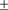 1.36	24.5 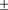 4.5	56.6 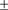 13.4	111 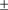 32	186 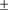 61
*H. sapiens* (*ex*; 2.1%) 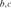	.171 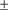 .019	.412 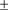 .042	1.12 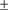 .12	3.07 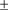 .39	8.21 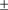 1.26	19.9 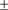 3.8	41.9 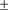 10.3	72.2 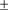 21.6	117 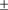 22
*H. sapiens* (*in*; 37%) 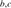	.182 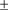 .020	.434 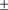 .043	1.18 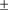 .13	3.26 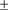 .40	8.84 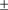 1.34	21.9 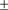 4.2	47.7 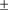 11.5	87.2 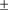 24.9	139 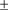 45
*A. thaliana* (5) 	.373 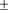 .005	.871 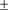 .013	2.20 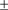 .04	5.89 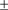 .10	16.0 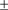 .3	42.1 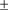 .8	109 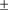 2	273 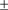 7	642 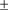 20
*A. thaliana* (*gn*; 55.8%) 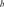	.333 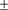 .004	.822 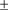 .011	2.06 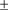 .03	5.57 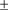 .08	15.9 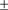 .2	44.9 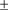 .7	129 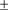 2	367 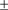 6	981 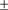 22
*A. thaliana* (*ig*; 44.1%) 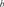	.394 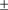 .007	.798 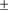 .014	1.94 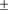 .04	4.95 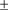 .10	12.3 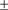 .2	28.9 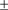 .6	66.1 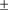 1.5	144 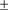 4	296 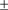 12
*A. thaliana* (*ex*; 32.9%) 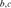	.288 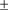 .003	.715 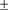 .007	1.75 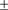 .02	4.72 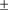 .05	13.6 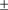 .1	38.9 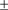 .4	113 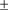 2	326 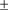 7	865 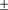 35
*A. thaliana* (*in*; 16.1%) 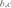	.350 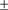 .003	.752 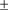 .006	1.80 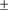 .02	4.42 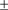 .04	11.1 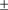 .1	27.3 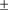 .4	68.1 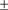 1.0	167 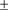 3	400 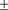 1
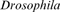 (4) 	.409 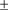 .142	.957 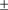 .213	2.54 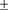 .46	6.90 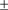 1.17	18.7 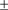 3.2	48.2 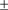 9.5	117 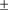 31	268 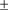 102	676 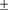 294
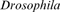 (*gn*; 56.4%) 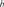	.432 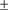 .108	1.02 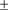 .15	2.71 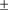 .30	7.35 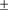 .85	20.0 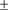 2.8	51.6 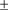 9.9	127 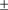 35	326 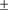 120	756 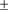 321
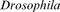 (*ig*; 43.5%) 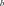	.392 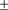 .194	.882 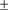 .305	2.30 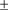 .66	6.15 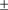 1.57	16.1 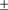 3.3	39.4 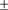 7.5	90.0 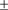 28.1	235 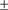 87	536 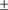 231
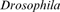 (*ex*; 23.9%) 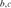	.478 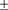 .023	1.16 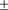 .09	2.82 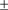 .41	7.55 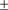 1.39	21.0 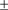 4.2	55.6 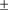 10.7	140 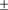 29	377 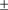 111	907 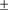 324
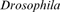 (*in*; 34.8%) 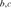	.378 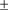 .145	.833 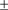 .168	2.15 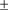 .30	5.65 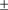 .73	14.8 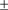 2.3	36.2 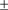 7.9	84.0 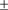 26.2	207 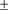 79	458 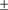 198
*C. elegans* (6) 	.119 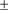 .012	.258 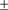 .032	.624 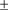 .089	1.63 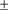 .26	4.46 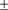 .78	12.6 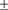 2.3	35.5 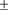 6.9	98.8 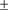 21.0	264 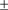 63
*C. elegans* (*gn*; 58.6%) 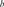	.126 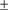 .017	.284 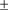 .047	.697 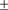 .135	1.83 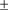 .40	5.06 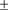 1.21	14.3 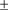 3.7	40.8 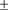 11.1	114 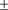 34	306 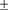 99
*C. elegans* (*ig*; 41.3%) 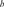	.109 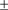 .009	.226 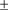 .022	.539 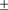 .061	1.39 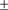 .18	3.78 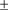 .51	10.5 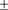 1.5	29.3 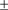 4.5	79.5 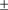 13.6	202 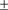 41
*C. elegans* (*ex*; 27.5%) 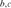	.184 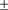 .010	.483 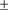 .025	1.28 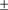 .07	3.64 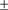 .23	10.9 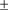 .7	33.2 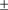 2.4	102 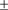 8	306 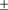 25	822 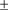 58
*C. elegans* (*in*; 32.3%) 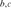	.085 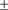 .015	.169 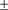 .037	.382 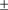 .096	.939 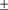 .265	2.44 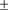 .73	6.52 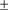 1.99	17.4 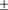 5.3	45.4 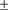 14.1	113 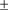 37
*S. pombe* (3) 	.362 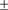 .010	.894 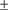 .030	2.41 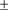 .09	6.74 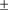 .28	19.2 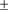 .9	54.6 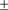 3.0	153 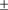 11	402 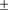 39	1013 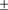 39
*S. pombe* (*gn*; 57.8%) 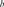	.339 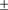 .002	.880 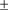 .006	2.38 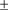 .01	6.82 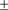 .05	20.2 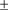 .2	59.6 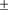 .8	173 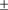 6	455 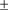 42	—
*S. pombe* (*ig*; 42.1%) 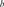	.364 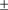 .019	.812 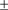 .045	2.08 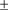 .12	5.31 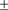 .32	13.5 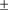 .8	33.6 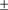 2.1	81.7 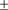 5.8	187 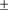 16	—
*S. pombe* (*ex*; 53.9%) 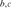	.357 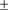 .007	.889 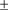 .018	2.40 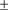 .06	6.73 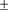 .18	19.2 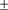 .6	54.4 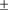 2.3	149 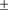 10	374 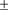 42	—
*S. pombe* (*in*; 3%) 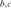	.361 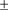 .007	.898 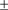 .017	2.41 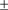 .06	6.53 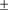 .14	17.0 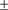 .4	38.2 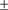 3.1	—	—	—
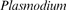 (14) 	1.40 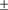 .20	.287 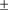 .019	.376 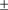 .023	.512 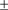 .036	.729 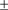 .059	.998 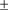 .089	1.34 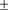 .13	1.73 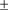 .19	—
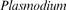 (*gn*; 56%) 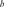	.595 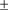 .118	.659 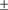 .085	1.02 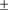 .12	1.86 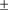 .29	3.59 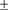 .74	6.73 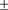 1.86	12.3 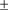 4.3	16.3 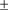 10.4	—
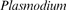 (*ig*; 44%) 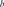	.665 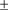 .108	.111 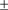 .017	.130 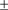 .017	.162 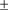 .022	.212 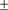 .031	.276 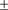 .042	.357 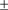 .057	.398 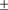 .032	—
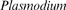 (*ex*; 53%) 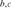	.515 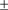 .058	.717 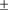 .060	1.12 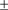 .07	2.10 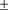 .11	4.21 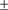 .23	8.30 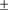 .56	16.0 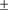 1.3	32.0 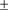 1.6	—
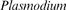 (*in*; 5.7%) 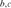	.163 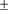 .019	.052 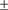 .002	.064 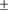 .003	.076 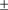 .003	.095 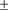 .004	.116 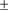 .003	—	—	—
*E. coli* (1) 	.373	.729	1.74	4.52	12.6	37.0	111	328	879
*E. coli* (*gn*; 88.7%) 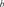	.346	.656	1.56	4.05	11.3	33.0	98.9	292	—
*E. coli* (*ig*; 11.2%) 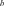	.553	1.22	2.60	6.33	16.0	39.3	83.9	—	—

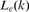
, 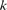 = 2 to 10, of chromosomes of model organisms. The 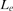
's given are mean
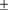
SD averaged over chromosomes of the organism, except for the single chromosome *E. coli.* See Table S2 for list of all computed 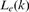
's. (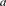) Number in parentheses indicates total number of complete chromosomes in organism. (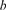) Abbreviations: *gn*, gene; *gn*, intergenic; *ex*, exon; *in*, intron. Percentage given indicates portion of complete sequence. “N-runs” or gaps in sequences are not counted. (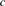) *Ex* and *in* segments selected as given by Genbank; sum of percentages for *ex* and *in* may be less than or exceed that of *gn* due to incomplete or duplicated segments. (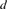) 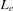
(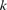) computed only if category has more than one sequence whose length exceeds 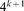.

**Table 2:** Average genomic equivalent lengths.

	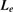 (kb)			
Category	( 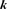 = ) 2	5	7	10
All	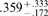	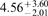	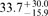	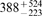
*gn* (41.8%)	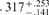	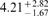	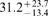	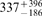
*ig* (59.6%)	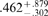	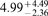	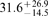	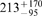
*ex* (3.3%)	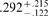	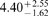	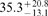	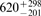
*in* (31.8%)	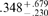	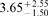	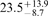	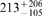
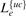 ( 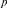 = 0.5)	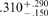	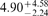	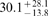	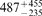
RSD model	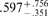	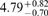	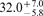	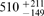

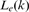
, 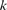 = 2, 5, 7 and 10, averaged over 865 chromosomes. Total sequences length is about 2.2
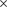
10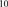 bases. Abbreviations: All, complete chromosome; *gn*, genes; *ig*, intergenic; *ex*, exons; *in*, introns. Percentage given indicates portion of complete sequence. 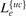 is defined in Eq. (1) and RSD results are averaged over 200 model sequences. See Table S4 for 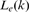 of other 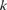 values.

### Summary of genomic data

We summarize the trends of genomic data: (a) 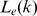 increases with 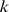. (b) For given 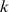, 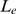 has no systematic dependence on 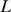 and has a weak dependence on 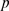. (c) For given 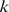, 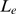 for different organisms are of the same order of magnitude. (d) Within a genome, 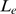 differs little among chromosomes. (e) There is remarkable agreement between the *gn* and *ex* data sets. (f) There is not a significant difference between the 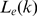
's for coding (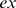 and 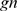) and non-coding (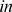 and 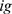) regions, and the agreement between the two regions improves when that fact that coding regions tend to be GC-rich is taken into account (Text S1 and Fig. S1). We remark that in splicing the 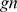 concatenate genes in positive and negative orientations from a 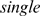 strand of DNA are concatenated, without inverting the negatively oriented genes (Methods). Similarly for the 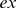 concatenate.

## Discussion

### Universal 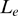 is not a result of inter-chromome similarity in 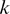-mer-content

**Figure 5:**
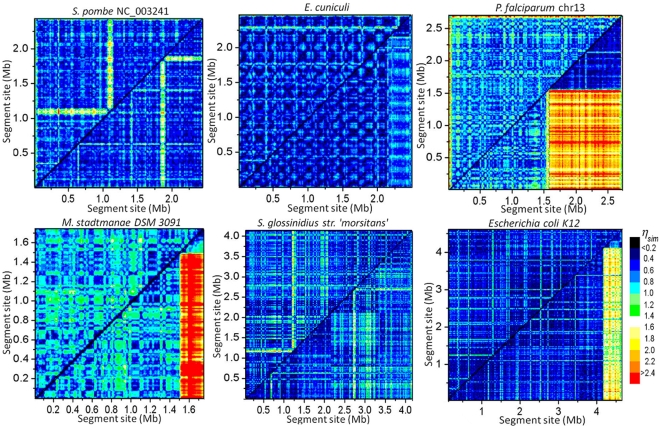
Intra-chromosomes similarity plots. Plots are for 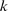 = 2 (Methods). Sliding window has width 25 kb and slide 10 kb; pixel size is 10 kb by 10 kb. In each plot, the coordinates for the upper-left triangle are sites along the chromosome (*chr*), and those for the lower-right triangle are along a concatenate composed of gene (*gn*, left side) and intergene (*ig*, right side) parts. In effect, the upper-left triangle shows *chr-chr* similarity, and the lower-right triangle shows *gn-gn* (lower-left sub-triangle), *ig-ig* (upper-right sub-triangle), and *gn-ig* (rectangular) similarities in three separate regions. The lengths of the *gn* and *ig* parts are given in Table 3.

Fig. 5 shows intra-chromosome 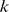-mer-content similarity plots (Methods) for six representative chromosomes. In the plots, a small value of 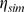 (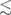
0.2, black-blue) indicates high degree of similarity, and a large value (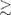
1, cyan to red) indicates the opposite. A general trend is that local 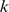-mer-content within a chromosome is fairly homogeneous [51], [52] on a scale as small as 50 kb. When 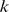-mer-contents of coding and non-coding parts show a significant difference, as is seen in the case of *P. falciparum*, *M. stadtmanae*, and *E. coli*, it is mainly caused by the *gn* part being substantially richer in GC content than the 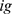 part (Table 3). Nevertheless, because 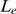 is defined such that first-order dependence in base composition is removed, within a chromosome the 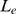
's for the 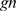 and 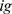 parts and for the whole chromosome generally have similar values (Table S3, 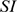).

**Table 3:** Intra-chromosome similarity indexes.

	Length (Mb)/ 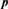			Average 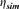			
Organism	*chr*	*gn*	*ig*	*chr-chr*	*gn-gn*	*ig-ig*	*gn-ig*
*S. pombe* Chr. 1	2.45/0.64	1.40/0.61	1.05/0.69	0.648	0.569	0.615	0.647
*E. cuniculi* (genome)	2.50/0.53	2.15/0.53	0.35/0.55	0.527	0.481	0.450	0.666
*P. falciparum* Chr. 13	2.73/0.82	1.55/0.79	1.18/0.87	0.801	0.742	0.641	2.11
*M. stadtmanae*	1.77/0.73	1.51/0.71	0.26/0.83	0.805	0.782	0.757	2.52
*S. glossinidius morsitans*	4.17/0.46	2.15/0.44	2.02/0.47	0.638	0.510	0.635	0.729
*E. coli K12*	4.64/0.50	4.12/0.49	0.52/0.58	0.517	0.481	0.548	1.63

**Figure 6:**
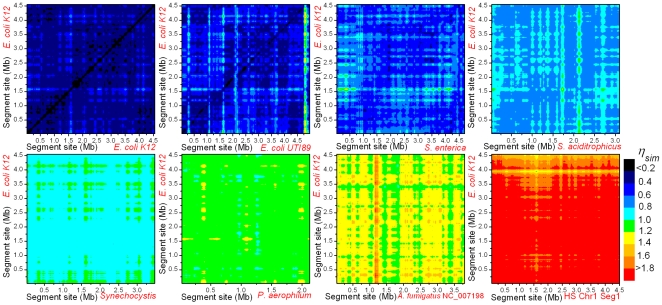
Intra- *E. coli* and inter-chromosome similarity plots. The plots are those of *E. coli* chromosome 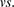 the chromosomes of, left to right and top to bottom, *E. coli*, *E. coli UT189*, *Salmonella*, the delta-proteobacteria *S. aciditrophicus*, the cyanobacteria *Synechocystis*, the archaea *P. aerophilum*, chromosome 5 of the fungus *A. fumigatus*, and the first 4.5 Mb segment from chromosome 1 of *H. sapiens*. Coordinates are sites along the sequence. Sliding window width is 100 kb and slide is 25 kb, pixel size is 25 kb by 25 kb.

Compositions and average regional similarity indexes of sequences shown in Fig. 6; *chr*, chromosome; *gn*, gene; *ig*, intergenic.

**Figure 7:**
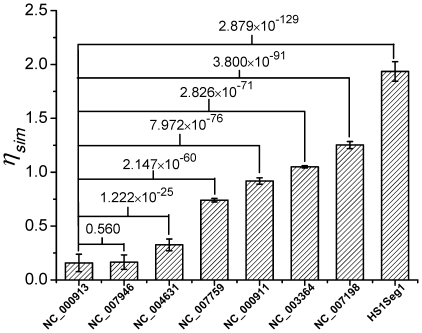
Comparison of inter-chromosome similarity matrices. Mean values and SD of the eight 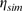-plots (of 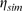-matrices) shown in Fig. 6 and P-values for the null assumption that the 2nd to 7th cases are the same as the 1st case.

Fig. 6 compares the intra-*E. coli* plot with inter-chromosome plots of *E. coli* versus seven other organisms whose phylogenetic distances to *E. coli* range from close to remote. The approximate monochromaticity of each plot reconfirms our previous observation that 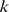-mer-content within a chromosome has a high degree of homogeneity (on a scale of 100 kb). We see close correlation between phyogenetic distance and the shades (colors) of the seven inter-chromosome plots. Fig. 7 gives the mean 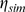 for the plots and P-values from Student t-tests for the null assumption that the inter-chromosome plots are the same as the intra- *E. coli* plot. These results verify that the observed near universal value in 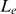 is not cause by similarity in 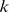-mer-content among chromosomes.

As an aside, we note that in Fig. 6 the plot for *S. pombe* indicates a 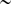
100 kb *ig* segment around the 1.1 Mb site has extraordinary low similarity with respect to all other regions of the chromosome. This could be the result of a non-genic horizontal/lateral transfer [53], [54] and suggests that similarity plots may be useful for locating such events.

### A universal formula for 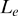


The 7360 pieces of data in the “All” set in Table 2 is well represented by the empirical formula,
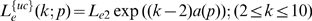
(1)

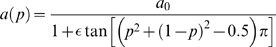
(2)where 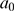 = 0.92, 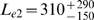 b, and 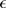 = 0.50
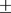
0.05. The central values of the formula are shown as solid lines in Fig. 3 and listed as the entries in the row labeled 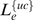 in Table 2. The denominator in Eq. (2) represents the residual 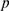-dependence indicated in the data in Fig. 3; it works well even for chromosomes with large 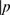 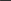
0.5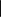 (Table S4, 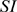). For the vast majority of genomic 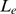
's, 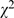 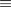 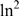
(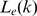/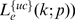 (Text S1) is less than 1 (Fig. S2) and, averaged over the 7360 pieces of data in the “All” set, 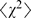 = 0.43. This means that on average the genomic 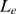 is within a factor of two of 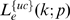. In recognizing that genomes as a category exhibit such a non-trivial common feature which is itself the manifest of an underlying but yet undetermined cause, we say genomes belong to a *universality class*. It is realized that Eq. (1) cannot be extended to 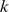 much greater than 10 (and not even to 10 for some of the smaller chromosomes), because a meaningful value for 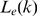 may be extracted only when a sequence is at least 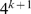 bases long.

### A universal formula for the standard deviation from the fluctuating part in 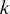-mer frequency

The short genomic 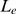 (relative to actual chromosome length) is a direct consequence of the genomic 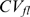 being much larger than its random-sequence counterpart. If we approximate 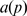 in Eq. (1) by 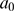 and approximate the factor 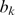 in Eq. (14) (Methods) by unity, then through Eq. (14) we convert Eq. (1) to a universal formula for the 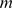-set-averaged standard deviation for the 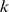-mer FFD:
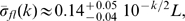
(3)where 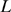 is the sequence length. The formula is meant to be applicable so long as 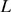 is several times greater than 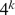. For sequences with 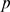 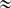
0.5, 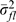 reduces to the usual variance. Note that for random sequences 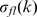 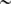 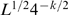. Since 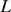 is large, genomic 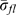 can be orders of magnitude greater than its random counterpart. For instance, for the 4.6 Mb chromosome, the 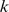 = 4 values for 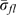 given by Eq. (3), the actual chromosome (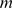-averaged), and a random sequence are 6440 b, 6230 b, and 134 b, respectively, and for the 228 Mb human chromosome 1, the corresponding values are 319,000 b, 380,000 b, and 943 b, respectively. To give statistical meaning to such differences, Table 4 examines universal genomes of various lengths and gives the fractions of 2-mers and 9-mers (in the genomes) whose frequencies have P-values that are less than P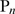 – the P-value corresponding to 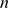 standard deviations away from the expected frequency in a random sequence – for 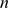 = 3, 6, and 8, respectively. Because 
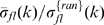
 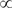 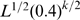, the fraction increases with decreasing 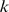 and increasing 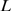 (for a given 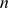). For instance, for a sequence 4.6 Mb long (length of *E. coli* chromosome), fourteen of the sixteen 2-mers have P
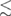
P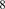 ( = 1.3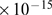), whereas only 26,000 of the 262,144 9-mers are so. In comparison, for a sequence 226 Mb long (length of human chromosome 1), all sixteen 2-mers and 213,000 of the 9-mers are so.

**Table 4:** P-values for 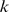-mer distribution in universality class.

Fraction of 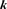 -mers whose P-value is less than P 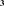 , P 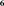 , or P 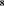						
	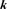 = 2 ( 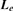 = 310 b)			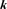 = 9 ( 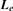 = 194 kb)		
Length (Mb)	P 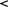 P 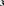	P 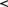 P 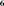	P 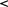 P 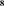	P 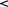 P 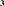	P 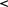 P 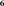	P 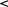 P 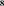
0.8	0.953	0.906	0.875	0.139	0.0031	0.0001
4.6	0.980	0.960	0.955	0.538	0.418	0.100
30	0.992	0.985	0.979	0.809	0.628	0.519
226	0.997	0.994	0.992	0.930	0.860	0.815

P-values for 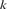-mer distribution given by Eq. (1) (at 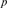 = 0.5). Null theory assumes genomes are random sequences. The P-values P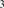 = 2.7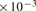, P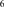 = 2.0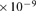, and P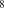 = 1.3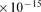 correspond to 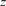-values of three, six and eight, respectively.

### Segmental duplication shortens 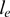


We now discuss probable causes for the formation of the universality class. We first list some general properties of the ratio 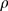 of 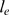 to the sequence length 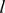: if the sequence is (nearly) random then 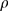
( = 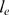/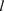)
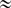
1; if it is far less random than a random sequence of length 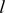 then 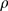 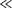
1; if it is essentially ordered then 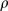 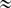
0; if it is the 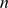-fold replication of a random sequence, then 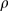 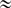
1/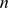. We illustrate how segmental duplication can cause a sequence to have 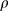 much less then one, by considering the effect of a generalization of the operation of replication on 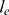. To be specific we label XY a concatenate composed of X and Y. If Y is a coarse-grained *rearrangement* of X, then, provided the scale of the rearrangements is not too small, 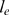
(X)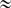 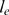
(Y) and concatenating X and Y is similar to doubling X by replication, hence 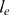
(XY) will be nearly equal to 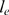
(X).

In general, if the 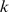-mer-contents of X and Y are similar, then (provided the sequences are sufficiently long) we expect 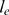
(XY)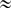 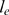
(X)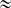 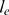
(Y). Conversely, if the 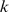-mer-contents of X and Y are significantly different, then we expect 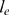
(XY)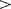 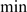
(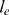
(X), 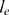
(Y)) (see Text S1 for an expanded discussion, including formulas given in Table S5). Results for testing these simple rules with real sequences are shown in Table 5. We expect agreement with theory to improve with increasing sequence length (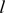). The first two rows of results in Table 5 verify that for random sequence 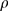 is always close to one, or 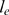 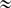 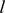. The results for AA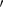 and BB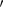 show that concatenating two equal-length segments from the *same* chromosome is indeed like doubling a sequence by replication. Chromosomes labeled C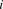 have 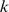-mer-contents relatively more similar to A (Figs. 4 and 5), therefore 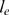
(AC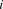)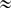 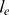
(AA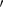)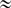 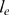
(A) as expected. Chromosomes labeled D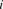 and B have 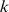-mer-contents more dissimilar to A, therefore 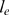
(AX)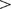 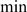
(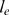
(A), 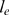
(X)). The case of AD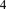, where D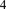 is *H. sapiens chr. 1*, is not an exception to the rule even for 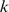 = 2, because 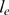
(D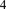)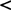 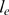
(A). In the bottom portion of Table 5 the approximate relation 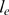 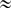 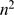 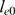 (Table S5; 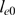 is the equivalent length of the genomic portion and 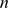 is the ratio of the length of the concatenate to the that of the genomic portion) is seen to hold: 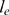
(RX)
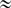
4
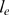
(X) (X being A or B), 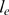
(RAB)
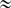
2.3
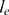
(AB), and 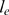
(RR'X)
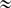
9
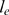
(X).

**Table 5:** Equivalent lengths of composite sequences.

	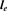			
	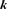 = 2		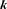 = 6	
Sequence	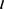 = 50	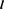 = 200	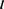 = 50	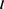 = 200
R	47.5 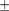 28.2	154 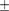 126	48.6 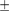 1.5	192 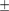 5
RR 	37.0 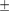 16.2	124 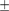 46	48.2 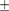 1.2	197 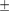 5
A	.348 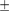 .037	.360 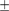 .033	9.55 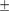 .69	11.7 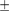 .7
AA 	.357 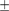 .046	.352 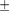 .023	9.88 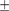 1.07	11.1 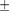 .7
AC 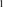	.351 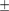 .061	.361 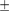 .021	9.37 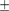 1.01	11.5 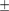 .6
AC 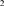	.354 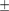 .043	.384 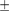 .045	9.18 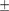 .83	11.6 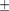 .9
AC 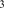	.359 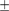 .051	.371 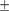 .034	11.0 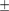 .9	14.2 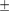 1.5
AD 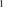	.411 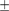 .044	.423 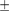 .024	11.8 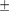 .9	14.3 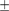 .6
AD 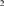	.942 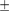 .275	1.05 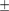 .09	14.9 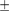 1.4	20.4 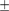 1.1
AD 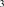	.598 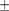 .104	.613 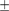 .052	17.9 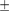 1.6	24.0 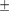 1.6
AD 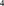	.324 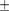 .052	.383 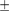 .055	11.2 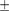 1.9	16.9 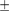 1.9
B	.124 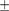 .029	.166 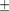 .099	5.17 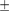 .68	6.54 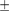 2.00
BB 	.232 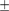 .155	.258 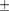 .183	6.16 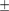 1.94	7.54 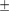 2.30
AB	.463 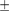 .241	.502 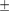 .263	11.2 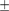 1.9	15.2 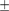 3.5
RA	1.19 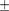 .09	1.34 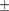 .20	22.6 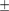 1.2	38.5 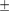 3.0
RB	.575 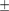 .321	.754 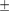 .637	15.6 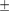 4.2	23.3 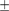 8.5
RAB	.873 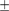 .424	1.10 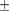 .49	18.4 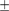 3.2	31.3 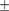 6.0
RR  A	2.63 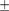 .66	3.16 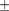 .30	31.5 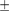 2.1	72.2 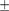 6.8
RR  B	1.03 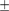 .62	1.37 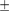 .70	22.9 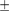 4.5	44.7 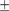 14.3

Equivalent lengths 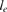 of composite sequences of total length 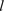 (in kb). The composite XY is the concatenation of two equal-length components X and Y. Similarly for the composite XYZ. A and A are segments from *E. coli*, and B and B are from *C. tetani* (2.80 Mb, 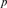 = 0.70). C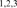 and D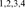, are the seven “other” chromosomes in Fig. 6, in the order given there. R and R are 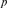 = 0.5 random sequences. Results are averaged over 10 samples in all cases.

### Artificial sequences generated by RSD growth model exhibit universal 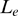


**Figure 8:**
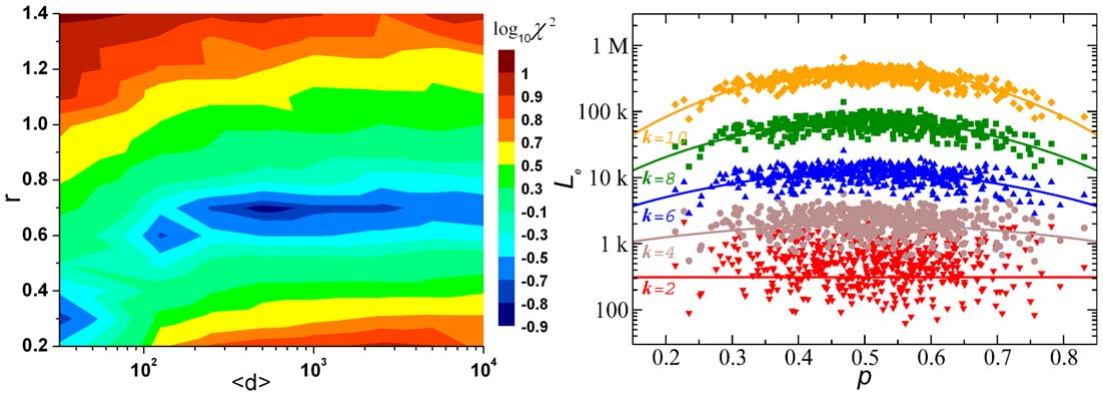
Results from minimal RSD model. Left: Equi-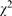 contour on the 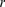-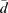 plane, with 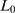 = 64 (bases). Right: 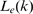, 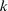 = 2, 4, 6, 8, 10 from 200 model sequences of length 2 Mb generated using the “best set” of parameters 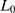 = 64, 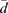 = 1000 (b) and 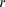 = 0.73 (b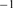). Lines in right panel are 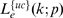 (Eq. (1)).

We show that a very simple growth model, the minimum random segmental duplication (RSD) model [49] (Methods; Text S1)), generates chromosome-length sequences that have 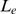
's very close to the universal 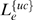 given by Eq. (1). In the model, simple segmental duplication (SD) serves to represent the numerous modes of DNA copying processes known to occur in genomes [9]–[11], [55], [56], and point mutation represents all small non-duplicating events. We consider random events because it is the simplest assumption and because it generates sequences with a reasonable degree of homogeneity [51], [52]. (It is known that genomes have long-range correlations that require tandem SDs to generate [46], [57]. Since tandem duplications do not effect 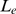, for simplicity they are not given special treatment in this study.) The three parameters of the model are 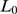 (initial length), 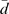 (average duplicated segment length), and 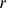 (cumulative point mutation per-base density) (Methods. 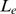 generated by the model is insensitive to sequence length provided it is longer than 0.5 Mb, allows a generous range in 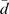 and a tighter range in 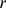, and is highly sensitive to 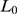 (Fig. S3, 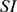). (Because RSD will at least initially cause 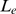 to be longer than 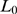 and because 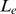 (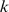 = 2)
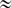
300 b, 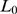 must be significantly less than 300 b.) Fig. 8 shows that, at 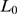 = 64, the model admits a basin of good values delimited by 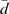 = 120 to 5000 and 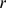 = 0.65 to 0.80. 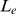
's of model sequences obtained using the “best set” of parameters 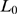 = 64, 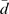 = 1000, and 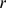 = 0.73 are shown in the right panel in Fig. 8, where the lines represent the universality class 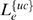 (Eq. (1)). The 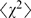 for these 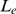
's is 0.18 and implies that on average, the model 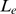 and 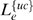 agree to within a factor of 1.6. This small 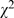 can easily be increased to match that of the genomic data (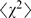 = 0.43) by using model parameters that cover suitable ranges of values centered around the best values.

The range of 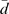 within the basin of good values seems biologically realistic, for it is consistent with the range of the characteristic lengths of genes. The isolated basin near 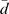 = 30, 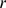 = 0.3 allows copious duplication of regulatory sequences, including microRNAs [58], that are much shorter than genes. The considerable size of the main basin implies that it is easily accessible in an evolutionary selective process. On the other hand, that 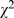 increases sharply outside the basin of good values demonstrates that even in the context of the RSD model it is very easy to generate sequences that are far outside the universality class.

### Rates of genome growth and duplication

The parameters of the RSD model are compatible with rates of genome growth and duplication determined using sequence comparison [37]–[39]. In a model where a genome grows at a constant per-time rate 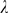, we have 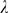 = 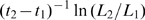 where 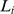 is the length of the genome at time 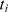 (Eq. (16), Methods). For human we can take 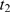 to be the current time because the human genome has grown 15% to 20% in the last 50 Mya (10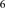 years) [39]. The ancestors of eubacteria and archaea-eukaria diverged 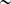
3.4 Gya (10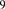 years) ago [59]–[61]), and before that proto-genomes most likely evolved as communities [62]–[64], and hence had a different growth regime than later times. The smallest bacterial genome is about 0.2 Mb; we take 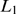 to be from 0.05 to 0.2 Mb and 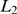 = 3 Gb. Then 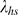 = 2.7
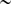
3.7/Mya. These rates imply the human genome grew 14
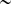
20% in the last 50 Mya, in agreement with [39]. If we assume the growth is purely SD and take the length of duplicated segment 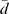 to be 500 b to 2 kb, then the rate of SD events is 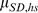 = 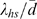 = 1.4
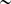
7.4/Mb/Mya. These values are comparable to the estimates of 3.9/Mb/Mya (from animal gene duplication rate of 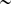
0.01 per gene per Mya [6] and human coding region 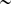
3% of genome), and 2.8/Mb/Mya (from human retrotransposition event rate [39]).

### Cumulative mutation density and mutation rates

The parameter 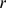 in the RSD model, the cumulative point mutation density, is related to the (per-site per-time) rate density 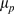 of “point mutations” – including small deletion and insertion but excluding SD – by 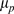 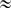 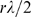 (Eq. (19), Methods). If we take the best value 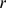 = 0.73 from the RSD model then 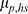 = 0.98
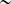
1.4
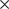
10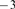/site/Mya. This agrees well with the value 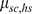 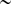
1
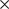
10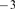/site/Mya [37]–[39] determined by sequence comparison.

We cannot assume the *E. coli* genome is still growing, as the human genome appears to be. Instead, like most bacteria *E. coli* probably acquired its full length in antiquity, not too long after ancestors of eubacteria and archaea-eukaria diverged [61]. If we assume *E. coli* acquired its current length of 4.6 Mb about 0.4 to 0.6 Gya after that, then with 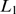 as before, we have 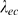 = 5.4
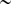
11/Mya, and 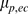 = 2.0
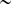
4.0
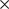
10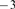/site/Mya. Fortuitously or perhaps this range of rates represent an equilibrium value, it is compatible with the sequence-comparison *E. coli* rate of 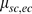 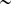
5
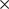
10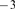/site/Mya based on mutations that (putatively) occurred in the last 0.5 Gya or less [37], [38]. There is some evidence that natural selection does cause genomes to have a relatively low and stable mutation rate. For instance, laboratory measured spontaneous mutation rates of *E. coli* [65], *C. elegans* [65], [66], and 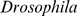 [65], [67] tend to be two or three orders of magnitudes higher than the characteristic rates of 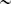
0.001/site/Mya of wild types.

Presumably the same selective force is what causes the 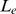
's, hence the cumulative mutation density 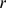, of coding and non-coding regions of a chromosome to be nearly equal. Such a force must be acting for otherwise we expect non-coding regions to have a significantly higher 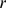, which is not the case.

## Materials and Methods

### Complete genome sequences

A total of 865 complete chromosomes were downloaded from the genome database [68] on 2006/10/01. The set is composed of 467 prokaryotic chromosomes (435 eubacteria and 32 archaea) and 398 chromosomes from 28 eukaryotes including: 12 unicellulars (*A. fumigatus* (8 chromosomes), *C. albicans* (1), *C. glabrata* (13), *C. neoformans* (14), *D. hansenii* (7), *E. cuniculi* (11), *E. gossypii* (7), *Kluyveromyces lactis* (6), *S. cerevisiae* (16), *S. pombe* (3), *Y. lipolytica* (6), *P. falciparum* (14)), 5 insects (*A. gambiae* (3), *A. mellifera* (16), *C. elegans* (6), *D. melanogaster* (4), *T. casteneum* (10)), 2 plants (*A. thaliana* (5), *O. sativa* (12), 9 vertebrates (*B. taurus* (30), *C. familiaris* (39), *D. rerio* (25), *G. gallus* (30), *H. sapiens* (24), *M. multatta* (21), *M. musculus* (21), *P. troglodytes* (25), *R. norvegicus* (21)). The complete list of sequences, their accession numbers, lengths and other properties relevant to this study are given in Table S1.

### Partition of 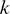-mers into 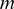-sets

We always speak of single-stranded sequences. We refer to a 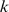-base nucleic word as a 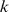-mer and denote the set of all 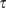 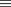 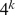 types of 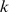-mers by 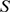. Given a sequence, we count the frequency of occurrence (or frequency) 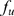 of each 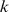-mer-type 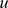 in 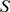 using an overlapping sliding window of width 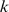 and slide one [36]. Then the sum of the frequencies is 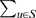 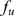 = 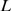
−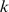
+1, here approximate by 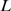, and the mean frequency is 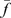 = 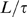. Let the fractional AT- and CG-content of a sequence be 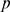 and 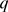 = 1−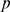, respectively. We say a sequence has an even-base composition when 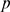 is equal to or very close to 0.5, otherwise it has biased base composition. Owing to Chargaff's second parity rule [69] 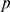 is an accurate and efficient classifier of base composition for statistical analysis. The 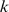-mers in a sequence are naturally partitioned into 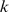
+1 “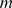-sets”, 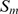, 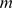 = 0,1,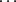 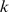, where each 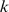-mer in 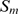 has 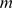 and only 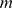 AT's; 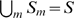. For example, in the case of 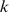 = 2, 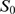 is the set {CC, CG, GC, GG}; 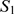 is the set {CA, CT, GA, GT, AC, AG, TC, TG}; and 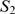 is the set {AA AT, TA, TT}. The the number of types of 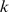-mers in 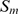 is 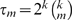, which satisfies the sum-rule 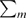 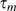 = 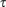 = 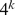. These relations derive from the binomial expansion (for given 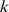)
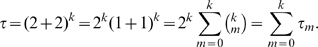
(4)Let 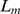 = 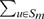 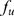 be the sum frequency of the 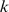-mers in 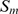. Then 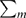 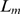 = 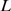 and the mean frequency of the 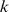-mers in 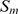 is 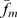 = 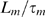. The large-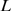 limit of 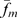 for a random sequence, 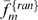, is obtained from the binomial expansion
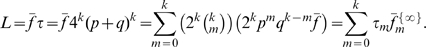
(5)That is,
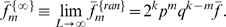
(6)Depending on 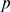, 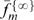 can vary widely, all collapsing to 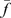 when 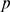 = 0.5. Eq. (6) not only provides an highly accurate estimate of the value of 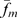 for genome-size random sequences, it also gives a reasonable estimate for genomic 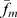 (Table 6).

**Table 6:** Average frequency of occurrence (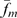) of 5-mers in 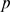 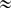
0.5 and 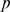 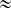
0.7 sequence.

	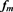					
Sequence	( 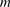 = ) 0	1	2	3	4	5
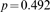						
*E. coli*	2509	2245	1877	1760	1944	2656
Random	2101	2044	1987	1922	1857	1795
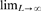 Random 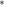	2114	2048	1983	1920	1860	1801
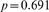						
*C. acetobutylicum*	154	397	918	1951	4272	10300
Random	176	394	882	1970	4400	9832
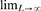 Random 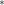	176	393	880	1968	4402	9845

All sequences normalized to a length of 2 Mb; 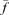 = 2
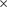
10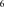/4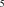 = 1953. Random means matching random sequence, or sequence obtained by scrambling the genome. 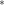
Values of 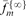 given by Eq. (6).

### Fluctuation in occurrence frequency

**Figure 9:**
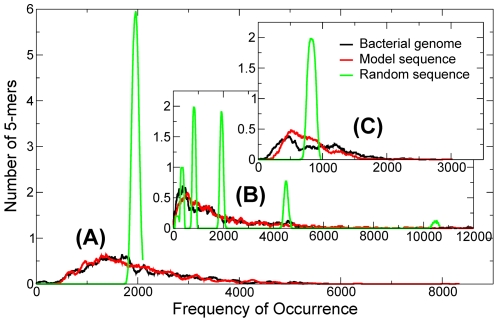
Frequency distributions of 5-mers. Frequency occurrence distributions, or spectra, of 5-mers from the genomes of two prokaryotes, (A) *E. coli* (with (A+T) content 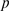 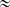
0.5) and (B) *C. acetobutylicum* (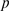 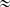
0.7), normalized to a sequence length of 2 Mb. Abscissa give occurrence frequency and ordinates give number of 5-mers averaged, for better viewing, over a range of 21 frequencies to reduce fluctuation. The black, green and red curves represent spectra of the complete genomes, the randomized genome sequences and sequences generated in a model (see text), respectively. (C) Details of the m = 2 subspectra from (B).

The coefficient of variation of the frequency distribution is 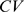 = 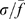, where 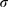 is the standard deviation. For random events of equal probability, here translated to 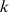-mer frequencies of a (long) random sequence with even-base composition, the distribution is Poisson and 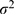 = 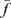, hence 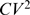 = 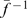 = 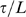, which tends to zero in the large-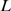 limit. This no longer holds when the random sequence has a biased base composition. As controls we consider random sequences that *match* genomes, namely those whose lengths and base compositions are the same as their genomic counterparts. In particular, such sequences obey Chargaff's second parity rule [69] in that their A and T, and C and G, separately have nearly equal probabilities. For any sequence whose 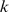-mers are partitioned into 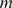-sets, using a generalization of the parallel axis theorem, we write as follows:
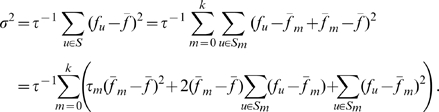
(7)The second term vanishes upon summing over 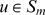, so 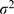 is composed of two parts,
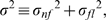
(8)a *non-fluctuating* part determined by average frequencies 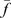 and 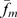,
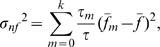
(9)and a *fluctuating* part determined by the fluctuation of 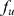 (in an 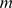-set) around an average frequency,
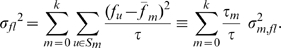
(10)Thus,
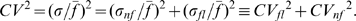
(11)The non-fluctuating, or “non-statistical”, part, 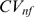, has a well-defined value in the large-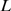 limit, obtained by replacing 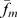 by 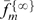 in Eq. (9):
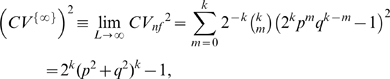
(12)which has a strong dependence on 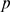 and vanishes 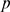 = 0.5. Because genomes are large, 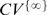 gives an accurate description of 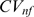 for genome-size random sequences; it also happens to do almost as well for genome (Fig. 1). Owing to the existence of this term, the 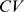 for a genomic sequence may be much greater than that of its matching random sequence (when 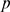 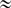
0.5; see, e.g., Fig. 9 (A)), or quite similar (when 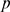 differs significantly from 0.5; see, e.g., Fig. 9 (B)). Because 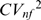 hardly depends on the distribution of the 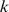-mers, it should be considered a *background* in 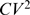 in relation to the *signal* which is 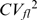.

For a random sequence, the frequency distribution in the subset 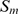 is nearly Poisson, hence 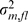 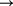 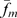 in the large-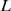 limit. Therefore, from Eq. (10),
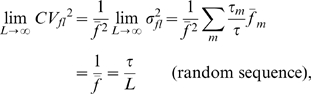
(13)which is exactly the limit expected of 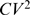 for an even-base (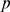 = 0.5) random sequence. In other words, for random sequences 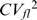, but not 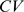, has the correct large-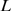 limit expected of a random system. The right-hand-side does not depend on 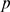, which is a reflection of the fact that for genome as well as random sequences, 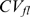 has at most a weak 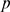-dependence; the main 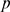-dependence having been removed when 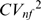 is subtracted from 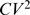. Because (for random sequences) 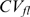 decreases with increasing 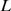 but 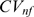 does not, there is a crossover value of 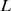 beyond which 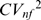 becomes the leading term in 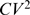 (when 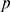 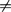
0.5). When 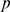 = 0.7, this crossover value is 42, 316 and 2851 (bases) for 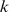 = 2, 4, and 6, respectively, which are orders of magnitudes shorter than even the smallest chromosomes. To summarize, if one wants to compare the *statistical* properties in the frequency distributions of 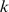-mers in the genomic and random sequence, one must use 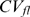, not 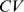.

### Two examples: *E. coli* and *C. acetobutylicum*

**Figure 10:**
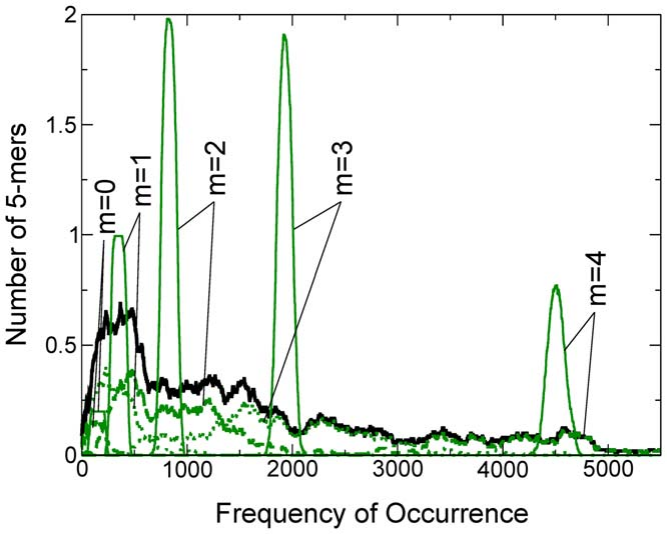
Frequency distributions of 5-mers in 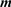-sets. Details of 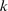 = 5, 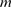-specific subspectra from the *C. acetobutylicum* genome (broken green curves) and matching random sequence (solid green curves); black curve is the same as in (B) Fig. 9. The five narrow subspectra peak (approximately) at 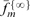, 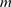 = 0 to 4, or at 152, 354, 827, 1939, 4500, respectively; the 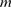 = 5 peak at 10500 is off scale (see Fig. 9 (B)).

We explain the formulation presented in the last two sections by presenting results of distributions, or spectra, of frequency of 5-mers (as an example), and values of quantities such as 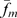, 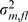, and 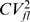 for two genomes with very different base compositions: *E. coli* (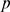 = 0.492) and *C. acetobutylicum* (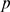 = 0.691). Here, a spectrum is the number of 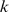-mers plotted against occurrence frequency. The spectra for the two genomes are shown as black curves in panels (A) and (B) of Fig. 9. The solid green curves characterized by narrow peaks are the spectra for random sequences obtained by scrambling the genomes. (The red curves are for sequences generated in the RSD model, see text.) In (A) the mean frequency of both spectra is 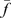 = 2
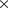
10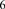/4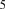 = 1953. However, the genomic spectrum is seen to be much broader then the random-sequence spectrum, indicating that whereas in the random sequence frequencies (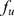) of individual 5-mers deviate little from the mean (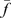), in the genomic sequence that is not the case; frequencies of individual 5-mers fluctuate widely around the mean. Drastically different from (A), the overall widths of genome and random-sequence spectra in (B) are similar. Instead of having a single peak, the random-sequence spectrum is composed of six widely spread narrow subspectra whose peaks are near the theoretical mean frequencies (for 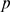 = 0.7) of the 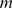-sets, 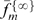 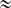
152, 354, 827, 1930, 4500, 10500, for 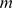 = 0 to 5, respectively. Eq. (6) shows that these mean values are determined by 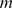 and the base composition of the sequence, or 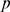, and does not depend on the fluctuation of frequencies of 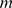-specific 5-mers. (B) and (C) in Fig. 9 show that in the random sequence frequency fluctuation within an 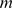-set is again small. In contrast, and just as in (A), frequency fluctuations of 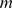 specific 5-mers in the genomic sequence are large (Fig. 9 (C) and Fig. 10 [70]).

Table 6 shows that 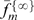 gives a very accurate estimate of 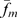 for random sequences and a fair one for genomic sequences. In the 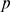 = 0.492 case, the relation 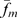 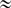 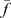 for all the 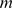
's explains the narrowness of the random spectrum in Fig. 9 (A): like its counterpart in (B), it is also composed of six subspectra, but unlike (B) whose subspectra are spread widely, now the subspectra are superimposed. Table 7 highlight important aspects of our formulation: (i) 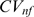 has a strong dependence on 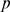 but not on whether a sequence is genomic or random; (ii) 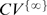 gives an excellent estimate of 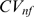 for random sequences, and a fair estimate for genomes; (iii) 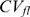 depends weakly on 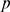 but strongly on whether a sequence is genomic (relative large value) or random (several orders of magnitude smaller, and much smaller than 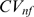 except when 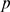 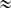
0.5). (iv) For random sequences Eq. (13) is a fairly accurate relation.

**Table 7:** Values of 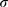
's from 5-mers in 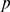 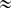
0.5 and 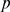 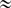
0.7 sequences.

	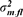 (in units of 10 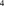 )						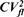	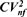	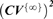
Sequence	( 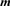 = ) 0	1	2	3	4	5			
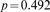									
*E. coli*	144	141	74.2	58.4	66.4	83.7	0.212	0.013	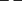
Random	.174	0.203	0.185	0.177	0.144	0.110	4.6 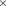 10 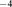	0.0012	0.0013
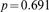									
*C. acetobutylicum*	0.60	6.95	26.1	65.4	97.1	336	0.145	1.00	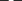
Random	0.011	0.038	0.102	0.218	0.500	1.24	5.8 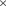 10 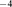	0.969	0.976

All sequences normalized to a length of 2 Mb; for 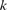 = 5, 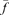 = 1953, 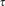 = 1024, and 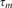 = 32, 160, 320, 160, 32, for 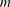 = 0 to 5.

### Equivalent length

The 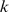-mers equivalent length of a sequence is defined as
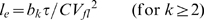
(14)where 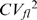 is given by the frequency distribution of 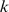-mers. Recalling that for a random sequence 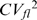 is inversely proportional sequence length (Eq. (13)), we see that 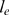 is the length of a random sequence whose 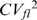 has the same value as that of the genome. The empirical factor 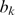 = 1−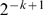, instead of the theoretical binomial factor 1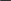 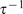, is used to ensure that for a random sequence, regardless of base composition, 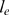 approximates the true sequence length with a high degree of accuracy. With the signal term 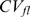 included but the strongly 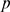-dependence background term 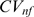 excluded in its definition, 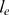 is expected to have at most a weak 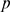-dependence. That is, 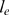 is a quantity with which we can compare on the same footing genomes with widely disparate base compositions.

### Genic, non-genic, exon, and intron concatenates

These various concatenates are formed by splicing corresponding sections from a single strand of the DNA sequence and them stitching the sections together in the order and orientation they appear in the sequence. In particular, the genic and exon concatenates include genetic codes in positive and negative orientations.

### Similarity index and similarity matrix

Given a pair of equal-length sequences 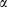 and 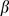, the similarity index 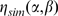 for the pair is defined as
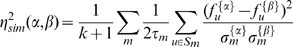
(15)where 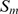 is an 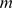-set and 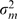 is the variance of the frequency of the 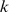-mers in 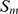. The pair are similar (in 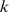-mer-content) when 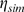 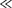
1, are (considered to be) identical when 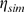 = 0, and are highly dissimilar when 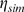 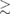
1. If we divide 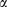 and 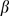 into (possibly overlapping) segments {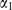,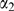,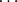} and {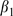,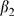,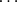}, respectively, then we call the matrix whose element (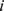,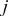) is valued 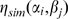 a similarity matrix. In Fig. 6, similarity matrices are displayed as similarity plots by color coding elements of similarity matrices.

### Minimum RSD model for genome growth

We denote by 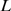 the designated length of a sequence and 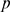 the designated AT-fraction of the sequence. We call the pair (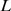, 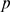) the *profile* of a sequence; in our model, the two profiles (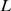, 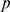) and (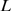, 1−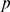) are mathematically equivalent. By a growth model we mean a computer algorithm for generating, from an initial sequence, a target sequence that has a given profile and other specific genome-like attributes. Ours is a model of random segmental duplication (RSD) [49] in which the three main steps are: (i) randomly select a site from the sequence, (ii) from that site cull a segment of random length (but from a given length distribution) for duplication; (iii) reinsert the duplicated segment into the sequence at a (second) randomly selected site. The model has three explicit parameters: 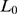, the initial sequence length; 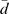, the average length of duplicated segments; 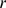, the cumulative point mutation density (replacement only), or number of mutations per site. The generation of a model sequence involves three steps: selection of initial sequence, growth by RSD, point mutations. An initial sequence (of length 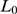) is chosen such that it has a target value 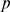 but is otherwise random. The lengths 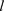 of the duplicated segments are selected with uniform probability within the range 1 to 2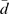, unless the current length of the genome 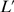 is less than 2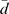, in which case 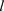 is selected from within the range 1 to 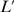. Growth is stopped when the length of the sequence exceeds the target length for the first time. Point mutations have a base bias defined by 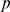 and are administered after the growth is complete. That is, the administration of point mutations on the sequence is not meant to emulate point mutations suffered by a genome during its growth. Rather, 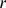 is meant to indicate the average cumulative number of point mutations per site experience by the genome throughout its life. Because RSD causes drifts in base composition, the profile of the generated sequence will have a profile that is a close approximation of, but not exactly equal to, the target profile.

### Mutation rates

We derive formulas for computing the rate density, or per site rate, of duplication events, 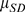, and the rate density of “point mutation” – including small deletion and insertion but excluding SD – events, 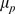. If the genome grows from time 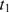 to time 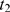 at a rate proportional to its length 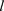, that is, 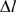 = 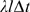 where 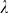 is the event rate (number of events per unit of time), then
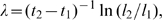
(16)If the grow is purely by SD and the average length of the duplicated segment is 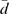, then
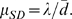
(17)


If 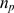 is the cumulative number of point mutations, then 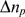 = 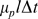. In SD dominated growth, the effect of point mutation on the overall length of a genome is negligible, so integrating the relation yields
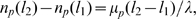
(18)For any 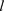 such that 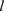 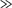 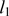, 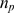 = 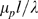. The cumulative mutation sites is greater than 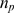 because mutation sites are copied during SD. The number of copied mutation sites satisfy 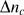 = 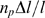 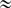 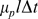 (for large 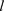). Therefore 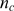 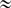 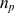, that is, the cumulative number of mutated sites is twice 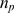. At full genome length 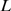, this number is 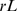, hence
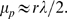
(19)


## Supporting Information

Figure S1 Category *L_e_* for coding and non-coding parts. Averages of *p* (fractional A/T-content) and *L_e_* for *k* = 7 (situations for other *k*s are similar) for the coding parts (solid symbols; *ex* for eukaryotes and *gn* for prokaryotes) and non-coding parts (hollow symbols; *in* for eukaryotes and *ig* for prokaryotes) of chromosomes. Symbols for categories are: vertebrates, red (square); unicellulars, blue (triangle-up); insects, orange (triangle-down); plants, green; prokaryotes, gray (bullet/circle). Numeral indicates number of chromosomes in each category. The curve represents *L_e_* for the universality class: *L_e_^{uc}^*(*k*; *p*).
(0.26 MB TIF)

Figure S2 Distributions of *χ^2^* versus *L* and *p*. Each symbol gives the *χ^2^* for one chromosomal *L_e_*. Top panels, for genic (*gn*) and exon (*ex*) concatenates. Bottom panels, for intergenic (*ig*) and intron (*in*) concatenates. Symbols, with color, number of data in group, and number of data whose *χ^2^* is less than 10^−3^ given in brackets, stand for: diamond, *gn* (blue; 7100; 229); square, *ex* (red; 2844, 95); triangle-down, *ig* (green; 6377, 270); triangle-up, *in* (orange; 2960, 104).
(0.77 MB TIF)

Figure S3 Results from minimal RSD model. Top-left: Equi-*χ^2^* contour as function of *r* and *d*, with *L_0_* = 64 (bases); length (*L*) of generated model sequence is 2 Mb and only *L_e_*(*k*) results for *k* = 7 are used. Top-right: *L_e_*(*k*), *k* = 2, 4, 6, 8, 10 from 200 model sequences generated using the “best” parameters *L_0_* = 64, <*d*> = 1000 (b) and *r* = 0.73 (cumulative point mutations per base). The lines are *L_e_^{uc}^*(*k*; *p*) that represent the universality class given in the main text. The *χ^2^* for the model sequences is 0.18. Bottom-left: *χ^2^* versus *L_0_* (otherwise best parameters); model sequences have *L* = 2 Mb and *p* = 0.5. Bottom-right: *L_e_* versus *L*, for a *p* = 0.5 model sequence generated using the best parameters.
(1.17 MB TIF)

Table S1 List of complete sequences included in the study (20 pp).
(0.13 MB PDF)

Table S2 Equivalent lengths of complete sequences (100 pp).
(0.36 MB PDF)

Table S3 *L_e_*(*k*), *k* = 2 to 10, averaged over categories of organisms.
(0.06 MB PDF)

Table S4 *L_e_* of sequences with highly biased compositions.
(0.06 MB PDF)

Table S5 Effect of replication and segmental duplication on *l_e_*.
(0.04 MB PDF)

Text S1 (0.07 MB PDF)
