# Supplementary figures and images for: Universal Global Imprints of Genome Growth and Evolution – Equivalent Length and Cumulative Mutation Density
Source: PLoS One. 2010 Apr 14;5(4):e9844. doi: 10.1371/journal.pone.0009844 (PMC2854691; doi:10.1371/journal.pone.0009844)

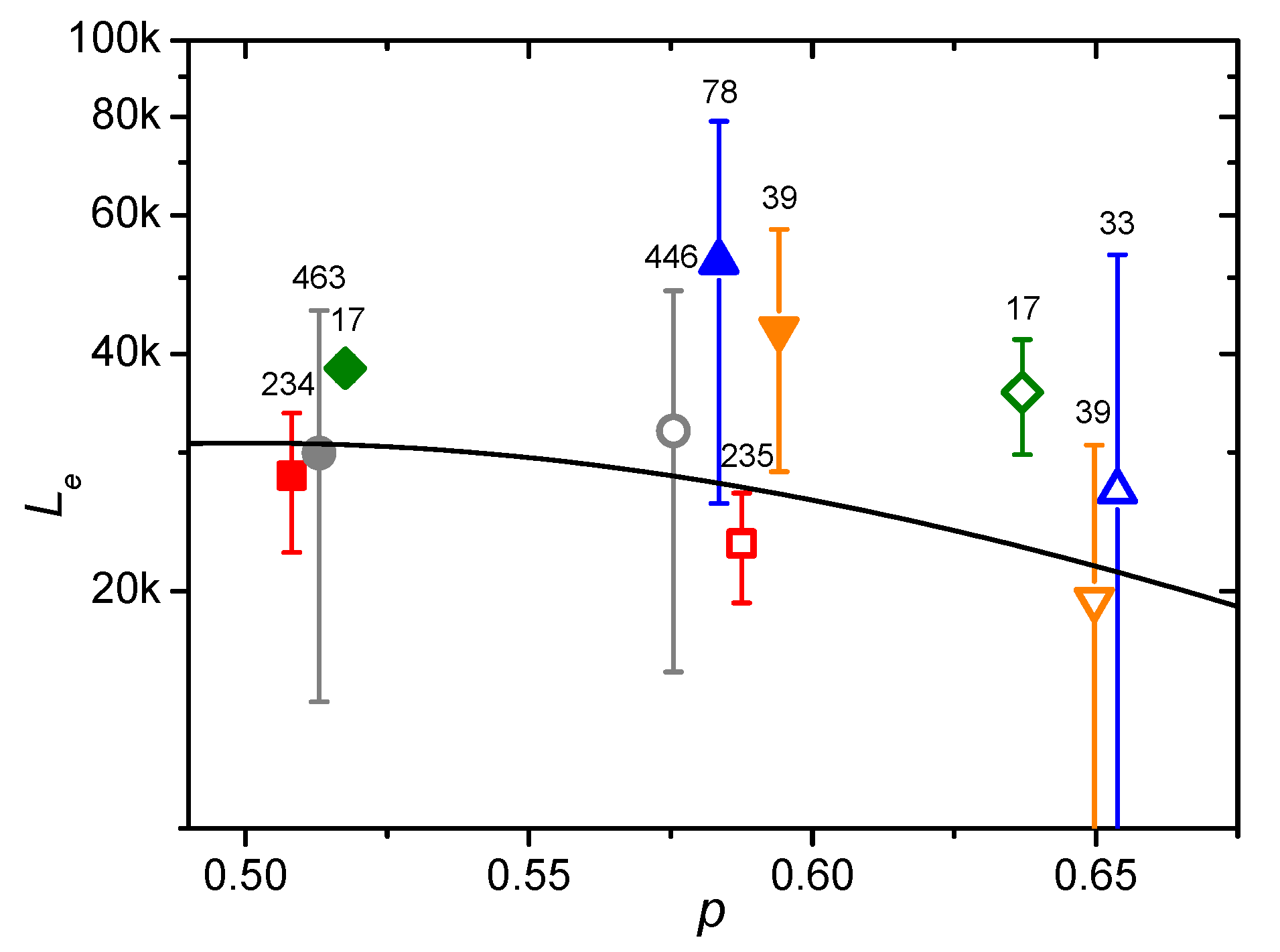

Supplement: Figure S1 — Category Le for coding and non-coding parts. Averages of p (fractional A/T-content) and Le for k = 7 (situations for other ks are similar) for the coding parts (solid symbols; ex for eukaryotes and gn for prokaryotes) and non-coding parts (hollow symbols; in for eukaryotes and ig for prokaryotes) of chromosomes. Symbols for categories are: vertebrates, red (square); unicellulars, blue (triangle-up); insects, orange (triangle-down); plants, green; prokaryotes, gray (bullet/circle). Numeral indicates number of chromosomes in each category. The curve represents Le for the universality class: Le{uc}(k; p). (0.26 MB TIF) [file pone.0009844.s001.tif]

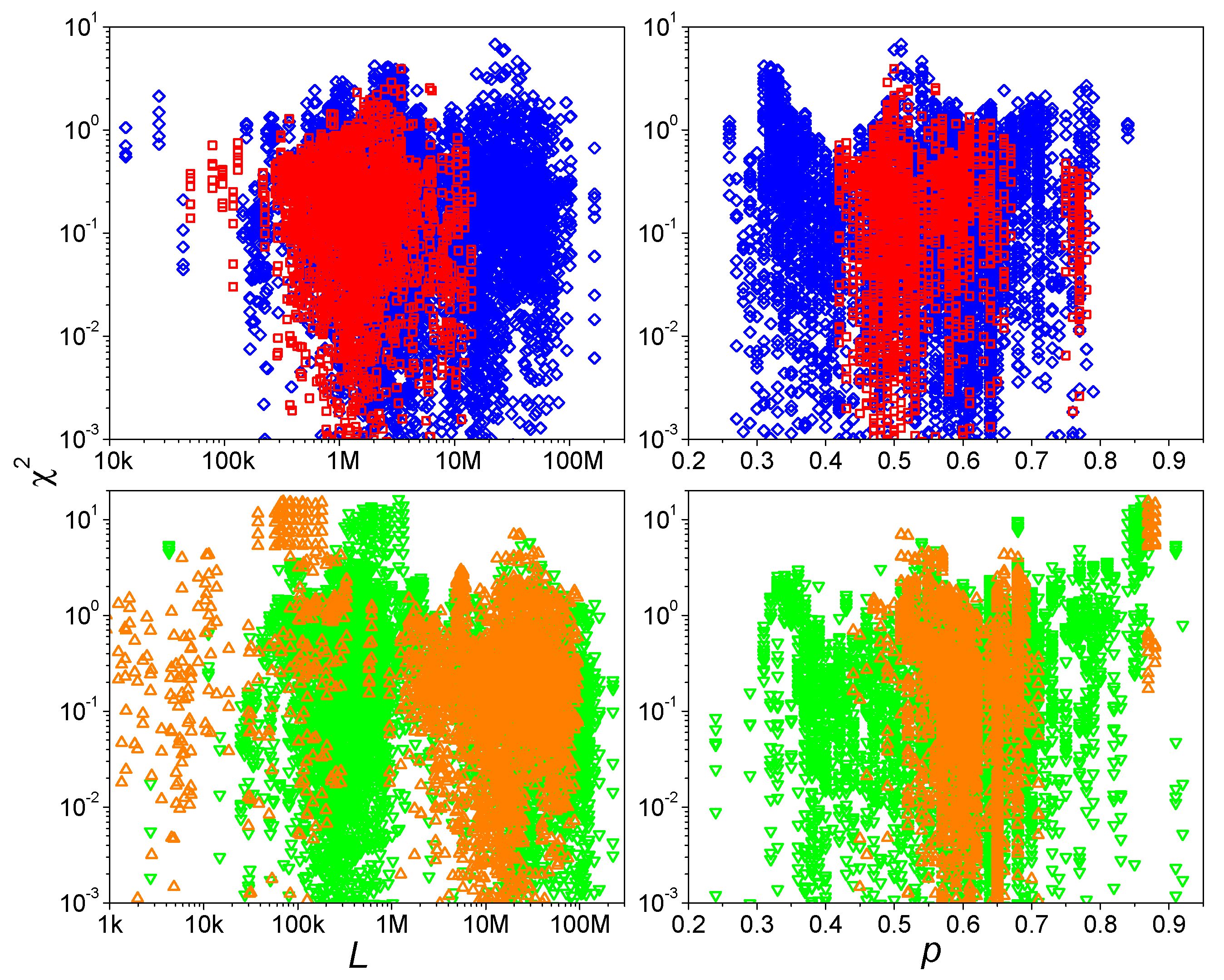

Supplement: Figure S2 — Distributions of χ2 versus L and p. Each symbol gives the χ2 for one chromosomal Le. Top panels, for genic (gn) and exon (ex) concatenates. Bottom panels, for intergenic (ig) and intron (in) concatenates. Symbols, with color, number of data in group, and number of data whose χ2 is less than 10−3 given in brackets, stand for: diamond, gn (blue; 7100; 229); square, ex (red; 2844, 95); triangle-down, ig (green; 6377, 270); triangle-up, in (orange; 2960, 104). (0.77 MB TIF) [file pone.0009844.s002.tif]

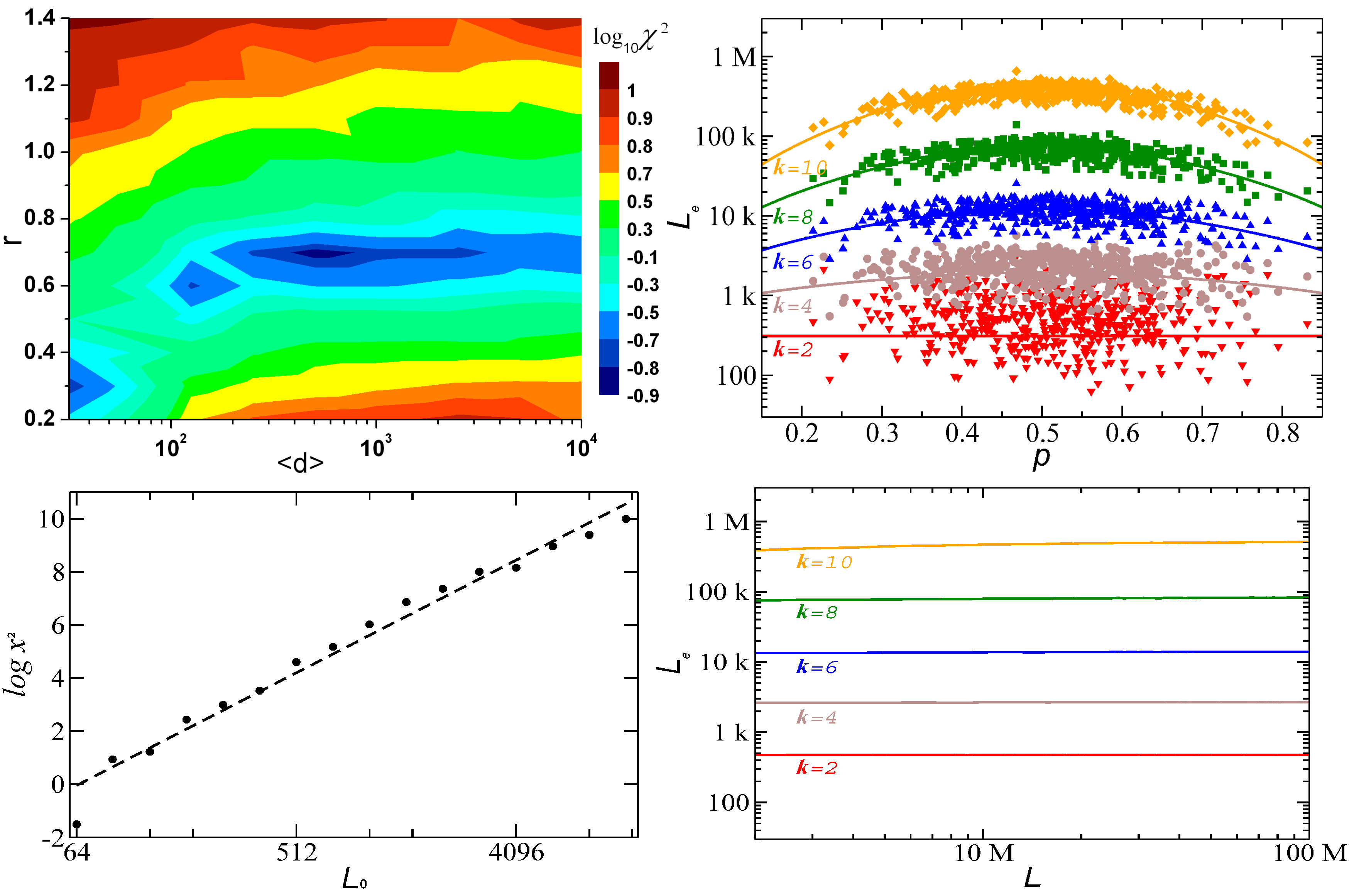

Supplement: Figure S3 — Results from minimal RSD model. Top-left: Equi-χ2 contour as function of r and d, with L0 = 64 (bases); length (L) of generated model sequence is 2 Mb and only Le(k) results for k = 7 are used. Top-right: Le(k), k = 2, 4, 6, 8, 10 from 200 model sequences generated using the “best” parameters L0 = 64, = 1000 (b) and r = 0.73 (cumulative point mutations per base). The lines are Le{uc}(k; p) that represent the universality class given in the main text. The χ2 for the model sequences is 0.18. Bottom-left: χ2 versus L0 (otherwise best parameters); model sequences have L = 2 Mb and p = 0.5. Bottom-right: Le versus L, for a p = 0.5 model sequence generated using the best parameters. (1.17 MB TIF) [file pone.0009844.s003.tif]
