# Supplementary material for: Universal Global Imprints of Genome Growth and Evolution – Equivalent Length and Cumulative Mutation Density
Source: PLoS One. 2010 Apr 14;5(4):e9844. doi: 10.1371/journal.pone.0009844 (PMC2854691; doi:10.1371/journal.pone.0009844)
Supplement: Table S1 — List of complete sequences included in the study (20 pp). (0.13 MB PDF) [file pone.0009844.s004.pdf]

Table S1. Complete sequences included in the study

The table is composed of five lists: List 1, 467 prokaryotes including 32 archaea and 435 bacteria; List 2, 106 unicells; List 3, 39 insects; List 4, 17 plants; List 5, 236 vertebrates. Columns in List 1 are: name of organism, NCBI accession number, and lengths and fractional A/T-content (p) of the chromosome and gene and intergenic concatenates. Columns in Lists 2 to 5 are: name of organism, NCBI accession number, and lengths and fractional A/T-content (p) of the chromosome, gene, intergenic, exon and intron concatenates. The combined length of the exon and intron concatenates are approximately equal to the length of the gene concatenate.

Sequences List 1: Pokaryotes (467).

Category	SN	L(mb),p		
		Chromosome	Gene	Intergenic
Archean (32)				
Aeropyrum pernix	NC_000854	1.66,0.44	1.48,0.43	0.18,0.5
Archaeoglobus fulgidus	NC_000917	2.17,0.52	2.02,0.51	0.15,0.63
Haloarcula marismortui ATCC 43049	NC_006396	3.13,0.38	2.71,0.37	0.41,0.44
Haloarcula marismortui ATCC 43049	NC_006397	0.28,0.43	0.22,0.42	0.06,0.48
Halobacterium sp	NC_002607	2.01,0.33	1.79,0.32	0.22,0.37
Methanobacterium thermoautotrophicum	NC_000916	1.75,0.51	1.59,0.5	0.15,0.62
Methanococcoides burtonii DSM 6242	NC_007955	2.57,0.6	2.2,0.58	0.36,0.68
Methanococcus jannaschii	NC_000909	1.66,0.69	1.47,0.68	0.18,0.75
Methanococcus jannaschii	NC_001732	0.05,0.72	0.04,0.71	0.01,0.75
Methanococcus jannaschii	NC_001733	0.01,0.72	0.01,0.71	*,0.74
Methanococcus maripaludis S2	NC_005791	1.66,0.67	1.49,0.66	0.16,0.78
Methanopyrus kandleri	NC_003551	1.69,0.39	1.51,0.39	0.18,0.4
Methanosaeta thermophila PT	NC_008553	1.87,0.47	1.58,0.46	0.29,0.53
Methanosarcina acetivorans	NC_003552	5.75,0.58	4.36,0.55	1.38,0.66
Methanosarcina barkeri fusaro	NC_007355	4.83,0.61	3.46,0.58	1.37,0.69
Methanosarcina mazei	NC_003901	4.09,0.59	3.08,0.56	1,0.68
Methanosphaera stadtmanae	NC_007681	1.76,0.73	1.5,0.71	0.26,0.83
Methanospirillum hungatei JF_1	NC_007796	3.54,0.55	3.14,0.54	0.4,0.63
Nanoarchaeum equitans	NC_005213	0.49,0.69	0.45,0.69	0.03,0.73
Natronomonas pharaonis	NC_007426	2.59,0.37	2.36,0.37	0.23,0.42
Picrophilus torridus DSM 9790	NC_005877	1.54,0.65	1.42,0.63	0.12,0.78
Pyrobaculum aerophilum	NC_003364	2.22,0.49	1.97,0.48	0.24,0.54
Pyrococcus abyssi	NC_000868	1.76,0.56	1.64,0.55	0.11,0.63
Pyrococcus furiosus	NC_003413	1.9,0.6	1.75,0.59	0.14,0.65
Pyrococcus horikoshii	NC_000961	1.73,0.59	1.61,0.58	0.12,0.65
Sulfolobus acidocaldarius DSM 639	NC_007181	2.22,0.64	1.92,0.63	0.3,0.7
Sulfolobus solfataricus	NC_002754	2.99,0.65	2.52,0.64	0.47,0.69
Sulfolobus tokodaii	NC_003106	2.69,0.68	2.24,0.67	0.44,0.72
Thermococcus kodakaraensis KOD1	NC_006624	2.08,0.49	1.92,0.48	0.16,0.59
Thermofilum pendens Hrk 5	NC_008698	1.78,0.43	1.62,0.42	0.15,0.49
Thermoplasma acidophilum	NC_002578	1.56,0.55	1.37,0.53	0.19,0.64
Thermoplasma volcanium	NC_002689	1.58,0.61	1.35,0.59	0.22,0.69
Bacterials (435)				
Acidobacteria bacterium Ellin345	NC_008009	5.65,0.42	5.05,0.42	0.59,0.47
Acidothermus cellulolyticus 11B	NC_008578	2.44,0.34	2.2,0.34	0.24,0.32
Acinetobacter sp ADP1	NC_005966	3.59,0.6	3.19,0.59	0.4,0.7
Aeromonas hydrophila ATCC 7966	NC_008570	4.74,0.39	4.2,0.38	0.54,0.48
Agrobacterium tumefaciens C58 Cereon	NC_003062	2.84,0.41	2.53,0.4	0.3,0.47
Agrobacterium tumefaciens C58 Cereon	NC_003063	2.07,0.41	1.89,0.41	0.17,0.47
Agrobacterium tumefaciens C58 UWash	NC_003304	2.84,0.41	2.5,0.4	0.33,0.47
Agrobacterium tumefaciens C58 UWash	NC_003305	2.07,0.41	1.87,0.41	0.2,0.47

next

Category	SN	L(mb),p		
		Chromosome	Gene	Intergenic
Alcanivorax borkumensis SK2	NC_008260	3.12,0.46	2.74,0.45	0.37,0.5
Alkalilimnicola ehrlichei MLHE_1	NC_008340	3.27,0.33	2.98,0.33	0.29,0.36
Anabaena variabilis ATCC 29413	NC_007413	6.36,0.59	5.22,0.58	1.14,0.64
Anaeromyxobacter dehalogenans 2CP_C	NC_007760	5.01,0.26	4.58,0.26	0.42,0.24
Anaplasma marginale St Maries	NC_004842	1.19,0.51	1.04,0.51	0.15,0.51
Anaplasma phagocytophilum HZ	NC_007797	1.47,0.59	1.06,0.58	0.4,0.62
Aquifex aeolicus	NC_000918	1.55,0.57	1.45,0.57	0.09,0.63
Arthrobacter FB24	NC_008541	4.69,0.35	4.24,0.35	0.45,0.39
Aster yellows witches_broom phytoplasma AYWB	NC_007716	0.7,0.74	0.52,0.72	0.17,0.8
Azoarcus sp EbN1	NC_006513	4.29,0.35	3.92,0.35	0.37,0.39
Bacillus anthracis Ames	NC_003997	5.22,0.65	4.47,0.64	0.75,0.7
Bacillus anthracis Ames 0581	NC_007530	5.22,0.65	4.48,0.64	0.74,0.7
Bacillus anthracis str Sterne	NC_005945	5.22,0.65	4.44,0.64	0.78,0.7
Bacillus cereus ATCC14579	NC_004722	5.41,0.65	4.56,0.64	0.84,0.7
Bacillus cereus ATCC 10987	NC_003909	5.22,0.65	4.52,0.64	0.69,0.7
Bacillus cereus ZK	NC_006274	5.3,0.65	4.51,0.64	0.78,0.7
Bacillus clausii KSM_K16	NC_006582	4.3,0.56	3.74,0.55	0.55,0.6
Bacillus halodurans	NC_002570	4.2,0.57	3.61,0.56	0.58,0.62
Bacillus licheniformis ATCC 14580	NC_006270	4.22,0.54	3.7,0.53	0.51,0.62
Bacillus licheniformis DSM 13	NC_006322	4.22,0.54	3.71,0.53	0.51,0.62
Bacillus subtilis	NC_000964	4.21,0.57	3.72,0.56	0.49,0.64
Bacillus thuringiensis Al Hakam	NC_008600	5.25,0.65	4.41,0.64	0.84,0.7
Bacillus thuringiensis konkukian	NC_005957	5.23,0.65	4.45,0.64	0.78,0.7
Bacteroides fragilis NCTC 9434	NC_003228	5.2,0.57	4.67,0.56	0.53,0.67
Bacteroides fragilis YCH46	NC_006347	5.27,0.57	4.77,0.56	0.5,0.67
Bacteroides thetaiotaomicron VPI_5482	NC_004663	6.26,0.58	5.62,0.57	0.63,0.67
Bartonella henselae Houston_1	NC_005956	1.93,0.62	1.48,0.6	0.45,0.68
Bartonella quintana Toulouse	NC_005955	1.58,0.62	1.24,0.6	0.33,0.67
Baumannia cicadellinicola Homalodisca coagulata	NC_007984	0.68,0.67	0.6,0.66	0.07,0.78
Bdellovibrio bacteriovorus	NC_005363	3.78,0.5	3.51,0.49	0.26,0.55
Bifidobacterium adolescentis ATCC 15703	NC_008618	2.08,0.41	1.83,0.4	0.25,0.48
Bifidobacterium longum	NC_004307	2.25,0.4	1.94,0.4	0.3,0.45
Bordetella bronchiseptica	NC_002927	5.33,0.32	4.93,0.32	0.4,0.37
Bordetella parapertussis	NC_002928	4.77,0.32	4.41,0.32	0.35,0.37
Bordetella pertussis	NC_002929	4.08,0.33	3.75,0.32	0.32,0.38
Borrelia afzelii PKo	NC_008277	0.9,0.72	0.85,0.72	0.05,0.79
Borrelia burgdorferi	NC_001318	0.91,0.72	0.85,0.71	0.05,0.79
Borrelia garinii PBi	NC_006156	0.9,0.72	0.84,0.72	0.05,0.79
Bradyrhizobium japonicum	NC_004463	9.1,0.36	7.9,0.36	1.2,0.41
Brucella abortus 9_941	NC_006932	2.12,0.43	1.86,0.42	0.26,0.51
Brucella abortus 9_941	NC_006933	1.16,0.43	1.04,0.42	0.11,0.52
Brucella melitensis	NC_003317	2.11,0.43	1.82,0.42	0.29,0.5
Brucella melitensis	NC_003318	1.17,0.43	1.03,0.42	0.14,0.5
Brucella melitensis biovar Abortus	NC_007618	2.12,0.43	1.84,0.42	0.27,0.5
Brucella melitensis biovar Abortus	NC_007624	1.15,0.43	1.03,0.42	0.12,0.51

next

Category	SN	L(mb),p		
		Chromosome	Gene	Intergenic
Brucella suis 1330	NC_004310	2.1,0.43	1.85,0.42	0.24,0.51
Brucella suis 1330	NC_004311	1.2,0.43	1.08,0.42	0.11,0.52
Buchnera aphidicola	NC_004545	0.61,0.75	0.51,0.73	0.1,0.85
Buchnera aphidicola Cc Cinara cedri	NC_008513	0.41,0.8	0.36,0.79	0.05,0.92
Buchnera aphidicola Sg	NC_004061	0.64,0.75	0.58,0.74	0.05,0.86
Buchnera sp	NC_002528	0.64,0.74	0.56,0.73	0.07,0.85
Burkholderia 383	NC_007509	1.39,0.35	1.2,0.34	0.19,0.4
Burkholderia 383	NC_007510	3.69,0.34	3.26,0.34	0.42,0.38
Burkholderia 383	NC_007511	3.58,0.34	3.14,0.33	0.43,0.38
Burkholderia cenocepacia AU 1054	NC_008060	3.29,0.34	2.92,0.33	0.37,0.37
Burkholderia cenocepacia AU 1054	NC_008061	2.78,0.34	2.44,0.33	0.34,0.38
Burkholderia cenocepacia AU 1054	NC_008062	1.19,0.33	1.06,0.33	0.13,0.38
Burkholderia cenocepacia HI2424	NC_008542	3.48,0.34	3.07,0.33	0.4,0.38
Burkholderia cenocepacia HI2424	NC_008543	2.99,0.34	2.63,0.33	0.36,0.38
Burkholderia cenocepacia HI2424	NC_008544	1.05,0.33	0.93,0.33	0.11,0.38
Burkholderia cepacia AMMD	NC_008390	3.55,0.34	3.13,0.33	0.42,0.37
Burkholderia cepacia AMMD	NC_008391	2.64,0.34	2.31,0.33	0.33,0.38
Burkholderia cepacia AMMD	NC_008392	1.28,0.34	1.12,0.33	0.15,0.39
Burkholderia mallei ATCC 23344	NC_006348	3.51,0.32	3.06,0.32	0.44,0.33
Burkholderia mallei ATCC 23344	NC_006349	2.32,0.32	2.02,0.31	0.3,0.33
Burkholderia pseudomallei 1710b	NC_007434	4.12,0.33	3.54,0.32	0.58,0.37
Burkholderia pseudomallei 1710b	NC_007435	3.18,0.32	2.74,0.32	0.43,0.35
Burkholderia pseudomallei K96243	NC_006350	4.07,0.33	3.43,0.33	0.63,0.34
Burkholderia pseudomallei K96243	NC_006351	3.17,0.32	2.63,0.32	0.53,0.34
Burkholderia thailandensis E264	NC_007650	2.91,0.32	2.53,0.32	0.38,0.35
Burkholderia thailandensis E264	NC_007651	3.8,0.33	3.31,0.33	0.48,0.36
Burkholderia xenovorans LB400	NC_007951	4.89,0.38	4.27,0.37	0.62,0.41
Burkholderia xenovorans LB400	NC_007952	3.36,0.38	2.96,0.37	0.4,0.42
Burkholderia xenovorans LB400	NC_007953	1.47,0.39	1.28,0.38	0.18,0.43
Campylobacter fetus 82_40	NC_008599	1.77,0.67	1.64,0.66	0.12,0.78
Campylobacter jejuni	NC_002163	1.64,0.7	1.56,0.69	0.07,0.81
Campylobacter jejuni RM1221	NC_003912	1.77,0.7	1.68,0.7	0.09,0.79
Candidatus Blochmannia floridanus	NC_005061	0.7,0.73	0.59,0.71	0.1,0.83
Candidatus Blochmannia pennsylvanicus BPEN	NC_007292	0.79,0.71	0.62,0.68	0.16,0.81
Candidatus Carsonella ruddii	NC_008512	0.15,0.84	0.15,0.84	*,0.91
Candidatus Carsonella ruddii PV	NC_008512	0.15,0.84	0.15,0.84	*,0.91
Candidatus Pelagibacter ubique HTCC1062	NC_007205	1.3,0.71	1.25,0.71	0.05,0.77
Candidatus Ruthia magnifica Cm Calyptogena magnifica	NC_008610	1.16,0.66	0.97,0.65	0.18,0.73
Carboxydothermus hydrogenoformans Z_2901	NC_007503	2.4,0.58	2.21,0.58	0.19,0.65
Caulobacter crescentus	NC_002696	4.01,0.33	3.65,0.33	0.35,0.38
Chlamydia muridarum	NC_002620	1.07,0.6	0.98,0.6	0.09,0.65
Chlamydia trachomatis	NC_000117	1.04,0.59	0.94,0.59	0.09,0.64
Chlamydia trachomatis A HAR_13	NC_007429	1.04,0.59	0.95,0.59	0.09,0.64
Chlamydophila abortus S26 3	NC_004552	1.14,0.61	1.04,0.6	0.1,0.68
Chlamydophila caviae	NC_003361	1.17,0.61	1.07,0.61	0.1,0.69

next

Category	SN	L(mb),p		
		Chromosome	Gene	Intergenic
Chlamydophila felis Fe C_56	NC_007899	1.16,0.61	1.06,0.6	0.09,0.68
Chlamydophila pneumoniae AR39	NC_002179	1.22,0.6	1.11,0.59	0.11,0.68
Chlamydophila pneumoniae CWL029	NC_000922	1.23,0.6	1.11,0.59	0.11,0.68
Chlamydophila pneumoniae J138	NC_002491	1.22,0.6	1.1,0.59	0.12,0.67
Chlamydophila pneumoniae TW 183	NC_005043	1.22,0.6	1.11,0.59	0.11,0.68
Chlorobium chlorochromatii CaD3	NC_007514	2.57,0.56	2.27,0.55	0.3,0.62
Chlorobium phaeobacteroides DSM 266	NC_008639	3.13,0.52	2.68,0.51	0.45,0.57
Chlorobium tepidum TLS	NC_002932	2.15,0.44	1.93,0.43	0.22,0.54
Chromobacterium violaceum	NC_005085	4.75,0.36	4.24,0.35	0.5,0.42
Chromohalobacter salexigens DSM 3043	NC_007963	3.69,0.37	3.35,0.36	0.34,0.41
Clostridium acetobutylicum	NC_003030	3.94,0.7	3.46,0.69	0.47,0.76
Clostridium novyi NT	NC_008593	2.54,0.72	2.25,0.71	0.28,0.78
Clostridium perfringens	NC_003366	3.03,0.72	2.58,0.71	0.45,0.8
Clostridium perfringens ATCC 13124	NC_008261	3.25,0.72	2.77,0.71	0.48,0.8
Clostridium perfringens SM101	NC_008262	2.89,0.72	2.38,0.71	0.51,0.77
Clostridium perfringens SM101	NC_008265	0.03,0.72	0.02,0.72	0.01,0.72
Clostridium tetani E88	NC_004557	2.79,0.72	2.42,0.71	0.37,0.76
Colwellia psychrerythraea 34H	NC_003910	5.37,0.62	4.61,0.62	0.76,0.68
Corynebacterium diphtheriae	NC_002935	2.48,0.47	2.25,0.46	0.23,0.53
Corynebacterium efficiens YS_314	NC_004369	3.14,0.37	2.87,0.37	0.27,0.44
Corynebacterium glutamicum ATCC 13032 Bielefeld	NC_006958	3.28,0.47	2.9,0.46	0.38,0.54
Corynebacterium glutamicum ATCC 13032 Kitasato	NC_003450	3.3,0.47	2.88,0.46	0.42,0.54
Corynebacterium jeikeium K411	NC_007164	2.46,0.39	2.21,0.38	0.24,0.47
Coxiella burnetii	NC_002971	1.99,0.58	1.79,0.58	0.2,0.6
Cyanobacteria bacterium Yellowstone A_Prime	NC_007775	2.93,0.4	2.54,0.39	0.38,0.46
Cyanobacteria bacterium Yellowstone B_Prime	NC_007776	3.04,0.42	2.62,0.41	0.42,0.48
Cytophaga hutchinsonii ATCC 33406	NC_008255	4.43,0.62	4,0.61	0.42,0.69
Dechloromonas aromatica RCB	NC_007298	4.5,0.41	4.15,0.41	0.34,0.49
Dehalococcoides CBDB1	NC_007356	1.39,0.53	1.26,0.53	0.13,0.62
Dehalococcoides ethenogenes 195	NC_002936	1.46,0.52	1.33,0.51	0.13,0.6
Deinococcus geothermalis DSM 11300	NC_008025	2.46,0.34	2.25,0.34	0.21,0.37
Deinococcus radiodurans	NC_001263	2.64,0.33	2.38,0.33	0.25,0.39
Deinococcus radiodurans	NC_001264	0.41,0.34	0.38,0.33	0.03,0.4
Desulfitobacterium hafniense Y51	NC_007907	5.72,0.53	4.88,0.52	0.84,0.6
Desulfotalea psychrophila LSv54	NC_006138	3.52,0.54	3.04,0.53	0.48,0.6
Desulfovibrio desulfuricans G20	NC_007519	3.73,0.43	3.39,0.42	0.33,0.45
Desulfovibrio vulgaris Hildenborough	NC_002937	3.57,0.37	3.11,0.37	0.45,0.39
Ehrlichia canis Jake	NC_007354	1.31,0.72	0.95,0.69	0.35,0.77
Ehrlichia chaffeensis Arkansas	NC_007799	1.17,0.7	0.94,0.69	0.23,0.76
Ehrlichia ruminantium Gardel	NC_006831	1.49,0.73	0.96,0.7	0.53,0.79
Ehrlichia ruminantium str. Welgevonden	NC_006832	1.51,0.73	0.96,0.7	0.54,0.79
Ehrlichia ruminantium Welgevonden	NC_005295	1.51,0.73	0.95,0.7	0.55,0.79
Enterococcus faecalis V583	NC_004668	3.21,0.63	2.85,0.62	0.35,0.68
Erwinia carotovora atroseptica SCRI1043	NC_004547	5.06,0.5	4.41,0.48	0.64,0.58
Erythrobacter litoralis HTCC2594	NC_007722	3.05,0.37	2.79,0.37	0.25,0.42

next

Category	SN	L(mb),p		
		Chromosome	Gene	Intergenic
Escherichia coli 536	NC_008253	4.93,0.5	4.4,0.49	0.53,0.59
Escherichia coli APEC O1	NC_008563	5.08,0.5	4.42,0.49	0.65,0.57
Escherichia coli CFT073	NC_004431	5.23,0.5	4.69,0.49	0.53,0.59
Escherichia coli K12	NC_000913	4.63,0.5	4.11,0.49	0.52,0.58
Escherichia coli O157H7	NC_002695	5.49,0.5	4.76,0.49	0.72,0.57
Escherichia coli O157H7 EDL933	NC_002655	5.52,0.5	4.85,0.49	0.66,0.58
Escherichia coli UTI89	NC_007946	5.06,0.5	4.55,0.49	0.51,0.58
Escherichia coli W3110	AC 000091	4.64,0.5	4.12,0.49	0.52,0.58
Francisella tularensis FSC 198	NC_008245	1.89,0.68	1.71,0.67	0.17,0.76
Francisella tularensis holarctica	NC_007880	1.89,0.68	1.68,0.67	0.21,0.75
Francisella tularensis holarctica OSU18	NC_008369	1.89,0.68	1.73,0.68	0.16,0.77
Francisella tularensis novicida U112	NC_008601	1.91,0.68	1.73,0.67	0.17,0.76
Francisella tularensis tularensis	NC_006570	1.89,0.68	1.71,0.67	0.17,0.76
Frankia alni ACN14a	NC_008278	7.49,0.28	6.47,0.27	1.01,0.29
Frankia CcI3	NC_007777	5.43,0.3	4.65,0.3	0.78,0.33
Fusobacterium nucleatum	NC_003454	2.17,0.73	1.95,0.73	0.21,0.78
Geobacillus kaustophilus HTA426	NC_006510	3.54,0.48	3.05,0.47	0.48,0.54
Geobacter metallireducens GS_15	NC_007517	3.99,0.41	3.66,0.4	0.33,0.5
Geobacter sulfurreducens	NC_002939	3.81,0.4	3.47,0.39	0.34,0.46
Gloeobacter violaceus	NC_005125	4.65,0.39	4.16,0.38	0.49,0.46
Gluconobacter oxydans 621H	NC_006677	2.7,0.39	2.44,0.39	0.25,0.45
Granulobacter bethesdensis CGDNIH1	NC_008343	2.7,0.41	2.49,0.41	0.21,0.48
Haemophilus ducreyi 35000HP	NC_002940	1.69,0.62	1.54,0.61	0.15,0.7
Haemophilus influenzae	NC_000907	1.83,0.62	1.64,0.61	0.18,0.7
Haemophilus influenzae 86 028NP	NC_007146	1.91,0.62	1.72,0.62	0.19,0.7
Haemophilus somnus 129PT	NC_008309	2,0.63	1.8,0.63	0.2,0.7
Hahella chejuensis KCTC 2396	NC_007645	7.21,0.47	6.36,0.46	0.84,0.54
Haloquadratum walsbyi	NC_008212	3.13,0.53	2.4,0.52	0.72,0.56
Helicobacter acinonychis Sheeba	NC_008229	1.55,0.62	1.4,0.62	0.15,0.69
Helicobacter hepaticus	NC_004917	1.79,0.65	1.68,0.64	0.11,0.71
Helicobacter pylori 26695	NC_000915	1.66,0.62	1.52,0.61	0.13,0.7
Helicobacter pylori HPAG1	NC_008086	1.59,0.61	1.47,0.61	0.12,0.7
Helicobacter pylori J99	NC_000921	1.64,0.61	1.48,0.61	0.15,0.68
Hyphomonas neptunium ATCC 15444	NC_008358	3.7,0.39	3.36,0.38	0.33,0.43
Idiomarina loihiensis L2TR	NC_006512	2.83,0.53	2.64,0.53	0.19,0.61
Jannaschia CCS1	NC_007802	4.31,0.38	3.92,0.38	0.39,0.42
Lactobacillus acidophilus NCFM	NC_006814	1.99,0.66	1.79,0.65	0.19,0.73
Lactobacillus brevis ATCC 367	NC_008497	2.29,0.54	1.99,0.53	0.29,0.61
Lactobacillus casei ATCC 334	NC_008526	2.89,0.54	2.48,0.53	0.41,0.58
Lactobacillus delbrueckii bulgaricus	NC_008054	1.86,0.51	1.64,0.49	0.22,0.63
Lactobacillus delbrueckii bulgaricus ATCC BAA_365	NC_008529	1.85,0.51	1.63,0.49	0.22,0.63
Lactobacillus gasseri ATCC 33323	NC_008530	1.89,0.65	1.73,0.65	0.15,0.72
Lactobacillus johnsonii NCC 533	NC_005362	1.99,0.66	1.81,0.65	0.18,0.73
Lactobacillus plantarum	NC_004567	3.3,0.56	2.82,0.55	0.48,0.63
Lactobacillus sakei 23K	NC_007576	1.88,0.59	1.73,0.58	0.15,0.69

next

Category	SN	L(mb),p		
		Chromosome	Gene	Intergenic
Lactobacillus salivarius UCC118	NC_007929	1.82,0.68	1.64,0.67	0.18,0.73
Lactococcus lactis	NC_002662	2.36,0.65	2.05,0.64	0.3,0.72
Lactococcus lactis cremoris SK11	NC_008527	2.43,0.65	2.13,0.64	0.3,0.73
Lawsonia intracellularis PHE MN1_00	NC_008011	1.45,0.67	1.22,0.66	0.23,0.75
Legionella pneumophila Lens	NC_006369	3.34,0.62	2.96,0.61	0.37,0.68
Legionella pneumophila Paris	NC_006368	3.5,0.62	3.1,0.61	0.39,0.68
Legionella pneumophila Philadelphia 1	NC_002942	3.39,0.62	3.01,0.62	0.38,0.68
Leifsonia xyli xyli CTCB0	NC_006087	2.58,0.33	2.11,0.32	0.47,0.36
Leptospira borgpetersenii serovar Hardjo_bovis JB197	NC_008510	3.57,0.6	2.91,0.59	0.66,0.65
Leptospira borgpetersenii serovar Hardjo_bovis JB197	NC_008511	0.29,0.6	0.24,0.59	0.05,0.65
Leptospira borgpetersenii serovar Hardjo_bovis L550	NC_008508	3.61,0.6	2.94,0.59	0.67,0.65
Leptospira borgpetersenii serovar Hardjo_bovis L550	NC_008509	0.31,0.6	0.26,0.59	0.05,0.65
Leptospira interrogans serovar Copenhageni	NC_005823	4.27,0.65	3.25,0.64	1.02,0.71
Leptospira interrogans serovar Copenhageni	NC_005824	0.35,0.66	0.26,0.64	0.08,0.71
Leptospira interrogans serovar Lai	NC_004342	4.33,0.65	3.38,0.64	0.94,0.71
Leptospira interrogans serovar Lai	NC_004343	0.35,0.65	0.28,0.64	0.07,0.71
Leuconostoc mesenteroides ATCC 8293	NC_008531	2.03,0.63	1.83,0.62	0.2,0.7
Listeria innocua	NC_003212	3.01,0.63	2.71,0.62	0.29,0.69
Listeria monocytogenes	NC_003210	2.94,0.63	2.65,0.62	0.28,0.69
Listeria monocytogenes 4b F2365	NC_002973	2.9,0.62	2.62,0.62	0.27,0.69
Listeria welshimeri serovar 6b SLCC5334	NC_008555	2.81,0.64	2.52,0.64	0.28,0.7
Magnetococcus MC_1	NC_008576	4.71,0.46	4.11,0.46	0.6,0.51
Magnetospirillum magneticum AMB_1	NC_007626	4.96,0.35	4.38,0.34	0.58,0.43
Mannheimia succiniciproducens MBEL55E	NC_006300	2.31,0.58	2.1,0.57	0.21,0.68
Maricaulis maris MCS10	NC_008347	3.36,0.38	3.04,0.37	0.32,0.41
Mesoplasma florum L1	NC_006055	0.79,0.73	0.74,0.73	0.04,0.79
Mesorhizobium BNC1	NC_008254	4.41,0.39	3.97,0.39	0.43,0.44
Mesorhizobium loti	NC_002678	7.03,0.38	6.09,0.37	0.93,0.43
Methylobacillus flagellatus KT	NC_007947	2.97,0.45	2.69,0.44	0.27,0.53
Methylococcus capsulatus Bath	NC_002977	3.3,0.37	2.99,0.37	0.31,0.41
Moorella thermoacetica ATCC 39073	NC_007644	2.62,0.45	2.31,0.44	0.31,0.53
Mycobacterium avium 104	NC_008595	5.47,0.32	5.04,0.31	0.42,0.36
Mycobacterium avium paratuberculosis	NC_002944	4.82,0.31	4.41,0.31	0.41,0.35
Mycobacterium bovis	NC_002945	4.34,0.35	3.95,0.35	0.38,0.38
Mycobacterium leprae	NC_002677	3.26,0.43	2.51,0.42	0.75,0.46
Mycobacterium MCS	NC_008146	5.7,0.32	5.33,0.32	0.37,0.37
Mycobacterium smegmatis MC2 155	NC_008596	6.98,0.33	6.52,0.33	0.46,0.39
Mycobacterium tuberculosis CDC1551	NC_002755	4.4,0.35	4.08,0.35	0.32,0.38
Mycobacterium tuberculosis H37Rv	NC_000962	4.41,0.35	4.01,0.35	0.39,0.38
Mycobacterium ulcerans Agy99	NC_008611	5.63,0.35	5.07,0.35	0.55,0.38
Mycoplasma capricolum ATCC 27343	NC_007633	1.01,0.77	0.91,0.76	0.09,0.82
Mycoplasma gallisepticum	NC_004829	0.99,0.69	0.9,0.68	0.09,0.75
Mycoplasma genitalium	NC_000908	0.58,0.69	0.53,0.69	0.04,0.7
Mycoplasma hyopneumoniae 232	NC_006360	0.89,0.72	0.8,0.71	0.08,0.81
Mycoplasma hyopneumoniae 7448	NC_007332	0.92,0.72	0.81,0.71	0.1,0.79

next

		L(mb),p		
Category	SN	Chromosome	Gene	Intergenic
Mycoplasma hyopneumoniae J	NC_007295	0.89,0.72	0.8,0.71	0.09,0.81
Mycoplasma mobile 163K	NC_006908	0.77,0.76	0.71,0.75	0.06,0.82
Mycoplasma mycoides	NC_005364	1.21,0.77	0.99,0.76	0.21,0.78
Mycoplasma penetrans	NC_004432	1.35,0.75	1.21,0.74	0.14,0.82
Mycoplasma pneumoniae	NC_000912	0.81,0.6	0.72,0.6	0.09,0.67
Mycoplasma pulmonis	NC_002771	0.96,0.74	0.87,0.73	0.08,0.82
Mycoplasma synoviae 53	NC_007294	0.79,0.72	0.73,0.71	0.06,0.78
Myxococcus xanthus DK 1622	NC_008095	9.13,0.32	8.35,0.31	0.78,0.34
Neisseria gonorrhoeae FA 1090	NC_002946	2.15,0.48	1.7,0.46	0.44,0.53
Neisseria meningitidis MC58	NC_003112	2.27,0.49	1.91,0.48	0.36,0.56
Neisseria meningitidis Z2491	NC_003116	2.18,0.49	1.83,0.47	0.35,0.56
Neorickettsia sennetsu Miyayama	NC_007798	0.85,0.59	0.75,0.59	0.09,0.63
Nitrobacter hamburgensis X14	NC_007964	4.4,0.39	3.71,0.38	0.69,0.43
Nitrobacter winogradskyi Nb_255	NC_007406	3.4,0.38	2.88,0.37	0.51,0.44
Nitrosococcus oceani ATCC 19707	NC_007484	3.48,0.5	3.02,0.49	0.45,0.57
Nitrosomonas europaea	NC_004757	2.81,0.5	2.48,0.49	0.32,0.56
Nitrosomonas eutropha C71	NC_008344	2.66,0.52	2.33,0.51	0.32,0.59
Nitrosospira multiformis ATCC 25196	NC_007614	3.18,0.47	2.73,0.46	0.45,0.52
Nocardia farcinica IFM10152	NC_006361	6.02,0.3	5.45,0.29	0.56,0.33
Nocardioides JS614	NC_008699	4.98,0.29	4.59,0.29	0.39,0.31
Nostoc sp	NC_003272	6.41,0.59	5.29,0.58	1.11,0.65
Novosphingobium aromaticivorans DSM 12444	NC_007794	3.56,0.35	3.28,0.35	0.27,0.4
Oceanobacillus iheyensis	NC_004193	3.63,0.65	3.14,0.64	0.49,0.68
Oenococcus oeni PSU_1	NC_008528	1.78,0.63	1.58,0.62	0.19,0.71
Onion yellows phytoplasma	NC_005303	0.86,0.73	0.63,0.71	0.22,0.77
Parachlamydia sp UWE25	NC_005861	2.41,0.66	1.99,0.64	0.41,0.73
Paracoccus denitrificans PD1222	NC_008686	2.85,0.34	2.6,0.33	0.25,0.38
Paracoccus denitrificans PD1222	NC_008687	1.73,0.34	1.57,0.33	0.15,0.38
Pasteurella multocida	NC_002663	2.25,0.6	2.03,0.59	0.21,0.68
Pediococcus pentosaceus ATCC 25745	NC_008525	1.83,0.63	1.65,0.62	0.18,0.7
Pelobacter carbinolicus	NC_007498	3.66,0.45	3.22,0.45	0.43,0.52
Pelobacter propionicus DSM 2379	NC_008609	4,0.41	3.58,0.41	0.42,0.48
Pelodictyon luteolum DSM 273	NC_007512	2.36,0.43	2.11,0.42	0.24,0.5
Photobacterium profundum SS9	NC_006370	4.08,0.59	3.43,0.57	0.65,0.64
Photobacterium profundum SS9	NC_006371	2.23,0.59	1.8,0.58	0.43,0.64
Photorhabdus luminescens	NC_005126	5.68,0.58	4.84,0.56	0.84,0.66
Pirellula sp	NC_005027	7.14,0.45	6.78,0.45	0.35,0.47
Polaromonas JS666	NC_007948	5.2,0.38	4.62,0.37	0.57,0.44
Porphyromonas gingivalis W83	NC_002950	2.34,0.52	1.99,0.51	0.34,0.59
Prochlorococcus marinus CCMP1375	NC_005042	1.75,0.64	1.56,0.63	0.18,0.7
Prochlorococcus marinus MED4	NC_005072	1.65,0.7	1.46,0.69	0.18,0.77
Prochlorococcus marinus MIT9313	NC_005071	2.41,0.5	1.98,0.48	0.42,0.57
Prochlorococcus marinus MIT 9312	NC_007577	1.7,0.69	1.53,0.68	0.17,0.77
Prochlorococcus marinus NATL2A	NC_007335	1.84,0.65	1.57,0.64	0.26,0.71
Propionibacterium acnes KPA171202	NC_006085	2.56,0.4	2.32,0.4	0.23,0.43

next

Category	SN	L(mb),p		
		Chromosome	Gene	Intergenic
Pseudoalteromonas atlantica T6c	NC_008228	5.18,0.56	4.52,0.55	0.65,0.62
Pseudoalteromonas haloplanktis TAC125	NC_007481	3.21,0.6	2.84,0.59	0.36,0.68
Pseudoalteromonas haloplanktis TAC125	NC_007482	0.63,0.61	0.55,0.6	0.08,0.68
Pseudomonas aeruginosa	NC_002516	6.26,0.34	5.62,0.33	0.64,0.39
Pseudomonas aeruginosa UCBPP_PA14	NC_008463	6.53,0.34	5.87,0.34	0.66,0.4
Pseudomonas entomophila L48	NC_008027	5.88,0.36	5.27,0.36	0.61,0.42
Pseudomonas fluorescens Pf_5	NC_004129	7.07,0.37	6.3,0.36	0.76,0.44
Pseudomonas fluorescens PfO_1	NC_007492	6.43,0.4	5.82,0.39	0.61,0.47
Pseudomonas putida KT2440	NC_002947	6.18,0.39	5.46,0.38	0.72,0.45
Pseudomonas syringae phaseolicola 1448A	NC_005773	5.92,0.42	5.22,0.42	0.7,0.49
Pseudomonas syringae pv B728a	NC_007005	6.09,0.41	5.39,0.41	0.69,0.47
Pseudomonas syringae tomato DC3000	NC_004578	6.39,0.42	5.59,0.41	0.8,0.48
Psychrobacter arcticum 273_4	NC_007204	2.65,0.58	2.17,0.56	0.47,0.66
Psychrobacter cryohalolentis K5	NC_007969	3.05,0.58	2.57,0.56	0.48,0.68
Ralstonia eutropha H16	NC_008313	4.05,0.34	3.57,0.33	0.48,0.38
Ralstonia eutropha H16	NC_008314	2.91,0.34	2.58,0.33	0.32,0.39
Ralstonia eutropha JMP134	NC_007347	3.8,0.36	3.4,0.35	0.39,0.4
Ralstonia eutropha JMP134	NC_007348	2.72,0.36	2.42,0.35	0.3,0.4
Ralstonia metallidurans CH34	NC_007973	3.92,0.37	3.53,0.36	0.39,0.41
Ralstonia metallidurans CH34	NC_007974	2.58,0.37	2.29,0.36	0.28,0.42
Ralstonia solanacearum	NC_003295	3.71,0.33	3.28,0.33	0.42,0.37
Rhizobium etli CFN 42	NC_007761	4.38,0.39	3.82,0.38	0.56,0.44
Rhizobium leguminosarum bv viciae 3841	NC_008380	5.05,0.39	4.41,0.39	0.64,0.46
Rhodobacter sphaeroides 2 4 1	NC_007493	3.18,0.31	2.85,0.31	0.32,0.32
Rhodobacter sphaeroides 2 4 1	NC_007494	0.94,0.31	0.83,0.31	0.11,0.33
Rhodococcus RHA1	NC_008268	7.8,0.33	7.13,0.33	0.67,0.37
Rhodoferax ferrireducens T118	NC_007908	4.71,0.41	4.27,0.4	0.43,0.48
Rhodopseudomonas palustris BisA53	NC_008435	5.5,0.36	4.75,0.36	0.74,0.4
Rhodopseudomonas palustris BisB18	NC_007925	5.51,0.36	4.75,0.35	0.75,0.39
Rhodopseudomonas palustris BisB5	NC_007958	4.89,0.36	4.27,0.35	0.62,0.4
Rhodopseudomonas palustris CGA009	NC_005296	5.45,0.35	4.78,0.35	0.67,0.39
Rhodopseudomonas palustris HaA2	NC_007778	5.33,0.34	4.67,0.34	0.66,0.37
Rhodospirillum rubrum ATCC 11170	NC_007643	4.35,0.35	3.86,0.34	0.48,0.4
Rickettsia bellii RML369_C	NC_007940	1.52,0.69	1.3,0.69	0.22,0.71
Rickettsia conorii	NC_003103	1.26,0.68	1.02,0.68	0.24,0.7
Rickettsia felis URRWXCal2	NC_007109	1.48,0.68	1.24,0.68	0.23,0.7
Rickettsia prowazekii	NC_000963	1.11,0.71	0.85,0.7	0.25,0.77
Rickettsia typhi wilmington	NC_006142	1.11,0.72	0.87,0.7	0.24,0.77
Roseobacter denitrificans OCh 114	NC_008209	4.13,0.42	3.73,0.41	0.39,0.46
Rubrobacter xylanophilus DSM 9941	NC_008148	3.22,0.3	3.01,0.3	0.2,0.36
Saccharophagus degradans 2_40	NC_007912	5.05,0.55	4.4,0.54	0.65,0.6
Salinibacter ruber DSM 13855	NC_007677	3.55,0.34	3.02,0.34	0.52,0.38
Salmonella enterica Choleraesuis	NC_006905	4.75,0.48	4.21,0.47	0.53,0.57
Salmonella enterica Paratypi ATCC 9150	NC_006511	4.58,0.48	4.02,0.47	0.56,0.57
Salmonella typhi	NC_003198	4.8,0.48	4.25,0.47	0.55,0.57

next

Category	SN	L(mb),p		
		Chromosome	Gene	Intergenic
Salmonella typhi Ty2	NC_004631	4.79,0.48	4.21,0.47	0.57,0.57
Salmonella typhimurium LT2	NC_003197	4.85,0.48	4.28,0.47	0.57,0.57
Shewanella amazonensis SB2B	NC_008700	4.3,0.47	3.84,0.46	0.46,0.54
Shewanella ANA_3	NC_008577	4.97,0.52	4.33,0.51	0.63,0.6
Shewanella denitrificans OS217	NC_007954	4.54,0.55	3.89,0.54	0.64,0.62
Shewanella frigidimarina NCIMB 400	NC_008345	4.84,0.59	4.14,0.58	0.69,0.65
Shewanella MR_4	NC_008321	4.7,0.53	4.09,0.51	0.6,0.6
Shewanella MR_7	NC_008322	4.79,0.53	4.16,0.51	0.62,0.6
Shewanella oneidensis	NC_004347	4.96,0.55	4.26,0.53	0.7,0.61
Shigella boydii Sb227	NC_007613	4.51,0.49	3.93,0.48	0.58,0.57
Shigella dysenteriae	NC_007606	4.36,0.49	3.75,0.48	0.61,0.56
Shigella flexneri 2a	NC_004337	4.6,0.5	4.07,0.49	0.53,0.57
Shigella flexneri 2a 2457T	NC_004741	4.59,0.5	3.97,0.48	0.62,0.57
Shigella flexneri 5 8401	NC_008258	4.57,0.5	3.99,0.48	0.58,0.57
Shigella sonnei Ss046	NC_007384	4.82,0.49	4.18,0.48	0.64,0.57
Silicibacter pomeroyi DSS_3	NC_003911	4.1,0.36	3.72,0.36	0.38,0.42
Silicibacter TM1040	NC_008044	3.2,0.4	2.85,0.4	0.34,0.43
Sinorhizobium meliloti	NC_003047	3.65,0.38	3.15,0.37	0.49,0.43
Sodalis glossinidius morsitans	NC_007712	4.17,0.46	2.15,0.44	2.01,0.47
Solibacter usitatus Ellin6076	NC_008536	9.96,0.39	9.11,0.38	0.85,0.44
Sphingopyxis alaskensis RB2256	NC_008048	3.34,0.35	3.04,0.35	0.29,0.39
Staphylococcus aureus aureus MRSA252	NC_002952	2.9,0.68	2.49,0.67	0.4,0.73
Staphylococcus aureus aureus MSSA476	NC_002953	2.79,0.68	2.39,0.67	0.4,0.73
Staphylococcus aureus COL	NC_002951	2.8,0.68	2.39,0.67	0.41,0.73
Staphylococcus aureus Mu50	NC_002758	2.87,0.68	2.43,0.67	0.44,0.73
Staphylococcus aureus MW2	NC_003923	2.82,0.68	2.38,0.67	0.43,0.73
Staphylococcus aureus N315	NC_002745	2.81,0.68	2.37,0.67	0.43,0.73
Staphylococcus aureus NCTC 8325	NC_007795	2.82,0.68	2.41,0.67	0.4,0.73
Staphylococcus aureus RF122	NC_007622	2.74,0.68	2.32,0.67	0.41,0.73
Staphylococcus aureus USA300	NC_007793	2.87,0.68	2.4,0.67	0.46,0.73
Staphylococcus epidermidis ATCC 12228	NC_004461	2.49,0.68	2.1,0.67	0.39,0.74
Staphylococcus epidermidis RP62A	NC_002976	2.61,0.68	2.23,0.67	0.38,0.74
Staphylococcus haemolyticus	NC_007168	2.68,0.68	2.33,0.67	0.35,0.73
Staphylococcus saprophyticus	NC_007350	2.51,0.67	2.13,0.66	0.38,0.73
Streptococcus agalactiae 2603	NC_004116	2.16,0.65	1.94,0.64	0.21,0.72
Streptococcus agalactiae A909	NC_007432	2.12,0.65	1.9,0.64	0.22,0.72
Streptococcus agalactiae NEM316	NC_004368	2.21,0.65	2.03,0.64	0.18,0.73
Streptococcus mutans	NC_004350	2.03,0.64	1.76,0.63	0.26,0.7
Streptococcus pneumoniae D39	NC_008533	2.04,0.61	1.78,0.6	0.25,0.68
Streptococcus pneumoniae R6	NC_003098	2.03,0.61	1.78,0.6	0.25,0.68
Streptococcus pneumoniae TIGR4	NC_003028	2.16,0.61	1.9,0.6	0.25,0.68
Streptococcus pyogenes M1 GAS	NC_002737	1.85,0.62	1.6,0.61	0.24,0.68
Streptococcus pyogenes MGAS10270	NC_008022	1.92,0.62	1.71,0.61	0.21,0.68
Streptococcus pyogenes MGAS10394	NC_006086	1.89,0.62	1.68,0.61	0.21,0.68
Streptococcus pyogenes MGAS10750	NC_008024	1.93,0.62	1.71,0.61	0.21,0.68

next

Category	SN	L(mb),p		
		Chromosome	Gene	Intergenic
Streptococcus pyogenes MGAS2096	NC_008023	1.86,0.62	1.64,0.61	0.21,0.68
Streptococcus pyogenes MGAS315	NC_004070	1.9,0.62	1.65,0.61	0.24,0.68
Streptococcus pyogenes MGAS5005	NC_007297	1.83,0.62	1.62,0.61	0.21,0.68
Streptococcus pyogenes MGAS6180	NC_007296	1.89,0.62	1.67,0.61	0.21,0.68
Streptococcus pyogenes MGAS8232	NC_003485	1.89,0.62	1.63,0.61	0.25,0.67
Streptococcus pyogenes MGAS9429	NC_008021	1.83,0.62	1.63,0.61	0.19,0.68
Streptococcus pyogenes SSI_1	NC_004606	1.89,0.62	1.63,0.61	0.26,0.67
Streptococcus thermophilus CNRZ1066	NC_006449	1.79,0.61	1.53,0.6	0.25,0.68
Streptococcus thermophilus LMD_9	NC_008532	1.85,0.61	1.59,0.6	0.26,0.68
Streptococcus thermophilus LMG 18311	NC_006448	1.79,0.61	1.53,0.6	0.25,0.68
Streptomyces avermitilis	NC_003155	9.02,0.3	7.8,0.29	1.22,0.32
Streptomyces coelicolor	NC_003888	8.66,0.28	7.76,0.28	0.9,0.31
Symbiobacterium thermophilum IAM14863	NC_006177	3.56,0.32	3.15,0.31	0.41,0.36
Synechococcus CC9311	NC_008319	2.6,0.48	2.27,0.47	0.33,0.55
Synechococcus CC9605	NC_007516	2.51,0.41	2.2,0.4	0.3,0.48
Synechococcus CC9902	NC_007513	2.23,0.46	2,0.46	0.22,0.53
Synechococcus elongatus PCC 6301	NC_006576	2.69,0.45	2.37,0.44	0.32,0.5
Synechococcus elongatus PCC 7942	NC_007604	2.69,0.45	2.4,0.44	0.28,0.5
Synechococcus sp WH8102	NC_005070	2.43,0.41	2.2,0.4	0.23,0.49
Synechocystis PCC6803	NC_000911	3.57,0.53	3.11,0.52	0.45,0.59
Syntrophobacter fumaroxidans MPOB	NC_008554	4.99,0.41	4.14,0.4	0.84,0.42
Syntrophomonas wolfei Goettingen	NC_008346	2.93,0.56	2.48,0.55	0.45,0.61
Syntrophus aciditrophicus SB	NC_007759	3.17,0.49	2.81,0.48	0.35,0.57
Thermoanaerobacter tengcongensis	NC_003869	2.68,0.63	2.45,0.62	0.23,0.68
Thermobifida fusca YX	NC_007333	3.64,0.33	3.12,0.32	0.51,0.37
Thermosynechococcus elongatus	NC_004113	2.59,0.47	2.33,0.46	0.26,0.52
Thermotoga maritima	NC_000853	1.86,0.54	1.77,0.54	0.08,0.6
Thermus thermophilus HB27	NC_005835	1.89,0.31	1.81,0.31	0.08,0.35
Thermus thermophilus HB8	NC_006461	1.84,0.31	1.77,0.31	0.07,0.35
Thiobacillus denitrificans ATCC 25259	NC_007404	2.9,0.34	2.69,0.34	0.2,0.38
Thiomicrospira crunogena XCL_2	NC_007520	2.42,0.57	2.2,0.56	0.22,0.66
Thiomicrospira denitrificans ATCC 33889	NC_007575	2.2,0.66	2.06,0.66	0.13,0.74
Treponema denticola ATCC 35405	NC_002967	2.84,0.63	2.63,0.62	0.2,0.69
Treponema pallidum	NC_000919	1.13,0.48	1.07,0.48	0.06,0.46
Trichodesmium erythraeum IMS101	NC_008312	7.75,0.66	4.94,0.64	2.8,0.71
Tropheryma whipplei TW08 27	NC_004551	0.92,0.54	0.79,0.54	0.13,0.56
Tropheryma whipplei Twist	NC_004572	0.92,0.54	0.8,0.54	0.12,0.56
Ureaplasma urealyticum	NC_002162	0.75,0.75	0.69,0.74	0.05,0.82
Vibrio cholerae	NC_002505	2.96,0.53	2.67,0.52	0.28,0.6
Vibrio cholerae	NC_002506	1.07,0.54	0.92,0.53	0.14,0.58
Vibrio fischeri ES114	NC_006840	2.9,0.62	2.56,0.61	0.34,0.68
Vibrio fischeri ES114	NC_006841	1.33,0.63	1.16,0.63	0.16,0.7
Vibrio parahaemolyticus	NC_004603	3.28,0.55	2.9,0.54	0.38,0.62
Vibrio parahaemolyticus	NC_004605	1.87,0.55	1.63,0.54	0.24,0.63
Vibrio vulnificus CMCP6	NC_004459	3.28,0.54	2.8,0.53	0.47,0.61

next

Category	SN	L(mb),p		
		Chromosome	Gene	Intergenic
Vibrio vulnificus CMCP6	NC_004460	1.84,0.53	1.58,0.52	0.25,0.61
Vibrio vulnificus YJ016	NC_005139	3.35,0.54	2.99,0.53	0.36,0.61
Vibrio vulnificus YJ016	NC_005140	1.85,0.53	1.66,0.52	0.19,0.61
Wigglesworthia brevipalpis	NC_004344	0.69,0.78	0.61,0.76	0.08,0.91
Wolbachia endosymbiont of Brugia malayi TRS	NC_006833	1.08,0.66	0.79,0.65	0.28,0.69
Wolbachia endosymbiont of Drosophila melanogaster	NC_002978	1.26,0.65	1.09,0.65	0.17,0.67
Wolinella succinogenes	NC_005090	2.11,0.52	1.99,0.52	0.11,0.61
Xanthomonas campestris	NC_003902	5.07,0.35	4.3,0.35	0.77,0.38
Xanthomonas campestris 8004	NC_007086	5.14,0.36	4.38,0.35	0.76,0.38
Xanthomonas campestris vesicatoria 85_10	NC_007508	5.17,0.36	4.53,0.35	0.64,0.38
Xanthomonas citri	NC_003919	5.17,0.36	4.45,0.35	0.71,0.38
Xanthomonas oryzae KACC10331	NC_006834	4.94,0.37	4.02,0.36	0.91,0.39
Xanthomonas oryzae MAFF 311018	NC_007705	4.94,0.37	4.15,0.36	0.78,0.4
Xylella fastidiosa	NC_002488	2.67,0.48	2.26,0.47	0.41,0.54
Xylella fastidiosa Temecula1	NC_004556	2.51,0.49	2.03,0.47	0.48,0.55
Yersinia pestis Antiqua	NC_008150	4.7,0.53	3.98,0.52	0.71,0.6
Yersinia pestis biovar Mediaevails	NC_005810	4.59,0.53	3.93,0.52	0.65,0.6
Yersinia pestis CO92	NC_003143	4.65,0.53	3.93,0.52	0.71,0.6
Yersinia pestis KIM	NC_004088	4.6,0.53	3.91,0.52	0.68,0.6
Yersinia pestis Nepal516	NC_008149	4.53,0.53	3.83,0.52	0.7,0.6
Yersinia pseudotuberculosis IP32953	NC_006155	4.74,0.53	4,0.52	0.73,0.6
Zymomonas mobilis ZM4	NC_006526	2.05,0.54	1.77,0.53	0.27,0.63

Sequences List 2: Unicell (106).

Category	SN	L(mb),p				
		Chromosome	Gene	Intergenic	Exon	Intron
Fungi (92)						
Aspergillus fumigatus	NC_007194	4.87,0.51	2.73,0.49	2.14,0.53	2.42,0.49	0.3,0.52
Aspergillus fumigatus	NC_007195	4.82,0.51	2.82,0.5	1.99,0.52	2.5,0.49	0.33,0.53
Aspergillus fumigatus	NC_007196	4.07,0.51	2.23,0.49	1.83,0.53	1.98,0.49	0.24,0.52
Aspergillus fumigatus	NC_007197	3.58,0.51	1.84,0.5	1.74,0.51	1.63,0.5	0.2,0.51
Aspergillus fumigatus	NC_007198	3.88,0.5	2.24,0.49	1.64,0.52	2,0.49	0.23,0.51
Aspergillus fumigatus	NC_007199	3.74,0.51	2.07,0.5	1.67,0.52	1.84,0.5	0.23,0.51
Aspergillus fumigatus	NC_007200	2.01,0.51	1.02,0.49	0.99,0.53	0.89,0.49	0.12,0.52
Aspergillus fumigatus	NC_007201	1.8,0.51	0.95,0.49	0.85,0.53	0.84,0.49	0.1,0.52
Candida albicans	NC_007436	0.94,0.67	0.58,0.66	0.36,0.69	0.58,0.66	*,0.69
Candida glabrata CBS138	NC_005967	0.48,0.61	0.3,0.58	0.17,0.65	0.29,0.58	*,0.68
Candida glabrata CBS138	NC_005968	0.5,0.62	0.32,0.6	0.17,0.65	0.31,0.6	*,0.62
Candida glabrata CBS138	NC_006026	0.55,0.61	0.35,0.59	0.2,0.64	0.34,0.59	*,0.68
Candida glabrata CBS138	NC_006027	0.65,0.61	0.42,0.6	0.22,0.65	0.41,0.6	*,0.69
Candida glabrata CBS138	NC_006028	0.68,0.62	0.44,0.6	0.23,0.65	0.43,0.6	*,0.67

next

Category	SN	L(mb),p				
		Chromosome	Gene	Intergenic	Exon	Intron
Candida glabrata CBS138	NC_006029	0.92,0.62	0.58,0.6	0.33,0.66	0.58,0.61	*,0.65
Candida glabrata CBS138	NC_006030	0.99,0.62	0.65,0.6	0.33,0.65	0.64,0.6	*,0.69
Candida glabrata CBS138	NC_006031	1.05,0.62	0.69,0.61	0.35,0.66	0.68,0.61	*,0.69
Candida glabrata CBS138	NC_006032	1.08,0.62	0.69,0.6	0.39,0.66	0.68,0.6	*,0.69
Candida glabrata CBS138	NC_006033	1.19,0.62	0.81,0.6	0.38,0.66	0.78,0.6	*,0.67
Candida glabrata CBS138	NC_006034	1.3,0.62	0.83,0.6	0.47,0.66	0.81,0.6	*,0.67
Candida glabrata CBS138	NC_006035	1.44,0.62	0.91,0.6	0.52,0.65	0.89,0.61	*,0.69
Candida glabrata CBS138	NC_006036	1.4,0.62	0.94,0.6	0.45,0.65	0.93,0.6	*,0.68
Cryptococcus neoformans var JEC21	NC_006670	2.3,0.52	1.75,0.51	0.54,0.54	1.42,0.5	0.28,0.56
Cryptococcus neoformans var JEC21	NC_006679	1.08,0.52	0.76,0.51	0.32,0.54	0.59,0.5	0.11,0.56
Cryptococcus neoformans var JEC21	NC_006680	1.01,0.52	0.77,0.51	0.24,0.54	0.61,0.5	0.12,0.57
Cryptococcus neoformans var JEC21	NC_006681	0.9,0.52	0.63,0.51	0.26,0.54	0.51,0.5	0.11,0.57
Cryptococcus neoformans var JEC21	NC_006682	0.78,0.52	0.57,0.51	0.21,0.54	0.43,0.5	0.1,0.57
Cryptococcus neoformans var JEC21	NC_006683	0.76,0.52	0.53,0.51	0.22,0.54	0.4,0.5	0.08,0.56
Cryptococcus neoformans var JEC21	NC_006684	1.63,0.52	1.22,0.51	0.4,0.55	0.97,0.5	0.2,0.57
Cryptococcus neoformans var JEC21	NC_006685	2.1,0.52	1.47,0.51	0.63,0.54	1.2,0.5	0.25,0.57
Cryptococcus neoformans var JEC21	NC_006686	1.78,0.52	1.35,0.51	0.42,0.54	1.11,0.5	0.22,0.56
Cryptococcus neoformans var JEC21	NC_006687	1.5,0.52	1.17,0.51	0.33,0.54	0.93,0.5	0.19,0.57
Cryptococcus neoformans var JEC21	NC_006691	1.43,0.52	1.08,0.51	0.35,0.55	0.85,0.5	0.18,0.57
Cryptococcus neoformans var JEC21	NC_006692	1.34,0.52	0.97,0.51	0.37,0.54	0.8,0.5	0.15,0.57
Cryptococcus neoformans var JEC21	NC_006693	1.19,0.52	0.85,0.51	0.33,0.54	0.66,0.5	0.14,0.57
Cryptococcus neoformans var JEC21	NC_006694	1.17,0.52	0.87,0.51	0.29,0.54	0.72,0.5	0.14,0.57
Debaryomyces hansenii CBS767	NC_006043	1.24,0.64	0.93,0.63	0.31,0.68	0.89,0.63	*,0.68
Debaryomyces hansenii CBS767	NC_006044	1.34,0.64	1,0.62	0.34,0.68	0.95,0.62	0.01,0.66
Debaryomyces hansenii CBS767	NC_006045	1.58,0.64	1.18,0.63	0.4,0.68	1.12,0.63	*,0.69
Debaryomyces hansenii CBS767	NC_006046	1.6,0.64	1.21,0.63	0.38,0.68	1.13,0.63	*,0.65
Debaryomyces hansenii CBS767	NC_006047	2.03,0.65	1.55,0.64	0.48,0.68	1.44,0.64	0.01,0.66
Debaryomyces hansenii CBS767	NC_006048	2.33,0.64	1.78,0.63	0.54,0.68	1.64,0.63	0.01,0.66
Debaryomyces hansenii CBS767	NC_006049	2.04,0.64	1.6,0.63	0.44,0.68	1.51,0.63	0.01,0.67
Encephalitozoon cuniculi	NC_003229	0.19,0.54	0.17,0.54	0.02,0.55	—	—
Encephalitozoon cuniculi	NC_003230	0.19,0.54	0.16,0.53	0.02,0.57	—	—
Encephalitozoon cuniculi	NC_003231	0.21,0.53	0.18,0.53	0.03,0.53	—	—
Encephalitozoon cuniculi	NC_003232	0.21,0.54	0.18,0.54	0.02,0.55	—	—
Encephalitozoon cuniculi	NC_003233	0.22,0.53	0.19,0.53	0.02,0.55	—	—
Encephalitozoon cuniculi	NC_003234	0.22,0.53	0.2,0.53	0.02,0.55	—	—
Encephalitozoon cuniculi	NC_003235	0.23,0.53	0.2,0.53	0.03,0.54	—	—
Encephalitozoon cuniculi	NC_003236	0.26,0.53	0.23,0.53	0.02,0.55	—	—
Encephalitozoon cuniculi	NC_003237	0.26,0.53	0.22,0.53	0.03,0.56	—	—
Encephalitozoon cuniculi	NC_003238	0.25,0.54	0.22,0.53	0.02,0.57	—	—
Encephalitozoon cuniculi	NC_003242	0.2,0.51	0.16,0.51	0.04,0.51	—	—
Eremothecium gossypii	NC_005782	0.69,0.49	0.54,0.48	0.14,0.53	0.54,0.48	*,0.5
Eremothecium gossypii	NC_005783	0.86,0.49	0.69,0.48	0.16,0.53	0.69,0.48	*,0.49
Eremothecium gossypii	NC_005784	0.9,0.47	0.73,0.47	0.17,0.51	0.72,0.47	*,0.44
Eremothecium gossypii	NC_005785	1.46,0.48	1.15,0.47	0.3,0.52	1.14,0.47	*,0.45
Eremothecium gossypii	NC_005786	1.51,0.49	1.2,0.48	0.3,0.52	1.2,0.48	*,0.49

next

Category	SN	L(mb),p				
		Chromosome	Gene	Intergenic	Exon	Intron
Eremothecium gossypii	NC_005787	1.81,0.49	1.46,0.48	0.35,0.52	1.44,0.48	*,0.49
Eremothecium gossypii	NC_005788	1.47,0.49	1.19,0.48	0.28,0.52	1.18,0.48	*,0.46
Kluyveromyces lactis NRRL Y_1140	NC_006037	1.06,0.61	0.74,0.6	0.31,0.65	0.73,0.6	*,0.64
Kluyveromyces lactis NRRL Y_1140	NC_006038	1.32,0.61	0.93,0.6	0.38,0.64	0.92,0.6	*,0.66
Kluyveromyces lactis NRRL Y_1140	NC_006039	1.75,0.62	1.25,0.61	0.49,0.65	1.24,0.61	*,0.67
Kluyveromyces lactis NRRL Y_1140	NC_006040	1.71,0.62	1.19,0.6	0.52,0.64	1.18,0.6	*,0.65
Kluyveromyces lactis NRRL Y_1140	NC_006041	2.23,0.62	1.58,0.6	0.64,0.65	1.57,0.6	0.01,0.66
Kluyveromyces lactis NRRL Y_1140	NC_006042	2.6,0.62	1.82,0.61	0.77,0.65	1.81,0.61	*,0.67
Saccharomyces cerevisiae	NC_001133	0.23,0.61	0.14,0.59	0.08,0.65	—	—
Saccharomyces cerevisiae	NC_001134	0.81,0.62	0.6,0.61	0.2,0.66	—	—
Saccharomyces cerevisiae	NC_001135	0.31,0.62	0.21,0.6	0.09,0.67	—	—
Saccharomyces cerevisiae	NC_001136	1.53,0.63	1.13,0.61	0.4,0.66	—	—
Saccharomyces cerevisiae	NC_001137	0.57,0.62	0.39,0.6	0.18,0.66	—	—
Saccharomyces cerevisiae	NC_001138	0.27,0.62	0.18,0.6	0.08,0.65	—	—
Saccharomyces cerevisiae	NC_001139	1.09,0.62	0.78,0.61	0.3,0.66	—	—
Saccharomyces cerevisiae	NC_001140	0.56,0.62	0.4,0.61	0.15,0.66	—	—
Saccharomyces cerevisiae	NC_001141	0.43,0.62	0.31,0.6	0.12,0.65	—	—
Saccharomyces cerevisiae	NC_001142	0.74,0.62	0.55,0.61	0.18,0.66	—	—
Saccharomyces cerevisiae	NC_001143	0.66,0.62	0.48,0.61	0.18,0.66	—	—
Saccharomyces cerevisiae	NC_001144	1.07,0.62	0.79,0.61	0.28,0.65	—	—
Saccharomyces cerevisiae	NC_001145	0.92,0.62	0.69,0.61	0.22,0.66	—	—
Saccharomyces cerevisiae	NC_001146	0.78,0.62	0.58,0.61	0.19,0.66	—	—
Saccharomyces cerevisiae	NC_001147	1.09,0.62	0.78,0.61	0.3,0.66	—	—
Saccharomyces cerevisiae	NC_001148	0.94,0.62	0.69,0.61	0.25,0.66	—	—
Schizosaccharomyces pombe	NC_003421	2.45,0.64	1.42,0.61	1.02,0.69	1.23,0.6	0.05,0.7
Schizosaccharomyces pombe	NC_003423	4.5,0.65	2.73,0.62	1.77,0.69	2.55,0.61	0.1,0.71
Schizosaccharomyces pombe	NC_003424	5.57,0.64	5.06,0.64	0.5,0.69	3.27,0.61	0.12,0.71
Yarrowia lipolytica CLIB99	NC_006067	2.3,0.52	1.03,0.47	1.26,0.56	0.98,0.47	0.01,0.52
Yarrowia lipolytica CLIB99	NC_006068	3.06,0.52	1.5,0.47	1.56,0.56	1.42,0.47	0.03,0.52
Yarrowia lipolytica CLIB99	NC_006069	3.27,0.52	1.41,0.47	1.86,0.55	1.33,0.47	0.03,0.52
Yarrowia lipolytica CLIB99	NC_006070	3.63,0.51	1.72,0.47	1.91,0.55	1.66,0.46	0.03,0.52
Yarrowia lipolytica CLIB99	NC_006071	4.22,0.51	2.17,0.47	2.04,0.55	2.1,0.47	0.02,0.52
Yarrowia lipolytica CLIB99	NC_006072	4,0.51	1.96,0.47	2.03,0.56	1.89,0.47	0.04,0.52
P. falciparum (13)						
Plasmodium falciparum	CHR_1	0.64,0.8	0.29,0.75	0.34,0.84	0.28,0.75	0.03,0.87
Plasmodium falciparum	CHR_2	0.94,0.81	0.52,0.77	0.42,0.85	0.46,0.76	0.05,0.87
Plasmodium falciparum	CHR_3	1.06,0.81	0.56,0.77	0.49,0.85	0.56,0.77	0.06,0.87
Plasmodium falciparum	CHR_4	1.2,0.8	0.66,0.76	0.54,0.85	0.64,0.75	0.06,0.88
Plasmodium falciparum	CHR_5	1.34,0.81	0.73,0.77	0.6,0.86	0.73,0.77	0.07,0.87
Plasmodium falciparum	CHR_6	1.37,0.81	0.75,0.77	0.61,0.86	0.75,0.77	0.08,0.87
Plasmodium falciparum	CHR_7	1.35,0.81	0.78,0.77	0.56,0.86	0.77,0.76	0.06,0.87
Plasmodium falciparum	CHR_8	1.32,0.81	0.71,0.77	0.6,0.86	0.7,0.77	0.08,0.87
Plasmodium falciparum	CHR_9	1.54,0.81	0.76,0.77	0.77,0.86	0.76,0.77	0.09,0.88
Plasmodium falciparum	CHR_10	1.69,0.81	0.95,0.77	0.73,0.85	0.84,0.76	0.11,0.87
Plasmodium falciparum	CHR_11	2.03,0.82	1.16,0.78	0.87,0.86	1.04,0.77	0.11,0.87

next

Category	SN	L(mb),p				
		Chromosome	Gene	Intergenic	Exon	Intron
Plasmodium falciparum	CHR_12	2.27,0.81	1.35,0.78	0.91,0.86	1.21,0.77	0.12,0.88
Plasmodium falciparum	CHR_13	2.72,0.81	1.53,0.77	1.18,0.86	1.53,0.77	0.15,0.88
Plasmodium falciparum	CHR_14	3.29,0.82	1.96,0.79	1.32,0.87	1.78,0.78	0.18,0.87

Sequences List 3: Insects (39).

Category	SN	L(mb),p				
		Chromosome	Gene	Intergenic	Exon	Intron
Anopheles gambiae	CHR_2	109.3,0.56	26.36,0.56	82.93,0.56	8.55,0.55	18.19,0.56
Anopheles gambiae	CHR_3	92.34,0.57	20.35,0.57	71.98,0.57	6.08,0.56	14.61,0.57
Anopheles gambiae	CHR_X	21.47,0.54	4.71,0.54	16.76,0.54	1.42,0.53	3.33,0.54
Apis mellifera	CHR_LG1	21.81,0.64	7.34,0.64	14.47,0.64	0.98,0.6	6.37,0.65
Apis mellifera	CHR_LG2	12.55,0.62	3.67,0.63	8.88,0.62	0.67,0.59	3.01,0.64
Apis mellifera	CHR_LG3	10.12,0.65	3.72,0.66	6.4,0.65	0.57,0.6	3.14,0.67
Apis mellifera	CHR_LG4	9.53,0.63	4.2,0.63	5.33,0.64	0.58,0.58	3.61,0.64
Apis mellifera	CHR_LG5	11.78,0.65	4.66,0.65	7.11,0.66	0.72,0.61	3.94,0.65
Apis mellifera	CHR_LG6	12.04,0.65	4.28,0.65	7.75,0.65	0.36,0.58	3.92,0.65
Apis mellifera	CHR_LG7	7.96,0.66	2.97,0.67	4.98,0.66	0.44,0.58	2.53,0.68
Apis mellifera	CHR_LG8	7.98,0.67	2.96,0.67	5.02,0.67	0.47,0.62	2.49,0.68
Apis mellifera	CHR_LG9	8.76,0.65	3.24,0.65	5.52,0.65	0.4,0.58	2.84,0.66
Apis mellifera	CHR_LG10	8.91,0.63	2.89,0.64	6.02,0.63	0.52,0.6	2.36,0.65
Apis mellifera	CHR_LG11	10.75,0.66	4.84,0.65	5.91,0.67	0.76,0.6	4.07,0.66
Apis mellifera	CHR_LG12	8.1,0.64	2.74,0.62	5.35,0.65	0.27,0.59	2.46,0.63
Apis mellifera	CHR_LG13	7.84,0.61	1.86,0.61	5.98,0.61	0.34,0.56	1.52,0.62
Apis mellifera	CHR_LG14	7.11,0.64	2.26,0.64	4.85,0.64	0.46,0.59	1.8,0.65
Apis mellifera	CHR_LG15	6.13,0.64	1.86,0.65	4.26,0.64	0.44,0.59	1.42,0.67
Apis mellifera	CHR_LG16	5.05,0.66	2.11,0.66	2.94,0.65	0.21,0.58	1.89,0.67
Caenorhabditis elegans	CHR_I	15.07,0.65	9.93,0.64	5.13,0.66	4.34,0.58	5.76,0.68
Caenorhabditis elegans	CHR_II	15.27,0.64	9.27,0.63	6,0.66	4.63,0.58	4.89,0.68
Caenorhabditis elegans	CHR_III	13.78,0.65	9.09,0.64	4.68,0.66	3.97,0.58	5.42,0.68
Caenorhabditis elegans	CHR_IV	17.49,0.66	9.62,0.64	7.86,0.68	4.41,0.59	5.35,0.69
Caenorhabditis elegans	CHR_V	20.92,0.65	12.03,0.64	8.88,0.67	6.2,0.6	6.04,0.68
Caenorhabditis elegans	CHR_X	17.71,0.65	8.84,0.64	8.87,0.67	4.04,0.58	4.9,0.68
Drosophila melanogaster	CHR_2	43.17,0.58	24.61,0.56	18.56,0.6	10.66,0.51	14.96,0.6
Drosophila melanogaster	CHR_3	51.67,0.58	29.49,0.56	22.18,0.6	12.33,0.5	18.33,0.6
Drosophila melanogaster	CHR_4	1.28,0.65	0.85,0.65	0.42,0.66	0.31,0.61	0.57,0.68
Drosophila melanogaster	CHR_X	22.21,0.58	11.87,0.56	10.34,0.6	4.98,0.49	7.34,0.61
Tribolium castaneum	CHR_LG01=X	5.55,0.66	2.77,0.66	2.77,0.66	0.63,0.65	2.05,0.66
Tribolium castaneum	CHR_LG02	9.4,0.65	4.78,0.64	4.61,0.65	1.2,0.63	3.25,0.64
Tribolium castaneum	CHR_LG03	22.41,0.68	12.09,0.68	10.32,0.68	1.57,0.66	9.96,0.68
Tribolium castaneum	CHR_LG04	11.54,0.65	5.34,0.65	6.19,0.65	1.39,0.64	3.23,0.65
Tribolium castaneum	CHR_LG05	13.85,0.65	7.03,0.64	6.81,0.65	1.86,0.63	4.8,0.64
Tribolium castaneum	CHR_LG06	8.28,0.68	4.08,0.67	4.19,0.68	1,0.66	2.84,0.68
Tribolium castaneum	CHR_LG07	13.02,0.65	7.22,0.65	5.8,0.65	1.76,0.64	5.05,0.65
Tribolium castaneum	CHR_LG08	11.5,0.67	5.42,0.66	6.07,0.67	1.38,0.64	3.9,0.67

next

Category	SN	L(mb),p				
		Chromosome	Gene	Intergenic	Exon	Intron
Tribolium castaneum	CHR_LG09	10.76,0.66	5.52,0.65	5.24,0.66	1.31,0.64	4.04,0.66
Tribolium castaneum	CHR_LG10	5.79,0.69	3.07,0.69	2.71,0.69	0.46,0.67	2.44,0.69

Sequences List 4: Plant (17).

Category	SN	L(mb),p				
		Chromosome	Gene	Intergenic	Exon	Intron
Arabidopsis thaliana	CHR_I	30.26,0.65	17.1,0.61	13.16,0.69	10.33,0.58	5.1,0.68
Arabidopsis thaliana	CHR_II	19.7,0.65	10.74,0.61	8.95,0.69	5.89,0.58	2.92,0.68
Arabidopsis thaliana	CHR_III	23.46,0.64	13.47,0.61	9.99,0.69	7.78,0.58	3.68,0.68
Arabidopsis thaliana	CHR_IV	18.58,0.64	10.51,0.61	8.06,0.69	5.98,0.58	3.03,0.68
Arabidopsis thaliana	CHR_V	26.97,0.65	14.66,0.61	12.31,0.69	9.18,0.58	4.46,0.68
Oryza sativa	CHR_01	43.58,0.57	20.08,0.54	23.53,0.59	13.99,0.49	13.19,0.62
Oryza sativa	CHR_02	35.92,0.57	16.53,0.55	19.42,0.59	11.49,0.49	10.72,0.62
Oryza sativa	CHR_03	36.33,0.57	17.26,0.54	19.12,0.59	12.23,0.49	11.43,0.62
Oryza sativa	CHR_04	35.23,0.56	16.22,0.54	19.06,0.58	10.85,0.49	9.15,0.61
Oryza sativa	CHR_05	29.86,0.57	13.5,0.54	16.4,0.59	9.43,0.49	8.07,0.62
Oryza sativa	CHR_06	31.22,0.57	13.62,0.54	17.56,0.59	9.14,0.49	7.92,0.62
Oryza sativa	CHR_07	29.68,0.57	13.08,0.54	16.58,0.59	8.76,0.49	7.48,0.62
Oryza sativa	CHR_08	28.3,0.57	12.4,0.54	15.91,0.59	8.26,0.49	7.31,0.62
Oryza sativa	CHR_09	23,0.57	10.07,0.54	12.91,0.59	6.55,0.49	5.85,0.62
Oryza sativa	CHR_10	22.86,0.57	9.87,0.54	13,0.59	6.69,0.49	5.54,0.62
Oryza sativa	CHR_11	28.45,0.58	12.35,0.55	16.06,0.6	7.95,0.5	6.76,0.62
Oryza sativa	CHR_12	27.49,0.57	11.78,0.55	15.71,0.6	7.8,0.5	6.73,0.62

Sequences List 5: Vertebrates (236).

Category	SN	L(mb),p				
		Chromosome	Gene	Intergenic	Exon	Intron
Bos taurus	CHR_01	81.96,0.6	21.74,0.59	60.22,0.6	1.2,0.52	20.61,0.59
Bos taurus	CHR_02	69.6,0.59	14.71,0.61	29.89,0.61	1.5,0.5	22.11,0.59
Bos taurus	CHR_03	70.03,0.58	23.45,0.58	46.58,0.58	1.73,0.49	21.85,0.58
Bos taurus	CHR_04	56.06,0.59	19.43,0.59	36.63,0.59	0.94,0.5	18.53,0.59
Bos taurus	CHR_05	61.94,0.57	21.58,0.57	40.35,0.58	1.63,0.47	20.07,0.57
Bos taurus	CHR_06	55.28,0.6	14.12,0.6	41.16,0.6	0.72,0.52	13.44,0.6
Bos taurus	CHR_07	56.56,0.57	19.37,0.54	37.19,0.58	1.82,0.45	17.71,0.55
Bos taurus	CHR_08	48.83,0.59	14.74,0.58	34.08,0.59	0.77,0.49	14.02,0.58
Bos taurus	CHR_09	50.58,0.6	14.35,0.6	36.23,0.6	0.69,0.53	13.7,0.6
Bos taurus	CHR_10	57.91,0.58	19.91,0.59	38,0.58	1.32,0.5	18.67,0.59
Bos taurus	CHR_11	70.92,0.57	23.28,0.56	47.64,0.58	1.6,0.48	21.79,0.57
Bos taurus	CHR_12	39.56,0.59	9.28,0.58	30.28,0.59	0.55,0.5	8.76,0.59
Bos taurus	CHR_13	51.46,0.56	15.6,0.56	35.86,0.57	1.12,0.47	14.55,0.56
Bos taurus	CHR_14	40.81,0.59	10.33,0.58	30.48,0.59	0.67,0.49	9.68,0.58
Bos taurus	CHR_15	43.12,0.58	12.92,0.56	30.2,0.58	1,0.47	11.96,0.57

next

Category	SN	L(mb),p				
		Chromosome	Gene	Intergenic	Exon	Intron
Bos taurus	CHR_16	47.47,0.57	14.83,0.57	32.63,0.58	1.01,0.48	13.87,0.57
Bos taurus	CHR_17	37.55,0.57	13.97,0.55	23.57,0.57	0.99,0.46	13.04,0.56
Bos taurus	CHR_18	46.63,0.54	16.68,0.53	29.95,0.55	1.71,0.44	15.05,0.54
Bos taurus	CHR_19	48.01,0.54	18.99,0.53	29.02,0.55	2.03,0.45	17.14,0.54
Bos taurus	CHR_20	34.49,0.59	9.39,0.59	25.09,0.59	0.43,0.52	8.99,0.59
Bos taurus	CHR_21	39.29,0.57	11.39,0.56	27.9,0.57	0.85,0.47	10.6,0.56
Bos taurus	CHR_22	39.38,0.57	14.27,0.56	25.11,0.58	0.95,0.46	13.4,0.57
Bos taurus	CHR_23	34.25,0.57	10.66,0.56	23.58,0.57	1,0.46	9.7,0.57
Bos taurus	CHR_24	36.44,0.58	9.55,0.58	26.88,0.59	0.52,0.51	9.02,0.58
Bos taurus	CHR_25	32.86,0.54	11.91,0.52	20.95,0.55	1.24,0.42	10.76,0.53
Bos taurus	CHR_26	28.83,0.57	9.39,0.57	19.44,0.58	0.63,0.49	8.79,0.57
Bos taurus	CHR_27	24.95,0.59	6.04,0.58	18.91,0.59	0.35,0.5	5.7,0.59
Bos taurus	CHR_28	28.32,0.58	8.85,0.58	19.47,0.58	0.51,0.51	8.38,0.58
Bos taurus	CHR_29	35.85,0.56	9.49,0.53	26.36,0.56	0.91,0.44	8.64,0.54
Bos taurus	CHR_X	39.93,0.59	9.97,0.58	29.95,0.59	0.69,0.49	9.33,0.59
Canis familiaris	CHR_01	121.61,0.59	42.25,0.58	79.35,0.59	2.2,0.47	40.12,0.59
Canis familiaris	CHR_02	84.34,0.58	31.65,0.58	52.68,0.58	1.57,0.48	30.15,0.58
Canis familiaris	CHR_03	91.19,0.6	27.5,0.59	63.68,0.61	1.09,0.5	26.46,0.59
Canis familiaris	CHR_04	88.14,0.6	28.92,0.59	59.21,0.61	1.2,0.5	27.81,0.6
Canis familiaris	CHR_05	88.46,0.56	31.9,0.55	56.56,0.57	1.95,0.45	30.06,0.56
Canis familiaris	CHR_06	76.9,0.58	28.71,0.57	48.19,0.59	1.71,0.46	27.1,0.57
Canis familiaris	CHR_07	80.3,0.6	32.72,0.59	47.57,0.6	1.38,0.5	31.41,0.6
Canis familiaris	CHR_08	73.88,0.6	25.12,0.59	48.75,0.6	1.26,0.5	23.96,0.6
Canis familiaris	CHR_09	60.58,0.55	30.19,0.54	30.39,0.55	2.2,0.45	28.14,0.55
Canis familiaris	CHR_10	68.87,0.58	25.26,0.57	43.61,0.58	1.22,0.48	24.11,0.57
Canis familiaris	CHR_11	73.82,0.6	23.53,0.59	50.28,0.61	1.04,0.51	22.57,0.6
Canis familiaris	CHR_12	72.22,0.62	24.95,0.61	47.26,0.62	1.18,0.5	23.85,0.61
Canis familiaris	CHR_13	62.63,0.61	19.67,0.6	42.96,0.61	0.79,0.5	18.93,0.61
Canis familiaris	CHR_14	60.58,0.62	20.67,0.61	39.9,0.62	0.72,0.52	19.99,0.62
Canis familiaris	CHR_15	63.71,0.6	20.99,0.6	42.71,0.6	0.98,0.51	20.07,0.61
Canis familiaris	CHR_16	58.75,0.6	17.32,0.59	41.43,0.6	0.82,0.48	16.54,0.59
Canis familiaris	CHR_17	64,0.59	22.14,0.58	41.86,0.59	1.19,0.49	21.02,0.59
Canis familiaris	CHR_18	55.22,0.58	20.08,0.56	35.13,0.59	1.31,0.46	18.84,0.57
Canis familiaris	CHR_19	53.53,0.62	12.54,0.62	40.99,0.62	0.41,0.54	12.15,0.62
Canis familiaris	CHR_20	57.66,0.56	26.19,0.55	31.46,0.58	1.8,0.43	24.48,0.55
Canis familiaris	CHR_21	50.7,0.6	18.95,0.6	31.75,0.61	0.85,0.5	18.11,0.6
Canis familiaris	CHR_22	61.17,0.63	13.45,0.61	47.72,0.63	0.44,0.52	13.03,0.61
Canis familiaris	CHR_23	52.05,0.61	19.3,0.6	32.75,0.61	0.67,0.52	18.68,0.61
Canis familiaris	CHR_24	47.36,0.56	15.77,0.55	31.58,0.56	0.91,0.45	14.86,0.56
Canis familiaris	CHR_25	51.07,0.59	19.3,0.59	31.76,0.59	0.81,0.49	18.53,0.59
Canis familiaris	CHR_26	38.67,0.55	15.3,0.55	23.36,0.55	0.9,0.45	14.45,0.55
Canis familiaris	CHR_27	45.63,0.6	17.2,0.6	28.43,0.61	0.96,0.49	16.29,0.61
Canis familiaris	CHR_28	40.89,0.57	16.59,0.57	24.29,0.57	0.76,0.48	15.86,0.58
Canis familiaris	CHR_29	41.63,0.62	11.26,0.62	30.36,0.62	0.39,0.55	10.89,0.62
Canis familiaris	CHR_30	39.95,0.59	17.12,0.59	22.82,0.59	0.9,0.5	16.28,0.6

next

		L(mb),p				
Category	SN	Chromosome	Gene	Intergenic	Exon	Intron
Canis familiaris	CHR_31	38.84,0.61	9.29,0.58	29.54,0.62	0.38,0.48	8.92,0.59
Canis familiaris	CHR_32	38.65,0.64	12.56,0.63	26.08,0.64	0.45,0.54	12.14,0.64
Canis familiaris	CHR_33	31.17,0.61	10.18,0.6	20.99,0.62	0.43,0.52	9.76,0.6
Canis familiaris	CHR_34	41.87,0.6	13.06,0.6	28.81,0.6	0.47,0.5	12.61,0.6
Canis familiaris	CHR_35	26.36,0.59	6.69,0.59	19.67,0.59	0.29,0.48	6.41,0.59
Canis familiaris	CHR_36	30.67,0.62	10.89,0.62	19.78,0.62	0.52,0.55	10.4,0.63
Canis familiaris	CHR_37	30.72,0.6	10.96,0.6	19.76,0.6	0.5,0.5	10.48,0.61
Canis familiaris	CHR_38	23.66,0.6	6.95,0.58	16.71,0.61	0.37,0.47	6.59,0.58
Canis familiaris	CHR_X	122.24,0.6	34.72,0.6	87.52,0.61	1.61,0.5	33.2,0.6
Danio rerio	CHR_01	58.77,0.64	25.32,0.64	33.46,0.64	1.87,0.53	23.52,0.65
Danio rerio	CHR_02	49.49,0.64	20.8,0.64	28.7,0.64	1.63,0.53	19.19,0.65
Danio rerio	CHR_03	42.35,0.64	17.47,0.64	24.88,0.64	1.53,0.53	15.98,0.65
Danio rerio	CHR_04	32.12,0.65	13.98,0.65	18.14,0.64	1.21,0.53	12.79,0.66
Danio rerio	CHR_05	64.5,0.64	27.95,0.64	36.56,0.64	2.19,0.53	25.82,0.65
Danio rerio	CHR_06	31.51,0.65	15.27,0.64	16.24,0.65	1.09,0.53	14.2,0.65
Danio rerio	CHR_07	59.41,0.64	25.67,0.64	33.74,0.64	1.91,0.53	23.81,0.65
Danio rerio	CHR_08	35.44,0.64	17.34,0.64	18.09,0.64	1.17,0.53	16.22,0.65
Danio rerio	CHR_09	41.78,0.64	18.78,0.64	23,0.64	1.55,0.53	17.3,0.65
Danio rerio	CHR_10	34.87,0.64	15.81,0.64	19.06,0.64	1.3,0.53	14.62,0.65
Danio rerio	CHR_11	35.2,0.64	14.36,0.64	20.83,0.64	0.98,0.52	13.42,0.65
Danio rerio	CHR_12	33.16,0.64	14.7,0.64	18.45,0.64	1.16,0.53	13.6,0.65
Danio rerio	CHR_13	35.7,0.64	16.03,0.64	19.66,0.64	1.29,0.53	14.76,0.65
Danio rerio	CHR_14	56.96,0.64	21.89,0.64	35.06,0.64	1.77,0.53	20.19,0.65
Danio rerio	CHR_15	34.53,0.64	14.08,0.64	20.44,0.64	1.18,0.53	12.93,0.65
Danio rerio	CHR_16	40.61,0.64	17.18,0.64	23.42,0.64	1.39,0.53	15.82,0.65
Danio rerio	CHR_17	40.25,0.64	18.13,0.64	22.11,0.64	1.39,0.53	16.76,0.65
Danio rerio	CHR_18	45.56,0.64	18.63,0.64	26.93,0.64	1.42,0.53	17.26,0.65
Danio rerio	CHR_19	48.95,0.64	19.23,0.64	29.72,0.64	1.46,0.53	17.83,0.65
Danio rerio	CHR_20	57.25,0.64	23.95,0.64	33.29,0.64	2.01,0.53	22.01,0.65
Danio rerio	CHR_21	38.61,0.64	16.08,0.64	22.53,0.64	1.16,0.53	14.97,0.65
Danio rerio	CHR_22	35.07,0.64	14.89,0.64	20.17,0.64	1.35,0.53	13.42,0.65
Danio rerio	CHR_23	40.33,0.64	16.74,0.64	23.58,0.64	1.41,0.52	15.37,0.65
Danio rerio	CHR_24	24.29,0.64	10.43,0.64	13.86,0.64	0.77,0.53	9.68,0.65
Danio rerio	CHR_25	24.5,0.64	9.42,0.64	15.08,0.64	0.71,0.53	8.72,0.65
Gallus gallus	CHR_01	184.99,0.61	80.57,0.6	104.41,0.61	4.45,0.53	74.82,0.61
Gallus gallus	CHR_02	143.84,0.61	61.63,0.61	82.2,0.61	3.15,0.54	58.1,0.61
Gallus gallus	CHR_03	107.45,0.61	50.29,0.6	57.15,0.61	2.97,0.52	47.16,0.61
Gallus gallus	CHR_04	89.01,0.61	37.01,0.6	52,0.61	2.48,0.52	34.37,0.6
Gallus gallus	CHR_05	54.07,0.6	26.66,0.59	27.4,0.6	2.06,0.52	24.52,0.6
Gallus gallus	CHR_06	33.39,0.59	17.64,0.59	15.75,0.59	1.2,0.51	16.04,0.6
Gallus gallus	CHR_07	35.4,0.59	17.37,0.59	18.02,0.59	1.31,0.52	15.87,0.6
Gallus gallus	CHR_08	28.18,0.59	14.75,0.58	13.42,0.59	1.19,0.5	13.55,0.59
Gallus gallus	CHR_09	23.05,0.58	11.24,0.58	11.81,0.58	0.99,0.5	10.24,0.58
Gallus gallus	CHR_10	22.46,0.58	11.66,0.58	10.8,0.58	1,0.51	10.64,0.59
Gallus gallus	CHR_11	19.1,0.59	8.17,0.57	10.93,0.6	0.76,0.49	7.19,0.58

next

Category	SN	L(mb),p Chromosome	Gene	Intergenic	Exon	Intron
Gallus gallus	CHR_12	19.04,0.57	10.63,0.57	8.4,0.57	0.82,0.49	9.71,0.58
Gallus gallus	CHR_13	17.9,0.56	8.47,0.56	9.42,0.57	0.77,0.48	7.66,0.57
Gallus gallus	CHR_14	20.15,0.56	11.51,0.56	8.63,0.57	1.12,0.48	10.39,0.57
Gallus gallus	CHR_15	12.22,0.55	6.46,0.56	5.75,0.55	0.78,0.49	5.68,0.57
Gallus gallus	CHR_16	0.43,0.46	0.23,0.45	0.2,0.48	0.07,0.42	0.15,0.47
Gallus gallus	CHR_17	9.89,0.53	5.73,0.53	4.16,0.53	0.66,0.47	5.06,0.54
Gallus gallus	CHR_18	8.79,0.55	4.41,0.55	4.38,0.54	0.5,0.49	3.91,0.56
Gallus gallus	CHR_19	9.31,0.54	5.94,0.54	3.37,0.54	0.7,0.48	5.24,0.55
Gallus gallus	CHR_20	13.29,0.55	6.5,0.55	6.79,0.55	0.75,0.47	5.73,0.55
Gallus gallus	CHR_21	6.04,0.53	3.42,0.54	2.61,0.53	0.49,0.48	2.93,0.55
Gallus gallus	CHR_22	2.18,0.57	1.34,0.57	0.84,0.57	0.09,0.49	1.24,0.57
Gallus gallus	CHR_23	5.03,0.51	2.4,0.51	2.62,0.51	0.43,0.45	1.97,0.52
Gallus gallus	CHR_24	5.87,0.51	3.03,0.52	2.84,0.51	0.36,0.46	2.66,0.52
Gallus gallus	CHR_26	3.66,0.5	2.06,0.5	1.6,0.5	0.35,0.46	1.69,0.51
Gallus gallus	CHR_27	3.19,0.51	1.88,0.5	1.31,0.51	0.33,0.45	1.54,0.52
Gallus gallus	CHR_28	4.04,0.53	2.42,0.51	1.62,0.54	0.38,0.45	2.04,0.53
Gallus gallus	CHR_32	1.01,0.48	0.74,0.47	0.27,0.49	0.13,0.43	0.61,0.48
Gallus gallus	CHR_W	4.36,0.61	1.86,0.62	2.5,0.61	0.11,0.57	1.74,0.62
Gallus gallus	CHR_Z	45.17,0.61	19.16,0.61	26.01,0.61	1.17,0.54	17.21,0.61
Homo sapiens	CHR_01	226.05,0.59	103.05,0.58	123.15,0.59	6.38,0.56	94.85,0.58
Homo sapiens	CHR_02	237.87,0.6	95.12,0.6	142.77,0.61	4.35,0.59	88.03,0.6
Homo sapiens	CHR_03	195.4,0.61	165.37,0.6	225.23,0.61	3.58,0.58	77.43,0.6
Homo sapiens	CHR_04	187.94,0.62	62.74,0.62	125.19,0.63	2.57,0.6	58.92,0.61
Homo sapiens	CHR_05	177.84,0.61	62.57,0.6	115.27,0.62	3.01,0.58	57.84,0.6
Homo sapiens	CHR_06	169.14,0.61	65.93,0.6	103.16,0.61	3.17,0.59	61.57,0.6
Homo sapiens	CHR_07	155.38,0.6	70.72,0.59	84.67,0.6	2.86,0.57	65.29,0.59
Homo sapiens	CHR_08	143.2,0.6	52.12,0.6	91.07,0.61	2.21,0.57	48.67,0.6
Homo sapiens	CHR_09	118.73,0.59	47.1,0.58	73.87,0.6	2.48,0.56	42.72,0.58
Homo sapiens	CHR_10	131.72,0.59	59.28,0.59	72.45,0.59	2.59,0.57	55.09,0.59
Homo sapiens	CHR_11	131.13,0.59	53.91,0.58	77.33,0.6	3.4,0.56	49.26,0.58
Homo sapiens	CHR_12	130.72,0.6	56.67,0.59	73.63,0.6	3.15,0.57	52.38,0.59
Homo sapiens	CHR_13	95.74,0.62	31.41,0.61	64.33,0.62	1.22,0.59	29.27,0.61
Homo sapiens	CHR_14	88.29,0.6	35.73,0.59	52.55,0.6	1.86,0.57	32.22,0.59
Homo sapiens	CHR_15	81.92,0.58	38.98,0.58	42.93,0.59	2.35,0.57	35,0.58
Homo sapiens	CHR_16	78.99,0.56	35.6,0.54	43.38,0.57	2.47,0.52	31.94,0.55
Homo sapiens	CHR_17	79.6,0.55	40.55,0.54	39.04,0.56	3.4,0.52	36.26,0.54
Homo sapiens	CHR_18	74.66,0.61	26.62,0.6	48.03,0.61	1.01,0.59	25.19,0.6
Homo sapiens	CHR_19	56.03,0.52	27.3,0.5	28.73,0.54	3.43,0.5	23.19,0.51
Homo sapiens	CHR_20	59.5,0.56	26.35,0.56	33.14,0.57	1.52,0.54	24.35,0.56
Homo sapiens	CHR_21	34.17,0.6	12.31,0.57	23.13,0.61	0.68,0.55	11.23,0.57
Homo sapiens	CHR_22	34.97,0.53	19.17,0.52	15.88,0.53	1.42,0.51	16.42,0.52
Homo sapiens	CHR_X	151.87,0.61	46.27,0.6	106.26,0.61	2.51,0.58	42.42,0.6
Homo sapiens	CHR_Y	24.87,0.61	5.47,0.6	20.17,0.61	0.22,0.58	3.42,0.6
Macaca mulatta	CHR_01	219.57,0.59	93.59,0.58	125.98,0.59	6.09,0.56	88.06,0.58
Macaca mulatta	CHR_02	186.01,0.61	72.19,0.6	113.82,0.62	3.39,0.58	69.01,0.6

next

Category	SN	L(mb),p				
		Chromosome	Gene	Intergenic	Exon	Intron
Macaca mulatta	CHR_03	180.61,0.6	69.51,0.59	111.09,0.6	3.38,0.56	66.17,0.59
Macaca mulatta	CHR_04	165.55,0.61	58.89,0.6	106.66,0.61	3.17,0.58	55.89,0.6
Macaca mulatta	CHR_05	178.8,0.63	54.49,0.62	124.3,0.63	2.38,0.61	52.33,0.62
Macaca mulatta	CHR_06	173.74,0.61	59.14,0.6	114.59,0.61	3.11,0.58	56.16,0.6
Macaca mulatta	CHR_07	162.96,0.59	66.23,0.59	96.73,0.59	4.03,0.56	62.54,0.59
Macaca mulatta	CHR_08	139.56,0.6	46.68,0.6	92.87,0.61	2.15,0.57	44.7,0.6
Macaca mulatta	CHR_09	126.87,0.59	50.41,0.59	76.45,0.59	2.46,0.57	48.2,0.59
Macaca mulatta	CHR_10	91.1,0.55	37.62,0.55	53.47,0.55	2.63,0.52	35.16,0.55
Macaca mulatta	CHR_11	133.01,0.6	54.59,0.59	78.42,0.6	3.36,0.57	51.37,0.59
Macaca mulatta	CHR_12	103.33,0.61	41.38,0.61	61.95,0.62	2.02,0.59	39.49,0.61
Macaca mulatta	CHR_13	128.79,0.59	43.96,0.59	84.83,0.6	2.24,0.57	41.91,0.59
Macaca mulatta	CHR_14	123.02,0.59	50.26,0.58	72.75,0.59	3.3,0.55	47.3,0.58
Macaca mulatta	CHR_15	106.87,0.59	38.74,0.58	68.12,0.6	2.34,0.55	36.62,0.58
Macaca mulatta	CHR_16	73.88,0.55	35.19,0.54	38.69,0.55	3.04,0.52	32.38,0.55
Macaca mulatta	CHR_17	92.05,0.62	26.27,0.61	65.77,0.63	1.18,0.59	25.19,0.61
Macaca mulatta	CHR_18	71.13,0.61	23.05,0.6	48.07,0.61	1,0.59	22.14,0.6
Macaca mulatta	CHR_19	52.13,0.52	22.59,0.51	29.54,0.54	2.83,0.49	19.93,0.51
Macaca mulatta	CHR_20	71.74,0.56	28.64,0.54	43.1,0.57	2.11,0.51	26.71,0.54
Macaca mulatta	CHR_X	144.36,0.61	41.88,0.61	102.47,0.62	2.46,0.59	39.58,0.61
Mus musculus	CHR_01	191.07,0.59	72.4,0.59	120.88,0.6	3.63,0.52	78.86,0.59
Mus musculus	CHR_02	177.84,0.58	71.3,0.57	109.32,0.59	4.72,0.51	64.96,0.58
Mus musculus	CHR_03	156.51,0.6	50.39,0.59	109.81,0.6	2.93,0.52	63.03,0.6
Mus musculus	CHR_04	150.56,0.58	53.9,0.56	98.49,0.59	3.58,0.5	53.49,0.57
Mus musculus	CHR_05	146,0.58	59.61,0.57	88.52,0.59	3.59,0.5	64.22,0.57
Mus musculus	CHR_06	145.05,0.59	59.24,0.58	89.45,0.6	3.12,0.51	71.23,0.59
Mus musculus	CHR_07	135.02,0.57	54.76,0.56	82.82,0.58	4.46,0.49	53.95,0.56
Mus musculus	CHR_08	123.98,0.58	44.49,0.56	79.98,0.59	2.7,0.49	42.68,0.57
Mus musculus	CHR_09	119.06,0.58	49.1,0.57	70.54,0.58	3.15,0.51	55.44,0.57
Mus musculus	CHR_10	125.06,0.59	50.27,0.58	78.6,0.6	2.81,0.51	46.08,0.58
Mus musculus	CHR_11	118.59,0.57	53.76,0.55	68.5,0.58	4.33,0.48	79.75,0.57
Mus musculus	CHR_12	112.29,0.59	37.95,0.58	74.75,0.59	1.96,0.51	33.98,0.58
Mus musculus	CHR_13	112.09,0.59	40.03,0.58	72.32,0.59	2.05,0.53	46.15,0.59
Mus musculus	CHR_14	114.98,0.59	40.81,0.58	76.65,0.6	2.18,0.52	37.73,0.58
Mus musculus	CHR_15	100.92,0.59	37.22,0.57	63.98,0.59	2.26,0.49	34.03,0.57
Mus musculus	CHR_16	93.7,0.6	62.6,0.58	121.4,0.6	3.52,0.52	58.19,0.58
Mus musculus	CHR_17	88.1,0.58	36.43,0.56	56.79,0.59	2.88,0.49	34.59,0.56
Mus musculus	CHR_18	88.47,0.59	30.59,0.58	57.99,0.6	1.52,0.51	27.3,0.58
Mus musculus	CHR_19	57.86,0.58	24.9,0.56	33.73,0.59	1.76,0.49	22.89,0.57
Mus musculus	CHR_X	157.66,0.61	45.82,0.61	118.16,0.61	2.63,0.54	42.25,0.61
Mus musculus	CHR_Y	26.77,0.62	3.62,0.61	23.59,0.62	0.36,0.57	2.7,0.62
Pan troglodytes	CHR_01	196.15,0.59	83.71,0.58	112.44,0.6	3.05,0.48	73.9,0.59
Pan troglodytes	CHR_02A	104.15,0.6	36.64,0.58	67.52,0.6	1.24,0.49	32.58,0.59
Pan troglodytes	CHR_02B	124.68,0.61	44.44,0.6	80.23,0.61	1.44,0.5	40.15,0.61
Pan troglodytes	CHR_03	188.48,0.61	71.5,0.6	116.97,0.62	1.99,0.49	64.28,0.6
Pan troglodytes	CHR_04	180.49,0.62	53.87,0.62	126.62,0.63	1.44,0.52	48.89,0.62

next

Category	SN	L(mb),p				
		Chromosome	Gene	Intergenic	Exon	Intron
Pan troglodytes	CHR_05	170.48,0.61	56.06,0.6	114.42,0.62	1.64,0.49	50.05,0.6
Pan troglodytes	CHR_06	164.15,0.61	56.2,0.6	107.94,0.62	1.85,0.49	50.14,0.6
Pan troglodytes	CHR_07	147.78,0.6	55.63,0.59	92.14,0.6	1.76,0.47	50.49,0.6
Pan troglodytes	CHR_08	138.15,0.61	43.44,0.6	94.71,0.61	1.27,0.48	39.14,0.6
Pan troglodytes	CHR_09	103.77,0.59	37.24,0.58	66.52,0.6	1.35,0.46	33.22,0.58
Pan troglodytes	CHR_10	125.21,0.59	48.37,0.59	76.84,0.59	1.43,0.48	40.94,0.59
Pan troglodytes	CHR_11	125.52,0.59	50.15,0.58	75.36,0.6	2.09,0.46	43.7,0.58
Pan troglodytes	CHR_12	100.99,0.59	40.1,0.58	60.88,0.6	1.6,0.47	37.01,0.59
Pan troglodytes	CHR_13	92.86,0.62	24.86,0.61	68,0.63	0.72,0.5	22.23,0.61
Pan troglodytes	CHR_14	82.92,0.6	31.92,0.59	50.99,0.6	1.08,0.47	28.65,0.59
Pan troglodytes	CHR_15	75.94,0.58	32.83,0.58	43.1,0.59	1.23,0.48	30.03,0.59
Pan troglodytes	CHR_16	68.51,0.56	25.6,0.54	42.91,0.57	1.36,0.43	22.12,0.55
Pan troglodytes	CHR_17	72.91,0.55	35.54,0.54	37.37,0.56	2.03,0.44	30.86,0.55
Pan troglodytes	CHR_18	72.59,0.61	25.64,0.6	46.94,0.61	0.67,0.5	22.88,0.6
Pan troglodytes	CHR_19	50.29,0.52	26.47,0.51	23.81,0.54	2.12,0.42	22.22,0.52
Pan troglodytes	CHR_20	57.35,0.57	21.99,0.56	35.36,0.57	0.92,0.44	18.74,0.56
Pan troglodytes	CHR_21	32.16,0.6	10.33,0.58	21.82,0.6	0.37,0.46	8.78,0.58
Pan troglodytes	CHR_22	30.42,0.53	15.53,0.52	14.89,0.53	0.8,0.42	13.14,0.53
Pan troglodytes	CHR_X	119.92,0.61	32.3,0.6	87.61,0.62	1.13,0.5	28.1,0.61
Pan troglodytes	CHR_Y	8.32,0.61	1.16,0.6	7.15,0.61	0.05,0.51	0.94,0.61
Rattus norvegicus	CHR_01	245.87,0.58	101.09,0.57	144.77,0.59	5.67,0.49	88.01,0.57
Rattus norvegicus	CHR_02	239.19,0.6	77.31,0.59	161.87,0.6	3,0.52	68.37,0.59
Rattus norvegicus	CHR_03	158.69,0.58	61.02,0.57	97.66,0.59	3.41,0.5	54.18,0.57
Rattus norvegicus	CHR_04	174.29,0.59	67.75,0.58	106.54,0.6	2.63,0.5	61.52,0.58
Rattus norvegicus	CHR_05	159.36,0.58	55.36,0.57	104,0.59	2.71,0.49	49.73,0.57
Rattus norvegicus	CHR_06	136.09,0.59	50.2,0.58	85.88,0.59	2.01,0.5	45.15,0.58
Rattus norvegicus	CHR_07	132.63,0.58	49.19,0.57	83.44,0.59	2.72,0.48	43.76,0.57
Rattus norvegicus	CHR_08	118.81,0.58	47.51,0.56	71.29,0.58	2.49,0.5	42.69,0.57
Rattus norvegicus	CHR_09	105.69,0.59	39.01,0.58	66.67,0.59	1.69,0.5	35.38,0.58
Rattus norvegicus	CHR_10	101.74,0.56	48.4,0.55	53.33,0.56	3.35,0.47	43.58,0.55
Rattus norvegicus	CHR_11	82.91,0.6	30.06,0.59	52.85,0.6	1.19,0.51	25.8,0.59
Rattus norvegicus	CHR_12	41.87,0.53	20.93,0.53	20.93,0.54	1.31,0.46	17.93,0.53
Rattus norvegicus	CHR_13	102.8,0.59	35.94,0.58	66.85,0.6	1.46,0.5	32.94,0.58
Rattus norvegicus	CHR_14	102.32,0.59	33.24,0.58	69.08,0.6	1.49,0.51	28.85,0.58
Rattus norvegicus	CHR_15	100.27,0.59	32.5,0.58	67.76,0.6	1.42,0.5	28.91,0.58
Rattus norvegicus	CHR_16	82.52,0.59	29.8,0.57	52.72,0.6	1.33,0.49	25.04,0.57
Rattus norvegicus	CHR_17	88.17,0.58	32.38,0.58	55.79,0.58	1.27,0.51	29.86,0.58
Rattus norvegicus	CHR_18	80.13,0.59	28.89,0.58	51.23,0.59	1.18,0.5	25.41,0.58
Rattus norvegicus	CHR_19	54.21,0.56	22.02,0.55	32.19,0.57	1.21,0.47	19.8,0.55
Rattus norvegicus	CHR_20	49.8,0.57	17.8,0.54	32,0.58	1.19,0.47	15.56,0.55
Rattus norvegicus	CHR_X	146.99,0.61	40.44,0.61	106.54,0.61	1.64,0.53	33.64,0.61
