# Supplementary material for: Universal Global Imprints of Genome Growth and Evolution – Equivalent Length and Cumulative Mutation Density
Source: PLoS One. 2010 Apr 14;5(4):e9844. doi: 10.1371/journal.pone.0009844 (PMC2854691; doi:10.1371/journal.pone.0009844)
Supplement: Table S2 — Equivalent lengths of complete sequences (100 pp). (0.36 MB PDF) [file pone.0009844.s005.pdf]

Table S2. Equivalent lengths of complete sequences

The table is composed of five lists: List 1, 467 prokaryotes including 32 archaea and 435 bacteria; List 2, 106 unicells; List 3, 39 insects; List 4, 17 plants; List 5, 236 vertebrates. Columns in each table are: name of organism, NCBI accession number, and equivalent lengths (L_e) (in kb) for k=2 to 10. For the prokaryotes (in List 1) the L_e's of the chromosome, and gene and intergenic concatenates are listed separately. For the eukaryotes (Lists 2-5) the L_e's of the chromosome, gene and intergenic concatenates, and exon and intron concatenates are listed separately.

Effective Length List 1: Pokaryotes (467).

Category	SN \ k	L_e (kb) 2	3	4	5	6	7	8	9	10
Archean (32)										
Aeropyrum pernix	NC_000854	0.247	0.567	1.30	3.43	9.50	26.8	76.5	208	—
Aeropyrum pernix	NC_000854 (Gene)	0.234	0.529	1.20	3.15	8.67	24.3	68.9	186	—
Aeropyrum pernix	NC_000854 (Intergenic)	0.335	0.801	1.95	5.12	13.7	34.3	—	—	—
Archaeoglobus fulgidus	NC_000917	0.232	0.569	1.38	3.67	10.3	29.6	86.5	243	—
Archaeoglobus fulgidus	NC_000917 (Gene)	0.234	0.564	1.35	3.57	9.95	28.5	82.8	232	—
Archaeoglobus fulgidus	NC_000917 (Intergenic)	0.162	0.412	1.08	2.94	7.81	18.9	—	—	—
Haloarcula marismortui ATCC 43049	NC_006396	0.177	0.428	1.00	2.55	6.88	18.5	51.4	145	—
Haloarcula marismortui ATCC 43049	NC_006396 (Gene)	0.152	0.371	0.860	2.17	5.83	15.6	43.0	121	—
Haloarcula marismortui ATCC 43049	NC_006396 (Intergenic)	0.640	1.17	2.75	7.19	19.7	53.0	128	—	—
Haloarcula marismortui ATCC 43049	NC_006397	0.347	0.792	1.94	5.16	14.2	37.4	88.2	—	—
Haloarcula marismortui ATCC 43049	NC_006397 (Gene)	0.253	0.615	1.52	4.04	11.1	29.0	—	—	—
Haloarcula marismortui ATCC 43049	NC_006397 (Intergenic)	1.77	1.69	3.81	9.26	20.5	—	—	—	—
Halobacterium sp	NC_002607	0.115	0.314	0.693	1.63	4.04	9.65	24.5	64.9	—
Halobacterium sp	NC_002607 (Gene)	0.104	0.284	0.621	1.45	3.58	8.51	21.5	56.7	—
Halobacterium sp	NC_002607 (Intergenic)	0.283	0.714	1.68	4.14	10.4	25.4	—	—	—
Methanobacterium thermoautotrophicum	NC_000916	0.216	0.467	1.14	3.12	8.87	25.7	74.5	206	—
Methanobacterium thermoautotrophicum	NC_000916 (Gene)	0.209	0.448	1.08	2.93	8.28	23.9	69.2	193	—
Methanobacterium thermoautotrophicum	NC_000916 (Intergenic)	0.292	0.593	1.45	3.75	9.13	18.6	—	—	—
Methanococcoides burtonii DSM 6242	NC_007955	0.395	0.922	2.26	6.18	17.8	51.8	150	406	—
Methanococcoides burtonii DSM 6242	NC_007955 (Gene)	0.357	0.832	2.02	5.51	15.8	45.9	134	361	—
Methanococcoides burtonii DSM 6242	NC_007955 (Intergenic)	0.769	1.66	4.05	10.2	25.5	57.3	110	—	—
Methanococcus jannaschii	NC_000909	0.255	0.625	1.42	3.42	8.58	22.0	57.1	144	—
Methanococcus jannaschii	NC_000909 (Gene)	0.237	0.587	1.33	3.18	7.99	20.6	53.8	138	—
Methanococcus jannaschii	NC_000909 (Intergenic)	0.389	0.792	1.72	3.70	7.52	14.1	—	—	—
Methanococcus jannaschii	NC_001732	0.279	0.692	1.59	3.69	8.21	—	—	—	—
Methanococcus jannaschii	NC_001732 (Gene)	0.196	0.500	1.18	2.78	6.30	—	—	—	—
Methanococcus jannaschii	NC_001732 (Intergenic)	0.392	0.811	1.60	3.22	—	—	—	—	—
Methanococcus jannaschii	NC_001733	0.132	0.363	0.875	2.00	4.18	—	—	—	—
Methanococcus jannaschii	NC_001733 (Gene)	0.111	0.300	0.721	1.66	—	—	—	—	—
Methanococcus jannaschii	NC_001733 (Intergenic)	0.297	0.723	1.27	—	—	—	—	—	—
Methanococcus maripaludis S2	NC_005791	0.178	0.415	1.02	2.64	7.01	18.8	50.9	135	—

next

Category	SN \ k	L_e (kb) 2	3	4	5	6	7	8	9	10
Methanococcus maripaludis S2	NC_005791 (Gene)	0.165	0.391	0.972	2.54	6.76	18.3	49.9	133	—
Methanococcus maripaludis S2	NC_005791 (Intergenic)	0.300	0.487	0.923	1.93	4.13	8.57	—	—	—
Methanopyrus kandleri	NC_003551	0.242	0.630	1.45	3.83	10.7	29.4	82.5	224	—
Methanopyrus kandleri	NC_003551 (Gene)	0.242	0.625	1.39	3.62	10.0	27.2	75.9	205	—
Methanopyrus kandleri	NC_003551 (Intergenic)	0.216	0.566	1.56	4.35	12.0	30.9	—	—	—
Methanosaeta thermophila PT	NC_008553	0.232	0.507	1.27	3.61	10.6	31.1	91.6	251	—
Methanosaeta thermophila PT	NC_008553 (Gene)	0.214	0.462	1.14	3.21	9.30	27.2	80.4	224	—
Methanosaeta thermophila PT	NC_008553 (Intergenic)	0.314	0.756	2.01	5.55	14.6	32.4	56.4	—	—
Methanosarcina acetivorans	NC_003552	0.273	0.583	1.51	4.24	12.6	38.2	117	350	939
Methanosarcina acetivorans	NC_003552 (Gene)	0.278	0.583	1.50	4.19	12.3	37.4	114	338	879
Methanosarcina acetivorans	NC_003552 (Intergenic)	0.239	0.520	1.31	3.60	10.3	29.5	81.9	207	—
Methanosarcina barkeri fusaro	NC_007355	0.303	0.676	1.76	4.98	14.7	44.2	134	390	1003
Methanosarcina barkeri fusaro	NC_007355 (Gene)	0.289	0.630	1.63	4.55	13.4	40.2	122	355	—
Methanosarcina barkeri fusaro	NC_007355 (Intergenic)	0.308	0.699	1.81	5.00	14.1	39.3	104	245	—
Methanosarcina mazei	NC_003901	0.253	0.527	1.34	3.73	10.9	32.7	99.3	294	—
Methanosarcina mazei	NC_003901 (Gene)	0.246	0.512	1.30	3.60	10.5	31.6	96.2	284	—
Methanosarcina mazei	NC_003901 (Intergenic)	0.255	0.517	1.27	3.39	9.35	25.6	68.5	—	—
Methanosphaera stadtmanae	NC_007681	0.914	1.88	2.87	6.11	13.8	31.4	75.6	179	—
Methanosphaera stadtmanae	NC_007681 (Gene)	0.732	1.64	2.62	5.72	13.1	29.9	72.8	176	—
Methanosphaera stadtmanae	NC_007681 (Intergenic)	0.838	0.856	1.14	1.97	3.77	7.60	—	—	—
Methanospirillum hungatei JF-1	NC_007796	0.231	0.489	1.25	3.50	10.3	31.3	95.8	284	—
Methanospirillum hungatei JF-1	NC_007796 (Gene)	0.222	0.467	1.20	3.34	9.83	29.8	91.2	271	—
Methanospirillum hungatei JF-1	NC_007796 (Intergenic)	0.327	0.644	1.55	4.03	10.9	28.6	64.9	—	—
Nanoarchaeum equitans	NC_005213	0.458	0.630	1.29	3.07	7.64	19.3	47.9	—	—
Nanoarchaeum equitans	NC_005213 (Gene)	0.448	0.612	1.26	2.98	7.42	18.7	46.7	—	—
Nanoarchaeum equitans	NC_005213 (Intergenic)	0.668	0.921	1.66	3.11	5.37	—	—	—	—
Natronomonas pharaonis	NC_007426	0.129	0.297	0.678	1.72	4.57	12.0	32.7	90.3	—
Natronomonas pharaonis	NC_007426 (Gene)	0.116	0.267	0.609	1.54	4.06	10.6	28.7	79.2	—
Natronomonas pharaonis	NC_007426 (Intergenic)	0.499	0.947	2.07	5.31	13.9	35.4	—	—	—
Picrophilus torridus DSM 9790	NC_005877	0.385	0.720	1.65	4.03	10.3	26.8	70.6	180	—
Picrophilus torridus DSM 9790	NC_005877 (Gene)	0.358	0.670	1.53	3.75	9.54	25.0	66.3	171	—
Picrophilus torridus DSM 9790	NC_005877 (Intergenic)	1.17	1.89	3.54	7.04	12.6	20.8	—	—	—
Pyrobaculum aerophilum	NC_003364	0.988	1.34	2.54	5.51	13.9	38.0	107	292	—
Pyrobaculum aerophilum	NC_003364 (Gene)	0.981	1.27	2.42	5.24	13.2	35.9	101	273	—

next

Category	SN \ k	L_e (kb) 2	3	4	5	6	7	8	9	10
Pyrobaculum aerophilum	NC_003364 (Intergenic)	0.800	1.49	2.90	6.33	14.8	34.6	—	—	—
Pyrococcus abyssi	NC_000868	0.266	0.620	1.57	4.28	12.2	35.4	103	281	—
Pyrococcus abyssi	NC_000868 (Gene)	0.264	0.605	1.52	4.13	11.7	33.8	97.8	266	—
Pyrococcus abyssi	NC_000868 (Intergenic)	0.207	0.529	1.43	3.84	9.78	22.7	—	—	—
Pyrococcus furiosus	NC_003413	0.212	0.548	1.44	4.01	11.5	33.8	98.6	269	—
Pyrococcus furiosus	NC_003413 (Gene)	0.210	0.538	1.41	3.91	11.2	32.9	96.2	266	—
Pyrococcus furiosus	NC_003413 (Intergenic)	0.193	0.485	1.26	3.33	7.94	16.3	—	—	—
Pyrococcus horikoshii	NC_000961	0.237	0.574	1.49	4.11	11.8	34.6	101	277	—
Pyrococcus horikoshii	NC_000961 (Gene)	0.234	0.563	1.45	3.99	11.4	33.5	97.8	269	—
Pyrococcus horikoshii	NC_000961 (Intergenic)	0.211	0.545	1.48	3.90	9.45	20.3	—	—	—
Sulfolobus acidocaldarius DSM 639	NC_007181	0.655	1.61	3.96	10.4	28.5	78.5	213	522	—
Sulfolobus acidocaldarius DSM 639	NC_007181 (Gene)	0.590	1.46	3.56	9.32	25.6	70.8	195	486	—
Sulfolobus acidocaldarius DSM 639	NC_007181 (Intergenic)	0.850	1.82	4.51	11.0	24.2	47.4	77.4	—	—
Sulfolobus solfataricus	NC_002754	0.681	1.56	3.73	9.43	25.4	69.0	185	452	—
Sulfolobus solfataricus	NC_002754 (Gene)	0.641	1.45	3.41	8.61	23.2	63.4	173	433	—
Sulfolobus solfataricus	NC_002754 (Intergenic)	0.753	1.80	4.43	10.5	23.0	44.6	73.1	—	—
Sulfolobus tokodaii	NC_003106	0.603	1.42	3.29	8.03	20.5	53.6	141	349	—
Sulfolobus tokodaii	NC_003106 (Gene)	0.567	1.33	3.02	7.34	18.8	49.2	131	335	—
Sulfolobus tokodaii	NC_003106 (Intergenic)	0.695	1.65	4.01	9.45	20.2	39.7	66.8	—	—
Thermococcus kodakaraensis KOD1	NC_006624	0.309	0.683	1.60	4.17	11.5	32.5	93.1	256	—
Thermococcus kodakaraensis KOD1	NC_006624 (Gene)	0.312	0.670	1.53	3.95	10.8	30.2	85.7	235	—
Thermococcus kodakaraensis KOD1	NC_006624 (Intergenic)	0.156	0.381	1.00	2.73	7.23	18.2	—	—	—
Thermofilum pendens Hrk 5	NC_008698	0.362	0.763	1.64	4.15	11.1	30.4	85.8	232	—
Thermofilum pendens Hrk 5	NC_008698 (Gene)	0.345	0.728	1.53	3.86	10.3	28.0	79.0	214	—
Thermofilum pendens Hrk 5	NC_008698 (Intergenic)	0.327	0.668	1.61	4.14	10.1	22.8	—	—	—
Thermoplasma acidophilum	NC_002578	0.379	0.704	1.79	5.01	14.6	43.4	128	342	—
Thermoplasma acidophilum	NC_002578 (Gene)	0.376	0.678	1.70	4.72	13.7	40.2	117	312	—
Thermoplasma acidophilum	NC_002578 (Intergenic)	0.381	0.829	2.17	5.91	16.0	39.2	—	—	—
Thermoplasma volcanium	NC_002689	0.905	1.37	3.36	9.03	25.7	73.9	206	500	—
Thermoplasma volcanium	NC_002689 (Gene)	0.807	1.23	3.01	8.13	23.1	66.4	185	447	—
Thermoplasma volcanium	NC_002689 (Intergenic)	1.59	2.51	5.77	13.8	32.6	68.7	—	—	—
Bacterials(435)										
Acidobacteria bacterium Ellin345	NC_008009	0.217	0.555	1.48	4.10	12.1	35.9	108	326	903
Acidobacteria bacterium Ellin345	NC_008009 (Gene)	0.197	0.501	1.32	3.63	10.7	31.5	94.7	285	793

next

Category	SN \ k	L_e (kb) 2	3	4	5	6	7	8	9	10
Acidobacteria bacterium Ellin345	NC_008009 (Intergenic)	0.404	1.04	2.84	8.06	23.0	61.3	137	—	—
Acidothermus cellulolyticus 11B	NC_008578	0.195	0.436	0.986	2.45	6.69	18.2	50.8	143	—
Acidothermus cellulolyticus 11B	NC_008578 (Gene)	0.186	0.420	0.947	2.34	6.36	17.2	47.9	134	—
Acidothermus cellulolyticus 11B	NC_008578 (Intergenic)	0.327	0.639	1.41	3.51	9.23	23.6	—	—	—
Acinetobacter sp ADP1	NC_005966	0.270	0.660	1.80	5.02	14.5	42.9	128	368	—
Acinetobacter sp ADP1	NC_005966 (Gene)	0.255	0.625	1.72	4.79	13.8	40.9	122	350	—
Acinetobacter sp ADP1	NC_005966 (Intergenic)	0.337	0.659	1.46	3.55	9.07	23.2	57.4	—	—
Aeromonas hydrophila ATCC 7966	NC_008570	0.365	0.668	1.29	3.09	7.96	20.1	54.1	148	380
Aeromonas hydrophila ATCC 7966	NC_008570 (Gene)	0.351	0.612	1.13	2.68	6.85	17.0	45.5	124	316
Aeromonas hydrophila ATCC 7966	NC_008570 (Intergenic)	0.355	0.883	2.17	5.67	15.5	42.3	107	—	—
Agrobacterium tumefaciens C58 Cereon	NC_003062	0.188	0.407	1.00	2.62	7.28	20.6	59.4	171	—
Agrobacterium tumefaciens C58 Cereon	NC_003062 (Gene)	0.174	0.372	0.912	2.36	6.53	18.3	52.6	150	—
Agrobacterium tumefaciens C58 Cereon	NC_003062 (Intergenic)	0.285	0.691	1.78	4.86	13.6	36.6	85.8	—	—
Agrobacterium tumefaciens C58 Cereon	NC_003063	0.198	0.432	1.07	2.79	7.80	22.2	64.1	182	—
Agrobacterium tumefaciens C58 Cereon	NC_003063 (Gene)	0.187	0.404	0.994	2.58	7.18	20.3	58.2	164	—
Agrobacterium tumefaciens C58 Cereon	NC_003063 (Intergenic)	0.321	0.769	2.00	5.43	15.1	39.0	—	—	—
Agrobacterium tumefaciens C58 UWash	NC_003304	0.188	0.407	1.00	2.62	7.28	20.6	59.4	171	—
Agrobacterium tumefaciens C58 UWash	NC_003304 (Gene)	0.173	0.370	0.907	2.35	6.50	18.2	52.2	149	—
Agrobacterium tumefaciens C58 UWash	NC_003304 (Intergenic)	0.275	0.662	1.70	4.63	13.0	35.6	86.4	—	—
Agrobacterium tumefaciens C58 UWash	NC_003305	0.198	0.432	1.07	2.79	7.81	22.2	64.1	182	—
Agrobacterium tumefaciens C58 UWash	NC_003305 (Gene)	0.185	0.400	0.985	2.56	7.12	20.1	57.7	163	—
Agrobacterium tumefaciens C58 UWash	NC_003305 (Intergenic)	0.324	0.769	1.98	5.37	14.9	39.3	—	—	—
Alcanivorax borkumensis SK2	NC_008260	0.436	0.901	2.26	6.02	17.2	50.5	150	425	—
Alcanivorax borkumensis SK2	NC_008260 (Gene)	0.415	0.821	2.04	5.42	15.4	44.9	132	373	—
Alcanivorax borkumensis SK2	NC_008260 (Intergenic)	0.516	1.29	3.13	8.37	23.1	61.3	138	—	—
Alkalilimnicola ehrlichei MLHE-1	NC_008340	1.12	1.13	1.84	4.11	10.1	23.9	61.0	157	—
Alkalilimnicola ehrlichei MLHE-1	NC_008340 (Gene)	1.03	1.00	1.60	3.58	8.76	20.7	53.3	140	—
Alkalilimnicola ehrlichei MLHE-1	NC_008340 (Intergenic)	1.75	3.53	4.95	8.82	14.4	20.9	26.7	—	—
Anabaena variabilis ATCC 29413	NC_007413	0.564	1.23	2.94	7.50	19.7	52.8	141	380	947
Anabaena variabilis ATCC 29413	NC_007413 (Gene)	0.536	1.14	2.69	6.84	17.9	47.6	127	350	913
Anabaena variabilis ATCC 29413	NC_007413 (Intergenic)	0.596	1.28	2.97	7.17	17.4	40.5	82.8	144	—
Anaeromyxobacter dehalogenans 2CP-C	NC_007760	0.126	0.260	0.521	1.20	2.96	6.79	16.7	42.7	102
Anaeromyxobacter dehalogenans 2CP-C	NC_007760 (Gene)	0.119	0.245	0.485	1.12	2.74	6.27	15.4	39.3	93.2
Anaeromyxobacter dehalogenans 2CP-C	NC_007760 (Intergenic)	0.251	0.490	1.08	2.37	5.33	12.0	27.0	—	—

next

Category	SN \ k	L_e (kb) 2	3	4	5	6	7	8	9	10
Anaplasma marginale St Maries	NC_004842	0.579	1.35	3.60	10.1	29.6	86.8	235	512	—
Anaplasma marginale St Maries	NC_004842 (Gene)	0.597	1.38	3.62	10.1	29.5	85.2	226	—	—
Anaplasma marginale St Maries	NC_004842 (Intergenic)	0.335	0.854	2.34	6.59	18.2	44.3	—	—	—
Anaplasma phagocytophilum HZ	NC_007797	0.967	1.79	4.29	11.4	32.1	88.6	224	454	—
Anaplasma phagocytophilum HZ	NC_007797 (Gene)	0.886	1.72	4.04	10.7	29.8	79.8	193	365	—
Anaplasma phagocytophilum HZ	NC_007797 (Intergenic)	0.931	1.65	4.00	10.5	27.8	68.4	137	—	—
Aquifex aeolicus	NC_000918	0.149	0.322	0.822	2.24	6.40	18.5	54.0	153	—
Aquifex aeolicus	NC_000918 (Gene)	0.149	0.320	0.817	2.23	6.34	18.3	53.4	150	—
Aquifex aeolicus	NC_000918 (Intergenic)	0.146	0.333	0.856	2.26	5.97	14.7	—	—	—
Arthrobacter FB24	NC_008541	0.970	0.981	1.81	4.29	11.2	29.1	79.4	219	567
Arthrobacter FB24	NC_008541 (Gene)	0.895	0.905	1.64	3.86	10.1	25.9	70.2	194	500
Arthrobacter FB24	NC_008541 (Intergenic)	1.29	1.66	3.56	8.86	23.1	58.3	129	—	—
Aster yellows witches-broom phytoplasma AYWB	NC_007716	0.095	0.212	0.507	1.27	3.29	8.59	21.9	—	—
Aster yellows witches-broom phytoplasma AYWB	NC_007716 (Gene)	0.096	0.221	0.548	1.41	3.71	9.78	25.2	—	—
Aster yellows witches-broom phytoplasma AYWB	NC_007716 (Intergenic)	0.089	0.180	0.385	0.875	2.05	4.91	—	—	—
Azoarcus sp EbN1	NC_006513	0.116	0.298	0.753	1.95	5.40	14.7	40.7	116	308
Azoarcus sp EbN1	NC_006513 (Gene)	0.107	0.274	0.689	1.78	4.91	13.3	36.6	104	—
Azoarcus sp EbN1	NC_006513 (Intergenic)	0.292	0.728	1.92	5.18	14.3	38.3	91.1	—	—
Bacillus anthracis Ames	NC_003997	0.616	1.21	2.79	6.87	17.9	48.2	133	366	933
Bacillus anthracis Ames	NC_003997 (Gene)	0.570	1.17	2.70	6.60	17.0	45.5	125	341	855
Bacillus anthracis Ames	NC_003997 (Intergenic)	0.616	0.753	1.55	3.72	9.45	24.1	60.5	—	—
Bacillus anthracis Ames 0581	NC_007530	0.615	1.21	2.79	6.87	17.9	48.2	133	366	933
Bacillus anthracis Ames 0581	NC_007530 (Gene)	0.570	1.17	2.70	6.60	17.0	45.5	125	341	856
Bacillus anthracis Ames 0581	NC_007530 (Intergenic)	0.615	0.748	1.54	3.69	9.37	23.9	59.9	—	—
Bacillus anthracis str Sterne	NC_005945	0.615	1.21	2.79	6.87	17.9	48.2	133	366	933
Bacillus anthracis str Sterne	NC_005945 (Gene)	0.571	1.17	2.70	6.61	17.1	45.6	126	342	856
Bacillus anthracis str Sterne	NC_005945 (Intergenic)	0.612	0.769	1.59	3.83	9.71	24.9	62.5	—	—
Bacillus cereus ATCC14579	NC_004722	0.617	1.24	2.87	7.11	18.6	50.1	138	378	952
Bacillus cereus ATCC14579	NC_004722 (Gene)	0.574	1.19	2.77	6.80	17.6	47.0	129	350	870
Bacillus cereus ATCC14579	NC_004722 (Intergenic)	0.610	0.832	1.75	4.23	10.8	27.4	68.2	—	—
Bacillus cereus ATCC 10987	NC_003909	0.612	1.21	2.78	6.87	18.0	48.5	134	367	926
Bacillus cereus ATCC 10987	NC_003909 (Gene)	0.562	1.15	2.67	6.57	17.1	45.7	126	343	857
Bacillus cereus ATCC 10987	NC_003909 (Intergenic)	0.655	0.807	1.65	3.94	9.96	25.2	61.5	—	—
Bacillus cereus ZK	NC_006274	0.619	1.22	2.80	6.90	18.0	48.4	134	367	929

next

			L_e (kb)								
Category	SN	\ k	2	3	4	5	6	7	8	9	10
Bacillus cereus ZK	NC_006274 (Gene)		0.575	1.17	2.71	6.63	17.1	45.7	126	341	849
Bacillus cereus ZK	NC_006274 (Intergenic)		0.612	0.780	1.62	3.88	9.85	25.1	63.0	—	—
Bacillus clausii KSM-K16	NC_006582		0.234	0.575	1.51	4.21	12.4	37.7	116	348	942
Bacillus clausii KSM-K16	NC_006582 (Gene)		0.217	0.540	1.43	3.99	11.8	35.6	109	326	—
Bacillus clausii KSM-K16	NC_006582 (Intergenic)		0.305	0.621	1.47	3.76	10.3	28.6	75.0	—	—
Bacillus halodurans	NC_002570		0.328	0.789	2.05	5.71	16.9	50.9	154	447	1100
Bacillus halodurans	NC_002570 (Gene)		0.315	0.767	2.01	5.62	16.5	49.6	150	429	—
Bacillus halodurans	NC_002570 (Intergenic)		0.316	0.631	1.48	3.86	10.7	29.6	76.1	—	—
Bacillus licheniformis ATCC 14580	NC_006270		0.182	0.399	1.05	2.98	8.83	27.0	83.6	255	720
Bacillus licheniformis ATCC 14580	NC_006270 (Gene)		0.173	0.385	1.02	2.89	8.55	26.1	80.4	244	—
Bacillus licheniformis ATCC 14580	NC_006270 (Intergenic)		0.203	0.391	0.934	2.48	6.95	19.8	54.0	—	—
Bacillus licheniformis DSM 13	NC_006322		0.182	0.399	1.05	2.98	8.83	27.0	83.5	255	719
Bacillus licheniformis DSM 13	NC_006322 (Gene)		0.173	0.384	1.02	2.88	8.54	26.0	80.2	243	—
Bacillus licheniformis DSM 13	NC_006322 (Intergenic)		0.204	0.393	0.938	2.50	6.99	19.9	54.2	—	—
Bacillus subtilis	NC_000964		0.232	0.518	1.37	3.85	11.4	34.5	106	318	866
Bacillus subtilis	NC_000964 (Gene)		0.221	0.502	1.33	3.77	11.2	33.8	103	309	—
Bacillus subtilis	NC_000964 (Intergenic)		0.262	0.478	1.11	2.88	7.87	21.8	57.9	—	—
Bacillus thuringiensis Al Hakam	NC_008600		0.632	1.23	2.83	6.96	18.1	48.8	135	368	926
Bacillus thuringiensis Al Hakam	NC_008600 (Gene)		0.581	1.19	2.73	6.68	17.3	46.2	127	343	846
Bacillus thuringiensis Al Hakam	NC_008600 (Intergenic)		0.705	0.885	1.81	4.31	10.9	27.8	69.5	—	—
Bacillus thuringiensis konkukian	NC_005957		0.627	1.22	2.80	6.90	18.0	48.4	134	365	917
Bacillus thuringiensis konkukian	NC_005957 (Gene)		0.580	1.18	2.71	6.63	17.1	45.7	126	340	842
Bacillus thuringiensis konkukian	NC_005957 (Intergenic)		0.634	0.781	1.61	3.85	9.74	24.9	62.3	—	—
Bacteroides fragilis NCTC 9434	NC_003228		0.788	1.20	2.75	7.27	20.8	61.4	184	535	1376
Bacteroides fragilis NCTC 9434	NC_003228 (Gene)		0.770	1.15	2.65	7.02	20.0	59.3	177	513	1306
Bacteroides fragilis NCTC 9434	NC_003228 (Intergenic)		0.498	0.897	2.03	4.99	12.9	32.6	76.7	—	—
Bacteroides fragilis YCH46	NC_006347		0.784	1.20	2.76	7.32	20.9	61.9	186	540	1392
Bacteroides fragilis YCH46	NC_006347 (Gene)		0.763	1.16	2.66	7.05	20.1	59.6	178	517	1318
Bacteroides fragilis YCH46	NC_006347 (Intergenic)		0.498	0.922	2.09	5.15	13.3	33.4	77.4	—	—
Bacteroides thetaiotaomicron VPI-5482	NC_004663		0.810	1.16	2.63	6.92	19.6	57.7	173	511	1366
Bacteroides thetaiotaomicron VPI-5482	NC_004663 (Gene)		0.765	1.08	2.46	6.47	18.3	54.0	162	477	1268
Bacteroides thetaiotaomicron VPI-5482	NC_004663 (Intergenic)		0.633	1.18	2.58	6.10	15.5	38.4	89.1	—	—
Bartonella henselae Houston-1	NC_005956		0.212	0.511	1.30	3.63	10.6	31.5	93.0	258	—
Bartonella henselae Houston-1	NC_005956 (Gene)		0.207	0.518	1.35	3.79	11.1	32.8	95.5	256	—

next

Category	SN \ k	L_e (kb) 2	3	4	5	6	7	8	9	10
Bartonella henselae Houston-1	NC_005956 (Intergenic)	0.193	0.394	0.927	2.45	6.80	18.9	50.3	—	—
Bartonella quintana Toulouse	NC_005955	0.207	0.510	1.32	3.71	10.9	32.6	96.3	266	—
Bartonella quintana Toulouse	NC_005955 (Gene)	0.200	0.508	1.34	3.77	11.1	32.9	96.0	257	—
Bartonella quintana Toulouse	NC_005955 (Intergenic)	0.200	0.421	1.02	2.74	7.69	21.4	55.8	—	—
Baumannia cicadellinicola Homalodisca coagulata	NC_007984	1.37	2.27	3.74	8.21	20.4	50.6	123	—	—
Baumannia cicadellinicola Homalodisca coagulata	NC_007984 (Gene)	1.47	2.13	3.51	7.78	19.3	47.9	116	—	—
Baumannia cicadellinicola Homalodisca coagulata	NC_007984 (Intergenic)	0.413	1.04	2.26	4.60	9.68	19.6	—	—	—
Bdellovibrio bacteriovorus	NC_005363	0.212	0.485	1.32	3.72	10.9	33.0	100	298	—
Bdellovibrio bacteriovorus	NC_005363 (Gene)	0.206	0.465	1.25	3.52	10.3	30.8	93.4	277	—
Bdellovibrio bacteriovorus	NC_005363 (Intergenic)	0.190	0.498	1.40	4.03	11.6	31.7	74.5	—	—
Bifidobacterium adolescentis ATCC 15703	NC_008618	0.205	0.481	1.22	3.27	9.21	25.5	72.1	199	—
Bifidobacterium adolescentis ATCC 15703	NC_008618 (Gene)	0.191	0.445	1.11	2.93	8.17	22.4	62.8	173	—
Bifidobacterium adolescentis ATCC 15703	NC_008618 (Intergenic)	0.313	0.735	1.85	5.02	13.6	34.1	—	—	—
Bifidobacterium longum	NC_004307	0.319	0.664	1.58	4.02	11.0	29.7	82.9	226	—
Bifidobacterium longum	NC_004307 (Gene)	0.289	0.582	1.36	3.45	9.36	25.1	69.8	190	—
Bifidobacterium longum	NC_004307 (Intergenic)	0.630	1.55	3.88	9.84	25.2	58.9	113	—	—
Bordetella bronchiseptica	NC_002927	0.172	0.312	0.654	1.55	3.91	9.64	25.1	66.9	170
Bordetella bronchiseptica	NC_002927 (Gene)	0.161	0.290	0.602	1.43	3.60	8.83	23.0	61.1	155
Bordetella bronchiseptica	NC_002927 (Intergenic)	0.445	0.785	1.62	3.62	8.28	18.6	39.3	—	—
Bordetella parapertussis	NC_002928	0.168	0.307	0.644	1.53	3.86	9.54	24.9	66.2	168
Bordetella parapertussis	NC_002928 (Gene)	0.158	0.286	0.595	1.41	3.56	8.75	22.8	60.6	153
Bordetella parapertussis	NC_002928 (Intergenic)	0.425	0.746	1.54	3.47	7.97	18.0	38.2	—	—
Bordetella pertussis	NC_002929	0.183	0.328	0.684	1.63	4.09	10.0	25.6	63.8	—
Bordetella pertussis	NC_002929 (Gene)	0.169	0.302	0.623	1.48	3.72	9.09	23.2	57.8	—
Bordetella pertussis	NC_002929 (Intergenic)	0.492	0.876	1.82	3.90	8.27	15.8	25.8	—	—
Borrelia afzelii PKo	NC_008277	0.157	0.375	0.915	2.33	6.07	15.9	41.6	—	—
Borrelia afzelii PKo	NC_008277 (Gene)	0.152	0.364	0.890	2.27	5.93	15.5	40.7	—	—
Borrelia afzelii PKo	NC_008277 (Intergenic)	0.232	0.490	1.06	2.42	5.38	—	—	—	—
Borrelia burgdorferi	NC_001318	0.147	0.352	0.867	2.22	5.83	15.3	40.3	—	—
Borrelia burgdorferi	NC_001318 (Gene)	0.143	0.344	0.849	2.18	5.72	15.0	39.6	—	—
Borrelia burgdorferi	NC_001318 (Intergenic)	0.206	0.440	0.973	2.26	5.22	—	—	—	—
Borrelia garinii PBi	NC_006156	0.158	0.376	0.917	2.33	6.06	15.9	41.5	—	—
Borrelia garinii PBi	NC_006156 (Gene)	0.153	0.367	0.898	2.29	5.96	15.6	40.8	—	—
Borrelia garinii PBi	NC_006156 (Intergenic)	0.212	0.439	0.928	2.12	4.81	—	—	—	—

next

Category	SN $\backslash$ k	L_e (kb)								
		2	3	4	5	6	7	8	9	10
Bradyrhizobium japonicum	NC_004463	0.136	0.332	0.811	2.10	5.83	15.8	44.4	128	351
Bradyrhizobium japonicum	NC_004463 (Gene)	0.122	0.295	0.706	1.81	4.99	13.4	37.2	107	292
Bradyrhizobium japonicum	NC_004463 (Intergenic)	0.287	0.763	2.10	5.85	16.5	45.1	111	230	—
Brucella abortus 9-941	NC_006932	0.181	0.409	1.07	2.85	7.90	22.7	65.9	187	—
Brucella abortus 9-941	NC_006932 (Gene)	0.166	0.371	0.962	2.55	7.02	20.0	57.7	164	—
Brucella abortus 9-941	NC_006932 (Intergenic)	0.254	0.603	1.56	4.24	11.5	29.9	—	—	—
Brucella abortus 9-941	NC_006933	0.183	0.415	1.08	2.86	7.90	22.6	64.4	176	—
Brucella abortus 9-941	NC_006933 (Gene)	0.170	0.384	0.992	2.61	7.18	20.4	57.8	—	—
Brucella abortus 9-941	NC_006933 (Intergenic)	0.275	0.649	1.69	4.54	11.9	28.4	—	—	—
Brucella melitensis	NC_003317	0.182	0.410	1.07	2.86	7.92	22.7	66.0	187	—
Brucella melitensis	NC_003317 (Gene)	0.168	0.374	0.970	2.57	7.06	20.1	57.8	164	—
Brucella melitensis	NC_003317 (Intergenic)	0.237	0.562	1.47	4.02	11.1	29.5	68.2	—	—
Brucella melitensis	NC_003318	0.183	0.415	1.08	2.86	7.90	22.6	64.6	176	—
Brucella melitensis	NC_003318 (Gene)	0.171	0.384	0.993	2.62	7.18	20.4	57.8	—	—
Brucella melitensis	NC_003318 (Intergenic)	0.264	0.632	1.65	4.46	12.0	30.0	—	—	—
Brucella melitensis biovar Abortus	NC_007618	0.181	0.409	1.07	2.85	7.90	22.7	65.8	187	—
Brucella melitensis biovar Abortus	NC_007618 (Gene)	0.166	0.371	0.962	2.55	7.02	20.0	57.6	163	—
Brucella melitensis biovar Abortus	NC_007618 (Intergenic)	0.250	0.597	1.56	4.24	11.6	30.5	69.0	—	—
Brucella melitensis biovar Abortus	NC_007624	0.182	0.415	1.08	2.86	7.89	22.5	64.4	175	—
Brucella melitensis biovar Abortus	NC_007624 (Gene)	0.170	0.384	0.991	2.61	7.17	20.3	57.6	—	—
Brucella melitensis biovar Abortus	NC_007624 (Intergenic)	0.270	0.644	1.69	4.57	12.1	29.6	—	—	—
Brucella suis 1330	NC_004310	0.180	0.406	1.06	2.83	7.83	22.5	65.2	185	—
Brucella suis 1330	NC_004310 (Gene)	0.166	0.369	0.958	2.54	6.99	19.9	57.3	163	—
Brucella suis 1330	NC_004310 (Intergenic)	0.256	0.605	1.57	4.25	11.5	29.6	—	—	—
Brucella suis 1330	NC_004311	0.184	0.418	1.09	2.89	7.99	22.8	65.4	179	—
Brucella suis 1330	NC_004311 (Gene)	0.172	0.387	1.00	2.64	7.25	20.6	58.7	160	—
Brucella suis 1330	NC_004311 (Intergenic)	0.286	0.676	1.76	4.78	12.6	30.1	—	—	—
Buchnera aphidicola	NC_004545	0.739	1.06	1.95	4.19	9.48	22.5	53.2	—	—
Buchnera aphidicola	NC_004545 (Gene)	0.646	0.952	1.78	3.88	8.86	21.3	51.1	—	—
Buchnera aphidicola	NC_004545 (Intergenic)	0.736	1.02	1.76	3.26	6.22	12.1	—	—	—
Buchnera aphidicola Cc Cinara cedri	NC_008513	0.271	0.339	0.546	0.985	1.90	3.91	8.30	—	—
Buchnera aphidicola Cc Cinara cedri	NC_008513 (Gene)	0.237	0.313	0.512	0.925	1.77	3.65	7.81	—	—
Buchnera aphidicola Cc Cinara cedri	NC_008513 (Intergenic)	0.756	0.470	0.680	1.14	2.00	—	—	—	—
Buchnera aphidicola Sg	NC_004061	0.253	0.416	0.782	1.66	3.74	8.92	21.7	—	—

next

Category	SN \ k	L_e (kb) 2	3	4	5	6	7	8	9	10
Buchnera aphidicola Sg	NC_004061 (Gene)	0.261	0.440	0.848	1.84	4.16	10.0	24.8	—	—
Buchnera aphidicola Sg	NC_004061 (Intergenic)	0.161	0.215	0.344	0.633	1.24	—	—	—	—
Buchnera sp	NC_002528	0.384	0.583	1.05	2.20	4.91	11.6	27.7	—	—
Buchnera sp	NC_002528 (Gene)	0.384	0.612	1.14	2.43	5.47	13.0	31.9	—	—
Buchnera sp	NC_002528 (Intergenic)	0.326	0.330	0.505	0.929	1.81	3.56	—	—	—
Burkholderia 383	NC_007509	0.083	0.224	0.582	1.53	4.29	11.7	32.3	90.2	—
Burkholderia 383	NC_007509 (Gene)	0.076	0.203	0.521	1.36	3.78	10.2	27.8	77.1	—
Burkholderia 383	NC_007509 (Intergenic)	0.152	0.409	1.12	3.10	8.65	23.2	—	—	—
Burkholderia 383	NC_007510	0.067	0.181	0.459	1.19	3.27	8.70	23.5	66.2	—
Burkholderia 383	NC_007510 (Gene)	0.061	0.164	0.408	1.05	2.87	7.57	20.3	57.0	—
Burkholderia 383	NC_007510 (Intergenic)	0.163	0.416	1.08	2.84	7.56	20.1	51.3	—	—
Burkholderia 383	NC_007511	0.065	0.177	0.449	1.16	3.20	8.56	23.2	65.8	—
Burkholderia 383	NC_007511 (Gene)	0.059	0.160	0.401	1.03	2.82	7.48	20.1	56.7	—
Burkholderia 383	NC_007511 (Intergenic)	0.144	0.377	1.00	2.67	7.24	19.5	50.8	—	—
Burkholderia cenocepacia AU 1054	NC_008060	0.061	0.165	0.412	1.05	2.86	7.51	20.0	55.8	—
Burkholderia cenocepacia AU 1054	NC_008060 (Gene)	0.056	0.150	0.370	0.937	2.54	6.63	17.5	48.6	—
Burkholderia cenocepacia AU 1054	NC_008060 (Intergenic)	0.141	0.365	0.944	2.46	6.43	16.7	42.1	—	—
Burkholderia cenocepacia AU 1054	NC_008061	0.061	0.166	0.419	1.07	2.94	7.80	20.9	58.5	—
Burkholderia cenocepacia AU 1054	NC_008061 (Gene)	0.055	0.150	0.375	0.953	2.60	6.82	18.2	50.6	—
Burkholderia cenocepacia AU 1054	NC_008061 (Intergenic)	0.132	0.351	0.929	2.46	6.61	17.6	44.8	—	—
Burkholderia cenocepacia AU 1054	NC_008062	0.061	0.165	0.417	1.06	2.91	7.69	20.5	56.3	—
Burkholderia cenocepacia AU 1054	NC_008062 (Gene)	0.056	0.151	0.376	0.953	2.60	6.80	18.0	49.2	—
Burkholderia cenocepacia AU 1054	NC_008062 (Intergenic)	0.134	0.351	0.921	2.43	6.38	16.3	—	—	—
Burkholderia cenocepacia HI2424	NC_008542	0.062	0.166	0.416	1.06	2.90	7.60	20.3	56.5	—
Burkholderia cenocepacia HI2424	NC_008542 (Gene)	0.056	0.150	0.370	0.937	2.55	6.63	17.6	48.7	—
Burkholderia cenocepacia HI2424	NC_008542 (Intergenic)	0.150	0.384	0.988	2.58	6.72	17.6	44.4	—	—
Burkholderia cenocepacia HI2424	NC_008543	0.061	0.165	0.416	1.07	2.92	7.74	20.8	58.1	—
Burkholderia cenocepacia HI2424	NC_008543 (Gene)	0.055	0.150	0.373	0.948	2.59	6.79	18.1	50.4	—
Burkholderia cenocepacia HI2424	NC_008543 (Intergenic)	0.133	0.351	0.930	2.46	6.59	17.6	45.1	—	—
Burkholderia cenocepacia HI2424	NC_008544	0.060	0.164	0.414	1.06	2.89	7.65	20.4	55.9	—
Burkholderia cenocepacia HI2424	NC_008544 (Gene)	0.056	0.152	0.380	0.960	2.62	6.87	18.2	—	—
Burkholderia cenocepacia HI2424	NC_008544 (Intergenic)	0.119	0.317	0.838	2.21	5.85	14.9	—	—	—
Burkholderia cepacia AMMD	NC_008390	0.061	0.166	0.414	1.06	2.89	7.59	20.2	56.4	—
Burkholderia cepacia AMMD	NC_008390 (Gene)	0.056	0.151	0.371	0.939	2.56	6.65	17.6	48.9	—

next

Category	SN \ k	L_e (kb) 2	3	4	5	6	7	8	9	10
Burkholderia cepacia AMMD	NC_008390 (Intergenic)	0.140	0.359	0.924	2.40	6.25	16.4	41.4	—	—
Burkholderia cepacia AMMD	NC_008391	0.060	0.164	0.414	1.06	2.92	7.75	20.9	58.5	—
Burkholderia cepacia AMMD	NC_008391 (Gene)	0.054	0.148	0.370	0.941	2.58	6.78	18.1	50.5	—
Burkholderia cepacia AMMD	NC_008391 (Intergenic)	0.128	0.337	0.888	2.36	6.29	16.7	42.8	—	—
Burkholderia cepacia AMMD	NC_008392	0.068	0.185	0.470	1.22	3.36	8.99	24.3	67.3	—
Burkholderia cepacia AMMD	NC_008392 (Gene)	0.063	0.170	0.427	1.10	3.01	7.99	21.4	59.0	—
Burkholderia cepacia AMMD	NC_008392 (Intergenic)	0.135	0.360	0.970	2.63	7.15	18.8	—	—	—
Burkholderia mallei ATCC 23344	NC_006348	0.046	0.125	0.312	0.767	2.04	5.19	13.3	35.9	—
Burkholderia mallei ATCC 23344	NC_006348 (Gene)	0.043	0.118	0.293	0.717	1.91	4.84	12.4	33.3	—
Burkholderia mallei ATCC 23344	NC_006348 (Intergenic)	0.072	0.179	0.433	1.03	2.46	5.90	14.0	—	—
Burkholderia mallei ATCC 23344	NC_006349	0.042	0.114	0.285	0.694	1.83	4.63	11.8	31.6	—
Burkholderia mallei ATCC 23344	NC_006349 (Gene)	0.040	0.109	0.270	0.655	1.73	4.38	11.1	29.9	—
Burkholderia mallei ATCC 23344	NC_006349 (Intergenic)	0.062	0.156	0.380	0.904	2.16	5.17	12.1	—	—
Burkholderia pseudomallei 1710b	NC_007434	0.048	0.131	0.331	0.818	2.18	5.59	14.4	39.3	—
Burkholderia pseudomallei 1710b	NC_007434 (Gene)	0.044	0.120	0.300	0.735	1.96	4.98	12.8	34.6	—
Burkholderia pseudomallei 1710b	NC_007434 (Intergenic)	0.091	0.235	0.603	1.52	3.88	9.87	24.6	—	—
Burkholderia pseudomallei 1710b	NC_007435	0.045	0.122	0.306	0.751	1.99	5.07	13.0	35.2	—
Burkholderia pseudomallei 1710b	NC_007435 (Gene)	0.042	0.114	0.285	0.695	1.84	4.67	12.0	32.3	—
Burkholderia pseudomallei 1710b	NC_007435 (Intergenic)	0.071	0.184	0.465	1.15	2.89	7.22	17.8	—	—
Burkholderia pseudomallei K96243	NC_006350	0.047	0.129	0.325	0.803	2.14	5.47	14.1	38.4	—
Burkholderia pseudomallei K96243	NC_006350 (Gene)	0.044	0.121	0.302	0.740	1.97	5.01	12.8	34.8	—
Burkholderia pseudomallei K96243	NC_006350 (Intergenic)	0.070	0.177	0.434	1.05	2.58	6.34	15.4	—	—
Burkholderia pseudomallei K96243	NC_006351	0.044	0.120	0.302	0.741	1.96	5.00	12.9	34.8	—
Burkholderia pseudomallei K96243	NC_006351 (Gene)	0.041	0.114	0.283	0.689	1.83	4.64	11.8	32.0	—
Burkholderia pseudomallei K96243	NC_006351 (Intergenic)	0.061	0.154	0.380	0.921	2.26	5.58	13.7	—	—
Burkholderia thailandensis E264	NC_007650	0.044	0.121	0.306	0.758	2.02	5.21	13.5	36.9	—
Burkholderia thailandensis E264	NC_007650 (Gene)	0.042	0.115	0.289	0.709	1.89	4.85	12.5	34.0	—
Burkholderia thailandensis E264	NC_007650 (Intergenic)	0.064	0.166	0.422	1.05	2.64	6.67	16.7	—	—
Burkholderia thailandensis E264	NC_007651	0.048	0.131	0.333	0.829	2.23	5.75	15.0	41.0	—
Burkholderia thailandensis E264	NC_007651 (Gene)	0.045	0.123	0.309	0.763	2.04	5.24	13.6	37.0	—
Burkholderia thailandensis E264	NC_007651 (Intergenic)	0.078	0.202	0.515	1.29	3.26	8.24	20.6	—	—
Burkholderia xenovorans LB400	NC_007951	0.106	0.278	0.734	1.97	5.59	15.8	45.3	132	367
Burkholderia xenovorans LB400	NC_007951 (Gene)	0.096	0.252	0.660	1.76	4.96	13.9	39.5	115	316
Burkholderia xenovorans LB400	NC_007951 (Intergenic)	0.211	0.548	1.47	4.03	11.3	32.0	86.4	—	—

next

Category	SN \ k	L_e (kb) 2	3	4	5	6	7	8	9	10
Burkholderia xenovorans LB400	NC_007952	0.101	0.269	0.717	1.93	5.48	15.5	44.6	130	—
Burkholderia xenovorans LB400	NC_007952 (Gene)	0.093	0.245	0.650	1.74	4.91	13.8	39.3	114	—
Burkholderia xenovorans LB400	NC_007952 (Intergenic)	0.206	0.536	1.45	4.02	11.4	32.1	83.6	—	—
Burkholderia xenovorans LB400	NC_007953	0.146	0.381	1.04	2.85	8.25	23.8	68.6	191	—
Burkholderia xenovorans LB400	NC_007953 (Gene)	0.133	0.347	0.938	2.56	7.37	21.1	60.3	167	—
Burkholderia xenovorans LB400	NC_007953 (Intergenic)	0.294	0.762	2.10	5.91	16.6	42.8	—	—	—
Campylobacter fetus 82-40	NC_008599	0.385	0.476	1.04	2.58	6.47	16.4	42.8	109	—
Campylobacter fetus 82-40	NC_008599 (Gene)	0.380	0.463	1.01	2.54	6.47	16.8	44.7	116	—
Campylobacter fetus 82-40	NC_008599 (Intergenic)	0.353	0.582	1.08	2.10	3.78	6.69	—	—	—
Campylobacter jejuni	NC_002163	0.129	0.250	0.603	1.53	4.02	10.8	29.4	79.0	—
Campylobacter jejuni	NC_002163 (Gene)	0.131	0.253	0.611	1.56	4.09	11.0	29.9	80.4	—
Campylobacter jejuni	NC_002163 (Intergenic)	0.093	0.182	0.402	0.911	2.09	4.70	—	—	—
Campylobacter jejuni RM1221	NC_003912	0.128	0.248	0.601	1.53	4.03	10.8	29.4	79.0	—
Campylobacter jejuni RM1221	NC_003912 (Gene)	0.129	0.250	0.606	1.55	4.08	10.9	29.7	79.9	—
Campylobacter jejuni RM1221	NC_003912 (Intergenic)	0.107	0.208	0.465	1.08	2.52	5.78	—	—	—
Candidatus Blochmannia floridanus	NC_005061	1.27	2.32	4.37	9.58	22.0	51.4	119	—	—
Candidatus Blochmannia floridanus	NC_005061 (Gene)	1.20	1.96	3.66	8.00	18.1	42.4	98.9	—	—
Candidatus Blochmannia floridanus	NC_005061 (Intergenic)	0.247	0.498	1.09	2.40	5.31	11.4	—	—	—
Candidatus Blochmannia pennsylvanicus BPEN	NC_007292	1.32	2.36	4.53	10.2	23.8	57.3	134	—	—
Candidatus Blochmannia pennsylvanicus BPEN	NC_007292 (Gene)	1.21	1.97	3.73	8.34	19.4	47.6	113	—	—
Candidatus Blochmannia pennsylvanicus BPEN	NC_007292 (Intergenic)	0.768	1.54	3.37	6.89	13.8	26.6	—	—	—
Candidatus Carsonella ruddii	NC_008512	0.119	0.206	0.363	0.672	1.26	2.41	—	—	—
Candidatus Carsonella ruddii	NC_008512 (Gene)	0.124	0.215	0.383	0.715	1.36	2.61	—	—	—
Candidatus Carsonella ruddii	NC_008512 (Intergenic)	0.037	0.056	0.084	0.131	—	—	—	—	—
Candidatus Carsonella ruddii PV	NC_008512	0.119	0.206	0.363	0.672	1.26	2.41	—	—	—
Candidatus Carsonella ruddii PV	NC_008512 (Gene)	0.124	0.215	0.383	0.715	1.36	2.61	—	—	—
Candidatus Carsonella ruddii PV	NC_008512 (Intergenic)	0.037	0.056	0.084	0.131	—	—	—	—	—
Candidatus Pelagibacter ubique HTCC1062	NC_007205	0.201	0.470	1.12	2.78	7.00	18.0	46.9	118	—
Candidatus Pelagibacter ubique HTCC1062	NC_007205 (Gene)	0.195	0.455	1.08	2.71	6.81	17.5	45.5	114	—
Candidatus Pelagibacter ubique HTCC1062	NC_007205 (Intergenic)	0.373	0.831	1.79	3.90	8.13	—	—	—	—
Candidatus Ruthia magnifica Cm Calyptogena magnifica	NC_008610	0.325	0.762	1.83	4.68	12.5	34.0	91.1	230	—
Candidatus Ruthia magnifica Cm Calyptogena magnifica	NC_008610 (Gene)	0.292	0.691	1.69	4.38	11.8	32.2	86.3	—	—
Candidatus Ruthia magnifica Cm Calyptogena magnifica	NC_008610 (Intergenic)	0.482	1.01	2.05	4.77	11.3	26.2	—	—	—
Carboxydothermus hydrogenoformans Z-2901	NC_007503	0.198	0.393	0.964	2.57	7.19	20.9	61.7	177	—

next

		L_e (kb)								
Category	SN \ k	2	3	4	5	6	7	8	9	10
Carboxydothermus hydrogenoformans Z-2901	NC_007503 (Gene)	0.206	0.399	0.977	2.59	7.21	20.9	61.6	177	—
Carboxydothermus hydrogenoformans Z-2901	NC_007503 (Intergenic)	0.128	0.284	0.665	1.71	4.43	10.9	—	—	—
Caulobacter crescentus	NC_002696	0.285	0.429	0.871	2.10	5.35	13.5	36.1	97.8	—
Caulobacter crescentus	NC_002696 (Gene)	0.267	0.389	0.779	1.87	4.76	11.9	31.9	86.2	—
Caulobacter crescentus	NC_002696 (Intergenic)	0.379	0.841	2.00	5.02	12.5	30.0	63.7	—	—
Chlamydia muridarum	NC_002620	0.243	0.584	1.52	4.22	12.3	36.1	104	268	—
Chlamydia muridarum	NC_002620 (Gene)	0.255	0.615	1.61	4.48	13.0	37.9	108	—	—
Chlamydia muridarum	NC_002620 (Intergenic)	0.124	0.276	0.661	1.74	4.72	12.4	—	—	—
Chlamydia trachomatis	NC_000117	0.275	0.644	1.65	4.59	13.3	39.0	111	—	—
Chlamydia trachomatis	NC_000117 (Gene)	0.294	0.690	1.78	4.92	14.3	41.3	116	—	—
Chlamydia trachomatis	NC_000117 (Intergenic)	0.122	0.264	0.633	1.66	4.57	12.1	—	—	—
Chlamydia trachomatis A HAR-13	NC_007429	0.276	0.647	1.66	4.60	13.4	39.2	112	—	—
Chlamydia trachomatis A HAR-13	NC_007429 (Gene)	0.294	0.691	1.78	4.93	14.3	41.3	116	—	—
Chlamydia trachomatis A HAR-13	NC_007429 (Intergenic)	0.125	0.273	0.654	1.72	4.73	12.5	—	—	—
Chlamydophila abortus S26 3	NC_004552	0.440	0.933	2.29	6.00	16.8	47.6	133	332	—
Chlamydophila abortus S26 3	NC_004552 (Gene)	0.461	0.979	2.40	6.29	17.4	49.1	135	—	—
Chlamydophila abortus S26 3	NC_004552 (Intergenic)	0.228	0.464	1.07	2.69	7.02	17.5	—	—	—
Chlamydophila caviae	NC_003361	0.360	0.786	1.96	5.20	14.5	41.2	116	295	—
Chlamydophila caviae	NC_003361 (Gene)	0.373	0.813	2.03	5.37	14.9	41.8	116	291	—
Chlamydophila caviae	NC_003361 (Intergenic)	0.191	0.410	0.955	2.41	6.25	15.7	—	—	—
Chlamydophila felis Fe C-56	NC_007899	0.330	0.744	1.89	5.09	14.4	41.4	117	300	—
Chlamydophila felis Fe C-56	NC_007899 (Gene)	0.346	0.779	1.99	5.33	15.0	42.8	120	300	—
Chlamydophila felis Fe C-56	NC_007899 (Intergenic)	0.165	0.359	0.849	2.18	5.76	14.6	—	—	—
Chlamydophila pneumoniae AR39	NC_002179	0.283	0.679	1.77	4.86	13.9	40.3	116	300	—
Chlamydophila pneumoniae AR39	NC_002179 (Gene)	0.292	0.705	1.84	5.06	14.5	41.7	118	302	—
Chlamydophila pneumoniae AR39	NC_002179 (Intergenic)	0.165	0.369	0.895	2.33	6.22	15.8	—	—	—
Chlamydophila pneumoniae CWL029	NC_000922	0.283	0.679	1.77	4.85	13.9	40.3	115	300	—
Chlamydophila pneumoniae CWL029	NC_000922 (Gene)	0.294	0.711	1.86	5.10	14.6	42.0	119	303	—
Chlamydophila pneumoniae CWL029	NC_000922 (Intergenic)	0.160	0.356	0.864	2.26	6.05	15.5	—	—	—
Chlamydophila pneumoniae J138	NC_002491	0.283	0.678	1.77	4.86	14.0	40.4	116	301	—
Chlamydophila pneumoniae J138	NC_002491 (Gene)	0.294	0.709	1.85	5.08	14.5	41.8	119	302	—
Chlamydophila pneumoniae J138	NC_002491 (Intergenic)	0.167	0.377	0.924	2.44	6.56	16.9	—	—	—
Chlamydophila pneumoniae TW 183	NC_005043	0.284	0.680	1.77	4.86	14.0	40.4	116	300	—
Chlamydophila pneumoniae TW 183	NC_005043 (Gene)	0.295	0.711	1.86	5.10	14.6	42.0	119	303	—

next

		L_e (kb)								
Category	SN \ k	2	3	4	5	6	7	8	9	10
Chlamydophila pneumoniae TW 183	NC_005043 (Intergenic)	0.160	0.360	0.879	2.30	6.16	15.7	—	—	—
Chlorobium chlorochromatii CaD3	NC_007514	0.207	0.472	1.17	3.19	9.09	26.8	79.5	228	—
Chlorobium chlorochromatii CaD3	NC_007514 (Gene)	0.195	0.440	1.10	3.00	8.51	25.0	73.8	210	—
Chlorobium chlorochromatii CaD3	NC_007514 (Intergenic)	0.245	0.565	1.26	3.18	8.55	22.8	54.7	—	—
Chlorobium phaeobacteroides DSM 266	NC_008639	0.283	0.586	1.44	3.91	11.3	33.9	102	287	—
Chlorobium phaeobacteroides DSM 266	NC_008639 (Gene)	0.276	0.565	1.40	3.80	11.0	33.1	100	290	—
Chlorobium phaeobacteroides DSM 266	NC_008639 (Intergenic)	0.267	0.594	1.35	3.34	8.39	19.9	39.7	—	—
Chlorobium tepidum TLS	NC_002932	0.225	0.510	1.29	3.45	9.52	26.8	77.0	216	—
Chlorobium tepidum TLS	NC_002932 (Gene)	0.209	0.474	1.19	3.16	8.64	24.1	68.6	192	—
Chlorobium tepidum TLS	NC_002932 (Intergenic)	0.254	0.585	1.46	3.95	11.1	30.3	—	—	—
Chromobacterium violaceum	NC_005085	0.197	0.345	0.716	1.73	4.47	11.2	29.8	80.6	206
Chromobacterium violaceum	NC_005085 (Gene)	0.188	0.320	0.649	1.56	4.00	9.91	26.2	70.7	179
Chromobacterium violaceum	NC_005085 (Intergenic)	0.268	0.567	1.34	3.40	9.05	24.4	62.9	—	—
Chromohalobacter salexigens DSM 3043	NC_007963	0.249	0.536	1.20	2.98	7.98	21.2	58.5	164	—
Chromohalobacter salexigens DSM 3043	NC_007963 (Gene)	0.235	0.498	1.10	2.71	7.20	18.9	51.8	144	—
Chromohalobacter salexigens DSM 3043	NC_007963 (Intergenic)	0.446	1.06	2.44	6.45	17.4	45.5	104	—	—
Clostridium acetobutylicum	NC_003030	0.597	1.12	2.60	6.61	17.4	45.8	123	323	—
Clostridium acetobutylicum	NC_003030 (Gene)	0.522	1.02	2.37	6.01	15.7	41.1	110	289	—
Clostridium acetobutylicum	NC_003030 (Intergenic)	1.47	1.83	3.73	8.49	19.4	42.6	88.9	—	—
Clostridium novyi NT	NC_008593	0.672	1.15	2.46	5.88	14.3	34.9	87.2	212	—
Clostridium novyi NT	NC_008593 (Gene)	0.594	1.05	2.22	5.26	12.7	30.7	76.7	187	—
Clostridium novyi NT	NC_008593 (Intergenic)	1.36	1.59	3.15	6.93	14.6	29.3	53.1	—	—
Clostridium perfringens	NC_003366	0.376	0.847	1.95	4.78	11.9	29.9	77.3	197	—
Clostridium perfringens	NC_003366 (Gene)	0.337	0.768	1.74	4.23	10.5	26.1	67.4	172	—
Clostridium perfringens	NC_003366 (Intergenic)	0.515	1.02	2.30	5.38	12.1	26.1	53.9	—	—
Clostridium perfringens ATCC 13124	NC_008261	0.385	0.859	1.97	4.83	12.0	30.1	77.8	199	—
Clostridium perfringens ATCC 13124	NC_008261 (Gene)	0.348	0.785	1.77	4.29	10.6	26.4	68.3	174	—
Clostridium perfringens ATCC 13124	NC_008261 (Intergenic)	0.494	0.984	2.21	5.13	11.4	24.8	51.6	—	—
Clostridium perfringens SM101	NC_008262	0.387	0.868	2.00	4.91	12.2	30.5	78.1	196	—
Clostridium perfringens SM101	NC_008262 (Gene)	0.329	0.749	1.70	4.13	10.2	25.3	65.0	164	—
Clostridium perfringens SM101	NC_008262 (Intergenic)	0.604	1.25	2.90	6.84	15.2	32.2	62.5	—	—
Clostridium perfringens SM101	NC_008265	0.087	0.251	0.643	1.64	3.94	—	—	—	—
Clostridium perfringens SM101	NC_008265 (Gene)	0.072	0.203	0.521	1.33	3.18	—	—	—	—
Clostridium perfringens SM101	NC_008265 (Intergenic)	0.139	0.399	0.934	2.04	—	—	—	—	—

next

Category	SN \ k	L_e (kb) 2	3	4	5	6	7	8	9	10
Clostridium tetani E88	NC_004557	0.547	0.901	1.96	4.72	11.6	29.0	73.9	186	—
Clostridium tetani E88	NC_004557 (Gene)	0.498	0.821	1.78	4.27	10.4	25.8	65.7	165	—
Clostridium tetani E88	NC_004557 (Intergenic)	0.980	1.65	3.51	8.18	18.6	39.9	78.4	—	—
Colwellia psychrerythraea 34H	NC_003910	0.565	1.24	2.85	7.13	19.3	54.6	157	444	1136
Colwellia psychrerythraea 34H	NC_003910 (Gene)	0.507	1.10	2.54	6.41	17.4	49.0	141	399	1024
Colwellia psychrerythraea 34H	NC_003910 (Intergenic)	0.820	1.85	3.93	8.71	21.1	51.0	115	—	—
Corynebacterium diphtheriae	NC_002935	0.579	1.40	3.32	8.64	24.3	69.3	196	513	—
Corynebacterium diphtheriae	NC_002935 (Gene)	0.529	1.27	3.01	7.83	22.0	62.5	177	464	—
Corynebacterium diphtheriae	NC_002935 (Intergenic)	0.644	1.39	3.44	9.09	23.5	55.2	—	—	—
Corynebacterium efficiens YS-314	NC_004369	0.472	0.867	1.57	3.59	9.30	23.9	64.0	173	—
Corynebacterium efficiens YS-314	NC_004369 (Gene)	0.455	0.811	1.43	3.26	8.38	21.4	57.1	154	—
Corynebacterium efficiens YS-314	NC_004369 (Intergenic)	0.373	0.840	2.06	5.43	14.6	37.8	84.2	—	—
Corynebacterium glutamicum ATCC 13032 Bielefeld	NC_006958	0.410	0.997	2.44	6.48	18.5	53.7	157	437	—
Corynebacterium glutamicum ATCC 13032 Bielefeld	NC_006958 (Gene)	0.380	0.904	2.16	5.68	16.1	46.4	136	378	—
Corynebacterium glutamicum ATCC 13032 Bielefeld	NC_006958 (Intergenic)	0.345	0.888	2.48	7.06	20.1	54.3	124	—	—
Corynebacterium glutamicum ATCC 13032 Kitasato	NC_003450	0.412	1.00	2.45	6.51	18.5	53.9	158	439	—
Corynebacterium glutamicum ATCC 13032 Kitasato	NC_003450 (Gene)	0.378	0.899	2.15	5.64	15.9	46.1	135	375	—
Corynebacterium glutamicum ATCC 13032 Kitasato	NC_003450 (Intergenic)	0.368	0.952	2.66	7.59	21.7	59.0	136	—	—
Corynebacterium jeikeium K411	NC_007164	0.845	1.49	2.90	6.75	17.5	45.5	122	314	—
Corynebacterium jeikeium K411	NC_007164 (Gene)	0.732	1.27	2.46	5.73	14.9	38.6	104	270	—
Corynebacterium jeikeium K411	NC_007164 (Intergenic)	0.746	1.69	4.23	10.7	26.4	56.5	—	—	—
Coxiella burnetii	NC_002971	0.278	0.663	1.68	4.62	13.3	39.5	115	308	—
Coxiella burnetii	NC_002971 (Gene)	0.260	0.621	1.59	4.38	12.6	37.6	110	301	—
Coxiella burnetii	NC_002971 (Intergenic)	0.390	0.923	2.08	5.01	11.6	23.4	—	—	—
Cyanobacteria bacterium Yellowstone A-Prime	NC_007775	0.244	0.555	1.29	3.19	8.25	21.1	57.1	157	—
Cyanobacteria bacterium Yellowstone A-Prime	NC_007775 (Gene)	0.247	0.540	1.22	2.97	7.63	19.5	52.5	143	—
Cyanobacteria bacterium Yellowstone A-Prime	NC_007775 (Intergenic)	0.181	0.471	1.25	3.38	8.83	21.8	50.2	—	—
Cyanobacteria bacterium Yellowstone B-Prime	NC_007776	0.228	0.530	1.29	3.26	8.42	21.6	58.3	160	—
Cyanobacteria bacterium Yellowstone B-Prime	NC_007776 (Gene)	0.222	0.502	1.21	3.00	7.77	20.0	54.5	150	—
Cyanobacteria bacterium Yellowstone B-Prime	NC_007776 (Intergenic)	0.220	0.568	1.50	3.95	9.67	21.9	46.3	—	—
Cytophaga hutchinsonii ATCC 33406	NC_008255	0.450	0.728	1.75	4.63	12.9	37.3	109	312	830
Cytophaga hutchinsonii ATCC 33406	NC_008255 (Gene)	0.424	0.681	1.65	4.37	12.2	35.2	102	294	—
Cytophaga hutchinsonii ATCC 33406	NC_008255 (Intergenic)	0.573	1.04	2.20	5.06	12.5	30.4	69.0	—	—
Dechloromonas aromatica RCB	NC_007298	0.284	0.514	1.23	3.20	8.90	25.2	73.5	213	579

next

Category	SN \ k	L_e (kb) 2	3	4	5	6	7	8	9	10
Dechloromonas aromatica RCB	NC_007298 (Gene)	0.272	0.482	1.14	2.95	8.16	23.0	66.5	192	—
Dechloromonas aromatica RCB	NC_007298 (Intergenic)	0.352	0.863	2.24	6.21	17.7	48.6	115	—	—
Dehalococcoides CBDB1	NC_007356	0.518	0.742	1.67	4.14	11.3	32.6	94.1	254	—
Dehalococcoides CBDB1	NC_007356 (Gene)	0.498	0.691	1.55	3.85	10.5	30.1	86.6	233	—
Dehalococcoides CBDB1	NC_007356 (Intergenic)	0.567	1.13	2.40	5.60	13.6	31.3	—	—	—
Dehalococcoides ethenogenes 195	NC_002936	0.523	0.689	1.51	3.71	10.0	28.5	81.8	222	—
Dehalococcoides ethenogenes 195	NC_002936 (Gene)	0.509	0.648	1.41	3.47	9.36	26.5	75.7	204	—
Dehalococcoides ethenogenes 195	NC_002936 (Intergenic)	0.523	1.02	2.23	5.34	13.1	30.1	—	—	—
Deinococcus geothermalis DSM 11300	NC_008025	0.691	1.22	2.23	5.00	11.6	27.8	71.8	186	—
Deinococcus geothermalis DSM 11300	NC_008025 (Gene)	0.639	1.11	2.01	4.50	10.4	24.8	64.0	166	—
Deinococcus geothermalis DSM 11300	NC_008025 (Intergenic)	0.451	1.01	2.45	6.21	15.5	37.7	—	—	—
Deinococcus radiodurans	NC_001263	0.403	0.861	1.58	3.46	7.55	17.2	42.5	109	—
Deinococcus radiodurans	NC_001263 (Gene)	0.357	0.756	1.38	3.01	6.62	15.1	37.3	95.5	—
Deinococcus radiodurans	NC_001263 (Intergenic)	0.376	0.897	2.24	5.60	12.7	28.6	—	—	—
Deinococcus radiodurans	NC_001264	0.390	0.826	1.52	3.36	7.37	16.7	39.5	—	—
Deinococcus radiodurans	NC_001264 (Gene)	0.362	0.761	1.39	3.07	6.75	15.3	36.2	—	—
Deinococcus radiodurans	NC_001264 (Intergenic)	0.315	0.785	1.92	4.48	9.00	—	—	—	—
Desulfitobacterium hafniense Y51	NC_007907	0.287	0.684	1.84	5.17	15.4	47.4	147	447	1216
Desulfitobacterium hafniense Y51	NC_007907 (Gene)	0.271	0.635	1.71	4.78	14.1	43.3	134	406	1100
Desulfitobacterium hafniense Y51	NC_007907 (Intergenic)	0.345	0.868	2.27	6.34	18.2	51.4	132	—	—
Desulfotalea psychrophila LSv54	NC_006138	0.352	0.784	1.90	5.09	14.6	43.5	131	380	—
Desulfotalea psychrophila LSv54	NC_006138 (Gene)	0.351	0.775	1.89	5.05	14.4	42.8	128	366	—
Desulfotalea psychrophila LSv54	NC_006138 (Intergenic)	0.268	0.580	1.35	3.53	9.70	27.2	72.1	—	—
Desulfovibrio desulfuricans G20	NC_007519	0.286	0.518	1.26	3.25	8.95	25.6	74.8	217	—
Desulfovibrio desulfuricans G20	NC_007519 (Gene)	0.277	0.509	1.24	3.19	8.77	24.9	72.4	208	—
Desulfovibrio desulfuricans G20	NC_007519 (Intergenic)	0.370	0.493	1.07	2.63	6.98	18.9	49.2	—	—
Desulfovibrio vulgaris Hildenborough	NC_002937	0.521	0.970	1.89	4.30	10.9	28.0	76.4	211	—
Desulfovibrio vulgaris Hildenborough	NC_002937 (Gene)	0.482	0.897	1.72	3.89	9.82	25.0	67.5	185	—
Desulfovibrio vulgaris Hildenborough	NC_002937 (Intergenic)	0.801	1.29	2.57	6.06	15.2	39.7	98.5	—	—
Ehrlichia canis Jake	NC_007354	1.20	2.62	4.75	10.6	26.2	64.5	159	358	—
Ehrlichia canis Jake	NC_007354 (Gene)	0.952	2.06	3.76	8.61	21.3	52.7	130	—	—
Ehrlichia canis Jake	NC_007354 (Intergenic)	0.784	1.64	3.45	7.63	17.7	40.2	85.7	—	—
Ehrlichia chaffeensis Arkansas	NC_007799	1.32	2.71	4.93	11.0	27.5	67.8	166	363	—
Ehrlichia chaffeensis Arkansas	NC_007799 (Gene)	1.06	2.22	4.11	9.39	23.3	57.7	141	—	—

next

Category	SN \ k		L_e (kb)								
			2	3	4	5	6	7	8	9	10
Ehrlichia chaffeensis Arkansas	NC_007799	(Intergenic)	0.972	1.95	3.96	8.66	19.4	41.3	—	—	—
Ehrlichia ruminantium Gardel	NC_006831		1.27	2.66	4.80	10.6	26.1	63.8	155	352	—
Ehrlichia ruminantium Gardel	NC_006831	(Gene)	1.29	2.59	4.38	9.66	23.6	57.4	140	—	—
Ehrlichia ruminantium Gardel	NC_006831	(Intergenic)	0.470	1.03	2.27	5.25	12.5	29.6	67.4	—	—
Ehrlichia ruminantium str. Welgevonden	NC_006832		1.26	2.64	4.75	10.5	25.9	63.3	154	346	—
Ehrlichia ruminantium str. Welgevonden	NC_006832	(Gene)	1.28	2.58	4.35	9.63	23.4	56.9	138	—	—
Ehrlichia ruminantium str. Welgevonden	NC_006832	(Intergenic)	0.463	1.01	2.23	5.17	12.4	29.4	67.1	—	—
Ehrlichia ruminantium Welgevonden	NC_005295		1.26	2.64	4.75	10.5	25.9	63.1	153	344	—
Ehrlichia ruminantium Welgevonden	NC_005295	(Gene)	1.27	2.56	4.32	9.55	23.2	56.4	137	—	—
Ehrlichia ruminantium Welgevonden	NC_005295	(Intergenic)	0.469	1.02	2.23	5.14	12.3	29.2	66.5	—	—
Enterococcus faecalis V583	NC_004668		0.219	0.561	1.47	4.01	11.3	32.7	94.8	269	—
Enterococcus faecalis V583	NC_004668	(Gene)	0.221	0.567	1.51	4.12	11.6	33.0	94.9	266	—
Enterococcus faecalis V583	NC_004668	(Intergenic)	0.151	0.323	0.737	1.86	5.02	13.8	36.0	—	—
Erwinia carotovora atroseptica SCRI1043	NC_004547		0.397	0.805	1.97	5.18	14.7	43.6	132	392	1053
Erwinia carotovora atroseptica SCRI1043	NC_004547	(Gene)	0.350	0.700	1.73	4.56	12.9	38.1	115	342	924
Erwinia carotovora atroseptica SCRI1043	NC_004547	(Intergenic)	0.905	1.80	3.36	7.80	19.8	50.8	119	—	—
Erythrobacter litoralis HTCC2594	NC_007722		0.155	0.351	0.909	2.39	6.60	18.4	52.0	148	—
Erythrobacter litoralis HTCC2594	NC_007722	(Gene)	0.142	0.320	0.823	2.15	5.93	16.4	46.2	131	—
Erythrobacter litoralis HTCC2594	NC_007722	(Intergenic)	0.402	0.950	2.45	6.56	17.5	44.2	—	—	—
Escherichia coli 536	NC_008253		0.391	0.756	1.79	4.66	13.0	38.4	116	344	934
Escherichia coli 536	NC_008253	(Gene)	0.360	0.682	1.62	4.21	11.7	34.4	104	307	837
Escherichia coli 536	NC_008253	(Intergenic)	0.632	1.34	2.81	6.85	17.7	45.2	103	—	—
Escherichia coli APEC O1	NC_008563		0.383	0.745	1.77	4.61	12.9	38.1	115	343	940
Escherichia coli APEC O1	NC_008563	(Gene)	0.350	0.666	1.59	4.12	11.5	33.7	102	302	831
Escherichia coli APEC O1	NC_008563	(Intergenic)	0.600	1.32	2.93	7.32	19.5	51.7	123	—	—
Escherichia coli CFT073	NC_004431		0.399	0.768	1.82	4.73	13.2	39.1	118	352	959
Escherichia coli CFT073	NC_004431	(Gene)	0.368	0.698	1.66	4.31	12.0	35.4	107	317	870
Escherichia coli CFT073	NC_004431	(Intergenic)	0.708	1.45	3.01	7.33	18.9	48.1	108	—	—
Escherichia coli K12	NC_000913		0.373	0.729	1.74	4.52	12.6	37.0	111	328	879
Escherichia coli K12	NC_000913	(Gene)	0.346	0.656	1.56	4.05	11.3	33.0	98.9	292	—
Escherichia coli K12	NC_000913	(Intergenic)	0.553	1.22	2.60	6.33	16.0	39.3	83.9	—	—
Escherichia coli O157H7	NC_002695		0.404	0.774	1.83	4.75	13.3	39.2	119	352	959
Escherichia coli O157H7	NC_002695	(Gene)	0.374	0.698	1.65	4.29	11.9	35.1	106	313	851
Escherichia coli O157H7	NC_002695	(Intergenic)	0.569	1.23	2.70	6.73	18.1	48.7	120	—	—

next

Category	SN \ k		L_e (kb) 2	3	4	5	6	7	8	9	10
Escherichia coli O157H7 EDL933	NC_002655		0.407	0.778	1.84	4.78	13.4	39.5	119	355	965
Escherichia coli O157H7 EDL933	NC_002655 (Gene)		0.376	0.704	1.67	4.33	12.1	35.5	107	316	861
Escherichia coli O157H7 EDL933	NC_002655 (Intergenic)		0.583	1.24	2.65	6.53	17.3	45.8	110	—	—
Escherichia coli UTI89	NC_007946		0.385	0.748	1.78	4.63	12.9	38.2	115	344	943
Escherichia coli UTI89	NC_007946 (Gene)		0.357	0.684	1.63	4.24	11.8	34.7	105	311	856
Escherichia coli UTI89	NC_007946 (Intergenic)		0.638	1.36	2.89	7.07	18.4	47.5	109	—	—
Escherichia coli W3110	AC 000091		0.373	0.729	1.74	4.52	12.6	37.1	111	328	877
Escherichia coli W3110	AC 000091 (Gene)		0.346	0.655	1.56	4.05	11.3	33.0	98.9	291	—
Escherichia coli W3110	AC 000091 (Intergenic)		0.557	1.23	2.62	6.36	16.1	39.6	84.5	—	—
Francisella tularensis FSC 198	NC_008245		0.714	1.20	2.58	6.25	16.0	41.3	107	258	—
Francisella tularensis FSC 198	NC_008245 (Gene)		0.666	1.13	2.43	5.92	15.1	39.2	102	246	—
Francisella tularensis FSC 198	NC_008245 (Intergenic)		0.884	1.45	3.01	6.50	13.8	26.6	—	—	—
Francisella tularensis holarctica	NC_007880		0.715	1.20	2.58	6.24	15.9	40.8	103	239	—
Francisella tularensis holarctica	NC_007880 (Gene)		0.665	1.13	2.43	5.91	15.1	38.5	97.6	225	—
Francisella tularensis holarctica	NC_007880 (Intergenic)		0.978	1.58	3.23	7.01	15.2	30.6	—	—	—
Francisella tularensis holarctica OSU18	NC_008369		0.716	1.20	2.58	6.25	15.9	40.8	103	239	—
Francisella tularensis holarctica OSU18	NC_008369 (Gene)		0.673	1.14	2.45	5.95	15.2	38.9	98.6	228	—
Francisella tularensis holarctica OSU18	NC_008369 (Intergenic)		0.894	1.47	3.08	6.68	14.6	29.6	—	—	—
Francisella tularensis novicida U112	NC_008601		0.695	1.18	2.55	6.20	15.9	41.5	110	280	—
Francisella tularensis novicida U112	NC_008601 (Gene)		0.648	1.12	2.41	5.87	15.1	39.3	104	265	—
Francisella tularensis novicida U112	NC_008601 (Intergenic)		0.976	1.58	3.15	6.88	14.7	29.7	—	—	—
Francisella tularensis tularensis	NC_006570		0.713	1.20	2.58	6.25	16.0	41.3	107	258	—
Francisella tularensis tularensis	NC_006570 (Gene)		0.666	1.13	2.43	5.92	15.1	39.2	102	246	—
Francisella tularensis tularensis	NC_006570 (Intergenic)		0.885	1.46	3.02	6.53	13.9	27.0	—	—	—
Frankia alni ACN14a	NC_008278		0.274	0.493	0.911	2.02	5.07	12.2	31.0	83.0	209
Frankia alni ACN14a	NC_008278 (Gene)		0.253	0.461	0.831	1.83	4.54	10.8	27.2	72.3	180
Frankia alni ACN14a	NC_008278 (Intergenic)		0.406	0.690	1.50	3.57	9.00	22.9	57.8	—	—
Frankia CcI3	NC_007777		0.364	0.635	1.26	2.97	7.71	19.5	52.0	143	367
Frankia CcI3	NC_007777 (Gene)		0.334	0.587	1.14	2.65	6.82	17.0	45.0	122	312
Frankia CcI3	NC_007777 (Intergenic)		0.550	0.939	2.14	5.47	14.6	38.9	99.8	—	—
Fusobacterium nucleatum	NC_003454		0.244	0.512	1.10	2.68	6.48	15.8	39.8	99.9	—
Fusobacterium nucleatum	NC_003454 (Gene)		0.240	0.508	1.10	2.68	6.44	15.6	39.3	98.6	—
Fusobacterium nucleatum	NC_003454 (Intergenic)		0.253	0.467	0.928	2.05	4.63	10.3	—	—	—
Geobacillus kaustophilus HTA426	NC_006510		0.160	0.379	0.966	2.65	7.63	22.7	68.5	204	—

next

Category	SN \ k	L_e (kb) 2	3	4	5	6	7	8	9	10
Geobacillus kaustophilus HTA426	NC_006510 (Gene)	0.145	0.350	0.898	2.45	7.05	20.9	62.4	184	—
Geobacillus kaustophilus HTA426	NC_006510 (Intergenic)	0.206	0.422	1.01	2.67	7.50	21.2	57.4	—	—
Geobacter metallireducens GS-15	NC_007517	0.464	1.05	2.14	5.28	14.2	37.8	105	291	—
Geobacter metallireducens GS-15	NC_007517 (Gene)	0.448	0.999	2.00	4.89	13.1	34.4	94.5	261	—
Geobacter metallireducens GS-15	NC_007517 (Intergenic)	0.435	0.996	2.31	6.08	16.5	43.9	99.8	—	—
Geobacter sulfurreducens	NC_002939	0.623	1.18	2.36	5.74	15.3	40.5	111	305	—
Geobacter sulfurreducens	NC_002939 (Gene)	0.596	1.11	2.18	5.27	14.0	36.6	100	275	—
Geobacter sulfurreducens	NC_002939 (Intergenic)	0.709	1.37	3.02	7.56	19.3	44.9	84.6	—	—
Gloeobacter violaceus	NC_005125	0.546	0.931	1.94	4.68	12.0	31.1	83.8	228	582
Gloeobacter violaceus	NC_005125 (Gene)	0.518	0.859	1.75	4.18	10.6	27.1	72.5	196	—
Gloeobacter violaceus	NC_005125 (Intergenic)	0.632	1.39	3.52	9.65	27.0	72.2	165	—	—
Gluconobacter oxydans 621H	NC_006677	0.306	0.604	1.50	3.90	10.6	29.5	84.2	235	—
Gluconobacter oxydans 621H	NC_006677 (Gene)	0.287	0.571	1.41	3.67	9.96	27.4	77.7	216	—
Gluconobacter oxydans 621H	NC_006677 (Intergenic)	0.357	0.679	1.70	4.50	12.5	33.9	—	—	—
Granulobacter bethesdensis CGDNIH1	NC_008343	0.280	0.551	1.37	3.58	9.79	27.7	80.2	228	—
Granulobacter bethesdensis CGDNIH1	NC_008343 (Gene)	0.268	0.527	1.31	3.41	9.29	26.2	75.3	213	—
Granulobacter bethesdensis CGDNIH1	NC_008343 (Intergenic)	0.413	0.780	1.89	5.02	13.7	36.1	—	—	—
Haemophilus ducreyi 35000HP	NC_002940	0.341	0.783	1.93	4.97	13.3	37.2	103	271	—
Haemophilus ducreyi 35000HP	NC_002940 (Gene)	0.327	0.746	1.85	4.78	12.8	35.7	99.3	259	—
Haemophilus ducreyi 35000HP	NC_002940 (Intergenic)	0.328	0.695	1.43	3.24	7.67	17.9	—	—	—
Haemophilus influenzae	NC_000907	0.230	0.567	1.52	4.08	11.1	31.1	83.9	210	—
Haemophilus influenzae	NC_000907 (Gene)	0.226	0.557	1.50	4.03	11.1	31.2	86.0	222	—
Haemophilus influenzae	NC_000907 (Intergenic)	0.179	0.377	0.864	2.11	5.07	11.5	—	—	—
Haemophilus influenzae 86 028NP	NC_007146	0.235	0.579	1.55	4.14	11.3	31.6	85.5	215	—
Haemophilus influenzae 86 028NP	NC_007146 (Gene)	0.229	0.565	1.52	4.08	11.2	31.6	87.2	226	—
Haemophilus influenzae 86 028NP	NC_007146 (Intergenic)	0.188	0.391	0.899	2.20	5.29	12.0	—	—	—
Haemophilus somnus 129PT	NC_008309	0.314	0.754	1.93	5.05	13.6	37.7	103	262	—
Haemophilus somnus 129PT	NC_008309 (Gene)	0.308	0.741	1.91	5.02	13.5	37.5	103	264	—
Haemophilus somnus 129PT	NC_008309 (Intergenic)	0.258	0.513	1.11	2.64	6.52	15.5	—	—	—
Hahella chejuensis KCTC 2396	NC_007645	0.439	0.867	1.96	5.03	14.0	40.8	122	361	1002
Hahella chejuensis KCTC 2396	NC_007645 (Gene)	0.398	0.776	1.75	4.47	12.4	35.8	106	316	886
Hahella chejuensis KCTC 2396	NC_007645 (Intergenic)	0.694	1.63	3.66	9.39	25.2	64.9	143	—	—
Haloquadratum walsbyi	NC_008212	0.339	0.775	1.98	5.59	16.5	49.0	146	411	—
Haloquadratum walsbyi	NC_008212 (Gene)	0.277	0.667	1.70	4.75	13.9	40.8	120	339	—

next

		L_e (kb)								
Category	SN \ k	2	3	4	5	6	7	8	9	10
Haloquadratum walsbyi	NC_008212 (Intergenic)	0.616	1.06	2.63	7.16	20.1	54.3	129	—	—
Helicobacter acinonychis Sheeba	NC_008229	0.141	0.277	0.657	1.67	4.50	12.5	35.3	98.7	—
Helicobacter acinonychis Sheeba	NC_008229 (Gene)	0.144	0.282	0.671	1.70	4.58	12.7	35.7	99.2	—
Helicobacter acinonychis Sheeba	NC_008229 (Intergenic)	0.110	0.222	0.503	1.22	3.11	7.91	—	—	—
Helicobacter hepaticus	NC_004917	0.239	0.466	1.08	2.75	7.32	20.0	55.5	152	—
Helicobacter hepaticus	NC_004917 (Gene)	0.233	0.457	1.06	2.71	7.24	19.8	55.0	150	—
Helicobacter hepaticus	NC_004917 (Intergenic)	0.244	0.463	1.01	2.38	5.76	13.6	—	—	—
Helicobacter pylori 26695	NC_000915	0.136	0.269	0.639	1.62	4.36	12.1	34.2	96.1	—
Helicobacter pylori 26695	NC_000915 (Gene)	0.139	0.276	0.656	1.66	4.48	12.4	35.0	97.6	—
Helicobacter pylori 26695	NC_000915 (Intergenic)	0.095	0.191	0.430	1.03	2.61	6.66	—	—	—
Helicobacter pylori HPAG1	NC_008086	0.136	0.269	0.635	1.61	4.32	12.0	33.9	95.0	—
Helicobacter pylori HPAG1	NC_008086 (Gene)	0.139	0.275	0.652	1.65	4.43	12.3	34.6	96.3	—
Helicobacter pylori HPAG1	NC_008086 (Intergenic)	0.096	0.190	0.420	0.999	2.52	6.36	—	—	—
Helicobacter pylori J99	NC_000921	0.140	0.275	0.651	1.65	4.43	12.3	34.9	97.9	—
Helicobacter pylori J99	NC_000921 (Gene)	0.143	0.280	0.663	1.68	4.51	12.5	35.2	98.2	—
Helicobacter pylori J99	NC_000921 (Intergenic)	0.108	0.216	0.488	1.18	2.99	7.63	—	—	—
Hyphomonas neptunium ATCC 15444	NC_008358	0.256	0.550	1.34	3.42	9.16	25.3	71.7	202	—
Hyphomonas neptunium ATCC 15444	NC_008358 (Gene)	0.234	0.500	1.21	3.09	8.28	22.8	64.3	181	—
Hyphomonas neptunium ATCC 15444	NC_008358 (Intergenic)	0.435	1.03	2.51	6.35	16.6	43.2	101	—	—
Idiomarina loihiensis L2TR	NC_006512	0.447	0.958	2.36	6.24	17.8	52.9	158	444	—
Idiomarina loihiensis L2TR	NC_006512 (Gene)	0.433	0.910	2.23	5.90	16.7	49.7	148	415	—
Idiomarina loihiensis L2TR	NC_006512 (Intergenic)	0.461	1.21	2.90	7.49	19.4	45.5	—	—	—
Jannaschia CCS1	NC_007802	0.298	0.654	1.64	4.19	10.9	29.5	82.8	233	619
Jannaschia CCS1	NC_007802 (Gene)	0.276	0.602	1.50	3.82	9.92	26.7	74.5	209	—
Jannaschia CCS1	NC_007802 (Intergenic)	0.577	1.30	3.26	8.48	21.9	56.0	127	—	—
Lactobacillus acidophilus NCFM	NC_006814	0.431	0.949	2.27	5.88	15.7	42.4	115	299	—
Lactobacillus acidophilus NCFM	NC_006814 (Gene)	0.420	0.904	2.15	5.51	14.7	39.4	107	278	—
Lactobacillus acidophilus NCFM	NC_006814 (Intergenic)	0.410	0.782	1.64	3.89	9.60	22.4	—	—	—
Lactobacillus brevis ATCC 367	NC_008497	0.532	0.983	2.50	6.92	20.3	61.3	182	495	—
Lactobacillus brevis ATCC 367	NC_008497 (Gene)	0.498	0.874	2.21	6.13	17.9	53.9	160	432	—
Lactobacillus brevis ATCC 367	NC_008497 (Intergenic)	0.494	1.15	2.63	6.59	17.3	43.8	96.6	—	—
Lactobacillus casei ATCC 334	NC_008526	0.244	0.574	1.55	4.49	13.5	41.8	128	366	—
Lactobacillus casei ATCC 334	NC_008526 (Gene)	0.226	0.519	1.39	4.01	12.0	37.1	113	327	—
Lactobacillus casei ATCC 334	NC_008526 (Intergenic)	0.311	0.757	1.95	5.29	14.3	36.3	77.5	—	—

next

Category	SN \ k	L_e (kb) 2	3	4	5	6	7	8	9	10
Lactobacillus delbrueckii bulgaricus	NC_008054	0.263	0.491	1.18	3.15	8.88	25.5	73.8	203	—
Lactobacillus delbrueckii bulgaricus	NC_008054 (Gene)	0.281	0.479	1.11	2.92	8.11	23.0	66.2	181	—
Lactobacillus delbrueckii bulgaricus	NC_008054 (Intergenic)	0.141	0.319	0.768	2.03	5.64	15.1	—	—	—
Lactobacillus delbrueckii bulgaricus ATCC BAA-365	NC_008529	0.261	0.488	1.18	3.14	8.83	25.4	73.6	203	—
Lactobacillus delbrueckii bulgaricus ATCC BAA-365	NC_008529 (Gene)	0.275	0.471	1.10	2.88	8.02	22.8	65.6	180	—
Lactobacillus delbrueckii bulgaricus ATCC BAA-365	NC_008529 (Intergenic)	0.149	0.342	0.826	2.19	6.08	16.3	—	—	—
Lactobacillus gasseri ATCC 33323	NC_008530	0.334	0.789	1.95	5.13	13.9	38.0	105	275	—
Lactobacillus gasseri ATCC 33323	NC_008530 (Gene)	0.329	0.762	1.87	4.89	13.1	35.8	98.2	256	—
Lactobacillus gasseri ATCC 33323	NC_008530 (Intergenic)	0.283	0.601	1.36	3.38	8.54	20.2	—	—	—
Lactobacillus johnsonii NCC 533	NC_005362	0.372	0.877	2.14	5.58	14.9	40.1	109	282	—
Lactobacillus johnsonii NCC 533	NC_005362 (Gene)	0.368	0.850	2.07	5.35	14.2	37.9	102	263	—
Lactobacillus johnsonii NCC 533	NC_005362 (Intergenic)	0.302	0.626	1.38	3.39	8.52	20.1	—	—	—
Lactobacillus plantarum	NC_004567	0.523	0.939	2.38	6.67	19.5	58.9	177	503	—
Lactobacillus plantarum	NC_004567 (Gene)	0.476	0.842	2.14	5.99	17.5	52.7	158	445	—
Lactobacillus plantarum	NC_004567 (Intergenic)	0.709	1.37	3.13	7.92	21.0	53.8	124	—	—
Lactobacillus sakei 23K	NC_007576	0.357	0.772	1.96	5.28	14.9	43.3	125	335	—
Lactobacillus sakei 23K	NC_007576 (Gene)	0.346	0.731	1.86	5.02	14.1	41.0	118	318	—
Lactobacillus sakei 23K	NC_007576 (Intergenic)	0.371	0.785	1.58	3.56	8.44	19.1	—	—	—
Lactobacillus salivarius UCC118	NC_007929	0.645	1.52	3.20	7.66	19.2	49.0	127	308	—
Lactobacillus salivarius UCC118	NC_007929 (Gene)	0.604	1.39	2.90	6.88	17.1	43.3	112	273	—
Lactobacillus salivarius UCC118	NC_007929 (Intergenic)	0.687	1.39	2.84	6.39	14.3	29.7	—	—	—
Lactococcus lactis	NC_002662	0.172	0.446	1.18	3.27	9.23	26.0	72.5	194	—
Lactococcus lactis	NC_002662 (Gene)	0.167	0.437	1.17	3.25	9.18	26.0	73.6	201	—
Lactococcus lactis	NC_002662 (Intergenic)	0.168	0.349	0.778	1.89	4.63	10.8	22.8	—	—
Lactococcus lactis cremoris SK11	NC_008527	0.180	0.461	1.23	3.39	9.65	27.4	76.4	201	—
Lactococcus lactis cremoris SK11	NC_008527 (Gene)	0.173	0.448	1.21	3.36	9.55	27.2	76.4	203	—
Lactococcus lactis cremoris SK11	NC_008527 (Intergenic)	0.177	0.367	0.809	1.96	4.83	11.5	25.1	—	—
Lawsonia intracellularis PHE MN1-00	NC_008011	0.809	1.59	2.84	6.59	17.1	44.0	115	287	—
Lawsonia intracellularis PHE MN1-00	NC_008011 (Gene)	0.600	1.28	2.45	5.87	15.4	39.9	106	262	—
Lawsonia intracellularis PHE MN1-00	NC_008011 (Intergenic)	0.664	1.18	1.95	4.06	8.88	19.7	—	—	—
Legionella pneumophila Lens	NC_006369	0.291	0.756	2.00	5.44	15.5	45.6	135	381	—
Legionella pneumophila Lens	NC_006369 (Gene)	0.275	0.712	1.89	5.18	14.8	43.6	129	363	—
Legionella pneumophila Lens	NC_006369 (Intergenic)	0.442	1.14	2.76	6.85	17.4	43.0	94.2	—	—
Legionella pneumophila Paris	NC_006368	0.290	0.756	2.00	5.45	15.6	45.8	136	385	—

next

Category	SN \ k		L_e (kb)								
			2	3	4	5	6	7	8	9	10
Legionella pneumophila Paris	NC_006368	(Gene)	0.275	0.716	1.90	5.22	14.9	44.0	130	369	—
Legionella pneumophila Paris	NC_006368	(Intergenic)	0.433	1.09	2.62	6.51	16.6	41.5	92.2	—	—
Legionella pneumophila Philadelphia 1	NC_002942		0.290	0.754	1.99	5.42	15.5	45.5	135	387	—
Legionella pneumophila Philadelphia 1	NC_002942	(Gene)	0.275	0.712	1.89	5.18	14.8	43.7	130	370	—
Legionella pneumophila Philadelphia 1	NC_002942	(Intergenic)	0.442	1.13	2.71	6.75	17.5	44.1	101	—	—
Leifsonia xyli xyli CTCB0	NC_006087		0.210	0.519	1.18	2.92	7.77	19.9	53.1	144	—
Leifsonia xyli xyli CTCB0	NC_006087	(Gene)	0.180	0.445	1.00	2.46	6.52	16.6	44.0	119	—
Leifsonia xyli xyli CTCB0	NC_006087	(Intergenic)	0.456	1.12	2.66	6.65	17.3	42.8	95.0	—	—
Leptospira borgpetersenii serovar Hardjo-bovis JB197	NC_008510		0.101	0.248	0.682	1.97	5.88	17.6	52.3	148	—
Leptospira borgpetersenii serovar Hardjo-bovis JB197	NC_008510	(Gene)	0.097	0.236	0.651	1.88	5.58	16.7	49.5	141	—
Leptospira borgpetersenii serovar Hardjo-bovis JB197	NC_008510	(Intergenic)	0.114	0.279	0.737	2.06	5.87	15.8	37.3	—	—
Leptospira borgpetersenii serovar Hardjo-bovis JB197	NC_008511		0.103	0.251	0.689	1.99	5.85	17.0	46.1	—	—
Leptospira borgpetersenii serovar Hardjo-bovis JB197	NC_008511	(Gene)	0.099	0.240	0.659	1.90	5.54	16.0	—	—	—
Leptospira borgpetersenii serovar Hardjo-bovis JB197	NC_008511	(Intergenic)	0.114	0.282	0.744	2.03	5.35	—	—	—	—
Leptospira borgpetersenii serovar Hardjo-bovis L550	NC_008508		0.101	0.247	0.681	1.97	5.87	17.6	52.3	149	—
Leptospira borgpetersenii serovar Hardjo-bovis L550	NC_008508	(Gene)	0.097	0.236	0.651	1.88	5.58	16.7	49.6	141	—
Leptospira borgpetersenii serovar Hardjo-bovis L550	NC_008508	(Intergenic)	0.114	0.277	0.732	2.05	5.83	15.7	37.2	—	—
Leptospira borgpetersenii serovar Hardjo-bovis L550	NC_008509		0.104	0.254	0.700	2.02	5.94	17.2	46.8	—	—
Leptospira borgpetersenii serovar Hardjo-bovis L550	NC_008509	(Gene)	0.100	0.243	0.666	1.92	5.60	16.2	—	—	—
Leptospira borgpetersenii serovar Hardjo-bovis L550	NC_008509	(Intergenic)	0.115	0.285	0.757	2.08	5.48	—	—	—	—
Leptospira interrogans serovar Copenhageni	NC_005823		0.128	0.294	0.769	2.14	6.11	17.4	49.3	135	344
Leptospira interrogans serovar Copenhageni	NC_005823	(Gene)	0.124	0.284	0.752	2.11	6.06	17.5	50.5	145	—
Leptospira interrogans serovar Copenhageni	NC_005823	(Intergenic)	0.132	0.291	0.690	1.72	4.45	11.1	25.4	—	—
Leptospira interrogans serovar Copenhageni	NC_005824		0.125	0.282	0.734	2.03	5.77	16.1	42.3	—	—
Leptospira interrogans serovar Copenhageni	NC_005824	(Gene)	0.120	0.270	0.707	1.97	5.56	15.4	40.4	—	—
Leptospira interrogans serovar Copenhageni	NC_005824	(Intergenic)	0.132	0.290	0.691	1.72	4.42	10.5	—	—	—
Leptospira interrogans serovar Lai	NC_004342		0.128	0.295	0.771	2.15	6.13	17.5	49.3	135	339
Leptospira interrogans serovar Lai	NC_004342	(Gene)	0.122	0.281	0.743	2.09	5.98	17.2	49.5	141	—
Leptospira interrogans serovar Lai	NC_004342	(Intergenic)	0.143	0.317	0.755	1.88	4.85	12.0	27.6	—	—
Leptospira interrogans serovar Lai	NC_004343		0.126	0.288	0.750	2.08	5.90	16.4	43.3	—	—
Leptospira interrogans serovar Lai	NC_004343	(Gene)	0.119	0.270	0.708	1.97	5.58	15.5	40.8	—	—
Leptospira interrogans serovar Lai	NC_004343	(Intergenic)	0.151	0.339	0.797	1.96	4.93	11.4	—	—	—
Leuconostoc mesenteroides ATCC 8293	NC_008531		0.335	0.843	2.16	5.92	16.9	49.0	140	375	—
Leuconostoc mesenteroides ATCC 8293	NC_008531	(Gene)	0.308	0.765	1.98	5.41	15.4	44.5	127	339	—

next

Category	SN \ k	L_e (kb) 2	3	4	5	6	7	8	9	10
Leuconostoc mesenteroides ATCC 8293	NC_008531 (Intergenic)	0.545	1.09	2.37	5.70	13.9	31.8	—	—	—
Listeria innocua	NC_003212	0.293	0.689	1.79	4.83	13.5	38.6	110	307	—
Listeria innocua	NC_003212 (Gene)	0.293	0.690	1.80	4.85	13.4	37.7	107	294	—
Listeria innocua	NC_003212 (Intergenic)	0.219	0.421	0.944	2.39	6.36	17.0	42.2	—	—
Listeria monocytogenes	NC_003210	0.295	0.685	1.78	4.83	13.6	39.1	113	315	—
Listeria monocytogenes	NC_003210 (Gene)	0.292	0.681	1.78	4.81	13.4	38.1	109	300	—
Listeria monocytogenes	NC_003210 (Intergenic)	0.228	0.427	0.953	2.40	6.38	17.0	42.3	—	—
Listeria monocytogenes 4b F2365	NC_002973	0.294	0.681	1.76	4.79	13.5	38.9	112	313	—
Listeria monocytogenes 4b F2365	NC_002973 (Gene)	0.291	0.677	1.77	4.77	13.3	37.9	108	299	—
Listeria monocytogenes 4b F2365	NC_002973 (Intergenic)	0.220	0.416	0.929	2.35	6.27	16.8	42.0	—	—
Listeria welshimeri serovar 6b SLCC5334	NC_008555	0.293	0.689	1.76	4.71	13.1	36.9	104	288	—
Listeria welshimeri serovar 6b SLCC5334	NC_008555 (Gene)	0.290	0.685	1.76	4.68	12.8	35.7	100	273	—
Listeria welshimeri serovar 6b SLCC5334	NC_008555 (Intergenic)	0.235	0.454	1.02	2.59	6.86	18.2	44.7	—	—
Magnetococcus MC-1	NC_008576	0.313	0.655	1.67	4.48	12.6	36.5	107	307	783
Magnetococcus MC-1	NC_008576 (Gene)	0.302	0.625	1.59	4.21	11.7	33.5	97.5	277	—
Magnetococcus MC-1	NC_008576 (Intergenic)	0.326	0.663	1.61	4.34	12.1	33.2	80.7	—	—
Magnetospirillum magneticum AMB-1	NC_007626	0.460	0.614	1.19	2.72	6.67	16.3	42.3	112	279
Magnetospirillum magneticum AMB-1	NC_007626 (Gene)	0.445	0.550	1.03	2.36	5.73	13.9	35.9	94.4	235
Magnetospirillum magneticum AMB-1	NC_007626 (Intergenic)	0.471	1.16	2.97	7.76	20.8	54.9	130	—	—
Mannheimia succiniciproducens MBEL55E	NC_006300	0.294	0.600	1.42	3.61	9.85	28.1	79.0	209	—
Mannheimia succiniciproducens MBEL55E	NC_006300 (Gene)	0.290	0.577	1.36	3.46	9.47	27.3	78.5	216	—
Mannheimia succiniciproducens MBEL55E	NC_006300 (Intergenic)	0.205	0.435	0.964	2.29	5.41	11.9	—	—	—
Maricaulis maris MCS10	NC_008347	0.262	0.493	1.18	3.06	8.28	22.8	64.7	183	—
Maricaulis maris MCS10	NC_008347 (Gene)	0.241	0.443	1.05	2.72	7.34	20.2	57.0	160	—
Maricaulis maris MCS10	NC_008347 (Intergenic)	0.507	1.23	2.97	7.48	18.8	46.4	98.0	—	—
Mesoplasma florum L1	NC_006055	0.193	0.505	1.22	3.06	7.63	18.7	46.3	—	—
Mesoplasma florum L1	NC_006055 (Gene)	0.190	0.497	1.20	2.99	7.38	17.9	44.2	—	—
Mesoplasma florum L1	NC_006055 (Intergenic)	0.161	0.304	0.626	1.38	3.07	—	—	—	—
Mesorhizobium BNC1	NC_008254	0.218	0.507	1.31	3.52	10.1	29.2	86.2	254	695
Mesorhizobium BNC1	NC_008254 (Gene)	0.204	0.472	1.21	3.25	9.22	26.6	77.9	228	—
Mesorhizobium BNC1	NC_008254 (Intergenic)	0.337	0.825	2.19	6.13	17.7	50.0	125	—	—
Mesorhizobium loti	NC_002678	0.174	0.359	0.841	2.16	5.94	16.3	46.4	134	371
Mesorhizobium loti	NC_002678 (Gene)	0.157	0.320	0.737	1.88	5.12	13.9	39.2	112	309
Mesorhizobium loti	NC_002678 (Intergenic)	0.319	0.755	1.99	5.56	16.2	47.3	131	—	—

next

Category	SN \ k	L_e (kb) 2	3	4	5	6	7	8	9	10
Methylobacillus flagellatus KT	NC_007947	0.235	0.560	1.43	3.88	11.2	32.9	97.8	282	—
Methylobacillus flagellatus KT	NC_007947 (Gene)	0.226	0.530	1.34	3.61	10.4	30.2	89.3	257	—
Methylobacillus flagellatus KT	NC_007947 (Intergenic)	0.322	0.852	2.25	6.21	17.0	41.8	80.5	—	—
Methylococcus capsulatus Bath	NC_002977	0.391	0.664	1.45	3.64	9.86	26.6	74.6	209	—
Methylococcus capsulatus Bath	NC_002977 (Gene)	0.377	0.627	1.35	3.37	9.08	24.3	67.8	189	—
Methylococcus capsulatus Bath	NC_002977 (Intergenic)	0.425	0.902	2.22	5.88	16.1	41.5	89.9	—	—
Moorella thermoacetica ATCC 39073	NC_007644	0.427	0.660	1.33	3.21	8.62	24.2	69.0	195	—
Moorella thermoacetica ATCC 39073	NC_007644 (Gene)	0.435	0.628	1.24	2.94	7.82	21.7	61.4	172	—
Moorella thermoacetica ATCC 39073	NC_007644 (Intergenic)	0.316	0.699	1.62	4.25	11.6	31.0	73.8	—	—
Mycobacterium avium 104	NC_008595	0.266	0.472	0.913	2.09	5.29	13.0	33.8	91.3	231
Mycobacterium avium 104	NC_008595 (Gene)	0.254	0.443	0.843	1.91	4.83	11.7	30.5	82.0	207
Mycobacterium avium 104	NC_008595 (Intergenic)	0.507	1.12	2.63	6.47	16.6	41.5	93.3	—	—
Mycobacterium avium paratuberculosis	NC_002944	0.264	0.461	0.881	2.00	5.04	12.3	31.9	85.9	217
Mycobacterium avium paratuberculosis	NC_002944 (Gene)	0.254	0.437	0.819	1.85	4.64	11.2	29.0	77.7	195
Mycobacterium avium paratuberculosis	NC_002944 (Intergenic)	0.413	0.877	2.03	4.92	12.7	32.1	75.5	—	—
Mycobacterium bovis	NC_002945	0.335	0.575	1.23	2.98	7.96	21.3	58.5	162	412
Mycobacterium bovis	NC_002945 (Gene)	0.319	0.540	1.14	2.75	7.31	19.4	53.1	146	—
Mycobacterium bovis	NC_002945 (Intergenic)	0.626	1.29	3.08	7.86	20.8	52.0	111	—	—
Mycobacterium leprae	NC_002677	0.620	1.08	2.47	6.54	18.7	54.9	162	453	—
Mycobacterium leprae	NC_002677 (Gene)	0.530	0.924	2.11	5.54	15.8	45.9	135	378	—
Mycobacterium leprae	NC_002677 (Intergenic)	1.06	1.83	4.31	11.5	32.3	86.0	194	—	—
Mycobacterium MCS	NC_008146	0.193	0.410	0.863	2.01	5.25	13.4	35.6	98.7	259
Mycobacterium MCS	NC_008146 (Gene)	0.184	0.389	0.810	1.88	4.88	12.3	32.7	90.5	237
Mycobacterium MCS	NC_008146 (Intergenic)	0.418	0.995	2.54	6.61	17.8	46.8	108	—	—
Mycobacterium smegmatis MC2 155	NC_008596	0.188	0.424	0.929	2.22	5.85	15.1	40.6	114	302
Mycobacterium smegmatis MC2 155	NC_008596 (Gene)	0.177	0.397	0.861	2.04	5.36	13.8	36.9	103	272
Mycobacterium smegmatis MC2 155	NC_008596 (Intergenic)	0.512	1.24	3.27	8.87	24.4	64.3	147	—	—
Mycobacterium tuberculosis CDC1551	NC_002755	0.334	0.575	1.23	2.98	7.98	21.3	58.7	163	417
Mycobacterium tuberculosis CDC1551	NC_002755 (Gene)	0.319	0.544	1.15	2.78	7.42	19.8	54.2	150	—
Mycobacterium tuberculosis CDC1551	NC_002755 (Intergenic)	0.690	1.39	3.30	8.33	21.6	51.7	104	—	—
Mycobacterium tuberculosis H37Rv	NC_000962	0.336	0.576	1.23	2.99	7.98	21.3	58.7	163	415
Mycobacterium tuberculosis H37Rv	NC_000962 (Gene)	0.319	0.540	1.14	2.75	7.32	19.5	53.3	147	—
Mycobacterium tuberculosis H37Rv	NC_000962 (Intergenic)	0.636	1.31	3.14	8.04	21.3	53.0	113	—	—
Mycobacterium ulcerans Agy99	NC_008611	0.361	0.607	1.31	3.19	8.48	22.5	60.0	153	324

next

Category	SN \ k	L_e (kb) 2	3	4	5	6	7	8	9	10
Mycobacterium ulcerans Agy99	NC_008611 (Gene)	0.338	0.568	1.21	2.92	7.73	20.4	54.3	139	303
Mycobacterium ulcerans Agy99	NC_008611 (Intergenic)	0.725	1.25	2.99	7.48	18.4	38.9	63.3	—	—
Mycoplasma capricolum ATCC 27343	NC_007633	0.214	0.477	1.09	2.60	6.18	14.3	34.4	—	—
Mycoplasma capricolum ATCC 27343	NC_007633 (Gene)	0.204	0.455	1.04	2.50	5.92	13.6	32.8	—	—
Mycoplasma capricolum ATCC 27343	NC_007633 (Intergenic)	0.241	0.433	0.856	1.78	3.79	7.84	—	—	—
Mycoplasma gallisepticum	NC_004829	0.380	0.666	1.45	3.53	9.06	23.5	60.6	—	—
Mycoplasma gallisepticum	NC_004829 (Gene)	0.394	0.657	1.41	3.42	8.77	22.8	59.3	—	—
Mycoplasma gallisepticum	NC_004829 (Intergenic)	0.219	0.514	1.13	2.47	5.27	9.45	—	—	—
Mycoplasma genitalium	NC_000908	0.141	0.336	0.816	2.11	5.60	14.9	39.4	—	—
Mycoplasma genitalium	NC_000908 (Gene)	0.140	0.333	0.809	2.09	5.54	14.7	39.0	—	—
Mycoplasma genitalium	NC_000908 (Intergenic)	0.135	0.327	0.777	1.85	4.14	—	—	—	—
Mycoplasma hyopneumoniae 232	NC_006360	0.081	0.179	0.412	1.05	2.82	7.71	21.3	—	—
Mycoplasma hyopneumoniae 232	NC_006360 (Gene)	0.084	0.189	0.445	1.16	3.13	8.61	23.8	—	—
Mycoplasma hyopneumoniae 232	NC_006360 (Intergenic)	0.055	0.097	0.187	0.396	0.913	2.17	—	—	—
Mycoplasma hyopneumoniae 7448	NC_007332	0.081	0.180	0.415	1.06	2.85	7.79	21.5	—	—
Mycoplasma hyopneumoniae 7448	NC_007332 (Gene)	0.085	0.193	0.455	1.18	3.21	8.83	24.4	—	—
Mycoplasma hyopneumoniae 7448	NC_007332 (Intergenic)	0.056	0.105	0.207	0.454	1.08	2.66	—	—	—
Mycoplasma hyopneumoniae J	NC_007295	0.082	0.182	0.417	1.06	2.86	7.83	21.7	—	—
Mycoplasma hyopneumoniae J	NC_007295 (Gene)	0.086	0.195	0.460	1.20	3.26	8.96	24.8	—	—
Mycoplasma hyopneumoniae J	NC_007295 (Intergenic)	0.052	0.095	0.181	0.384	0.893	2.14	—	—	—
Mycoplasma mobile 163K	NC_006908	0.098	0.230	0.559	1.44	3.73	9.41	24.0	—	—
Mycoplasma mobile 163K	NC_006908 (Gene)	0.096	0.226	0.552	1.43	3.68	9.27	23.7	—	—
Mycoplasma mobile 163K	NC_006908 (Intergenic)	0.119	0.242	0.531	1.23	2.78	5.76	—	—	—
Mycoplasma mycoides	NC_005364	0.217	0.488	1.12	2.69	6.36	14.6	34.0	72.4	—
Mycoplasma mycoides	NC_005364 (Gene)	0.210	0.470	1.08	2.57	6.03	13.8	32.6	—	—
Mycoplasma mycoides	NC_005364 (Intergenic)	0.229	0.465	1.03	2.40	5.49	11.6	—	—	—
Mycoplasma penetrans	NC_004432	0.230	0.569	1.34	3.26	7.81	18.5	45.4	109	—
Mycoplasma penetrans	NC_004432 (Gene)	0.224	0.558	1.30	3.16	7.56	17.9	44.2	106	—
Mycoplasma penetrans	NC_004432 (Intergenic)	0.206	0.432	0.956	2.05	4.34	8.71	—	—	—
Mycoplasma pneumoniae	NC_000912	0.165	0.418	1.15	3.23	9.23	26.3	72.4	—	—
Mycoplasma pneumoniae	NC_000912 (Gene)	0.175	0.438	1.19	3.35	9.52	27.0	73.6	—	—
Mycoplasma pneumoniae	NC_000912 (Intergenic)	0.097	0.248	0.668	1.80	4.66	11.3	—	—	—
Mycoplasma pulmonis	NC_002771	0.085	0.194	0.465	1.21	3.23	8.45	22.1	—	—
Mycoplasma pulmonis	NC_002771 (Gene)	0.087	0.202	0.488	1.29	3.44	8.97	23.5	—	—

next

Category	SN \ k	L_e (kb) 2	3	4	5	6	7	8	9	10
Mycoplasma pulmonis	NC_002771 (Intergenic)	0.064	0.121	0.252	0.572	1.35	3.19	—	—	—
Mycoplasma synoviae 53	NC_007294	0.140	0.325	0.813	2.12	5.49	13.8	34.4	—	—
Mycoplasma synoviae 53	NC_007294 (Gene)	0.143	0.336	0.840	2.18	5.62	14.1	35.1	—	—
Mycoplasma synoviae 53	NC_007294 (Intergenic)	0.095	0.188	0.415	0.998	2.40	—	—	—	—
Myxococcus xanthus DK 1622	NC_008095	0.554	0.897	1.47	3.40	8.60	20.5	53.7	146	373
Myxococcus xanthus DK 1622	NC_008095 (Gene)	0.506	0.817	1.33	3.06	7.71	18.3	47.6	129	329
Myxococcus xanthus DK 1622	NC_008095 (Intergenic)	1.14	2.07	4.29	10.4	26.2	65.5	153	—	—
Neisseria gonorrhoeae FA 1090	NC_002946	0.193	0.401	0.897	2.23	5.96	16.3	43.9	109	—
Neisseria gonorrhoeae FA 1090	NC_002946 (Gene)	0.181	0.383	0.862	2.13	5.71	15.8	44.3	121	—
Neisseria gonorrhoeae FA 1090	NC_002946 (Intergenic)	0.216	0.418	0.894	2.13	5.19	11.5	21.3	—	—
Neisseria meningitidis MC58	NC_003112	0.207	0.438	0.987	2.47	6.60	17.9	46.5	108	—
Neisseria meningitidis MC58	NC_003112 (Gene)	0.195	0.417	0.946	2.36	6.37	17.7	50.2	138	—
Neisseria meningitidis MC58	NC_003112 (Intergenic)	0.213	0.417	0.839	1.79	3.58	6.14	8.93	—	—
Neisseria meningitidis Z2491	NC_003116	0.201	0.428	0.968	2.42	6.46	17.4	45.1	104	—
Neisseria meningitidis Z2491	NC_003116 (Gene)	0.190	0.407	0.923	2.30	6.20	17.2	48.7	134	—
Neisseria meningitidis Z2491	NC_003116 (Intergenic)	0.209	0.413	0.825	1.75	3.43	5.75	8.24	—	—
Neorickettsia sennetsu Miyayama	NC_007798	0.542	1.14	2.86	7.98	23.5	68.4	184	—	—
Neorickettsia sennetsu Miyayama	NC_007798 (Gene)	0.502	1.08	2.69	7.52	22.0	63.7	170	—	—
Neorickettsia sennetsu Miyayama	NC_007798 (Intergenic)	0.835	1.53	3.72	9.66	23.4	46.9	—	—	—
Nitrobacter hamburgensis X14	NC_007964	0.149	0.361	0.937	2.52	7.18	20.4	59.1	172	464
Nitrobacter hamburgensis X14	NC_007964 (Gene)	0.134	0.320	0.816	2.16	6.11	17.2	49.3	143	—
Nitrobacter hamburgensis X14	NC_007964 (Intergenic)	0.273	0.697	1.93	5.44	15.4	41.0	94.2	—	—
Nitrobacter winogradskyi Nb-255	NC_007406	0.140	0.338	0.875	2.35	6.66	18.9	54.3	155	—
Nitrobacter winogradskyi Nb-255	NC_007406 (Gene)	0.127	0.304	0.772	2.04	5.75	16.1	46.2	132	—
Nitrobacter winogradskyi Nb-255	NC_007406 (Intergenic)	0.228	0.581	1.59	4.36	11.6	28.2	57.8	—	—
Nitrosococcus oceani ATCC 19707	NC_007484	0.544	1.14	2.85	7.76	22.6	68.5	208	591	—
Nitrosococcus oceani ATCC 19707	NC_007484 (Gene)	0.521	1.07	2.68	7.30	21.1	63.9	193	543	—
Nitrosococcus oceani ATCC 19707	NC_007484 (Intergenic)	0.551	1.30	3.19	8.55	23.7	63.7	148	—	—
Nitrosomonas europaea	NC_004757	0.327	0.604	1.52	4.20	12.3	37.2	113	324	—
Nitrosomonas europaea	NC_004757 (Gene)	0.307	0.567	1.43	3.94	11.5	34.6	104	298	—
Nitrosomonas europaea	NC_004757 (Intergenic)	0.451	0.853	2.08	5.59	15.4	41.2	93.6	—	—
Nitrosomonas eutropha C71	NC_008344	0.330	0.704	1.84	5.18	15.3	46.8	142	404	—
Nitrosomonas eutropha C71	NC_008344 (Gene)	0.303	0.647	1.70	4.78	14.2	43.1	131	369	—
Nitrosomonas eutropha C71	NC_008344 (Intergenic)	0.541	1.14	2.83	7.49	20.4	53.4	118	—	—

next

Category	SN \ k		L_e (kb)								
			2	3	4	5	6	7	8	9	10
Nitrosospira multiformis ATCC 25196	NC_007614		0.368	0.806	2.07	5.77	17.0	51.7	157	454	—
Nitrosospira multiformis ATCC 25196	NC_007614 (Gene)		0.346	0.758	1.95	5.40	15.8	47.6	144	409	—
Nitrosospira multiformis ATCC 25196	NC_007614 (Intergenic)		0.386	0.864	2.24	6.27	18.3	52.5	133	—	—
Nocardia farcinica IFM10152	NC_006361		0.217	0.405	0.817	1.85	4.61	11.1	28.2	75.2	189
Nocardia farcinica IFM10152	NC_006361 (Gene)		0.206	0.380	0.752	1.69	4.18	9.95	25.2	66.8	167
Nocardia farcinica IFM10152	NC_006361 (Intergenic)		0.314	0.643	1.61	4.13	10.7	27.5	68.0	—	—
Nocardioides JS614	NC_008699		0.242	0.477	0.835	1.89	4.80	11.3	28.9	77.5	192
Nocardioides JS614	NC_008699 (Gene)		0.224	0.442	0.766	1.72	4.35	10.2	26.0	69.5	172
Nocardioides JS614	NC_008699 (Intergenic)		0.588	1.18	2.55	6.21	15.7	37.3	77.9	—	—
Nostoc sp	NC_003272		0.561	1.23	2.93	7.48	19.7	52.9	142	387	985
Nostoc sp	NC_003272 (Gene)		0.534	1.14	2.69	6.85	17.9	47.9	129	354	926
Nostoc sp	NC_003272 (Intergenic)		0.603	1.33	3.10	7.50	18.5	44.5	95.1	175	—
Novosphingobium aromaticivorans DSM 12444	NC_007794		0.193	0.411	1.01	2.58	6.91	18.6	51.7	146	—
Novosphingobium aromaticivorans DSM 12444	NC_007794 (Gene)		0.179	0.380	0.928	2.35	6.29	16.9	46.7	131	—
Novosphingobium aromaticivorans DSM 12444	NC_007794 (Intergenic)		0.404	0.891	2.23	5.79	15.3	39.7	90.2	—	—
Oceanobacillus iheyensis	NC_004193		0.762	1.62	3.71	9.04	23.6	63.7	175	465	—
Oceanobacillus iheyensis	NC_004193 (Gene)		0.696	1.47	3.33	8.07	20.8	55.7	153	406	—
Oceanobacillus iheyensis	NC_004193 (Intergenic)		0.903	1.60	3.46	8.24	20.6	49.8	108	—	—
Oenococcus oeni PSU-1	NC_008528		0.165	0.399	1.06	2.98	8.61	25.3	73.6	207	—
Oenococcus oeni PSU-1	NC_008528 (Gene)		0.157	0.380	1.02	2.87	8.30	24.5	71.6	201	—
Oenococcus oeni PSU-1	NC_008528 (Intergenic)		0.198	0.462	1.09	2.72	6.99	17.3	—	—	—
Onion yellows phytoplasma	NC_005303		0.100	0.230	0.570	1.47	3.83	10.0	25.6	—	—
Onion yellows phytoplasma	NC_005303 (Gene)		0.102	0.239	0.605	1.58	4.17	11.0	28.2	—	—
Onion yellows phytoplasma	NC_005303 (Intergenic)		0.094	0.202	0.464	1.12	2.71	6.53	—	—	—
Parachlamydia sp UWE25	NC_005861		0.162	0.414	1.12	3.10	8.82	25.3	72.8	201	—
Parachlamydia sp UWE25	NC_005861 (Gene)		0.163	0.425	1.17	3.27	9.34	26.9	77.1	209	—
Parachlamydia sp UWE25	NC_005861 (Intergenic)		0.147	0.336	0.818	2.06	5.39	14.2	36.4	—	—
Paracoccus denitrificans PD1222	NC_008686		0.213	0.388	0.872	2.11	5.32	13.3	34.7	92.2	—
Paracoccus denitrificans PD1222	NC_008686 (Gene)		0.200	0.359	0.798	1.93	4.84	12.0	31.2	82.7	—
Paracoccus denitrificans PD1222	NC_008686 (Intergenic)		0.366	0.724	1.67	4.03	9.69	21.6	—	—	—
Paracoccus denitrificans PD1222	NC_008687		0.209	0.384	0.864	2.09	5.28	13.2	34.4	90.6	—
Paracoccus denitrificans PD1222	NC_008687 (Gene)		0.193	0.353	0.787	1.90	4.77	11.8	30.8	80.7	—
Paracoccus denitrificans PD1222	NC_008687 (Intergenic)		0.400	0.784	1.82	4.49	11.1	26.5	—	—	—
Pasteurella multocida	NC_002663		0.281	0.722	1.93	5.20	14.4	41.5	118	313	—

next

Category	SN \ k	L_e (kb) 2	3	4	5	6	7	8	9	10
Pasteurella multocida	NC_002663 (Gene)	0.280	0.720	1.93	5.21	14.5	41.6	119	317	—
Pasteurella multocida	NC_002663 (Intergenic)	0.197	0.404	0.893	2.20	5.61	13.8	—	—	—
Pediococcus pentosaceus ATCC 25745	NC_008525	0.452	0.933	2.33	6.27	17.5	49.5	138	358	—
Pediococcus pentosaceus ATCC 25745	NC_008525 (Gene)	0.443	0.885	2.19	5.88	16.3	45.8	127	329	—
Pediococcus pentosaceus ATCC 25745	NC_008525 (Intergenic)	0.331	0.709	1.59	3.98	10.0	23.8	—	—	—
Pelobacter carbinolicus	NC_007498	0.437	0.754	1.86	4.98	14.2	42.0	125	361	—
Pelobacter carbinolicus	NC_007498 (Gene)	0.424	0.715	1.75	4.64	13.1	38.2	113	324	—
Pelobacter carbinolicus	NC_007498 (Intergenic)	0.344	0.715	1.78	4.91	14.0	38.6	89.8	—	—
Pelobacter propionicus DSM 2379	NC_008609	0.554	1.06	2.10	5.13	13.7	36.5	102	282	—
Pelobacter propionicus DSM 2379	NC_008609 (Gene)	0.533	0.987	1.91	4.64	12.3	32.3	89.3	246	—
Pelobacter propionicus DSM 2379	NC_008609 (Intergenic)	0.515	1.08	2.46	6.35	17.3	46.6	109	—	—
Pelodictyon luteolum DSM 273	NC_007512	0.427	0.777	1.74	4.48	12.5	35.4	102	284	—
Pelodictyon luteolum DSM 273	NC_007512 (Gene)	0.399	0.727	1.62	4.15	11.5	32.3	92.6	255	—
Pelodictyon luteolum DSM 273	NC_007512 (Intergenic)	0.461	0.903	2.09	5.36	14.6	38.8	—	—	—
Photobacterium profundum SS9	NC_006370	0.560	1.22	2.97	7.87	22.3	65.0	189	517	—
Photobacterium profundum SS9	NC_006370 (Gene)	0.493	1.05	2.57	6.84	19.4	56.8	166	456	—
Photobacterium profundum SS9	NC_006370 (Intergenic)	1.02	2.34	4.73	10.7	25.5	59.1	122	—	—
Photobacterium profundum SS9	NC_006371	0.558	1.28	3.11	8.21	23.1	66.1	182	433	—
Photobacterium profundum SS9	NC_006371 (Gene)	0.479	1.09	2.67	7.08	20.0	57.7	160	383	—
Photobacterium profundum SS9	NC_006371 (Intergenic)	1.09	2.48	5.31	12.4	29.2	62.3	111	—	—
Photorhabdus luminescens	NC_005126	0.549	1.21	2.88	7.50	20.9	60.7	174	457	962
Photorhabdus luminescens	NC_005126 (Gene)	0.496	1.07	2.58	6.77	19.0	56.3	169	485	1201
Photorhabdus luminescens	NC_005126 (Intergenic)	0.987	2.22	4.25	8.35	16.5	29.6	45.1	—	—
Pirellula sp	NC_005027	0.147	0.372	0.998	2.86	8.58	26.3	82.0	256	762
Pirellula sp	NC_005027 (Gene)	0.144	0.362	0.970	2.77	8.31	25.4	79.2	247	735
Pirellula sp	NC_005027 (Intergenic)	0.214	0.571	1.58	4.58	13.4	37.2	87.9	—	—
Polaromonas JS666	NC_007948	0.232	0.502	1.20	3.08	8.43	23.1	65.4	186	499
Polaromonas JS666	NC_007948 (Gene)	0.214	0.455	1.07	2.71	7.38	20.1	56.4	160	431
Polaromonas JS666	NC_007948 (Intergenic)	0.364	0.939	2.50	6.76	18.7	49.1	111	—	—
Porphyromonas gingivalis W83	NC_002950	0.578	0.997	2.39	6.46	18.6	54.3	156	407	—
Porphyromonas gingivalis W83	NC_002950 (Gene)	0.563	0.971	2.32	6.25	17.8	51.8	150	399	—
Porphyromonas gingivalis W83	NC_002950 (Intergenic)	0.387	0.708	1.65	4.35	11.7	28.7	58.0	—	—
Prochlorococcus marinus CCMP1375	NC_005042	0.241	0.660	1.81	5.04	14.1	40.1	115	309	—
Prochlorococcus marinus CCMP1375	NC_005042 (Gene)	0.238	0.650	1.77	4.93	13.7	38.9	111	296	—

next

Category	SN \ k	L_e (kb) 2	3	4	5	6	7	8	9	10
Prochlorococcus marinus CCMP1375	NC_005042 (Intergenic)	0.246	0.646	1.75	4.57	11.8	29.3	—	—	—
Prochlorococcus marinus MED4	NC_005072	0.219	0.555	1.44	3.79	9.83	26.1	70.4	181	—
Prochlorococcus marinus MED4	NC_005072 (Gene)	0.212	0.543	1.42	3.77	9.80	26.1	70.5	181	—
Prochlorococcus marinus MED4	NC_005072 (Intergenic)	0.241	0.545	1.28	2.97	6.88	16.0	—	—	—
Prochlorococcus marinus MIT9313	NC_005071	0.219	0.574	1.56	4.52	13.7	41.6	127	368	—
Prochlorococcus marinus MIT9313	NC_005071 (Gene)	0.217	0.557	1.49	4.28	12.9	38.9	118	335	—
Prochlorococcus marinus MIT9313	NC_005071 (Intergenic)	0.215	0.595	1.67	4.85	14.3	41.4	107	—	—
Prochlorococcus marinus MIT 9312	NC_007577	0.205	0.519	1.35	3.57	9.35	25.1	68.2	178	—
Prochlorococcus marinus MIT 9312	NC_007577 (Gene)	0.200	0.510	1.34	3.56	9.37	25.2	68.6	178	—
Prochlorococcus marinus MIT 9312	NC_007577 (Intergenic)	0.222	0.497	1.17	2.67	6.25	14.7	—	—	—
Prochlorococcus marinus NATL2A	NC_007335	0.227	0.605	1.65	4.52	12.5	35.4	101	270	—
Prochlorococcus marinus NATL2A	NC_007335 (Gene)	0.220	0.590	1.61	4.44	12.3	34.7	98.5	261	—
Prochlorococcus marinus NATL2A	NC_007335 (Intergenic)	0.241	0.589	1.50	3.76	9.67	24.6	58.4	—	—
Propionibacterium acnes KPA171202	NC_006085	0.700	1.40	2.76	6.75	18.3	50.2	141	385	—
Propionibacterium acnes KPA171202	NC_006085 (Gene)	0.659	1.30	2.54	6.21	16.7	45.8	128	348	—
Propionibacterium acnes KPA171202	NC_006085 (Intergenic)	1.12	2.63	5.77	14.7	37.1	83.6	—	—	—
Pseudoalteromonas atlantica T6c	NC_008228	0.435	1.04	2.73	7.46	21.7	65.9	202	595	1526
Pseudoalteromonas atlantica T6c	NC_008228 (Gene)	0.411	0.970	2.54	6.94	20.2	61.0	186	544	1381
Pseudoalteromonas atlantica T6c	NC_008228 (Intergenic)	0.538	1.34	3.22	8.31	22.6	61.5	152	—	—
Pseudoalteromonas haloplanktis TAC125	NC_007481	0.284	0.660	1.70	4.47	12.4	35.8	104	294	—
Pseudoalteromonas haloplanktis TAC125	NC_007481 (Gene)	0.276	0.636	1.64	4.32	12.0	34.6	100	283	—
Pseudoalteromonas haloplanktis TAC125	NC_007481 (Intergenic)	0.283	0.654	1.54	3.79	9.50	23.9	56.7	—	—
Pseudoalteromonas haloplanktis TAC125	NC_007482	0.259	0.609	1.56	4.10	11.3	31.6	85.2	—	—
Pseudoalteromonas haloplanktis TAC125	NC_007482 (Gene)	0.254	0.594	1.52	4.00	11.0	30.8	82.2	—	—
Pseudoalteromonas haloplanktis TAC125	NC_007482 (Intergenic)	0.239	0.552	1.33	3.24	7.86	17.8	—	—	—
Pseudomonas aeruginosa	NC_002516	0.329	0.465	0.883	2.06	5.23	12.9	34.5	93.7	241
Pseudomonas aeruginosa	NC_002516 (Gene)	0.302	0.418	0.779	1.81	4.55	11.2	29.6	80.1	205
Pseudomonas aeruginosa	NC_002516 (Intergenic)	0.554	1.00	2.23	5.60	14.8	39.4	98.7	—	—
Pseudomonas aeruginosa UCBPP-PA14	NC_008463	0.335	0.478	0.913	2.14	5.44	13.5	36.1	98.1	252
Pseudomonas aeruginosa UCBPP-PA14	NC_008463 (Gene)	0.305	0.427	0.801	1.87	4.71	11.6	30.8	83.4	214
Pseudomonas aeruginosa UCBPP-PA14	NC_008463 (Intergenic)	0.595	1.10	2.47	6.25	16.6	44.4	111	—	—
Pseudomonas entomophila L48	NC_008027	0.363	0.545	1.08	2.62	6.77	17.2	46.4	126	315
Pseudomonas entomophila L48	NC_008027 (Gene)	0.343	0.495	0.953	2.28	5.88	14.8	40.1	110	287
Pseudomonas entomophila L48	NC_008027 (Intergenic)	0.498	1.05	2.38	5.08	10.1	17.8	26.3	—	—

next

Category	SN \ k	L_e (kb) 2	3	4	5	6	7	8	9	10
Pseudomonas fluorescens Pf-5	NC_004129	0.381	0.570	1.15	2.76	7.09	18.1	49.2	135	351
Pseudomonas fluorescens Pf-5	NC_004129 (Gene)	0.368	0.520	1.02	2.42	6.16	15.6	42.1	115	301
Pseudomonas fluorescens Pf-5	NC_004129 (Intergenic)	0.399	0.943	2.34	6.02	15.6	38.0	77.5	—	—
Pseudomonas fluorescens PfO-1	NC_007492	0.267	0.516	1.20	3.03	8.23	22.6	64.0	183	500
Pseudomonas fluorescens PfO-1	NC_007492 (Gene)	0.248	0.467	1.06	2.67	7.20	19.6	55.2	157	429
Pseudomonas fluorescens PfO-1	NC_007492 (Intergenic)	0.380	0.949	2.45	6.63	18.6	50.6	121	—	—
Pseudomonas putida KT2440	NC_002947	0.323	0.556	1.24	3.16	8.58	23.2	65.8	187	499
Pseudomonas putida KT2440	NC_002947 (Gene)	0.301	0.498	1.08	2.73	7.37	19.8	55.8	158	428
Pseudomonas putida KT2440	NC_002947 (Intergenic)	0.478	1.13	2.98	7.81	19.9	46.3	89.4	—	—
Pseudomonas syringae phaseolicola 1448A	NC_005773	0.283	0.583	1.46	3.88	10.9	31.2	91.3	264	699
Pseudomonas syringae phaseolicola 1448A	NC_005773 (Gene)	0.263	0.526	1.29	3.40	9.48	26.9	78.4	227	608
Pseudomonas syringae phaseolicola 1448A	NC_005773 (Intergenic)	0.456	1.12	2.95	7.93	21.1	52.4	107	—	—
Pseudomonas syringae pv B728a	NC_007005	0.283	0.567	1.38	3.64	10.1	28.6	83.0	240	652
Pseudomonas syringae pv B728a	NC_007005 (Gene)	0.264	0.512	1.22	3.18	8.80	24.6	71.1	206	567
Pseudomonas syringae pv B728a	NC_007005 (Intergenic)	0.458	1.12	2.93	7.88	20.7	50.5	101	—	—
Pseudomonas syringae tomato DC3000	NC_004578	0.267	0.562	1.41	3.72	10.4	29.7	86.7	251	676
Pseudomonas syringae tomato DC3000	NC_004578 (Gene)	0.247	0.504	1.24	3.24	9.00	25.4	73.7	213	575
Pseudomonas syringae tomato DC3000	NC_004578 (Intergenic)	0.443	1.08	2.86	7.92	22.1	58.7	133	—	—
Psychrobacter arcticum 273-4	NC_007204	0.459	0.839	1.95	5.17	14.5	41.5	114	272	—
Psychrobacter arcticum 273-4	NC_007204 (Gene)	0.379	0.701	1.66	4.47	12.7	37.4	109	297	—
Psychrobacter arcticum 273-4	NC_007204 (Intergenic)	0.928	1.51	2.67	5.71	11.5	20.2	28.6	—	—
Psychrobacter cryohalolentis K5	NC_007969	0.483	0.874	2.00	5.32	15.1	44.2	131	375	—
Psychrobacter cryohalolentis K5	NC_007969 (Gene)	0.400	0.734	1.73	4.63	13.2	38.8	115	330	—
Psychrobacter cryohalolentis K5	NC_007969 (Intergenic)	1.17	1.77	2.83	6.33	15.6	38.4	89.6	—	—
Ralstonia eutropha H16	NC_008313	0.175	0.352	0.768	1.89	4.98	12.7	34.0	92.9	—
Ralstonia eutropha H16	NC_008313 (Gene)	0.159	0.314	0.675	1.65	4.32	10.9	29.0	78.7	—
Ralstonia eutropha H16	NC_008313 (Intergenic)	0.376	0.816	1.93	4.90	13.1	34.8	87.3	—	—
Ralstonia eutropha H16	NC_008314	0.168	0.347	0.774	1.92	5.09	13.2	35.5	97.4	—
Ralstonia eutropha H16	NC_008314 (Gene)	0.152	0.311	0.684	1.69	4.45	11.4	30.6	83.4	—
Ralstonia eutropha H16	NC_008314 (Intergenic)	0.400	0.903	2.12	5.40	14.2	37.0	87.1	—	—
Ralstonia eutropha JMP134	NC_007347	0.158	0.360	0.867	2.24	6.17	16.8	47.0	134	—
Ralstonia eutropha JMP134	NC_007347 (Gene)	0.144	0.325	0.772	1.99	5.44	14.7	40.9	116	—
Ralstonia eutropha JMP134	NC_007347 (Intergenic)	0.370	0.857	2.12	5.54	15.1	40.7	99.9	—	—
Ralstonia eutropha JMP134	NC_007348	0.156	0.362	0.891	2.32	6.42	17.6	49.5	140	—

next

Category	SN \ k	L_e (kb) 2	3	4	5	6	7	8	9	10
Ralstonia eutropha JMP134	NC_007348 (Gene)	0.141	0.324	0.791	2.05	5.64	15.4	42.9	121	—
Ralstonia eutropha JMP134	NC_007348 (Intergenic)	0.394	0.944	2.36	6.25	17.0	44.7	103	—	—
Ralstonia metallidurans CH34	NC_007973	0.192	0.430	1.04	2.69	7.39	20.1	56.6	161	—
Ralstonia metallidurans CH34	NC_007973 (Gene)	0.174	0.385	0.916	2.36	6.46	17.4	48.9	139	—
Ralstonia metallidurans CH34	NC_007973 (Intergenic)	0.462	1.16	2.94	7.82	21.3	56.2	129	—	—
Ralstonia metallidurans CH34	NC_007974	0.202	0.461	1.13	2.96	8.20	22.6	63.9	180	—
Ralstonia metallidurans CH34	NC_007974 (Gene)	0.182	0.410	0.998	2.59	7.14	19.5	54.9	154	—
Ralstonia metallidurans CH34	NC_007974 (Intergenic)	0.506	1.29	3.33	8.94	24.2	60.7	126	—	—
Ralstonia solanacearum	NC_003295	0.154	0.342	0.774	1.93	5.13	13.2	35.4	97.1	—
Ralstonia solanacearum	NC_003295 (Gene)	0.141	0.309	0.686	1.70	4.50	11.4	30.5	83.4	—
Ralstonia solanacearum	NC_003295 (Intergenic)	0.299	0.699	1.74	4.53	12.0	31.8	78.8	—	—
Rhizobium etli CFN 42	NC_007761	0.151	0.341	0.821	2.15	6.01	16.9	48.5	141	388
Rhizobium etli CFN 42	NC_007761 (Gene)	0.137	0.306	0.726	1.88	5.23	14.5	41.4	119	—
Rhizobium etli CFN 42	NC_007761 (Intergenic)	0.267	0.674	1.78	4.98	14.5	41.8	111	—	—
Rhizobium leguminosarum bv viciae 3841	NC_008380	0.154	0.346	0.834	2.18	6.10	17.1	49.2	143	396
Rhizobium leguminosarum bv viciae 3841	NC_008380 (Gene)	0.140	0.310	0.735	1.90	5.29	14.7	41.8	121	333
Rhizobium leguminosarum bv viciae 3841	NC_008380 (Intergenic)	0.263	0.663	1.78	5.01	14.5	41.0	105	—	—
Rhodobacter sphaeroides 2 4 1	NC_007493	0.187	0.405	0.899	2.15	5.42	13.4	34.7	92.0	—
Rhodobacter sphaeroides 2 4 1	NC_007493 (Gene)	0.167	0.359	0.787	1.87	4.71	11.6	29.8	78.8	—
Rhodobacter sphaeroides 2 4 1	NC_007493 (Intergenic)	0.456	0.850	1.94	4.64	11.4	28.2	65.6	—	—
Rhodobacter sphaeroides 2 4 1	NC_007494	0.192	0.413	0.923	2.20	5.56	13.7	34.9	—	—
Rhodobacter sphaeroides 2 4 1	NC_007494 (Gene)	0.172	0.368	0.811	1.92	4.82	11.8	29.9	—	—
Rhodobacter sphaeroides 2 4 1	NC_007494 (Intergenic)	0.455	0.831	1.91	4.55	11.0	25.1	—	—	—
Rhodococcus RHA1	NC_008268	0.173	0.425	0.959	2.36	6.39	16.6	45.2	129	348
Rhodococcus RHA1	NC_008268 (Gene)	0.162	0.398	0.888	2.17	5.83	15.1	40.8	116	311
Rhodococcus RHA1	NC_008268 (Intergenic)	0.384	0.893	2.32	6.37	18.0	49.3	126	—	—
Rhodoferax ferrireducens T118	NC_007908	0.200	0.466	1.18	3.16	8.90	25.3	73.4	213	582
Rhodoferax ferrireducens T118	NC_007908 (Gene)	0.190	0.436	1.09	2.89	8.09	22.8	65.8	190	519
Rhodoferax ferrireducens T118	NC_007908 (Intergenic)	0.308	0.808	2.25	6.45	18.7	51.0	117	—	—
Rhodopseudomonas palustris BisA53	NC_008435	0.123	0.282	0.668	1.68	4.56	12.2	33.5	94.5	253
Rhodopseudomonas palustris BisA53	NC_008435 (Gene)	0.113	0.252	0.585	1.46	3.91	10.3	28.1	78.8	210
Rhodopseudomonas palustris BisA53	NC_008435 (Intergenic)	0.231	0.599	1.61	4.41	12.0	31.8	76.0	—	—
Rhodopseudomonas palustris BisB18	NC_007925	0.119	0.269	0.629	1.56	4.18	11.1	30.2	84.6	224
Rhodopseudomonas palustris BisB18	NC_007925 (Gene)	0.110	0.243	0.555	1.36	3.62	9.45	25.6	71.3	189

next

Category	SN \ k	L_e (kb) 2	3	4	5	6	7	8	9	10
Rhodopseudomonas palustris BisB18	NC_007925 (Intergenic)	0.201	0.518	1.38	3.66	9.59	24.6	57.9	—	—
Rhodopseudomonas palustris BisB5	NC_007958	0.104	0.247	0.597	1.51	4.14	11.1	30.7	87.0	234
Rhodopseudomonas palustris BisB5	NC_007958 (Gene)	0.095	0.223	0.530	1.33	3.60	9.56	26.2	73.6	197
Rhodopseudomonas palustris BisB5	NC_007958 (Intergenic)	0.193	0.502	1.37	3.79	10.5	28.5	70.5	—	—
Rhodopseudomonas palustris CGA009	NC_005296	0.120	0.274	0.640	1.63	4.45	11.9	33.1	94.1	254
Rhodopseudomonas palustris CGA009	NC_005296 (Gene)	0.111	0.248	0.568	1.43	3.88	10.3	28.3	80.0	215
Rhodopseudomonas palustris CGA009	NC_005296 (Intergenic)	0.221	0.575	1.54	4.25	11.8	31.7	78.8	—	—
Rhodopseudomonas palustris HaA2	NC_007778	0.100	0.235	0.549	1.37	3.68	9.67	26.2	73.1	193
Rhodopseudomonas palustris HaA2	NC_007778 (Gene)	0.092	0.212	0.486	1.20	3.20	8.31	22.3	62.0	163
Rhodopseudomonas palustris HaA2	NC_007778 (Intergenic)	0.191	0.501	1.33	3.55	9.41	24.2	55.9	—	—
Rhodospirillum rubrum ATCC 11170	NC_007643	0.344	0.480	0.979	2.25	5.56	13.9	36.3	96.5	246
Rhodospirillum rubrum ATCC 11170	NC_007643 (Gene)	0.307	0.415	0.844	1.94	4.77	11.8	30.8	81.5	—
Rhodospirillum rubrum ATCC 11170	NC_007643 (Intergenic)	0.331	0.744	1.72	4.36	11.5	30.3	75.1	—	—
Rickettsia bellii RML369-C	NC_007940	0.709	1.36	3.02	7.16	17.5	43.4	107	238	—
Rickettsia bellii RML369-C	NC_007940 (Gene)	0.616	1.19	2.67	6.40	15.8	39.8	101	236	—
Rickettsia bellii RML369-C	NC_007940 (Intergenic)	1.49	2.39	3.57	6.46	11.6	19.3	—	—	—
Rickettsia conorii	NC_003103	1.09	1.66	3.56	8.21	19.9	49.6	121	268	—
Rickettsia conorii	NC_003103 (Gene)	1.04	1.49	3.17	7.34	17.8	44.8	112	—	—
Rickettsia conorii	NC_003103 (Intergenic)	1.18	2.25	4.37	8.78	17.4	31.6	—	—	—
Rickettsia felis URRWXCal2	NC_007109	0.955	1.52	3.30	7.65	18.4	44.9	106	220	—
Rickettsia felis URRWXCal2	NC_007109 (Gene)	0.932	1.40	3.01	7.03	17.0	42.7	107	249	—
Rickettsia felis URRWXCal2	NC_007109 (Intergenic)	0.808	1.66	2.82	5.00	8.84	14.0	—	—	—
Rickettsia prowazekii	NC_000963	1.39	2.42	4.82	10.6	25.0	60.2	145	318	—
Rickettsia prowazekii	NC_000963 (Gene)	1.10	1.90	3.84	8.63	20.3	48.9	118	—	—
Rickettsia prowazekii	NC_000963 (Intergenic)	2.84	4.70	8.18	15.7	31.1	61.0	—	—	—
Rickettsia typhi wilmington	NC_006142	1.36	2.45	4.84	10.6	24.9	60.0	145	318	—
Rickettsia typhi wilmington	NC_006142 (Gene)	1.10	1.94	3.89	8.71	20.4	49.1	118	—	—
Rickettsia typhi wilmington	NC_006142 (Intergenic)	2.23	4.05	7.19	14.3	29.2	58.0	—	—	—
Roseobacter denitrificans OCh 114	NC_008209	0.214	0.497	1.31	3.47	9.59	27.6	81.2	238	—
Roseobacter denitrificans OCh 114	NC_008209 (Gene)	0.203	0.472	1.24	3.25	8.94	25.5	74.7	218	—
Roseobacter denitrificans OCh 114	NC_008209 (Intergenic)	0.300	0.710	1.91	5.31	15.0	41.5	102	—	—
Rubrobacter xylanophilus DSM 9941	NC_008148	0.669	1.20	1.67	3.56	8.19	18.1	44.8	114	—
Rubrobacter xylanophilus DSM 9941	NC_008148 (Gene)	0.673	1.20	1.60	3.38	7.74	16.9	41.5	106	—
Rubrobacter xylanophilus DSM 9941	NC_008148 (Intergenic)	0.482	0.799	1.68	3.92	9.38	22.5	—	—	—

next

Category	SN \ k	L_e (kb) 2	3	4	5	6	7	8	9	10
Saccharophagus degradans 2-40	NC_007912	0.334	0.740	1.92	5.24	15.2	46.0	140	415	1099
Saccharophagus degradans 2-40	NC_007912 (Gene)	0.330	0.709	1.82	4.96	14.4	43.2	131	387	1024
Saccharophagus degradans 2-40	NC_007912 (Intergenic)	0.309	0.771	1.98	5.28	14.5	39.3	95.7	—	—
Salinibacter ruber DSM 13855	NC_007677	0.497	1.18	2.22	5.33	13.5	33.2	87.5	233	—
Salinibacter ruber DSM 13855	NC_007677 (Gene)	0.450	1.05	1.89	4.51	11.4	27.5	71.9	191	—
Salinibacter ruber DSM 13855	NC_007677 (Intergenic)	0.605	1.42	3.39	8.58	22.5	58.7	140	—	—
Salmonella enterica Choleraesuis	NC_006905	0.413	0.729	1.67	4.22	11.6	33.8	101	298	817
Salmonella enterica Choleraesuis	NC_006905 (Gene)	0.371	0.648	1.50	3.79	10.4	30.1	89.7	264	722
Salmonella enterica Choleraesuis	NC_006905 (Intergenic)	0.820	1.52	2.86	6.73	17.3	44.9	106	—	—
Salmonella enterica Paratypi ATCC 9150	NC_006511	0.408	0.721	1.65	4.18	11.5	33.4	99.7	294	804
Salmonella enterica Paratypi ATCC 9150	NC_006511 (Gene)	0.365	0.637	1.47	3.73	10.2	29.6	88.0	259	—
Salmonella enterica Paratypi ATCC 9150	NC_006511 (Intergenic)	0.778	1.47	2.77	6.54	16.9	43.8	104	—	—
Salmonella typhi	NC_003198	0.423	0.743	1.70	4.31	11.8	34.6	103	305	834
Salmonella typhi	NC_003198 (Gene)	0.380	0.659	1.52	3.86	10.6	30.8	91.8	270	740
Salmonella typhi	NC_003198 (Intergenic)	0.780	1.47	2.75	6.47	16.6	42.9	101	—	—
Salmonella typhi Ty2	NC_004631	0.422	0.742	1.70	4.31	11.8	34.6	103	305	832
Salmonella typhi Ty2	NC_004631 (Gene)	0.378	0.656	1.52	3.85	10.6	30.6	91.2	269	734
Salmonella typhi Ty2	NC_004631 (Intergenic)	0.767	1.44	2.73	6.48	16.7	43.5	103	—	—
Salmonella typhimurium LT2	NC_003197	0.410	0.722	1.65	4.17	11.4	33.3	99.6	295	811
Salmonella typhimurium LT2	NC_003197 (Gene)	0.368	0.638	1.47	3.73	10.2	29.6	88.0	259	715
Salmonella typhimurium LT2	NC_003197 (Intergenic)	0.774	1.46	2.76	6.51	16.8	43.7	104	—	—
Shewanella amazonensis SB2B	NC_008700	0.325	0.682	1.69	4.57	13.0	38.0	113	328	875
Shewanella amazonensis SB2B	NC_008700 (Gene)	0.317	0.643	1.58	4.22	11.9	34.4	101	293	—
Shewanella amazonensis SB2B	NC_008700 (Intergenic)	0.296	0.760	1.90	5.12	14.2	38.8	95.4	—	—
Shewanella ANA-3	NC_008577	0.522	1.07	2.55	6.55	18.3	53.7	161	468	1215
Shewanella ANA-3	NC_008577 (Gene)	0.511	1.00	2.38	6.07	16.8	49.0	145	419	1077
Shewanella ANA-3	NC_008577 (Intergenic)	0.444	1.07	2.46	6.32	17.2	46.6	117	—	—
Shewanella denitrificans OS217	NC_007954	0.389	0.906	2.31	6.17	17.6	52.0	156	452	1159
Shewanella denitrificans OS217	NC_007954 (Gene)	0.363	0.833	2.12	5.64	16.0	47.2	141	407	—
Shewanella denitrificans OS217	NC_007954 (Intergenic)	0.492	1.17	2.77	7.03	18.9	49.8	120	—	—
Shewanella frigidimarina NCIMB 400	NC_008345	0.443	0.981	2.44	6.36	17.9	52.3	155	449	1155
Shewanella frigidimarina NCIMB 400	NC_008345 (Gene)	0.398	0.881	2.21	5.78	16.3	47.5	141	404	—
Shewanella frigidimarina NCIMB 400	NC_008345 (Intergenic)	0.834	1.59	3.26	7.48	19.0	48.8	119	—	—
Shewanella MR-4	NC_008321	0.529	1.07	2.55	6.56	18.3	53.6	160	464	1194

next

Category	SN \ k	L_e (kb) 2	3	4	5	6	7	8	9	10
Shewanella MR-4	NC_008321 (Gene)	0.516	1.01	2.38	6.05	16.8	48.7	144	414	—
Shewanella MR-4	NC_008321 (Intergenic)	0.445	1.07	2.45	6.23	16.9	45.7	115	—	—
Shewanella MR-7	NC_008322	0.528	1.08	2.57	6.63	18.5	54.4	163	473	1220
Shewanella MR-7	NC_008322 (Gene)	0.515	1.01	2.39	6.11	17.0	49.5	147	422	—
Shewanella MR-7	NC_008322 (Intergenic)	0.443	1.06	2.46	6.30	17.1	46.4	117	—	—
Shewanella oneidensis	NC_004347	0.463	1.00	2.49	6.60	18.8	55.8	167	474	1141
Shewanella oneidensis	NC_004347 (Gene)	0.444	0.930	2.30	6.07	17.2	50.9	152	429	1032
Shewanella oneidensis	NC_004347 (Intergenic)	0.480	1.16	2.77	7.13	19.1	49.7	114	—	—
Shigella boydii Sb227	NC_007613	0.387	0.741	1.76	4.60	12.8	37.1	106	275	560
Shigella boydii Sb227	NC_007613 (Gene)	0.353	0.661	1.58	4.11	11.4	32.9	94.4	246	—
Shigella boydii Sb227	NC_007613 (Intergenic)	0.577	1.25	2.70	6.62	16.9	40.1	77.4	—	—
Shigella dysenteriae	NC_007606	0.387	0.745	1.78	4.61	12.7	35.4	91.8	193	302
Shigella dysenteriae	NC_007606 (Gene)	0.357	0.668	1.60	4.14	11.4	32.1	86.3	195	—
Shigella dysenteriae	NC_007606 (Intergenic)	0.534	1.16	2.51	5.80	12.6	22.7	31.4	—	—
Shigella flexneri 2a	NC_004337	0.391	0.754	1.80	4.69	13.1	38.3	113	310	710
Shigella flexneri 2a	NC_004337 (Gene)	0.365	0.687	1.64	4.27	11.9	34.6	102	280	—
Shigella flexneri 2a	NC_004337 (Intergenic)	0.563	1.25	2.71	6.71	17.5	44.6	98.0	—	—
Shigella flexneri 2a 2457T	NC_004741	0.392	0.753	1.80	4.69	13.1	38.3	113	312	720
Shigella flexneri 2a 2457T	NC_004741 (Gene)	0.357	0.670	1.60	4.17	11.6	33.7	99.0	274	—
Shigella flexneri 2a 2457T	NC_004741 (Intergenic)	0.576	1.26	2.77	6.93	18.2	47.3	106	—	—
Shigella flexneri 5 8401	NC_008258	0.388	0.749	1.79	4.66	13.0	38.0	112	310	718
Shigella flexneri 5 8401	NC_008258 (Gene)	0.355	0.669	1.60	4.16	11.6	33.7	98.9	274	—
Shigella flexneri 5 8401	NC_008258 (Intergenic)	0.580	1.29	2.80	6.96	18.2	46.1	99.8	—	—
Shigella sonnei Ss046	NC_007384	0.393	0.754	1.79	4.66	13.0	37.6	108	282	582
Shigella sonnei Ss046	NC_007384 (Gene)	0.357	0.669	1.59	4.13	11.5	33.2	95.9	254	—
Shigella sonnei Ss046	NC_007384 (Intergenic)	0.585	1.28	2.79	6.85	17.1	38.7	69.0	—	—
Silicibacter pomeroyi DSS-3	NC_003911	0.284	0.497	1.11	2.69	6.74	17.2	45.9	124	—
Silicibacter pomeroyi DSS-3	NC_003911 (Gene)	0.262	0.450	0.995	2.39	5.97	15.1	40.3	109	—
Silicibacter pomeroyi DSS-3	NC_003911 (Intergenic)	0.424	0.968	2.40	6.18	15.9	41.1	95.8	—	—
Silicibacter TM1040	NC_008044	0.253	0.584	1.49	3.91	10.8	30.5	88.1	251	—
Silicibacter TM1040	NC_008044 (Gene)	0.236	0.541	1.37	3.58	9.80	27.5	79.0	224	—
Silicibacter TM1040	NC_008044 (Intergenic)	0.364	0.832	2.14	5.74	15.6	42.0	100	—	—
Sinorhizobium meliloti	NC_003047	0.153	0.359	0.893	2.35	6.59	18.5	52.9	152	—
Sinorhizobium meliloti	NC_003047 (Gene)	0.135	0.314	0.773	2.02	5.63	15.6	44.5	128	—

next

Category	SN \ k	L_e (kb) 2	3	4	5	6	7	8	9	10
Sinorhizobium meliloti	NC_003047 (Intergenic)	0.312	0.747	1.95	5.16	13.6	33.4	70.3	—	—
Sodalis glossinidius morsitans	NC_007712	0.433	0.828	1.84	4.62	12.8	36.8	108	309	—
Sodalis glossinidius morsitans	NC_007712 (Gene)	0.349	0.651	1.47	3.72	10.2	29.0	83.5	233	—
Sodalis glossinidius morsitans	NC_007712 (Intergenic)	0.547	1.08	2.33	5.79	15.9	45.9	131	339	—
Solibacter usitatus Ellin6076	NC_008536	0.287	0.607	1.47	3.83	10.7	30.0	86.8	255	711
Solibacter usitatus Ellin6076	NC_008536 (Gene)	0.265	0.557	1.34	3.47	9.63	26.9	77.8	228	644
Solibacter usitatus Ellin6076	NC_008536 (Intergenic)	0.659	1.53	3.82	9.86	24.5	55.5	104	—	—
Sphingopyxis alaskensis RB2256	NC_008048	0.102	0.236	0.589	1.46	3.87	10.3	28.1	77.9	—
Sphingopyxis alaskensis RB2256	NC_008048 (Gene)	0.093	0.215	0.534	1.32	3.49	9.29	25.1	69.6	—
Sphingopyxis alaskensis RB2256	NC_008048 (Intergenic)	0.315	0.706	1.67	4.12	10.2	24.8	52.8	—	—
Staphylococcus aureus aureus MRSA252	NC_002952	0.583	1.51	3.50	8.59	21.8	56.5	149	376	—
Staphylococcus aureus aureus MRSA252	NC_002952 (Gene)	0.519	1.32	3.03	7.37	18.6	48.1	126	321	—
Staphylococcus aureus aureus MRSA252	NC_002952 (Intergenic)	1.20	2.27	4.85	11.0	25.2	54.5	108	—	—
Staphylococcus aureus aureus MSSA476	NC_002953	0.582	1.50	3.47	8.48	21.5	55.6	146	370	—
Staphylococcus aureus aureus MSSA476	NC_002953 (Gene)	0.516	1.31	2.98	7.23	18.3	47.0	123	313	—
Staphylococcus aureus aureus MSSA476	NC_002953 (Intergenic)	1.20	2.23	4.83	10.9	24.9	53.8	108	—	—
Staphylococcus aureus COL	NC_002951	0.583	1.50	3.47	8.47	21.5	55.5	145	368	—
Staphylococcus aureus COL	NC_002951 (Gene)	0.512	1.30	2.96	7.17	18.1	46.4	122	308	—
Staphylococcus aureus COL	NC_002951 (Intergenic)	1.25	2.32	4.97	11.3	25.9	56.1	112	—	—
Staphylococcus aureus Mu50	NC_002758	0.590	1.53	3.53	8.63	21.9	56.7	149	378	—
Staphylococcus aureus Mu50	NC_002758 (Gene)	0.517	1.31	3.00	7.29	18.4	47.5	125	317	—
Staphylococcus aureus Mu50	NC_002758 (Intergenic)	1.23	2.37	5.13	11.6	26.5	57.5	115	—	—
Staphylococcus aureus MW2	NC_003923	0.580	1.50	3.47	8.48	21.5	55.6	146	370	—
Staphylococcus aureus MW2	NC_003923 (Gene)	0.510	1.30	2.95	7.15	18.0	46.4	122	310	—
Staphylococcus aureus MW2	NC_003923 (Intergenic)	1.21	2.28	4.96	11.2	25.6	55.5	111	—	—
Staphylococcus aureus N315	NC_002745	0.585	1.51	3.50	8.55	21.7	56.1	147	373	—
Staphylococcus aureus N315	NC_002745 (Gene)	0.512	1.30	2.97	7.20	18.2	46.8	123	311	—
Staphylococcus aureus N315	NC_002745 (Intergenic)	1.22	2.38	5.17	11.6	26.6	57.5	114	—	—
Staphylococcus aureus NCTC 8325	NC_007795	0.581	1.51	3.48	8.51	21.6	55.9	147	372	—
Staphylococcus aureus NCTC 8325	NC_007795 (Gene)	0.511	1.31	2.98	7.24	18.3	47.2	124	315	—
Staphylococcus aureus NCTC 8325	NC_007795 (Intergenic)	1.28	2.36	5.04	11.4	26.0	56.3	112	—	—
Staphylococcus aureus RF122	NC_007622	0.598	1.54	3.54	8.65	21.9	56.6	149	377	—
Staphylococcus aureus RF122	NC_007622 (Gene)	0.525	1.33	3.02	7.32	18.5	47.6	125	317	—
Staphylococcus aureus RF122	NC_007622 (Intergenic)	1.27	2.36	5.08	11.5	26.3	57.0	115	—	—

next

		L_e (kb)								
Category	SN $\backslash\ k$	2	3	4	5	6	7	8	9	10
Staphylococcus aureus USA300	NC_007793	0.586	1.51	3.49	8.53	21.6	55.9	147	372	—
Staphylococcus aureus USA300	NC_007793 (Gene)	0.510	1.30	2.95	7.14	18.0	46.3	122	309	—
Staphylococcus aureus USA300	NC_007793 (Intergenic)	1.22	2.35	5.08	11.6	26.8	58.6	119	—	—
Staphylococcus epidermidis ATCC 12228	NC_004461	0.805	2.00	4.34	10.4	26.0	65.5	168	407	—
Staphylococcus epidermidis ATCC 12228	NC_004461 (Gene)	0.696	1.73	3.73	8.84	22.0	55.4	142	345	—
Staphylococcus epidermidis ATCC 12228	NC_004461 (Intergenic)	1.57	2.84	5.63	12.3	27.1	56.4	107	—	—
Staphylococcus epidermidis RP62A	NC_002976	0.803	2.02	4.39	10.5	26.3	66.5	171	419	—
Staphylococcus epidermidis RP62A	NC_002976 (Gene)	0.699	1.75	3.77	8.95	22.3	56.2	145	354	—
Staphylococcus epidermidis RP62A	NC_002976 (Intergenic)	1.65	2.88	5.69	12.2	26.9	55.8	107	—	—
Staphylococcus haemolyticus	NC_007168	0.906	2.16	4.51	10.6	26.0	64.8	163	379	—
Staphylococcus haemolyticus	NC_007168 (Gene)	0.792	1.85	3.79	8.79	21.5	53.4	134	315	—
Staphylococcus haemolyticus	NC_007168 (Intergenic)	1.44	2.82	6.00	13.4	28.4	56.3	100	—	—
Staphylococcus saprophyticus	NC_007350	0.694	1.71	3.91	9.52	24.2	62.3	162	403	—
Staphylococcus saprophyticus	NC_007350 (Gene)	0.590	1.43	3.24	7.84	19.8	51.0	133	332	—
Staphylococcus saprophyticus	NC_007350 (Intergenic)	1.86	2.70	5.34	11.6	25.4	54.9	109	—	—
Streptococcus agalactiae 2603	NC_004116	0.480	1.16	2.68	6.96	19.0	52.0	143	367	—
Streptococcus agalactiae 2603	NC_004116 (Gene)	0.464	1.12	2.63	6.83	18.5	50.2	137	348	—
Streptococcus agalactiae 2603	NC_004116 (Intergenic)	0.368	0.705	1.51	3.70	9.21	22.2	—	—	—
Streptococcus agalactiae A909	NC_007432	0.483	1.16	2.69	6.98	19.0	52.0	142	365	—
Streptococcus agalactiae A909	NC_007432 (Gene)	0.471	1.14	2.66	6.88	18.6	50.4	137	346	—
Streptococcus agalactiae A909	NC_007432 (Intergenic)	0.359	0.698	1.49	3.66	9.16	22.3	—	—	—
Streptococcus agalactiae NEM316	NC_004368	0.462	1.12	2.61	6.78	18.5	50.7	140	361	—
Streptococcus agalactiae NEM316	NC_004368 (Gene)	0.452	1.11	2.59	6.71	18.2	49.6	136	348	—
Streptococcus agalactiae NEM316	NC_004368 (Intergenic)	0.432	0.757	1.52	3.64	8.94	21.3	—	—	—
Streptococcus mutans	NC_004350	0.251	0.632	1.59	4.35	12.4	35.3	101	278	—
Streptococcus mutans	NC_004350 (Gene)	0.246	0.619	1.57	4.29	12.1	34.4	98.1	266	—
Streptococcus mutans	NC_004350 (Intergenic)	0.211	0.478	1.12	2.93	7.81	20.2	—	—	—
Streptococcus pneumoniae D39	NC_008533	0.315	0.753	1.89	5.15	14.7	42.5	122	324	—
Streptococcus pneumoniae D39	NC_008533 (Gene)	0.309	0.746	1.86	5.08	14.4	41.4	119	319	—
Streptococcus pneumoniae D39	NC_008533 (Intergenic)	0.255	0.486	1.15	2.98	7.76	19.1	—	—	—
Streptococcus pneumoniae R6	NC_003098	0.314	0.752	1.88	5.14	14.7	42.4	121	324	—
Streptococcus pneumoniae R6	NC_003098 (Gene)	0.310	0.748	1.87	5.09	14.5	41.6	119	321	—
Streptococcus pneumoniae R6	NC_003098 (Intergenic)	0.250	0.480	1.15	2.98	7.68	18.5	—	—	—
Streptococcus pneumoniae TIGR4	NC_003028	0.317	0.759	1.90	5.21	14.8	42.5	120	309	—

next

Category	SN \ k	L_e (kb) 2	3	4	5	6	7	8	9	10
Streptococcus pneumoniae TIGR4	NC_003028 (Gene)	0.312	0.757	1.89	5.16	14.6	41.7	117	305	—
Streptococcus pneumoniae TIGR4	NC_003028 (Intergenic)	0.249	0.461	1.10	2.82	7.13	16.6	—	—	—
Streptococcus pyogenes M1 GAS	NC_002737	0.332	0.782	1.94	5.27	15.1	43.4	124	334	—
Streptococcus pyogenes M1 GAS	NC_002737 (Gene)	0.323	0.765	1.93	5.25	14.9	42.8	122	321	—
Streptococcus pyogenes M1 GAS	NC_002737 (Intergenic)	0.283	0.569	1.25	3.17	8.36	21.8	—	—	—
Streptococcus pyogenes MGAS10270	NC_008022	0.339	0.796	1.97	5.35	15.3	44.1	127	341	—
Streptococcus pyogenes MGAS10270	NC_008022 (Gene)	0.329	0.777	1.95	5.33	15.2	43.6	124	330	—
Streptococcus pyogenes MGAS10270	NC_008022 (Intergenic)	0.284	0.568	1.23	3.07	8.01	20.7	—	—	—
Streptococcus pyogenes MGAS10394	NC_006086	0.340	0.801	1.99	5.41	15.5	44.7	128	343	—
Streptococcus pyogenes MGAS10394	NC_006086 (Gene)	0.331	0.786	1.98	5.39	15.4	44.2	126	332	—
Streptococcus pyogenes MGAS10394	NC_006086 (Intergenic)	0.283	0.555	1.21	3.06	8.03	20.8	—	—	—
Streptococcus pyogenes MGAS10750	NC_008024	0.336	0.792	1.97	5.35	15.3	44.0	126	338	—
Streptococcus pyogenes MGAS10750	NC_008024 (Gene)	0.326	0.775	1.95	5.32	15.2	43.4	123	327	—
Streptococcus pyogenes MGAS10750	NC_008024 (Intergenic)	0.282	0.568	1.24	3.12	8.20	21.2	—	—	—
Streptococcus pyogenes MGAS2096	NC_008023	0.339	0.799	1.98	5.39	15.4	44.5	127	341	—
Streptococcus pyogenes MGAS2096	NC_008023 (Gene)	0.330	0.785	1.97	5.38	15.4	44.1	125	330	—
Streptococcus pyogenes MGAS2096	NC_008023 (Intergenic)	0.286	0.568	1.24	3.12	8.17	21.1	—	—	—
Streptococcus pyogenes MGAS315	NC_004070	0.339	0.793	1.96	5.33	15.2	44.0	126	338	—
Streptococcus pyogenes MGAS315	NC_004070 (Gene)	0.329	0.775	1.95	5.31	15.2	43.5	123	325	—
Streptococcus pyogenes MGAS315	NC_004070 (Intergenic)	0.289	0.570	1.23	3.09	8.11	21.1	—	—	—
Streptococcus pyogenes MGAS5005	NC_007297	0.334	0.787	1.95	5.30	15.1	43.6	125	334	—
Streptococcus pyogenes MGAS5005	NC_007297 (Gene)	0.324	0.768	1.93	5.26	15.0	43.0	122	321	—
Streptococcus pyogenes MGAS5005	NC_007297 (Intergenic)	0.277	0.552	1.20	3.03	7.92	20.5	—	—	—
Streptococcus pyogenes MGAS6180	NC_007296	0.336	0.789	1.95	5.30	15.1	43.6	125	335	—
Streptococcus pyogenes MGAS6180	NC_007296 (Gene)	0.327	0.775	1.95	5.29	15.1	43.2	123	324	—
Streptococcus pyogenes MGAS6180	NC_007296 (Intergenic)	0.277	0.548	1.19	2.98	7.82	20.3	—	—	—
Streptococcus pyogenes MGAS8232	NC_003485	0.338	0.793	1.96	5.34	15.3	44.0	126	337	—
Streptococcus pyogenes MGAS8232	NC_003485 (Gene)	0.325	0.766	1.92	5.25	15.0	42.9	122	321	—
Streptococcus pyogenes MGAS8232	NC_003485 (Intergenic)	0.308	0.628	1.38	3.50	9.23	24.0	—	—	—
Streptococcus pyogenes MGAS9429	NC_008021	0.336	0.789	1.95	5.31	15.2	43.7	125	334	—
Streptococcus pyogenes MGAS9429	NC_008021 (Gene)	0.327	0.773	1.94	5.30	15.1	43.3	123	324	—
Streptococcus pyogenes MGAS9429	NC_008021 (Intergenic)	0.278	0.552	1.20	3.00	7.83	20.1	—	—	—
Streptococcus pyogenes SSI-1	NC_004606	0.337	0.787	1.95	5.29	15.1	43.7	125	337	—
Streptococcus pyogenes SSI-1	NC_004606 (Gene)	0.324	0.764	1.92	5.24	14.9	42.9	122	322	—

next

Category	SN \ k	L_e (kb) 2	3	4	5	6	7	8	9	10
Streptococcus pyogenes SSI-1	NC_004606 (Intergenic)	0.307	0.619	1.36	3.43	9.07	23.7	56.4	—	—
Streptococcus thermophilus CNRZ1066	NC_006449	0.421	0.998	2.44	6.57	18.6	53.0	148	379	—
Streptococcus thermophilus CNRZ1066	NC_006449 (Gene)	0.419	0.984	2.39	6.38	17.9	50.4	140	353	—
Streptococcus thermophilus CNRZ1066	NC_006449 (Intergenic)	0.305	0.658	1.55	4.07	10.9	28.1	—	—	—
Streptococcus thermophilus LMD-9	NC_008532	0.430	1.02	2.49	6.70	19.0	53.9	151	384	—
Streptococcus thermophilus LMD-9	NC_008532 (Gene)	0.422	0.991	2.40	6.42	18.0	50.6	140	352	—
Streptococcus thermophilus LMD-9	NC_008532 (Intergenic)	0.323	0.663	1.52	3.96	10.5	27.3	—	—	—
Streptococcus thermophilus LMG 18311	NC_006448	0.426	1.01	2.46	6.62	18.8	53.3	149	382	—
Streptococcus thermophilus LMG 18311	NC_006448 (Gene)	0.422	0.991	2.40	6.39	17.9	50.4	140	352	—
Streptococcus thermophilus LMG 18311	NC_006448 (Intergenic)	0.311	0.668	1.57	4.12	11.1	28.8	—	—	—
Streptomyces avermitilis	NC_003155	0.412	0.820	1.48	3.43	8.59	20.3	52.6	142	358
Streptomyces avermitilis	NC_003155 (Gene)	0.354	0.705	1.25	2.87	7.14	16.7	43.2	116	292
Streptomyces avermitilis	NC_003155 (Intergenic)	1.07	1.92	4.32	10.6	26.4	65.8	161	355	—
Streptomyces coelicolor	NC_003888	0.464	0.792	1.32	2.97	7.28	16.6	42.2	112	275
Streptomyces coelicolor	NC_003888 (Gene)	0.421	0.710	1.15	2.58	6.29	14.3	36.1	95.2	234
Streptomyces coelicolor	NC_003888 (Intergenic)	0.703	1.29	3.03	7.42	18.0	43.9	105	—	—
Symbiobacterium thermophilum IAM14863	NC_006177	1.75	1.35	1.86	4.14	10.3	24.0	61.6	161	—
Symbiobacterium thermophilum IAM14863	NC_006177 (Gene)	1.64	1.16	1.57	3.50	8.70	20.1	51.4	134	—
Symbiobacterium thermophilum IAM14863	NC_006177 (Intergenic)	1.62	2.48	4.95	11.0	25.3	56.6	111	—	—
Synechococcus CC9311	NC_008319	0.245	0.633	1.70	4.90	14.8	45.1	138	400	—
Synechococcus CC9311	NC_008319 (Gene)	0.241	0.612	1.62	4.64	13.9	42.1	128	366	—
Synechococcus CC9311	NC_008319 (Intergenic)	0.264	0.730	2.06	5.94	17.4	49.1	117	—	—
Synechococcus CC9605	NC_007516	0.299	0.637	1.43	3.77	10.6	29.9	86.8	246	—
Synechococcus CC9605	NC_007516 (Gene)	0.291	0.602	1.32	3.44	9.60	26.7	76.8	216	—
Synechococcus CC9605	NC_007516 (Intergenic)	0.315	0.819	2.22	6.33	18.3	50.0	115	—	—
Synechococcus CC9902	NC_007513	0.268	0.655	1.69	4.75	14.1	42.4	128	364	—
Synechococcus CC9902	NC_007513 (Gene)	0.265	0.638	1.63	4.54	13.4	40.0	120	339	—
Synechococcus CC9902	NC_007513 (Intergenic)	0.279	0.749	2.08	5.94	17.2	46.2	—	—	—
Synechococcus elongatus PCC 6301	NC_006576	0.305	0.607	1.41	3.34	7.62	16.7	39.7	107	—
Synechococcus elongatus PCC 6301	NC_006576 (Gene)	0.298	0.581	1.34	3.17	7.29	16.2	38.9	105	—
Synechococcus elongatus PCC 6301	NC_006576 (Intergenic)	0.295	0.670	1.63	3.87	8.44	17.1	35.2	—	—
Synechococcus elongatus PCC 7942	NC_007604	0.303	0.605	1.40	3.32	7.57	16.6	39.4	106	—
Synechococcus elongatus PCC 7942	NC_007604 (Gene)	0.295	0.575	1.33	3.14	7.22	16.0	38.4	103	—
Synechococcus elongatus PCC 7942	NC_007604 (Intergenic)	0.300	0.690	1.69	3.98	8.66	17.4	35.5	—	—

next

Category	SN \ k	L_e (kb) 2	3	4	5	6	7	8	9	10
Synechococcus sp WH8102	NC_005070	0.336	0.662	1.41	3.65	10.1	28.0	80.5	226	—
Synechococcus sp WH8102	NC_005070 (Gene)	0.328	0.627	1.31	3.36	9.27	25.5	72.7	203	—
Synechococcus sp WH8102	NC_005070 (Intergenic)	0.380	0.959	2.54	7.16	20.2	52.0	—	—	—
Synechocystis PCC6803	NC_000911	0.223	0.526	1.37	3.62	9.81	26.5	68.1	168	—
Synechocystis PCC6803	NC_000911 (Gene)	0.223	0.518	1.34	3.52	9.46	25.2	64.1	157	—
Synechocystis PCC6803	NC_000911 (Intergenic)	0.193	0.481	1.26	3.40	9.45	25.9	65.4	—	—
Syntrophobacter fumaroxidans MPOB	NC_008554	0.297	0.697	1.81	4.93	14.2	41.4	123	358	941
Syntrophobacter fumaroxidans MPOB	NC_008554 (Gene)	0.299	0.696	1.76	4.72	13.4	38.4	113	326	—
Syntrophobacter fumaroxidans MPOB	NC_008554 (Intergenic)	0.272	0.606	1.57	4.32	12.2	34.4	89.5	—	—
Syntrophomonas wolfei Goettingen	NC_008346	0.423	0.836	2.00	5.30	15.0	44.4	130	357	—
Syntrophomonas wolfei Goettingen	NC_008346 (Gene)	0.402	0.758	1.81	4.78	13.6	40.2	120	339	—
Syntrophomonas wolfei Goettingen	NC_008346 (Intergenic)	0.481	1.07	2.45	6.07	14.9	33.1	61.1	—	—
Syntrophus aciditrophicus SB	NC_007759	0.216	0.489	1.30	3.66	10.9	33.5	103	308	—
Syntrophus aciditrophicus SB	NC_007759 (Gene)	0.214	0.480	1.27	3.57	10.6	32.3	99.3	294	—
Syntrophus aciditrophicus SB	NC_007759 (Intergenic)	0.213	0.503	1.32	3.67	10.6	30.1	76.0	—	—
Thermoanaerobacter tengcongensis	NC_003869	0.232	0.486	1.20	3.23	9.04	25.9	74.3	203	—
Thermoanaerobacter tengcongensis	NC_003869 (Gene)	0.234	0.490	1.21	3.25	9.07	25.9	75.0	210	—
Thermoanaerobacter tengcongensis	NC_003869 (Intergenic)	0.182	0.366	0.848	2.15	5.36	12.0	—	—	—
Thermobifida fusca YX	NC_007333	1.01	1.75	2.75	5.88	14.0	33.0	85.1	223	—
Thermobifida fusca YX	NC_007333 (Gene)	0.834	1.48	2.30	4.90	11.6	27.2	69.8	182	—
Thermobifida fusca YX	NC_007333 (Intergenic)	1.33	1.77	3.60	8.59	21.0	52.8	124	—	—
Thermosynechococcus elongatus	NC_004113	0.291	0.720	1.76	4.49	11.7	30.0	74.8	187	—
Thermosynechococcus elongatus	NC_004113 (Gene)	0.278	0.682	1.65	4.19	10.8	27.6	68.6	171	—
Thermosynechococcus elongatus	NC_004113 (Intergenic)	0.329	0.840	2.15	5.84	15.6	37.2	—	—	—
Thermotoga maritima	NC_000853	0.158	0.325	0.815	2.27	6.65	20.0	60.9	179	—
Thermotoga maritima	NC_000853 (Gene)	0.156	0.320	0.801	2.22	6.52	19.6	59.4	175	—
Thermotoga maritima	NC_000853 (Intergenic)	0.175	0.384	0.934	2.36	5.82	12.7	—	—	—
Thermus thermophilus HB27	NC_005835	0.100	0.233	0.489	1.11	2.73	6.26	15.2	38.6	—
Thermus thermophilus HB27	NC_005835 (Gene)	0.100	0.232	0.478	1.08	2.64	6.00	14.5	36.7	—
Thermus thermophilus HB27	NC_005835 (Intergenic)	0.097	0.226	0.580	1.47	3.77	9.26	—	—	—
Thermus thermophilus HB8	NC_006461	0.099	0.231	0.486	1.11	2.72	6.24	15.1	38.5	—
Thermus thermophilus HB8	NC_006461 (Gene)	0.099	0.230	0.476	1.07	2.63	5.99	14.4	36.7	—
Thermus thermophilus HB8	NC_006461 (Intergenic)	0.095	0.222	0.564	1.43	3.62	8.67	—	—	—
Thiobacillus denitrificans ATCC 25259	NC_007404	0.106	0.266	0.645	1.62	4.39	11.6	31.5	88.3	—

next

Category	SN \ k		L_e (kb)								
			2	3	4	5	6	7	8	9	10
Thiobacillus denitrificans ATCC 25259	NC_007404	(Gene)	0.098	0.246	0.594	1.49	4.01	10.6	28.6	80.0	—
Thiobacillus denitrificans ATCC 25259	NC_007404	(Intergenic)	0.322	0.770	1.90	4.82	12.5	31.2	—	—	—
Thiomicrospira crunogena XCL-2	NC_007520		0.226	0.576	1.56	4.46	13.3	40.1	120	339	—
Thiomicrospira crunogena XCL-2	NC_007520	(Gene)	0.221	0.562	1.53	4.38	13.0	39.4	118	335	—
Thiomicrospira crunogena XCL-2	NC_007520	(Intergenic)	0.188	0.448	1.05	2.70	7.13	18.2	—	—	—
Thiomicrospira denitrificans ATCC 33889	NC_007575		0.309	0.487	1.04	2.63	6.98	19.0	53.0	146	—
Thiomicrospira denitrificans ATCC 33889	NC_007575	(Gene)	0.304	0.480	1.03	2.58	6.85	18.6	51.8	143	—
Thiomicrospira denitrificans ATCC 33889	NC_007575	(Intergenic)	0.279	0.479	1.03	2.42	5.94	14.3	—	—	—
Treponema denticola ATCC 35405	NC_002967		0.238	0.417	0.972	2.55	7.08	20.3	58.6	166	—
Treponema denticola ATCC 35405	NC_002967	(Gene)	0.230	0.405	0.953	2.51	6.96	19.9	57.5	163	—
Treponema denticola ATCC 35405	NC_002967	(Intergenic)	0.306	0.523	1.08	2.56	6.38	15.5	—	—	—
Treponema pallidum	NC_000919		0.468	0.750	1.78	4.84	14.0	41.6	121	312	—
Treponema pallidum	NC_000919	(Gene)	0.443	0.733	1.75	4.77	13.8	40.9	118	303	—
Treponema pallidum	NC_000919	(Intergenic)	0.881	0.790	1.70	4.23	10.6	—	—	—	—
Trichodesmium erythraeum IMS101	NC_008312		0.408	0.961	2.27	5.72	15.1	41.2	114	313	798
Trichodesmium erythraeum IMS101	NC_008312	(Gene)	0.389	0.926	2.20	5.62	14.9	40.2	111	298	725
Trichodesmium erythraeum IMS101	NC_008312	(Intergenic)	0.416	0.897	1.95	4.60	11.5	29.8	77.8	195	—
Tropheryma whipplei TW08 27	NC_004551		0.661	1.30	3.15	8.55	24.1	68.2	177	—	—
Tropheryma whipplei TW08 27	NC_004551	(Gene)	0.545	1.15	2.88	7.91	22.5	64.5	170	—	—
Tropheryma whipplei TW08 27	NC_004551	(Intergenic)	0.385	0.754	1.72	4.15	9.92	21.0	—	—	—
Tropheryma whipplei Twist	NC_004572		0.658	1.29	3.14	8.52	24.1	68.0	177	—	—
Tropheryma whipplei Twist	NC_004572	(Gene)	0.545	1.15	2.87	7.90	22.6	64.8	172	—	—
Tropheryma whipplei Twist	NC_004572	(Intergenic)	0.291	0.610	1.43	3.46	8.31	17.7	—	—	—
Ureaplasma urealyticum	NC_002162		0.212	0.461	1.05	2.53	6.04	14.4	35.1	—	—
Ureaplasma urealyticum	NC_002162	(Gene)	0.201	0.435	1.00	2.41	5.73	13.7	33.2	—	—
Ureaplasma urealyticum	NC_002162	(Intergenic)	0.261	0.521	1.06	2.28	4.88	—	—	—	—
Vibrio cholerae	NC_002505		0.318	0.729	1.97	5.48	16.0	48.0	144	411	—
Vibrio cholerae	NC_002505	(Gene)	0.301	0.680	1.83	5.08	14.8	44.2	133	376	—
Vibrio cholerae	NC_002505	(Intergenic)	0.382	0.942	2.33	6.06	16.2	40.5	86.3	—	—
Vibrio cholerae	NC_002506		0.294	0.700	1.90	5.30	15.2	43.2	110	224	—
Vibrio cholerae	NC_002506	(Gene)	0.278	0.648	1.76	4.91	14.3	42.2	119	—	—
Vibrio cholerae	NC_002506	(Intergenic)	0.308	0.767	1.80	3.84	6.88	9.75	—	—	—
Vibrio fischeri ES114	NC_006840		0.516	1.17	2.77	6.98	18.9	52.7	148	397	—
Vibrio fischeri ES114	NC_006840	(Gene)	0.486	1.08	2.51	6.34	17.2	47.6	134	358	—

next

Category	SN \ k	L_e (kb) 2	3	4	5	6	7	8	9	10
Vibrio fischeri ES114	NC_006840 (Intergenic)	0.551	1.27	2.73	5.91	14.0	32.5	71.0	—	—
Vibrio fischeri ES114	NC_006841	0.525	1.25	3.00	7.54	20.2	55.1	148	359	—
Vibrio fischeri ES114	NC_006841 (Gene)	0.480	1.13	2.71	6.84	18.4	50.3	136	328	—
Vibrio fischeri ES114	NC_006841 (Intergenic)	0.858	1.92	3.92	8.14	17.8	35.7	—	—	—
Vibrio parahaemolyticus	NC_004603	0.351	0.865	2.33	6.57	19.4	58.4	175	493	—
Vibrio parahaemolyticus	NC_004603 (Gene)	0.336	0.811	2.15	6.00	17.6	52.5	157	439	—
Vibrio parahaemolyticus	NC_004603 (Intergenic)	0.354	0.906	2.36	6.37	17.5	45.3	103	—	—
Vibrio parahaemolyticus	NC_004605	0.301	0.759	2.08	5.89	17.5	52.8	157	424	—
Vibrio parahaemolyticus	NC_004605 (Gene)	0.279	0.693	1.88	5.32	15.7	47.2	139	374	—
Vibrio parahaemolyticus	NC_004605 (Intergenic)	0.409	1.05	2.77	7.41	19.9	49.1	—	—	—
Vibrio vulnificus CMCP6	NC_004459	0.303	0.740	2.01	5.70	17.0	51.4	155	436	—
Vibrio vulnificus CMCP6	NC_004459 (Gene)	0.294	0.699	1.87	5.26	15.5	46.9	141	403	—
Vibrio vulnificus CMCP6	NC_004459 (Intergenic)	0.270	0.693	1.81	4.95	13.6	33.9	70.2	—	—
Vibrio vulnificus CMCP6	NC_004460	0.265	0.651	1.75	4.96	14.7	44.5	134	370	—
Vibrio vulnificus CMCP6	NC_004460 (Gene)	0.248	0.599	1.61	4.51	13.3	40.0	119	328	—
Vibrio vulnificus CMCP6	NC_004460 (Intergenic)	0.329	0.836	2.14	5.76	15.9	41.5	—	—	—
Vibrio vulnificus YJ016	NC_005139	0.309	0.749	2.03	5.75	17.1	51.8	156	438	—
Vibrio vulnificus YJ016	NC_005139 (Gene)	0.297	0.709	1.90	5.37	15.9	48.1	145	417	—
Vibrio vulnificus YJ016	NC_005139 (Intergenic)	0.298	0.755	1.94	5.22	13.9	32.9	63.4	—	—
Vibrio vulnificus YJ016	NC_005140	0.264	0.648	1.74	4.91	14.5	44.1	132	368	—
Vibrio vulnificus YJ016	NC_005140 (Gene)	0.249	0.604	1.62	4.55	13.4	40.5	121	335	—
Vibrio vulnificus YJ016	NC_005140 (Intergenic)	0.358	0.893	2.25	5.93	15.7	38.9	—	—	—
Wigglesworthia brevipalpis	NC_004344	0.279	0.443	0.887	1.93	4.24	9.66	22.1	—	—
Wigglesworthia brevipalpis	NC_004344 (Gene)	0.268	0.426	0.860	1.89	4.17	9.61	22.4	—	—
Wigglesworthia brevipalpis	NC_004344 (Intergenic)	0.279	0.451	0.795	1.41	2.48	4.43	—	—	—
Wolbachia endosymbiont of Brugia malayi TRS	NC_006833	0.482	1.14	2.78	7.30	20.1	55.6	147	336	—
Wolbachia endosymbiont of Brugia malayi TRS	NC_006833 (Gene)	0.447	1.06	2.60	6.85	18.8	51.9	136	—	—
Wolbachia endosymbiont of Brugia malayi TRS	NC_006833 (Intergenic)	0.479	1.12	2.70	6.83	17.6	41.9	83.6	—	—
Wolbachia endosymbiont of Drosophila melanogaster	NC_002978	0.431	1.00	2.47	6.49	17.8	48.5	125	275	—
Wolbachia endosymbiont of Drosophila melanogaster	NC_002978 (Gene)	0.381	0.901	2.23	5.93	16.4	45.7	123	290	—
Wolbachia endosymbiont of Drosophila melanogaster	NC_002978 (Intergenic)	0.769	1.49	2.84	5.78	11.0	18.3	—	—	—
Wolinella succinogenes	NC_005090	0.150	0.307	0.744	1.97	5.48	15.8	46.1	133	—
Wolinella succinogenes	NC_005090 (Gene)	0.149	0.303	0.733	1.94	5.37	15.4	44.8	129	—
Wolinella succinogenes	NC_005090 (Intergenic)	0.138	0.316	0.784	2.09	5.64	14.8	—	—	—

next

Category	SN \ k	L_e (kb) 2	3	4	5	6	7	8	9	10
Xanthomonas campestris	NC_003902	0.144	0.321	0.757	1.93	5.27	14.2	39.6	112	301
Xanthomonas campestris	NC_003902 (Gene)	0.140	0.298	0.684	1.72	4.64	12.3	33.9	95.0	254
Xanthomonas campestris	NC_003902 (Intergenic)	0.169	0.440	1.18	3.24	9.05	25.1	65.5	—	—
Xanthomonas campestris 8004	NC_007086	0.146	0.325	0.770	1.97	5.37	14.5	40.4	114	308
Xanthomonas campestris 8004	NC_007086 (Gene)	0.142	0.303	0.697	1.76	4.74	12.6	34.7	97.5	260
Xanthomonas campestris 8004	NC_007086 (Intergenic)	0.173	0.452	1.22	3.34	9.32	25.9	67.5	—	—
Xanthomonas campestris vesicatoria 85-10	NC_007508	0.143	0.320	0.765	1.97	5.41	14.7	41.4	118	322
Xanthomonas campestris vesicatoria 85-10	NC_007508 (Gene)	0.139	0.300	0.704	1.79	4.89	13.1	36.6	104	280
Xanthomonas campestris vesicatoria 85-10	NC_007508 (Intergenic)	0.184	0.479	1.27	3.50	9.81	27.5	73.2	—	—
Xanthomonas citri	NC_003919	0.142	0.318	0.763	1.97	5.43	14.8	41.6	119	325
Xanthomonas citri	NC_003919 (Gene)	0.138	0.298	0.700	1.78	4.87	13.1	36.5	103	279
Xanthomonas citri	NC_003919 (Intergenic)	0.176	0.456	1.21	3.35	9.43	26.6	71.5	—	—
Xanthomonas oryzae KACC10331	NC_006834	0.147	0.338	0.829	2.17	6.06	16.6	46.7	129	318
Xanthomonas oryzae KACC10331	NC_006834 (Gene)	0.139	0.310	0.745	1.93	5.32	14.4	40.1	110	—
Xanthomonas oryzae KACC10331	NC_006834 (Intergenic)	0.188	0.485	1.30	3.62	10.3	28.7	74.3	—	—
Xanthomonas oryzae MAFF 311018	NC_007705	0.147	0.337	0.828	2.17	6.04	16.6	46.4	127	309
Xanthomonas oryzae MAFF 311018	NC_007705 (Gene)	0.139	0.310	0.745	1.93	5.31	14.4	39.8	108	—
Xanthomonas oryzae MAFF 311018	NC_007705 (Intergenic)	0.199	0.521	1.41	3.95	11.2	30.8	76.8	—	—
Xylella fastidiosa	NC_002488	0.282	0.775	2.07	5.80	17.1	51.0	152	426	—
Xylella fastidiosa	NC_002488 (Gene)	0.273	0.725	1.92	5.37	15.8	46.7	138	381	—
Xylella fastidiosa	NC_002488 (Intergenic)	0.297	0.894	2.42	6.62	18.7	51.3	124	—	—
Xylella fastidiosa Temecula1	NC_004556	0.339	0.880	2.29	6.33	18.5	54.7	161	439	—
Xylella fastidiosa Temecula1	NC_004556 (Gene)	0.325	0.811	2.09	5.79	16.9	49.5	144	384	—
Xylella fastidiosa Temecula1	NC_004556 (Intergenic)	0.380	1.01	2.55	6.68	18.4	50.4	124	—	—
Yersinia pestis Antiqua	NC_008150	0.549	1.10	2.58	6.68	18.7	55.1	162	435	931
Yersinia pestis Antiqua	NC_008150 (Gene)	0.477	0.937	2.24	5.85	16.5	48.5	142	381	—
Yersinia pestis Antiqua	NC_008150 (Intergenic)	1.20	2.37	4.27	9.26	22.1	52.6	112	—	—
Yersinia pestis biovar Mediaevails	NC_005810	0.548	1.09	2.57	6.64	18.6	55.2	165	470	1124
Yersinia pestis biovar Mediaevails	NC_005810 (Gene)	0.475	0.938	2.24	5.86	16.5	49.1	147	419	—
Yersinia pestis biovar Mediaevails	NC_005810 (Intergenic)	1.35	2.56	4.42	9.41	22.4	53.9	117	—	—
Yersinia pestis CO92	NC_003143	0.547	1.09	2.57	6.66	18.7	55.2	164	457	1046
Yersinia pestis CO92	NC_003143 (Gene)	0.476	0.936	2.24	5.84	16.5	48.8	145	404	—
Yersinia pestis CO92	NC_003143 (Intergenic)	1.17	2.34	4.22	9.19	22.0	52.8	113	—	—
Yersinia pestis KIM	NC_004088	0.547	1.09	2.57	6.66	18.7	55.3	165	467	1102

next

Category	SN \ k	L_e (kb) 2	3	4	5	6	7	8	9	10
Yersinia pestis KIM	NC_004088 (Gene)	0.474	0.936	2.24	5.84	16.5	48.9	146	414	—
Yersinia pestis KIM	NC_004088 (Intergenic)	1.27	2.48	4.42	9.56	22.9	55.3	121	—	—
Yersinia pestis Nepal516	NC_008149	0.547	1.09	2.57	6.66	18.7	55.3	165	463	1083
Yersinia pestis Nepal516	NC_008149 (Gene)	0.475	0.932	2.23	5.82	16.4	48.6	145	409	—
Yersinia pestis Nepal516	NC_008149 (Intergenic)	1.18	2.37	4.27	9.27	22.2	53.1	114	—	—
Yersinia pseudotuberculosis IP32953	NC_006155	0.542	1.08	2.54	6.57	18.4	54.7	165	483	1235
Yersinia pseudotuberculosis IP32953	NC_006155 (Gene)	0.469	0.919	2.20	5.73	16.1	48.1	145	426	—
Yersinia pseudotuberculosis IP32953	NC_006155 (Intergenic)	1.21	2.47	4.34	9.36	22.7	56.0	128	—	—
Zymomonas mobilis ZM4	NC_006526	0.262	0.527	1.27	3.36	9.48	28.1	83.9	242	—
Zymomonas mobilis ZM4	NC_006526 (Gene)	0.255	0.512	1.25	3.30	9.27	27.3	81.4	232	—
Zymomonas mobilis ZM4	NC_006526 (Intergenic)	0.234	0.451	0.997	2.56	6.94	18.9	48.5	—	—

Effective Length List 2: Unicell (106).

Category	SN \ k	L_e (kb) 2	3	4	5	6	7	8	9	10
Fungi (92)										
Aspergillus fumigatus	NC_007194	0.786	1.97	4.82	13.4	40.1	119	349	942	2026
Aspergillus fumigatus	NC_007194 (Gene)	0.695	1.74	4.22	11.8	35.1	103	299	767	—
Aspergillus fumigatus	NC_007194 (Intergenic)	0.908	2.26	5.58	15.4	44.8	128	341	763	—
Aspergillus fumigatus	NC_007194 (Exon)	0.650	1.64	3.97	11.1	33.2	97.8	282	719	—
Aspergillus fumigatus	NC_007194 (Intron)	1.23	2.85	6.37	15.1	36.7	81.0	149	—	—
Aspergillus fumigatus	NC_007195	0.778	1.96	4.75	13.2	39.0	115	337	907	1958
Aspergillus fumigatus	NC_007195 (Gene)	0.726	1.83	4.40	12.2	36.3	107	307	788	—
Aspergillus fumigatus	NC_007195 (Intergenic)	0.845	2.13	5.20	14.2	41.2	117	309	691	—
Aspergillus fumigatus	NC_007195 (Exon)	0.653	1.66	4.01	11.2	33.5	98.9	286	730	—
Aspergillus fumigatus	NC_007195 (Intron)	1.45	3.47	7.67	17.5	41.6	90.8	166	—	—
Aspergillus fumigatus	NC_007196	0.837	2.11	5.13	14.2	42.3	125	362	943	—
Aspergillus fumigatus	NC_007196 (Gene)	0.700	1.77	4.33	12.1	36.2	107	303	739	—
Aspergillus fumigatus	NC_007196 (Intergenic)	0.983	2.49	6.01	16.4	47.1	133	348	752	—
Aspergillus fumigatus	NC_007196 (Exon)	0.667	1.69	4.15	11.7	34.9	102	289	695	—
Aspergillus fumigatus	NC_007196 (Intron)	1.10	2.62	6.02	15.1	38.7	86.1	—	—	—

next

			L_e (kb)								
Category	SN	$\backslash\ k$	2	3	4	5	6	7	8	9	10
Aspergillus fumigatus	NC_007197		0.844	2.13	5.14	14.2	42.3	124	355	898	—
Aspergillus fumigatus	NC_007197 (Gene)		0.806	2.04	4.88	13.5	39.7	114	309	695	—
Aspergillus fumigatus	NC_007197 (Intergenic)		0.882	2.23	5.41	15.0	44.1	126	337	736	—
Aspergillus fumigatus	NC_007197 (Exon)		0.799	2.02	4.85	13.4	39.5	113	303	667	—
Aspergillus fumigatus	NC_007197 (Intron)		0.878	2.21	5.17	13.5	35.2	78.5	—	—	—
Aspergillus fumigatus	NC_007198		0.709	1.80	4.50	12.7	38.2	114	336	889	—
Aspergillus fumigatus	NC_007198 (Gene)		0.656	1.67	4.15	11.7	35.2	104	297	734	—
Aspergillus fumigatus	NC_007198 (Intergenic)		0.786	1.98	4.97	13.9	41.2	119	318	692	—
Aspergillus fumigatus	NC_007198 (Exon)		0.629	1.60	3.98	11.3	33.9	100.0	284	694	—
Aspergillus fumigatus	NC_007198 (Intron)		0.990	2.42	5.89	14.6	36.9	79.9	—	—	—
Aspergillus fumigatus	NC_007199		0.855	2.16	5.23	14.5	43.0	126	361	916	—
Aspergillus fumigatus	NC_007199 (Gene)		0.765	1.93	4.72	13.2	39.3	115	319	753	—
Aspergillus fumigatus	NC_007199 (Intergenic)		0.952	2.42	5.79	15.8	45.4	127	328	687	—
Aspergillus fumigatus	NC_007199 (Exon)		0.762	1.92	4.70	13.2	39.1	114	314	723	—
Aspergillus fumigatus	NC_007199 (Intron)		0.802	2.04	4.92	13.3	35.7	82.0	—	—	—
Aspergillus fumigatus	NC_007200		0.840	2.11	5.22	14.6	43.5	126	345	777	—
Aspergillus fumigatus	NC_007200 (Gene)		0.739	1.86	4.54	12.6	37.3	106	271	—	—
Aspergillus fumigatus	NC_007200 (Intergenic)		0.927	2.32	5.82	16.2	47.0	128	306	—	—
Aspergillus fumigatus	NC_007200 (Exon)		0.703	1.77	4.31	12.0	35.4	99.9	254	—	—
Aspergillus fumigatus	NC_007200 (Intron)		0.966	2.35	5.72	14.0	32.6	61.5	—	—	—
Aspergillus fumigatus	NC_007201		0.919	2.28	5.55	15.4	45.4	131	354	780	—
Aspergillus fumigatus	NC_007201 (Gene)		0.768	1.90	4.62	12.9	38.1	108	273	—	—
Aspergillus fumigatus	NC_007201 (Intergenic)		1.03	2.57	6.28	17.0	48.0	128	295	—	—
Aspergillus fumigatus	NC_007201 (Exon)		0.722	1.79	4.37	12.2	36.1	101	254	—	—
Aspergillus fumigatus	NC_007201 (Intron)		1.25	2.76	6.30	14.4	31.3	55.9	—	—	—
Candida albicans	NC_007436		0.275	0.691	1.70	4.21	10.7	25.5	58.0	—	—
Candida albicans	NC_007436 (Gene)		0.227	0.568	1.39	3.58	9.54	25.0	62.3	—	—
Candida albicans	NC_007436 (Intergenic)		0.315	0.660	1.42	2.87	5.72	10.8	20.5	—	—
Candida albicans	NC_007436 (Exon)		0.226	0.567	1.39	3.57	9.51	24.9	62.1	—	—
Candida albicans	NC_007436 (Intron)		0.125	0.237	0.473	—	—	—	—	—	—
Candida glabrata CBS138	NC_005967		0.747	1.71	3.87	10.1	27.6	71.0	159	—	—
Candida glabrata CBS138	NC_005967 (Gene)		0.638	1.43	3.05	7.79	21.1	53.0	115	—	—
Candida glabrata CBS138	NC_005967 (Intergenic)		0.909	1.63	3.57	8.52	20.3	43.8	—	—	—
Candida glabrata CBS138	NC_005967 (Exon)		0.617	1.39	2.97	7.59	20.5	51.8	113	—	—

next

Category	SN \ k	L_e (kb) 2	3	4	5	6	7	8	9	10
Candida glabrata CBS138	NC_005967 (Intron)	0.400	0.796	1.35	—	—	—	—	—	—
Candida glabrata CBS138	NC_005968	0.729	1.68	3.89	10.2	27.8	71.5	162	—	—
Candida glabrata CBS138	NC_005968 (Gene)	0.586	1.32	2.95	7.66	21.0	54.4	121	—	—
Candida glabrata CBS138	NC_005968 (Intergenic)	1.02	1.87	3.99	9.13	20.2	40.9	—	—	—
Candida glabrata CBS138	NC_005968 (Exon)	0.573	1.30	2.91	7.58	20.8	54.1	121	—	—
Candida glabrata CBS138	NC_005968 (Intron)	0.133	0.274	—	—	—	—	—	—	—
Candida glabrata CBS138	NC_006026	0.842	1.86	4.15	10.8	29.2	74.6	167	—	—
Candida glabrata CBS138	NC_006026 (Gene)	0.655	1.47	3.12	7.95	21.5	54.6	120	—	—
Candida glabrata CBS138	NC_006026 (Intergenic)	1.23	2.04	4.48	10.7	24.4	49.4	—	—	—
Candida glabrata CBS138	NC_006026 (Exon)	0.640	1.44	3.08	7.86	21.3	54.4	121	—	—
Candida glabrata CBS138	NC_006026 (Intron)	0.070	0.165	—	—	—	—	—	—	—
Candida glabrata CBS138	NC_006027	0.705	1.63	3.84	10.2	28.3	75.4	181	—	—
Candida glabrata CBS138	NC_006027 (Gene)	0.569	1.32	2.94	7.66	21.1	55.9	132	—	—
Candida glabrata CBS138	NC_006027 (Intergenic)	1.04	1.86	4.22	10.2	23.9	50.9	—	—	—
Candida glabrata CBS138	NC_006027 (Exon)	0.557	1.29	2.89	7.54	20.8	55.1	130	—	—
Candida glabrata CBS138	NC_006027 (Intron)	0.192	0.391	0.681	—	—	—	—	—	—
Candida glabrata CBS138	NC_006028	0.676	1.58	3.65	9.57	26.4	69.3	163	—	—
Candida glabrata CBS138	NC_006028 (Gene)	0.560	1.32	2.91	7.50	20.6	53.9	125	—	—
Candida glabrata CBS138	NC_006028 (Intergenic)	0.916	1.61	3.58	8.43	19.6	41.7	—	—	—
Candida glabrata CBS138	NC_006029	0.667	1.56	3.70	9.80	27.2	73.5	185	—	—
Candida glabrata CBS138	NC_006029 (Gene)	0.533	1.29	2.94	7.69	21.4	58.1	145	—	—
Candida glabrata CBS138	NC_006029 (Intergenic)	0.939	1.61	3.52	8.43	20.1	45.4	94.5	—	—
Candida glabrata CBS138	NC_006029 (Exon)	0.529	1.28	2.91	7.62	21.3	57.6	144	—	—
Candida glabrata CBS138	NC_006029 (Intron)	0.379	0.707	1.10	—	—	—	—	—	—
Candida glabrata CBS138	NC_006030	0.669	1.56	3.65	9.66	26.8	72.5	184	—	—
Candida glabrata CBS138	NC_006030 (Gene)	0.553	1.30	2.91	7.61	21.2	57.8	147	—	—
Candida glabrata CBS138	NC_006030 (Intergenic)	0.973	1.68	3.65	8.50	19.5	42.3	86.3	—	—
Candida glabrata CBS138	NC_006030 (Exon)	0.547	1.28	2.87	7.49	20.9	56.9	144	—	—
Candida glabrata CBS138	NC_006030 (Intron)	0.433	0.875	1.41	2.30	—	—	—	—	—
Candida glabrata CBS138	NC_006031	0.671	1.56	3.65	9.70	27.0	73.8	189	410	—
Candida glabrata CBS138	NC_006031 (Gene)	0.548	1.30	2.95	7.76	21.6	59.3	151	—	—
Candida glabrata CBS138	NC_006031 (Intergenic)	0.979	1.62	3.56	8.48	20.1	44.9	93.3	—	—
Candida glabrata CBS138	NC_006031 (Exon)	0.546	1.29	2.94	7.71	21.5	59.0	150	—	—
Candida glabrata CBS138	NC_006031 (Intron)	0.825	1.30	1.65	—	—	—	—	—	—

next

Category	SN \ k	L_e (kb) 2	3	4	5	6	7	8	9	10
Candida glabrata CBS138	NC_006032	0.750	1.70	3.94	10.4	28.8	77.3	194	406	—
Candida glabrata CBS138	NC_006032 (Gene)	0.644	1.47	3.24	8.46	23.5	63.5	157	—	—
Candida glabrata CBS138	NC_006032 (Intergenic)	0.911	1.55	3.40	8.01	18.9	42.1	88.2	—	—
Candida glabrata CBS138	NC_006032 (Exon)	0.622	1.42	3.18	8.34	23.2	63.0	157	—	—
Candida glabrata CBS138	NC_006032 (Intron)	0.271	0.569	0.925	—	—	—	—	—	—
Candida glabrata CBS138	NC_006033	0.684	1.60	3.72	9.81	27.5	75.2	193	423	—
Candida glabrata CBS138	NC_006033 (Gene)	0.567	1.33	2.96	7.73	21.6	59.1	151	—	—
Candida glabrata CBS138	NC_006033 (Intergenic)	0.994	1.74	3.84	9.15	21.8	48.6	99.9	—	—
Candida glabrata CBS138	NC_006033 (Exon)	0.554	1.30	2.91	7.61	21.3	58.5	150	—	—
Candida glabrata CBS138	NC_006033 (Intron)	0.497	0.925	1.65	2.64	—	—	—	—	—
Candida glabrata CBS138	NC_006034	0.660	1.56	3.71	9.85	27.4	75.1	196	443	—
Candida glabrata CBS138	NC_006034 (Gene)	0.548	1.31	3.02	7.98	22.5	62.2	162	—	—
Candida glabrata CBS138	NC_006034 (Intergenic)	0.860	1.58	3.50	8.34	19.8	45.0	97.2	—	—
Candida glabrata CBS138	NC_006034 (Exon)	0.550	1.31	3.02	7.96	22.4	62.1	162	—	—
Candida glabrata CBS138	NC_006034 (Intron)	0.353	0.798	1.44	2.23	—	—	—	—	—
Candida glabrata CBS138	NC_006035	0.671	1.55	3.66	9.72	27.1	73.9	191	425	—
Candida glabrata CBS138	NC_006035 (Gene)	0.554	1.28	2.92	7.69	21.5	58.7	149	—	—
Candida glabrata CBS138	NC_006035 (Intergenic)	0.876	1.57	3.44	8.30	20.1	46.5	102	—	—
Candida glabrata CBS138	NC_006035 (Exon)	0.552	1.29	2.93	7.73	21.8	60.5	160	—	—
Candida glabrata CBS138	NC_006035 (Intron)	0.555	0.919	1.16	—	—	—	—	—	—
Candida glabrata CBS138	NC_006036	0.687	1.60	3.78	10.0	28.0	77.1	203	465	—
Candida glabrata CBS138	NC_006036 (Gene)	0.580	1.38	3.11	8.14	22.8	63.5	168	—	—
Candida glabrata CBS138	NC_006036 (Intergenic)	0.899	1.55	3.47	8.28	19.6	43.9	93.3	—	—
Candida glabrata CBS138	NC_006036 (Exon)	0.577	1.37	3.09	8.08	22.6	63.0	167	—	—
Candida glabrata CBS138	NC_006036 (Intron)	0.669	1.03	1.84	3.13	—	—	—	—	—
Cryptococcus neoformans var JEC21	NC_006670	0.653	1.57	3.65	9.77	28.2	81.2	231	595	—
Cryptococcus neoformans var JEC21	NC_006670 (Gene)	0.588	1.41	3.32	8.97	26.0	74.3	209	522	—
Cryptococcus neoformans var JEC21	NC_006670 (Intergenic)	0.877	2.03	4.43	10.9	28.9	75.3	174	—	—
Cryptococcus neoformans var JEC21	NC_006670 (Exon)	0.454	1.10	2.59	7.04	20.6	59.0	166	419	—
Cryptococcus neoformans var JEC21	NC_006670 (Intron)	2.42	2.32	3.46	5.54	10.3	19.1	36.1	—	—
Cryptococcus neoformans var JEC21	NC_006679	0.622	1.48	3.39	8.91	25.2	70.3	187	419	—
Cryptococcus neoformans var JEC21	NC_006679 (Gene)	0.573	1.36	3.10	8.16	23.1	63.1	162	—	—
Cryptococcus neoformans var JEC21	NC_006679 (Intergenic)	0.730	1.71	3.83	9.66	25.5	63.9	134	—	—
Cryptococcus neoformans var JEC21	NC_006679 (Exon)	0.452	1.08	2.44	6.49	18.5	50.8	131	—	—

next

Category	SN	\ k	L_e (kb) 2	3	4	5	6	7	8	9	10
Cryptococcus neoformans var JEC21	NC_006679 (Intron)		2.01	2.17	3.06	4.76	8.64	15.6	—	—	—
Cryptococcus neoformans var JEC21	NC_006680		0.629	1.49	3.42	9.00	25.5	70.6	185	—	—
Cryptococcus neoformans var JEC21	NC_006680 (Gene)		0.560	1.33	3.06	8.09	22.9	62.5	161	—	—
Cryptococcus neoformans var JEC21	NC_006680 (Intergenic)		0.843	1.92	4.24	10.4	26.7	63.0	—	—	—
Cryptococcus neoformans var JEC21	NC_006680 (Exon)		0.432	1.04	2.39	6.38	18.2	50.0	129	—	—
Cryptococcus neoformans var JEC21	NC_006680 (Intron)		2.14	2.21	3.32	5.30	9.77	17.6	—	—	—
Cryptococcus neoformans var JEC21	NC_006681		0.658	1.57	3.69	9.85	28.0	77.6	201	—	—
Cryptococcus neoformans var JEC21	NC_006681 (Gene)		0.610	1.44	3.35	8.93	25.2	68.4	170	—	—
Cryptococcus neoformans var JEC21	NC_006681 (Intergenic)		0.760	1.83	4.28	10.9	28.6	68.8	134	—	—
Cryptococcus neoformans var JEC21	NC_006681 (Exon)		0.462	1.10	2.55	6.88	19.7	53.7	134	—	—
Cryptococcus neoformans var JEC21	NC_006681 (Intron)		1.84	2.10	3.31	5.39	9.95	17.9	—	—	—
Cryptococcus neoformans var JEC21	NC_006682		0.669	1.58	3.61	9.48	26.5	72.3	183	—	—
Cryptococcus neoformans var JEC21	NC_006682 (Gene)		0.612	1.42	3.23	8.44	23.5	62.7	154	—	—
Cryptococcus neoformans var JEC21	NC_006682 (Intergenic)		0.803	1.94	4.44	11.1	28.2	64.7	—	—	—
Cryptococcus neoformans var JEC21	NC_006682 (Exon)		0.476	1.10	2.49	6.57	18.4	49.2	121	—	—
Cryptococcus neoformans var JEC21	NC_006682 (Intron)		1.84	1.99	3.10	5.05	9.33	16.7	—	—	—
Cryptococcus neoformans var JEC21	NC_006683		0.648	1.53	3.50	9.20	25.8	70.6	178	—	—
Cryptococcus neoformans var JEC21	NC_006683 (Gene)		0.597	1.39	3.16	8.28	23.1	61.8	151	—	—
Cryptococcus neoformans var JEC21	NC_006683 (Intergenic)		0.742	1.75	3.99	9.99	25.6	59.9	—	—	—
Cryptococcus neoformans var JEC21	NC_006683 (Exon)		0.450	1.05	2.37	6.28	17.7	47.5	115	—	—
Cryptococcus neoformans var JEC21	NC_006683 (Intron)		2.33	2.29	3.25	4.92	8.65	14.9	—	—	—
Cryptococcus neoformans var JEC21	NC_006684		0.630	1.51	3.55	9.51	27.3	77.7	215	520	—
Cryptococcus neoformans var JEC21	NC_006684 (Gene)		0.559	1.34	3.13	8.39	24.1	67.7	184	434	—
Cryptococcus neoformans var JEC21	NC_006684 (Intergenic)		0.855	2.02	4.68	12.0	31.8	79.9	168	—	—
Cryptococcus neoformans var JEC21	NC_006684 (Exon)		0.431	1.04	2.41	6.51	18.8	53.0	144	—	—
Cryptococcus neoformans var JEC21	NC_006684 (Intron)		2.25	2.36	3.60	5.79	10.7	19.6	—	—	—
Cryptococcus neoformans var JEC21	NC_006685		0.653	1.58	3.70	9.90	28.5	81.8	229	573	—
Cryptococcus neoformans var JEC21	NC_006685 (Gene)		0.574	1.38	3.26	8.79	25.4	72.5	201	489	—
Cryptococcus neoformans var JEC21	NC_006685 (Intergenic)		0.866	2.09	4.66	11.7	31.2	80.3	178	—	—
Cryptococcus neoformans var JEC21	NC_006685 (Exon)		0.440	1.07	2.52	6.89	20.1	57.6	161	394	—
Cryptococcus neoformans var JEC21	NC_006685 (Intron)		2.08	2.18	3.36	5.47	10.2	18.9	—	—	—
Cryptococcus neoformans var JEC21	NC_006686		0.633	1.54	3.64	9.83	28.4	81.5	227	557	—
Cryptococcus neoformans var JEC21	NC_006686 (Gene)		0.559	1.36	3.26	8.86	25.8	73.7	204	487	—
Cryptococcus neoformans var JEC21	NC_006686 (Intergenic)		0.924	2.13	4.74	11.7	30.0	73.4	155	—	—

next

Category	SN \ k		L_e (kb) 2	3	4	5	6	7	8	9	10
Cryptococcus neoformans var JEC21	NC_006686	(Exon)	0.432	1.07	2.56	7.04	20.7	59.4	165	397	—
Cryptococcus neoformans var JEC21	NC_006686	(Intron)	2.10	2.19	3.37	5.45	10.2	18.9	—	—	—
Cryptococcus neoformans var JEC21	NC_006687		0.628	1.51	3.52	9.39	27.0	76.7	210	501	—
Cryptococcus neoformans var JEC21	NC_006687	(Gene)	0.576	1.38	3.21	8.59	24.7	69.7	189	437	—
Cryptococcus neoformans var JEC21	NC_006687	(Intergenic)	0.791	1.91	4.38	11.0	28.7	70.6	145	—	—
Cryptococcus neoformans var JEC21	NC_006687	(Exon)	0.443	1.06	2.47	6.67	19.3	54.8	149	—	—
Cryptococcus neoformans var JEC21	NC_006687	(Intron)	1.95	2.13	3.20	5.17	9.66	17.8	—	—	—
Cryptococcus neoformans var JEC21	NC_006691		0.659	1.58	3.68	9.82	28.1	79.7	217	510	—
Cryptococcus neoformans var JEC21	NC_006691	(Gene)	0.603	1.44	3.34	8.91	25.5	71.4	191	433	—
Cryptococcus neoformans var JEC21	NC_006691	(Intergenic)	0.840	1.98	4.53	11.4	29.7	73.0	151	—	—
Cryptococcus neoformans var JEC21	NC_006691	(Exon)	0.455	1.09	2.52	6.80	19.6	55.3	149	—	—
Cryptococcus neoformans var JEC21	NC_006691	(Intron)	2.42	2.56	3.69	5.63	10.3	18.7	—	—	—
Cryptococcus neoformans var JEC21	NC_006692		0.631	1.52	3.52	9.36	26.7	75.5	205	482	—
Cryptococcus neoformans var JEC21	NC_006692	(Gene)	0.585	1.39	3.21	8.52	24.2	67.4	179	—	—
Cryptococcus neoformans var JEC21	NC_006692	(Intergenic)	0.736	1.78	4.12	10.4	27.7	70.1	150	—	—
Cryptococcus neoformans var JEC21	NC_006692	(Exon)	0.452	1.08	2.49	6.69	19.2	53.8	144	—	—
Cryptococcus neoformans var JEC21	NC_006692	(Intron)	2.64	2.32	3.32	5.08	9.22	16.7	—	—	—
Cryptococcus neoformans var JEC21	NC_006693		0.626	1.51	3.51	9.30	26.4	73.8	198	453	—
Cryptococcus neoformans var JEC21	NC_006693	(Gene)	0.571	1.37	3.17	8.40	23.8	65.4	170	—	—
Cryptococcus neoformans var JEC21	NC_006693	(Intergenic)	0.769	1.85	4.26	10.8	28.4	71.0	149	—	—
Cryptococcus neoformans var JEC21	NC_006693	(Exon)	0.441	1.07	2.47	6.62	19.0	52.4	137	—	—
Cryptococcus neoformans var JEC21	NC_006693	(Intron)	1.68	2.00	3.18	5.22	9.65	17.6	—	—	—
Cryptococcus neoformans var JEC21	NC_006694		0.622	1.49	3.48	9.26	26.4	74.3	199	454	—
Cryptococcus neoformans var JEC21	NC_006694	(Gene)	0.557	1.33	3.11	8.27	23.5	65.2	172	—	—
Cryptococcus neoformans var JEC21	NC_006694	(Intergenic)	0.820	1.94	4.39	11.0	28.5	68.6	135	—	—
Cryptococcus neoformans var JEC21	NC_006694	(Exon)	0.429	1.03	2.40	6.47	18.6	51.9	137	—	—
Cryptococcus neoformans var JEC21	NC_006694	(Intron)	2.12	2.18	3.20	5.03	9.27	16.9	—	—	—
Debaryomyces hansenii CBS767	NC_006043		0.671	1.61	4.02	10.7	29.9	82.6	214	472	—
Debaryomyces hansenii CBS767	NC_006043	(Gene)	0.434	1.09	2.73	7.29	20.5	57.0	150	—	—
Debaryomyces hansenii CBS767	NC_006043	(Intergenic)	5.70	4.79	9.89	20.8	45.1	89.9	153	—	—
Debaryomyces hansenii CBS767	NC_006043	(Exon)	0.425	1.07	2.68	7.16	20.1	56.1	148	—	—
Debaryomyces hansenii CBS767	NC_006043	(Intron)	2.28	2.85	2.92	3.19	—	—	—	—	—
Debaryomyces hansenii CBS767	NC_006044		0.717	1.69	4.19	11.1	31.1	85.1	217	466	—
Debaryomyces hansenii CBS767	NC_006044	(Gene)	0.471	1.17	2.89	7.69	21.6	60.2	159	—	—

next

			L_e (kb)								
Category	SN	\ k	2	3	4	5	6	7	8	9	10
Debaryomyces hansenii CBS767	NC_006044 (Intergenic)		5.12	3.97	7.93	16.7	35.1	65.6	110	—	—
Debaryomyces hansenii CBS767	NC_006044 (Exon)		0.460	1.14	2.82	7.52	21.1	58.8	154	—	—
Debaryomyces hansenii CBS767	NC_006044 (Intron)		2.48	3.71	4.63	5.99	—	—	—	—	—
Debaryomyces hansenii CBS767	NC_006045		0.679	1.61	4.03	10.7	30.0	83.7	221	509	—
Debaryomyces hansenii CBS767	NC_006045 (Gene)		0.438	1.09	2.73	7.33	20.5	57.8	155	367	—
Debaryomyces hansenii CBS767	NC_006045 (Intergenic)		5.47	4.48	9.16	19.9	43.8	89.1	160	—	—
Debaryomyces hansenii CBS767	NC_006045 (Exon)		0.440	1.09	2.74	7.32	20.5	57.5	154	361	—
Debaryomyces hansenii CBS767	NC_006045 (Intron)		0.715	0.972	1.38	—	—	—	—	—	—
Debaryomyces hansenii CBS767	NC_006046		0.693	1.64	4.07	10.7	30.0	83.6	222	512	—
Debaryomyces hansenii CBS767	NC_006046 (Gene)		0.453	1.12	2.80	7.47	21.0	59.1	159	374	—
Debaryomyces hansenii CBS767	NC_006046 (Intergenic)		6.39	4.54	9.22	19.3	41.7	85.5	156	—	—
Debaryomyces hansenii CBS767	NC_006046 (Exon)		0.451	1.12	2.78	7.39	20.7	58.2	156	366	—
Debaryomyces hansenii CBS767	NC_006046 (Intron)		1.50	2.21	3.06	4.19	—	—	—	—	—
Debaryomyces hansenii CBS767	NC_006047		0.670	1.61	4.08	10.8	30.2	84.7	228	551	—
Debaryomyces hansenii CBS767	NC_006047 (Gene)		0.455	1.14	2.88	7.64	21.3	60.0	164	403	—
Debaryomyces hansenii CBS767	NC_006047 (Intergenic)		4.91	4.18	8.58	18.7	41.9	88.5	168	—	—
Debaryomyces hansenii CBS767	NC_006047 (Exon)		0.451	1.13	2.85	7.55	21.0	59.2	161	392	—
Debaryomyces hansenii CBS767	NC_006047 (Intron)		1.35	2.40	3.83	5.23	—	—	—	—	—
Debaryomyces hansenii CBS767	NC_006048		0.682	1.63	4.06	10.7	30.1	84.9	232	572	—
Debaryomyces hansenii CBS767	NC_006048 (Gene)		0.449	1.12	2.82	7.50	21.1	60.0	166	421	—
Debaryomyces hansenii CBS767	NC_006048 (Intergenic)		5.84	4.46	9.08	19.3	42.0	88.5	168	—	—
Debaryomyces hansenii CBS767	NC_006048 (Exon)		0.439	1.09	2.75	7.31	20.6	58.2	161	404	—
Debaryomyces hansenii CBS767	NC_006048 (Intron)		1.70	2.93	4.29	5.87	—	—	—	—	—
Debaryomyces hansenii CBS767	NC_006049		0.654	1.56	3.89	10.2	28.6	80.2	218	534	—
Debaryomyces hansenii CBS767	NC_006049 (Gene)		0.442	1.10	2.75	7.29	20.4	57.7	159	396	—
Debaryomyces hansenii CBS767	NC_006049 (Intergenic)		6.97	4.34	8.62	17.4	38.0	81.3	157	—	—
Debaryomyces hansenii CBS767	NC_006049 (Exon)		0.439	1.09	2.73	7.23	20.2	57.0	156	388	—
Debaryomyces hansenii CBS767	NC_006049 (Intron)		2.45	2.62	3.76	4.99	—	—	—	—	—
Encephalitozoon cuniculi	NC_003229		0.264	0.576	1.40	3.82	10.8	29.3	—	—	—
Encephalitozoon cuniculi	NC_003229 (Gene)		0.257	0.549	1.31	3.56	10.0	26.8	—	—	—
Encephalitozoon cuniculi	NC_003229 (Intergenic)		0.282	0.696	1.76	4.33	9.22	—	—	—	—
Encephalitozoon cuniculi	NC_003230		0.282	0.611	1.48	4.03	11.4	30.9	—	—	—
Encephalitozoon cuniculi	NC_003230 (Gene)		0.272	0.578	1.38	3.73	10.4	27.9	—	—	—
Encephalitozoon cuniculi	NC_003230 (Intergenic)		0.321	0.766	1.91	4.67	10.2	—	—	—	—

next

Category	SN \ k	L_e (kb) 2	3	4	5	6	7	8	9	10
Encephalitozoon cuniculi	NC_003231	0.261	0.579	1.43	3.91	11.1	30.5	—	—	—
Encephalitozoon cuniculi	NC_003231 (Gene)	0.247	0.541	1.31	3.57	10.1	27.3	—	—	—
Encephalitozoon cuniculi	NC_003231 (Intergenic)	0.204	0.533	1.37	3.48	8.21	—	—	—	—
Encephalitozoon cuniculi	NC_003232	0.245	0.547	1.36	3.74	10.6	29.1	—	—	—
Encephalitozoon cuniculi	NC_003232 (Gene)	0.239	0.523	1.28	3.51	9.89	26.8	—	—	—
Encephalitozoon cuniculi	NC_003232 (Intergenic)	0.243	0.601	1.55	3.95	8.97	—	—	—	—
Encephalitozoon cuniculi	NC_003233	0.250	0.546	1.32	3.60	10.2	28.0	—	—	—
Encephalitozoon cuniculi	NC_003233 (Gene)	0.250	0.537	1.28	3.46	9.73	26.3	—	—	—
Encephalitozoon cuniculi	NC_003233 (Intergenic)	0.223	0.527	1.33	3.40	8.00	—	—	—	—
Encephalitozoon cuniculi	NC_003234	0.230	0.519	1.29	3.56	10.2	28.3	—	—	—
Encephalitozoon cuniculi	NC_003234 (Gene)	0.226	0.501	1.23	3.37	9.59	26.3	—	—	—
Encephalitozoon cuniculi	NC_003234 (Intergenic)	0.247	0.611	1.57	3.89	8.51	—	—	—	—
Encephalitozoon cuniculi	NC_003235	0.253	0.565	1.38	3.79	10.9	30.2	—	—	—
Encephalitozoon cuniculi	NC_003235 (Gene)	0.247	0.540	1.29	3.54	10.0	27.5	—	—	—
Encephalitozoon cuniculi	NC_003235 (Intergenic)	0.261	0.636	1.64	4.29	9.99	—	—	—	—
Encephalitozoon cuniculi	NC_003236	0.253	0.562	1.38	3.79	10.9	30.3	75.6	—	—
Encephalitozoon cuniculi	NC_003236 (Gene)	0.250	0.545	1.32	3.61	10.3	28.4	—	—	—
Encephalitozoon cuniculi	NC_003236 (Intergenic)	0.249	0.608	1.56	3.97	9.05	—	—	—	—
Encephalitozoon cuniculi	NC_003237	0.255	0.570	1.40	3.84	11.0	30.6	76.2	—	—
Encephalitozoon cuniculi	NC_003237 (Gene)	0.255	0.555	1.34	3.64	10.3	28.4	—	—	—
Encephalitozoon cuniculi	NC_003237 (Intergenic)	0.236	0.587	1.53	4.01	9.69	—	—	—	—
Encephalitozoon cuniculi	NC_003238	0.259	0.575	1.42	3.92	11.3	31.5	—	—	—
Encephalitozoon cuniculi	NC_003238 (Gene)	0.255	0.553	1.34	3.69	10.5	29.1	—	—	—
Encephalitozoon cuniculi	NC_003238 (Intergenic)	0.278	0.684	1.76	4.52	10.2	—	—	—	—
Encephalitozoon cuniculi	NC_003242	0.262	0.563	1.36	3.69	10.3	27.6	—	—	—
Encephalitozoon cuniculi	NC_003242 (Gene)	0.251	0.552	1.32	3.57	9.95	26.4	—	—	—
Encephalitozoon cuniculi	NC_003242 (Intergenic)	0.277	0.494	1.10	2.61	5.93	—	—	—	—
Eremothecium gossypii	NC_005782	0.927	2.10	4.47	11.9	33.4	88.7	212	—	—
Eremothecium gossypii	NC_005782 (Gene)	0.781	1.78	3.64	9.52	26.5	69.5	166	—	—
Eremothecium gossypii	NC_005782 (Intergenic)	1.02	2.28	5.78	15.0	34.5	66.0	—	—	—
Eremothecium gossypii	NC_005782 (Exon)	0.772	1.76	3.59	9.39	26.1	68.6	164	—	—
Eremothecium gossypii	NC_005782 (Intron)	0.195	0.408	0.617	—	—	—	—	—	—
Eremothecium gossypii	NC_005783	0.950	2.12	4.64	12.4	35.2	95.1	234	—	—
Eremothecium gossypii	NC_005783 (Gene)	0.811	1.84	3.89	10.3	29.1	78.1	191	—	—

next

Category	SN \ k	L_e (kb) 2	3	4	5	6	7	8	9	10
Eremothecium gossypii	NC_005783 (Intergenic)	1.16	2.45	5.94	14.9	34.8	68.6	—	—	—
Eremothecium gossypii	NC_005783 (Exon)	0.805	1.82	3.86	10.3	28.9	77.4	189	—	—
Eremothecium gossypii	NC_005783 (Intron)	0.649	0.962	1.18	—	—	—	—	—	—
Eremothecium gossypii	NC_005784	0.856	1.98	4.20	11.1	31.2	83.1	208	—	—
Eremothecium gossypii	NC_005784 (Gene)	0.749	1.75	3.53	9.17	25.5	67.0	167	—	—
Eremothecium gossypii	NC_005784 (Intergenic)	0.945	2.07	5.28	13.5	31.9	65.2	—	—	—
Eremothecium gossypii	NC_005784 (Exon)	0.743	1.74	3.50	9.10	25.3	66.5	166	—	—
Eremothecium gossypii	NC_005784 (Intron)	0.207	0.469	0.830	—	—	—	—	—	—
Eremothecium gossypii	NC_005785	0.973	2.19	4.66	12.5	35.6	98.7	263	588	—
Eremothecium gossypii	NC_005785 (Gene)	0.834	1.89	3.87	10.2	29.0	79.2	209	467	—
Eremothecium gossypii	NC_005785 (Intergenic)	1.10	2.39	5.99	15.7	39.3	88.0	158	—	—
Eremothecium gossypii	NC_005785 (Exon)	0.829	1.88	3.84	10.1	28.7	78.5	207	464	—
Eremothecium gossypii	NC_005785 (Intron)	0.332	0.743	1.34	2.19	—	—	—	—	—
Eremothecium gossypii	NC_005786	0.977	2.21	4.80	12.9	36.9	103	274	613	—
Eremothecium gossypii	NC_005786 (Gene)	0.825	1.91	4.03	10.7	30.5	83.9	222	495	—
Eremothecium gossypii	NC_005786 (Intergenic)	1.11	2.32	5.82	15.2	38.5	87.2	159	—	—
Eremothecium gossypii	NC_005786 (Exon)	0.817	1.89	3.99	10.6	30.2	83.0	220	490	—
Eremothecium gossypii	NC_005786 (Intron)	0.397	0.725	1.14	1.72	—	—	—	—	—
Eremothecium gossypii	NC_005787	0.960	2.18	4.64	12.4	35.5	98.6	267	630	—
Eremothecium gossypii	NC_005787 (Gene)	0.827	1.89	3.87	10.2	29.1	79.6	214	504	—
Eremothecium gossypii	NC_005787 (Intergenic)	1.07	2.39	6.01	16.0	40.6	93.3	175	—	—
Eremothecium gossypii	NC_005787 (Exon)	0.817	1.87	3.83	10.1	28.7	78.6	212	498	—
Eremothecium gossypii	NC_005787 (Intron)	0.495	0.945	1.72	2.50	—	—	—	—	—
Eremothecium gossypii	NC_005788	0.946	2.13	4.54	12.1	34.5	95.0	253	573	—
Eremothecium gossypii	NC_005788 (Gene)	0.809	1.84	3.81	10.0	28.5	77.7	206	465	—
Eremothecium gossypii	NC_005788 (Intergenic)	1.07	2.30	5.76	15.0	37.2	82.9	149	—	—
Eremothecium gossypii	NC_005788 (Exon)	0.799	1.82	3.76	9.91	28.2	76.7	203	460	—
Eremothecium gossypii	NC_005788 (Intron)	0.293	0.669	1.15	—	—	—	—	—	—
Kluyveromyces lactis NRRL Y-1140	NC_006037	0.537	1.32	3.29	8.77	24.6	67.8	175	385	—
Kluyveromyces lactis NRRL Y-1140	NC_006037 (Gene)	0.446	1.10	2.65	7.01	19.7	54.9	143	—	—
Kluyveromyces lactis NRRL Y-1140	NC_006037 (Intergenic)	0.833	1.56	3.66	8.53	19.4	41.3	80.7	—	—
Kluyveromyces lactis NRRL Y-1140	NC_006037 (Exon)	0.443	1.09	2.63	6.94	19.5	54.4	142	—	—
Kluyveromyces lactis NRRL Y-1140	NC_006037 (Intron)	0.504	1.09	2.12	3.35	—	—	—	—	—
Kluyveromyces lactis NRRL Y-1140	NC_006038	0.492	1.23	3.10	8.33	23.5	66.1	177	412	—

next

Category	SN \ k	L_e (kb)								
		2	3	4	5	6	7	8	9	10
Kluyveromyces lactis NRRL Y-1140	NC_006038 (Gene)	0.408	1.02	2.52	6.71	19.0	54.0	146	—	—
Kluyveromyces lactis NRRL Y-1140	NC_006038 (Intergenic)	0.778	1.48	3.51	8.38	19.6	42.9	87.0	—	—
Kluyveromyces lactis NRRL Y-1140	NC_006038 (Exon)	0.405	1.01	2.49	6.64	18.8	53.4	145	—	—
Kluyveromyces lactis NRRL Y-1140	NC_006038 (Intron)	0.612	1.20	2.18	3.54	—	—	—	—	—
Kluyveromyces lactis NRRL Y-1140	NC_006039	0.451	1.13	2.89	7.86	22.5	64.0	176	432	—
Kluyveromyces lactis NRRL Y-1140	NC_006039 (Gene)	0.377	0.969	2.44	6.58	18.9	54.4	152	375	—
Kluyveromyces lactis NRRL Y-1140	NC_006039 (Intergenic)	0.702	1.30	3.06	7.35	17.4	38.6	80.5	—	—
Kluyveromyces lactis NRRL Y-1140	NC_006039 (Exon)	0.376	0.963	2.42	6.53	18.7	54.0	151	372	—
Kluyveromyces lactis NRRL Y-1140	NC_006039 (Intron)	0.678	1.20	2.23	3.50	—	—	—	—	—
Kluyveromyces lactis NRRL Y-1140	NC_006040	0.509	1.27	3.19	8.57	24.3	68.4	185	443	—
Kluyveromyces lactis NRRL Y-1140	NC_006040 (Gene)	0.416	1.05	2.57	6.85	19.4	55.5	153	371	—
Kluyveromyces lactis NRRL Y-1140	NC_006040 (Intergenic)	0.832	1.60	3.82	9.06	21.2	46.7	95.4	—	—
Kluyveromyces lactis NRRL Y-1140	NC_006040 (Exon)	0.414	1.04	2.55	6.79	19.3	55.0	152	368	—
Kluyveromyces lactis NRRL Y-1140	NC_006040 (Intron)	0.342	0.918	1.71	2.55	—	—	—	—	—
Kluyveromyces lactis NRRL Y-1140	NC_006041	0.491	1.22	3.08	8.31	23.6	67.2	185	465	—
Kluyveromyces lactis NRRL Y-1140	NC_006041 (Gene)	0.414	1.04	2.54	6.80	19.4	55.7	156	397	—
Kluyveromyces lactis NRRL Y-1140	NC_006041 (Intergenic)	0.727	1.40	3.32	7.94	18.6	41.5	88.1	—	—
Kluyveromyces lactis NRRL Y-1140	NC_006041 (Exon)	0.411	1.03	2.52	6.74	19.2	55.2	155	394	—
Kluyveromyces lactis NRRL Y-1140	NC_006041 (Intron)	0.938	1.75	3.18	5.09	—	—	—	—	—
Kluyveromyces lactis NRRL Y-1140	NC_006042	0.463	1.16	2.94	7.96	22.6	64.3	178	455	—
Kluyveromyces lactis NRRL Y-1140	NC_006042 (Gene)	0.384	0.980	2.44	6.56	18.8	54.6	156	411	—
Kluyveromyces lactis NRRL Y-1140	NC_006042 (Intergenic)	0.716	1.32	3.04	7.15	16.5	36.4	77.6	—	—
Kluyveromyces lactis NRRL Y-1140	NC_006042 (Exon)	0.383	0.976	2.43	6.53	18.7	54.3	155	409	—
Kluyveromyces lactis NRRL Y-1140	NC_006042 (Intron)	0.451	1.09	2.20	3.44	—	—	—	—	—
Saccharomyces cerevisiae	NC_001133	0.477	1.11	2.69	6.98	18.2	43.4	—	—	—
Saccharomyces cerevisiae	NC_001133 (Gene)	0.419	1.04	2.55	6.72	17.7	41.2	—	—	—
Saccharomyces cerevisiae	NC_001133 (Intergenic)	0.547	0.902	1.86	4.14	9.08	17.8	—	—	—
Saccharomyces cerevisiae	NC_001134	0.443	1.05	2.66	7.14	19.8	53.2	132	—	—
Saccharomyces cerevisiae	NC_001134 (Gene)	0.381	0.976	2.54	6.96	19.9	55.5	141	—	—
Saccharomyces cerevisiae	NC_001134 (Intergenic)	0.621	0.836	1.67	3.59	7.83	16.3	—	—	—
Saccharomyces cerevisiae	NC_001135	0.498	1.16	2.86	7.50	20.1	49.5	104	—	—
Saccharomyces cerevisiae	NC_001135 (Gene)	0.398	1.02	2.59	7.00	19.4	49.2	—	—	—
Saccharomyces cerevisiae	NC_001135 (Intergenic)	0.817	1.03	2.00	4.22	8.90	16.9	—	—	—
Saccharomyces cerevisiae	NC_001136	0.451	1.07	2.69	7.25	20.4	56.6	150	349	—

next

			L_e (kb)								
Category	SN	$\backslash$ k	2	3	4	5	6	7	8	9	10
Saccharomyces cerevisiae	NC_001136 (Gene)		0.386	0.984	2.52	6.91	19.9	56.9	155	364	—
Saccharomyces cerevisiae	NC_001136 (Intergenic)		0.640	0.941	1.94	4.35	9.93	21.6	44.5	—	—
Saccharomyces cerevisiae	NC_001137		0.496	1.18	2.92	7.79	21.3	55.7	131	—	—
Saccharomyces cerevisiae	NC_001137 (Gene)		0.416	1.06	2.69	7.31	20.6	55.4	130	—	—
Saccharomyces cerevisiae	NC_001137 (Intergenic)		0.665	1.04	2.12	4.70	10.4	21.5	—	—	—
Saccharomyces cerevisiae	NC_001138		0.470	1.11	2.74	7.22	19.3	47.4	99.4	—	—
Saccharomyces cerevisiae	NC_001138 (Gene)		0.377	0.970	2.48	6.70	18.4	45.6	—	—	—
Saccharomyces cerevisiae	NC_001138 (Intergenic)		0.721	1.00	1.95	4.18	9.02	17.7	—	—	—
Saccharomyces cerevisiae	NC_001139		0.461	1.09	2.74	7.35	20.4	55.7	142	310	—
Saccharomyces cerevisiae	NC_001139 (Gene)		0.377	0.961	2.49	6.82	19.6	55.7	148	—	—
Saccharomyces cerevisiae	NC_001139 (Intergenic)		0.753	1.01	2.01	4.36	9.61	20.0	39.7	—	—
Saccharomyces cerevisiae	NC_001140		0.474	1.11	2.77	7.44	20.5	53.8	125	—	—
Saccharomyces cerevisiae	NC_001140 (Gene)		0.406	1.03	2.66	7.32	20.8	56.3	133	—	—
Saccharomyces cerevisiae	NC_001140 (Intergenic)		0.643	0.907	1.82	4.03	8.88	17.6	—	—	—
Saccharomyces cerevisiae	NC_001141		0.466	1.10	2.75	7.40	20.3	52.2	118	—	—
Saccharomyces cerevisiae	NC_001141 (Gene)		0.402	1.02	2.60	7.12	19.9	51.9	114	—	—
Saccharomyces cerevisiae	NC_001141 (Intergenic)		0.632	0.928	1.89	4.18	9.10	18.3	—	—	—
Saccharomyces cerevisiae	NC_001142		0.480	1.15	2.91	7.86	21.9	59.0	146	—	—
Saccharomyces cerevisiae	NC_001142 (Gene)		0.396	1.02	2.65	7.29	20.8	58.0	146	—	—
Saccharomyces cerevisiae	NC_001142 (Intergenic)		0.829	1.06	2.09	4.47	9.69	19.9	—	—	—
Saccharomyces cerevisiae	NC_001143		0.442	1.06	2.67	7.16	19.9	53.6	132	—	—
Saccharomyces cerevisiae	NC_001143 (Gene)		0.363	0.938	2.44	6.67	19.0	52.4	129	—	—
Saccharomyces cerevisiae	NC_001143 (Intergenic)		0.740	0.980	1.95	4.23	9.36	19.9	—	—	—
Saccharomyces cerevisiae	NC_001144		0.465	1.12	2.83	7.62	21.3	58.4	148	318	—
Saccharomyces cerevisiae	NC_001144 (Gene)		0.397	1.01	2.62	7.17	20.6	58.4	153	—	—
Saccharomyces cerevisiae	NC_001144 (Intergenic)		0.688	1.03	2.15	4.71	10.4	21.3	41.1	—	—
Saccharomyces cerevisiae	NC_001145		0.459	1.09	2.75	7.39	20.7	56.6	143	—	—
Saccharomyces cerevisiae	NC_001145 (Gene)		0.392	0.999	2.58	7.06	20.2	56.8	147	—	—
Saccharomyces cerevisiae	NC_001145 (Intergenic)		0.689	0.893	1.78	3.89	8.68	18.4	—	—	—
Saccharomyces cerevisiae	NC_001146		0.481	1.12	2.80	7.55	21.0	57.1	142	—	—
Saccharomyces cerevisiae	NC_001146 (Gene)		0.417	1.04	2.66	7.29	20.9	58.5	148	—	—
Saccharomyces cerevisiae	NC_001146 (Intergenic)		0.644	0.909	1.89	4.20	9.32	19.7	—	—	—
Saccharomyces cerevisiae	NC_001147		0.465	1.09	2.74	7.37	20.6	56.6	147	326	—
Saccharomyces cerevisiae	NC_001147 (Gene)		0.390	0.996	2.58	7.09	20.4	58.0	153	—	—

next

Category	SN \ k	L_e (kb) 2	3	4	5	6	7	8	9	10
Saccharomyces cerevisiae	NC_001147 (Intergenic)	0.662	0.936	1.88	4.21	9.53	20.8	43.2	—	—
Saccharomyces cerevisiae	NC_001148	0.454	1.09	2.76	7.45	20.9	57.4	147	—	—
Saccharomyces cerevisiae	NC_001148 (Gene)	0.387	0.993	2.58	7.12	20.5	57.8	149	—	—
Saccharomyces cerevisiae	NC_001148 (Intergenic)	0.652	0.962	1.98	4.40	9.94	21.4	—	—	—
Schizosaccharomyces pombe	NC_003421	0.347	0.852	2.29	6.35	17.9	50.4	138	347	—
Schizosaccharomyces pombe	NC_003421 (Gene)	0.335	0.867	2.35	6.71	19.8	58.0	164	406	—
Schizosaccharomyces pombe	NC_003421 (Intergenic)	0.339	0.745	1.89	4.81	12.1	30.2	72.2	—	—
Schizosaccharomyces pombe	NC_003421 (Exon)	0.321	0.834	2.25	6.44	19.0	55.9	158	391	—
Schizosaccharomyces pombe	NC_003421 (Intron)	0.302	0.671	1.35	2.57	4.84	—	—	—	—
Schizosaccharomyces pombe	NC_003423	0.376	0.928	2.50	7.01	20.0	57.1	160	423	972
Schizosaccharomyces pombe	NC_003423 (Gene)	0.346	0.895	2.42	6.94	20.5	60.5	175	461	—
Schizosaccharomyces pombe	NC_003423 (Intergenic)	0.384	0.845	2.14	5.43	13.7	33.9	82.2	188	—
Schizosaccharomyces pombe	NC_003423 (Exon)	0.327	0.847	2.29	6.56	19.4	57.3	166	435	—
Schizosaccharomyces pombe	NC_003423 (Intron)	0.333	0.700	1.36	2.61	5.01	9.53	—	—	—
Schizosaccharomyces pombe	NC_003424	0.367	0.910	2.46	6.89	19.8	56.6	160	435	1055
Schizosaccharomyces pombe	NC_003424 (Gene)	0.362	0.908	2.46	6.94	20.1	58.3	168	463	1130
Schizosaccharomyces pombe	NC_003424 (Intergenic)	0.388	0.843	2.11	5.29	13.0	30.7	68.0	—	—
Schizosaccharomyces pombe	NC_003424 (Exon)	0.325	0.843	2.28	6.55	19.4	58.0	173	488	—
Schizosaccharomyces pombe	NC_003424 (Intron)	0.340	0.716	1.42	2.74	5.35	10.4	—	—	—
Yarrowia lipolytica CLIB99	NC_006067	0.855	1.50	3.30	8.77	24.4	66.3	175	417	—
Yarrowia lipolytica CLIB99	NC_006067 (Gene)	0.531	1.06	2.20	5.73	16.0	43.6	117	—	—
Yarrowia lipolytica CLIB99	NC_006067 (Intergenic)	1.12	1.61	3.54	8.87	22.4	54.1	124	256	—
Yarrowia lipolytica CLIB99	NC_006067 (Exon)	0.522	1.04	2.14	5.54	15.4	42.0	112	—	—
Yarrowia lipolytica CLIB99	NC_006067 (Intron)	0.605	1.15	2.37	4.92	8.77	—	—	—	—
Yarrowia lipolytica CLIB99	NC_006068	0.989	1.65	3.51	9.29	25.9	70.8	192	490	—
Yarrowia lipolytica CLIB99	NC_006068 (Gene)	0.514	1.01	2.06	5.30	14.7	40.4	111	287	—
Yarrowia lipolytica CLIB99	NC_006068 (Intergenic)	1.38	1.82	4.07	10.3	26.0	63.4	150	325	—
Yarrowia lipolytica CLIB99	NC_006068 (Exon)	0.497	0.983	2.00	5.11	14.1	38.8	107	274	—
Yarrowia lipolytica CLIB99	NC_006068 (Intron)	0.588	0.981	2.16	4.84	9.68	—	—	—	—
Yarrowia lipolytica CLIB99	NC_006069	0.847	1.50	3.33	8.92	25.1	69.3	189	475	—
Yarrowia lipolytica CLIB99	NC_006069 (Gene)	0.539	1.06	2.18	5.64	15.7	43.1	118	295	—
Yarrowia lipolytica CLIB99	NC_006069 (Intergenic)	1.05	1.59	3.63	9.33	24.4	61.6	149	327	—
Yarrowia lipolytica CLIB99	NC_006069 (Exon)	0.512	1.02	2.10	5.42	15.0	41.4	114	287	—
Yarrowia lipolytica CLIB99	NC_006069 (Intron)	0.588	0.992	2.18	4.71	9.21	—	—	—	—

next

Category	SN \ k	L_e (kb) 2	3	4	5	6	7	8	9	10
Yarrowia lipolytica CLIB99	NC_006070	0.918	1.55	3.34	8.86	24.7	67.9	185	477	—
Yarrowia lipolytica CLIB99	NC_006070 (Gene)	0.531	1.05	2.14	5.49	15.1	41.5	114	299	—
Yarrowia lipolytica CLIB99	NC_006070 (Intergenic)	1.23	1.66	3.71	9.43	24.2	60.5	145	321	—
Yarrowia lipolytica CLIB99	NC_006070 (Exon)	0.520	1.03	2.08	5.34	14.7	40.2	111	289	—
Yarrowia lipolytica CLIB99	NC_006070 (Intron)	0.616	0.996	2.17	4.71	9.01	—	—	—	—
Yarrowia lipolytica CLIB99	NC_006071	1.06	1.71	3.55	9.32	26.0	71.5	197	517	1171
Yarrowia lipolytica CLIB99	NC_006071 (Gene)	0.518	1.02	2.07	5.33	14.8	41.3	117	315	—
Yarrowia lipolytica CLIB99	NC_006071 (Intergenic)	1.50	1.92	4.21	10.5	26.4	64.1	151	335	—
Yarrowia lipolytica CLIB99	NC_006071 (Exon)	0.503	0.992	2.02	5.19	14.4	40.2	113	307	—
Yarrowia lipolytica CLIB99	NC_006071 (Intron)	0.748	1.06	2.18	4.51	8.04	—	—	—	—
Yarrowia lipolytica CLIB99	NC_006072	1.03	1.68	3.53	9.27	25.8	70.6	193	502	—
Yarrowia lipolytica CLIB99	NC_006072 (Gene)	0.535	1.04	2.09	5.35	14.8	40.7	113	302	—
Yarrowia lipolytica CLIB99	NC_006072 (Intergenic)	1.41	1.84	4.04	10.1	25.4	62.1	148	329	—
Yarrowia lipolytica CLIB99	NC_006072 (Exon)	0.516	1.01	2.02	5.16	14.2	39.1	109	291	—
Yarrowia lipolytica CLIB99	NC_006072 (Intron)	0.615	0.980	2.13	4.75	9.53	—	—	—	—
P. falciparum (14)										
Plasmodium falciparum	CHR_1	1.64	0.319	0.395	0.531	0.759	1.04	1.40	—	—
Plasmodium falciparum	CHR_1 (Gene)	0.353	0.553	0.939	1.85	3.84	7.73	15.1	—	—
Plasmodium falciparum	CHR_1 (Intergenic)	0.684	0.151	0.168	0.207	0.275	0.358	0.466	—	—
Plasmodium falciparum	CHR_1 (Exon)	0.336	0.541	0.925	1.83	3.81	7.69	15.1	—	—
Plasmodium falciparum	CHR_1 (Intron)	0.195	0.055	0.070	0.083	0.105	—	—	—	—
Plasmodium falciparum	CHR_2	1.76	0.297	0.384	0.525	0.749	1.03	1.39	—	—
Plasmodium falciparum	CHR_2 (Gene)	0.747	0.533	0.849	1.47	2.61	4.33	6.75	—	—
Plasmodium falciparum	CHR_2 (Intergenic)	0.819	0.143	0.164	0.205	0.274	0.361	0.472	—	—
Plasmodium falciparum	CHR_2 (Exon)	0.523	0.670	1.10	2.16	4.49	9.24	18.6	—	—
Plasmodium falciparum	CHR_2 (Intron)	0.148	0.056	0.070	0.084	0.105	—	—	—	—
Plasmodium falciparum	CHR_3	1.53	0.307	0.395	0.532	0.756	1.04	1.39	1.82	—
Plasmodium falciparum	CHR_3 (Gene)	0.570	0.754	1.14	2.10	4.16	8.13	15.5	—	—
Plasmodium falciparum	CHR_3 (Intergenic)	0.601	0.114	0.130	0.160	0.208	0.269	0.348	—	—
Plasmodium falciparum	CHR_3 (Exon)	0.564	0.752	1.14	2.10	4.17	8.14	15.6	—	—
Plasmodium falciparum	CHR_3 (Intron)	0.188	0.052	0.062	0.072	0.090	—	—	—	—
Plasmodium falciparum	CHR_4	1.28	0.304	0.405	0.560	0.809	1.12	1.52	2.00	—
Plasmodium falciparum	CHR_4 (Gene)	0.528	0.640	1.02	1.92	3.83	7.39	13.9	—	—
Plasmodium falciparum	CHR_4 (Intergenic)	0.509	0.118	0.140	0.175	0.231	0.299	0.388	—	—

next

			L_e (kb)							
Category	SN \ k	2	3	4	5	6	7	8	9	10
Plasmodium falciparum	CHR_4 (Exon)	0.494	0.681	1.08	2.06	4.17	8.25	16.0	—	—
Plasmodium falciparum	CHR_4 (Intron)	0.154	0.049	0.059	0.070	0.090	—	—	—	—
Plasmodium falciparum	CHR_5	1.21	0.272	0.361	0.489	0.688	0.933	1.24	1.60	—
Plasmodium falciparum	CHR_5 (Gene)	0.465	0.685	1.09	2.04	4.14	8.21	16.0	—	—
Plasmodium falciparum	CHR_5 (Intergenic)	0.561	0.094	0.111	0.136	0.175	0.224	0.286	—	—
Plasmodium falciparum	CHR_5 (Exon)	0.460	0.682	1.08	2.04	4.14	8.20	16.0	—	—
Plasmodium falciparum	CHR_5 (Intron)	0.138	0.051	0.063	0.074	0.091	0.113	—	—	—
Plasmodium falciparum	CHR_6	1.24	0.295	0.391	0.536	0.764	1.05	1.40	1.82	—
Plasmodium falciparum	CHR_6 (Gene)	0.542	0.777	1.15	2.07	4.05	7.75	14.4	—	—
Plasmodium falciparum	CHR_6 (Intergenic)	0.514	0.100	0.119	0.147	0.193	0.249	0.320	—	—
Plasmodium falciparum	CHR_6 (Exon)	0.530	0.773	1.14	2.06	4.04	7.73	14.4	—	—
Plasmodium falciparum	CHR_6 (Intron)	0.142	0.050	0.061	0.073	0.092	0.115	—	—	—
Plasmodium falciparum	CHR_7	1.19	0.315	0.425	0.597	0.867	1.21	1.65	2.17	—
Plasmodium falciparum	CHR_7 (Gene)	0.501	0.670	1.05	1.96	3.91	7.61	14.4	—	—
Plasmodium falciparum	CHR_7 (Intergenic)	0.636	0.109	0.129	0.160	0.209	0.271	0.350	—	—
Plasmodium falciparum	CHR_7 (Exon)	0.481	0.685	1.06	2.00	4.00	7.82	14.9	—	—
Plasmodium falciparum	CHR_7 (Intron)	0.163	0.051	0.063	0.074	0.095	0.119	—	—	—
Plasmodium falciparum	CHR_8	1.36	0.270	0.355	0.482	0.680	0.926	1.24	1.61	—
Plasmodium falciparum	CHR_8 (Gene)	0.565	0.722	1.09	1.99	3.89	7.40	13.7	—	—
Plasmodium falciparum	CHR_8 (Intergenic)	0.672	0.098	0.116	0.143	0.186	0.240	0.309	—	—
Plasmodium falciparum	CHR_8 (Exon)	0.539	0.734	1.10	2.01	3.94	7.52	14.0	—	—
Plasmodium falciparum	CHR_8 (Intron)	0.205	0.055	0.067	0.079	0.100	0.123	—	—	—
Plasmodium falciparum	CHR_9	1.46	0.261	0.336	0.449	0.628	0.846	1.12	1.44	—
Plasmodium falciparum	CHR_9 (Gene)	0.574	0.758	1.20	2.27	4.58	9.09	17.5	—	—
Plasmodium falciparum	CHR_9 (Intergenic)	0.564	0.102	0.119	0.145	0.189	0.242	0.310	—	—
Plasmodium falciparum	CHR_9 (Exon)	0.566	0.758	1.20	2.27	4.60	9.15	17.7	—	—
Plasmodium falciparum	CHR_9 (Intron)	0.176	0.052	0.064	0.074	0.091	0.111	—	—	—
Plasmodium falciparum	CHR_10	1.82	0.292	0.374	0.504	0.707	0.960	1.28	1.66	—
Plasmodium falciparum	CHR_10 (Gene)	0.801	0.569	0.857	1.40	2.38	3.78	5.64	—	—
Plasmodium falciparum	CHR_10 (Intergenic)	0.850	0.130	0.151	0.189	0.249	0.326	0.426	—	—
Plasmodium falciparum	CHR_10 (Exon)	0.538	0.742	1.13	2.10	4.16	8.14	15.5	—	—
Plasmodium falciparum	CHR_10 (Intron)	0.153	0.055	0.068	0.080	0.100	0.123	—	—	—
Plasmodium falciparum	CHR_11	1.34	0.264	0.348	0.474	0.670	0.910	1.21	1.56	—
Plasmodium falciparum	CHR_11 (Gene)	0.703	0.587	0.901	1.51	2.65	4.33	6.64	9.61	—

next

Category	SN \ k	L_e (kb) 2	3	4	5	6	7	8	9	10
Plasmodium falciparum	CHR_11 (Intergenic)	0.801	0.108	0.128	0.162	0.213	0.279	0.362	—	—
Plasmodium falciparum	CHR_11 (Exon)	0.527	0.733	1.12	2.06	4.08	7.93	15.2	—	—
Plasmodium falciparum	CHR_11 (Intron)	0.149	0.054	0.065	0.077	0.095	0.116	—	—	—
Plasmodium falciparum	CHR_12	1.25	0.275	0.363	0.493	0.697	0.948	1.26	1.63	—
Plasmodium falciparum	CHR_12 (Gene)	0.683	0.595	0.932	1.58	2.79	4.62	7.23	10.6	—
Plasmodium falciparum	CHR_12 (Intergenic)	0.688	0.105	0.124	0.154	0.202	0.263	0.339	—	—
Plasmodium falciparum	CHR_12 (Exon)	0.527	0.756	1.19	2.23	4.44	8.74	16.8	31.2	—
Plasmodium falciparum	CHR_12 (Intron)	0.157	0.052	0.063	0.074	0.093	0.114	—	—	—
Plasmodium falciparum	CHR_13	1.30	0.286	0.379	0.521	0.741	1.02	1.36	1.77	—
Plasmodium falciparum	CHR_13 (Gene)	0.575	0.786	1.22	2.29	4.61	9.22	18.0	34.3	
Plasmodium falciparum	CHR_13 (Intergenic)	0.632	0.094	0.110	0.136	0.176	0.226	0.291	0.366	—
Plasmodium falciparum	CHR_13 (Exon)	0.571	0.785	1.22	2.29	4.61	9.23	18.0	34.3	—
Plasmodium falciparum	CHR_13 (Intron)	0.167	0.053	0.064	0.075	0.094	0.115	—	—	—
Plasmodium falciparum	CHR_14	1.20	0.264	0.353	0.486	0.693	0.952	1.27	1.65	—
Plasmodium falciparum	CHR_14 (Gene)	0.725	0.598	0.921	1.56	2.79	4.66	7.29	10.8	—
Plasmodium falciparum	CHR_14 (Intergenic)	0.781	0.100	0.120	0.151	0.199	0.260	0.338	0.430	—
Plasmodium falciparum	CHR_14 (Exon)	0.561	0.757	1.16	2.16	4.29	8.47	16.3	30.6	—
Plasmodium falciparum	CHR_14 (Intron)	0.148	0.053	0.064	0.076	0.095	0.116	—	—	—

Effective Length List 3: Insects (39).

Category	SN \ k	L_e (kb) 2	3	4	5	6	7	8	9	10
Anopheles gambiae	CHR_2	0.349	0.833	2.19	5.88	15.9	40.8	96.9	201	360
Anopheles gambiae	CHR_2 (Gene)	0.359	0.858	2.25	6.07	16.4	42.3	101	211	379
Anopheles gambiae	CHR_2 (Intergenic)	0.346	0.826	2.17	5.83	15.7	40.3	95.5	198	353
Anopheles gambiae	CHR_2 (Exon)	0.362	0.861	2.25	6.02	16.2	41.6	99.2	208	374
Anopheles gambiae	CHR_2 (Intron)	0.359	0.859	2.27	6.12	16.6	42.9	103	214	380
Anopheles gambiae	CHR_3	0.337	0.815	2.17	5.91	16.3	43.1	108	239	460
Anopheles gambiae	CHR_3 (Gene)	0.353	0.851	2.26	6.16	17.0	45.1	113	251	479
Anopheles gambiae	CHR_3 (Intergenic)	0.333	0.806	2.14	5.85	16.1	42.6	106	235	452
Anopheles gambiae	CHR_3 (Exon)	0.365	0.881	2.32	6.30	17.3	45.8	114	249	464

next

		L_e (kb)								
Category	SN \ k	2	3	4	5	6	7	8	9	10
Anopheles gambiae	CHR_3 (Intron)	0.348	0.841	2.24	6.12	16.9	45.0	113	250	477
Anopheles gambiae	CHR_X	0.391	0.865	2.15	5.14	11.7	23.3	41.0	63.1	87.8
Anopheles gambiae	CHR_X (Gene)	0.420	0.924	2.28	5.45	12.4	25.0	44.8	70.3	99.3
Anopheles gambiae	CHR_X (Intergenic)	0.383	0.849	2.11	5.05	11.5	22.8	39.8	60.8	84.2
Anopheles gambiae	CHR_X (Exon)	0.432	0.960	2.38	5.81	13.6	28.1	51.9	83.5	—
Anopheles gambiae	CHR_X (Intron)	0.415	0.912	2.24	5.33	12.0	24.0	42.4	65.6	—
Apis mellifera	CHR_LG1	0.218	0.493	1.22	3.07	7.57	17.5	37.5	73.4	132
Apis mellifera	CHR_LG1 (Gene)	0.235	0.534	1.33	3.37	8.38	19.6	42.5	84.0	152
Apis mellifera	CHR_LG1 (Intergenic)	0.210	0.473	1.17	2.92	7.19	16.5	35.2	68.3	122
Apis mellifera	CHR_LG1 (Exon)	0.512	1.24	2.91	7.84	22.0	60.4	159	—	—
Apis mellifera	CHR_LG1 (Intron)	0.208	0.469	1.15	2.88	7.05	16.1	34.4	66.8	119
Apis mellifera	CHR_LG2	0.223	0.503	1.24	3.13	7.70	17.7	37.6	72.2	128
Apis mellifera	CHR_LG2 (Gene)	0.255	0.567	1.39	3.47	8.41	18.9	39.4	74.1	—
Apis mellifera	CHR_LG2 (Intergenic)	0.212	0.479	1.18	2.99	7.39	17.1	36.5	70.5	125
Apis mellifera	CHR_LG2 (Exon)	0.497	1.23	2.85	7.63	21.3	57.4	143	—	—
Apis mellifera	CHR_LG2 (Intron)	0.217	0.475	1.14	2.78	6.56	14.3	29.0	53.3	—
Apis mellifera	CHR_LG3	0.203	0.458	1.12	2.80	6.85	15.6	33.1	64.1	115
Apis mellifera	CHR_LG3 (Gene)	0.239	0.535	1.31	3.27	7.97	18.1	38.3	73.6	—
Apis mellifera	CHR_LG3 (Intergenic)	0.185	0.419	1.03	2.56	6.27	14.2	30.3	58.6	105
Apis mellifera	CHR_LG3 (Exon)	0.546	1.32	3.04	8.15	22.5	59.0	142	—	—
Apis mellifera	CHR_LG3 (Intron)	0.201	0.447	1.08	2.65	6.33	14.0	29.1	55.1	—
Apis mellifera	CHR_LG4	0.218	0.491	1.21	3.03	7.42	16.9	35.6	67.9	120
Apis mellifera	CHR_LG4 (Gene)	0.225	0.512	1.27	3.21	7.95	18.4	39.3	75.7	134
Apis mellifera	CHR_LG4 (Intergenic)	0.212	0.475	1.16	2.89	7.01	15.7	32.7	61.6	107
Apis mellifera	CHR_LG4 (Exon)	0.587	1.40	3.12	8.32	23.1	61.2	148	—	—
Apis mellifera	CHR_LG4 (Intron)	0.195	0.441	1.08	2.69	6.55	14.8	31.2	59.2	—
Apis mellifera	CHR_LG5	0.240	0.537	1.32	3.31	8.15	18.7	39.9	77.2	137
Apis mellifera	CHR_LG5 (Gene)	0.249	0.559	1.38	3.45	8.50	19.5	41.5	79.5	139
Apis mellifera	CHR_LG5 (Intergenic)	0.234	0.523	1.29	3.22	7.90	18.1	38.6	74.7	132
Apis mellifera	CHR_LG5 (Exon)	0.543	1.31	3.00	8.02	22.0	58.5	147	—	—
Apis mellifera	CHR_LG5 (Intron)	0.211	0.472	1.15	2.83	6.85	15.3	31.9	59.9	—
Apis mellifera	CHR_LG6	0.231	0.522	1.29	3.23	7.95	18.1	38.5	74.8	135
Apis mellifera	CHR_LG6 (Gene)	0.246	0.550	1.35	3.35	8.14	18.4	38.8	74.5	131
Apis mellifera	CHR_LG6 (Intergenic)	0.224	0.507	1.26	3.17	7.82	17.9	38.1	74.0	133

next

Category	SN \ k	L_e (kb) 2	3	4	5	6	7	8	9	10
Apis mellifera	CHR_LG6 (Exon)	0.550	1.35	3.06	8.15	22.4	57.4	128	—	—
Apis mellifera	CHR_LG6 (Intron)	0.228	0.506	1.23	3.02	7.27	16.2	33.8	64.3	—
Apis mellifera	CHR_LG7	0.215	0.485	1.19	2.96	7.22	16.3	34.4	66.2	119
Apis mellifera	CHR_LG7 (Gene)	0.235	0.528	1.30	3.25	7.99	18.4	39.4	77.1	—
Apis mellifera	CHR_LG7 (Intergenic)	0.204	0.461	1.13	2.80	6.77	15.1	31.4	59.5	104
Apis mellifera	CHR_LG7 (Exon)	0.388	0.961	2.33	6.34	17.9	48.2	116	—	—
Apis mellifera	CHR_LG7 (Intron)	0.214	0.471	1.14	2.79	6.67	14.9	31.1	59.9	—
Apis mellifera	CHR_LG8	0.229	0.511	1.24	3.01	7.12	15.6	31.5	58.0	99.8
Apis mellifera	CHR_LG8 (Gene)	0.256	0.568	1.37	3.31	7.76	16.7	33.4	60.7	—
Apis mellifera	CHR_LG8 (Intergenic)	0.214	0.481	1.16	2.84	6.75	14.8	30.1	55.6	95.0
Apis mellifera	CHR_LG8 (Exon)	0.504	1.19	2.81	7.55	20.7	54.4	130	—	—
Apis mellifera	CHR_LG8 (Intron)	0.221	0.484	1.15	2.71	6.21	13.0	25.3	45.3	—
Apis mellifera	CHR_LG9	0.200	0.454	1.12	2.80	6.84	15.5	32.6	62.6	112
Apis mellifera	CHR_LG9 (Gene)	0.200	0.458	1.14	2.88	7.10	16.3	35.0	68.2	—
Apis mellifera	CHR_LG9 (Intergenic)	0.200	0.450	1.11	2.76	6.68	14.9	31.1	58.8	103
Apis mellifera	CHR_LG9 (Exon)	0.444	1.10	2.66	7.16	19.9	52.5	123	—	—
Apis mellifera	CHR_LG9 (Intron)	0.179	0.406	0.998	2.48	6.04	13.6	28.7	55.2	—
Apis mellifera	CHR_LG10	0.230	0.508	1.24	3.07	7.48	16.9	35.1	65.2	110
Apis mellifera	CHR_LG10 (Gene)	0.268	0.583	1.40	3.46	8.34	18.5	37.5	68.3	—
Apis mellifera	CHR_LG10 (Intergenic)	0.213	0.476	1.16	2.90	7.07	16.1	33.5	62.4	105
Apis mellifera	CHR_LG10 (Exon)	0.561	1.33	2.99	7.87	21.3	55.6	133	—	—
Apis mellifera	CHR_LG10 (Intron)	0.224	0.482	1.14	2.76	6.48	13.9	27.6	49.1	—
Apis mellifera	CHR_LG11	0.239	0.530	1.28	3.17	7.64	17.0	35.1	65.9	114
Apis mellifera	CHR_LG11 (Gene)	0.239	0.536	1.31	3.27	7.98	18.0	37.7	71.5	124
Apis mellifera	CHR_LG11 (Intergenic)	0.238	0.524	1.26	3.08	7.35	16.2	32.9	60.7	104
Apis mellifera	CHR_LG11 (Exon)	0.510	1.23	2.89	7.81	21.7	59.0	150	—	—
Apis mellifera	CHR_LG11 (Intron)	0.208	0.459	1.11	2.71	6.46	14.2	28.9	53.8	—
Apis mellifera	CHR_LG12	0.209	0.477	1.18	3.02	7.57	17.7	38.5	75.7	137
Apis mellifera	CHR_LG12 (Gene)	0.214	0.488	1.21	3.08	7.69	17.9	38.5	73.9	—
Apis mellifera	CHR_LG12 (Intergenic)	0.206	0.470	1.17	2.98	7.46	17.4	37.9	74.6	135
Apis mellifera	CHR_LG12 (Exon)	0.570	1.36	3.10	8.20	22.0	53.4	110	—	—
Apis mellifera	CHR_LG12 (Intron)	0.192	0.435	1.07	2.69	6.65	15.3	32.4	61.8	—
Apis mellifera	CHR_LG13	0.196	0.445	1.09	2.69	6.53	14.7	30.5	57.3	99.3
Apis mellifera	CHR_LG13 (Gene)	0.219	0.491	1.19	2.96	7.17	16.0	32.6	59.7	—

next

		L_e (kb)								
Category	SN \ k	2	3	4	5	6	7	8	9	10
Apis mellifera	CHR_LG13 (Intergenic)	0.190	0.432	1.05	2.61	6.33	14.2	29.7	55.9	96.5
Apis mellifera	CHR_LG13 (Exon)	0.446	1.08	2.65	7.20	20.1	53.1	120	—	—
Apis mellifera	CHR_LG13 (Intron)	0.187	0.412	0.984	2.38	5.58	12.1	23.9	43.1	—
Apis mellifera	CHR_LG14	0.193	0.433	1.05	2.60	6.24	13.9	28.6	53.8	94.1
Apis mellifera	CHR_LG14 (Gene)	0.222	0.498	1.21	2.99	7.19	16.0	32.9	61.4	—
Apis mellifera	CHR_LG14 (Intergenic)	0.181	0.406	0.986	2.43	5.84	12.9	26.7	50.1	87.0
Apis mellifera	CHR_LG14 (Exon)	0.538	1.28	2.91	7.72	21.1	54.4	122	—	—
Apis mellifera	CHR_LG14 (Intron)	0.179	0.398	0.953	2.29	5.35	11.5	23.2	42.5	—
Apis mellifera	CHR_LG15	0.213	0.476	1.17	2.90	7.04	16.0	33.7	64.7	115
Apis mellifera	CHR_LG15 (Gene)	0.267	0.594	1.45	3.60	8.70	19.6	40.6	76.6	—
Apis mellifera	CHR_LG15 (Intergenic)	0.193	0.434	1.06	2.63	6.41	14.6	30.7	58.9	103
Apis mellifera	CHR_LG15 (Exon)	0.482	1.15	2.77	7.45	20.6	53.9	125	—	—
Apis mellifera	CHR_LG15 (Intron)	0.221	0.481	1.15	2.75	6.39	13.8	27.7	51.0	—
Apis mellifera	CHR_LG16	0.219	0.481	1.15	2.79	6.63	14.5	29.4	54.0	91.7
Apis mellifera	CHR_LG16 (Gene)	0.237	0.526	1.27	3.13	7.50	16.6	34.1	64.0	—
Apis mellifera	CHR_LG16 (Intergenic)	0.207	0.451	1.07	2.57	6.03	13.0	25.9	46.2	—
Apis mellifera	CHR_LG16 (Exon)	0.576	1.35	3.09	8.04	21.1	49.8	—	—	—
Apis mellifera	CHR_LG16 (Intron)	0.214	0.473	1.14	2.75	6.53	14.2	29.0	53.9	—
Caenorhabditis elegans	CHR_I	0.110	0.233	0.553	1.42	3.84	10.7	29.8	80.9	209
Caenorhabditis elegans	CHR_I (Gene)	0.111	0.242	0.576	1.47	3.99	11.1	30.8	83.5	214
Caenorhabditis elegans	CHR_I (Intergenic)	0.105	0.214	0.504	1.29	3.50	9.72	26.9	71.6	176
Caenorhabditis elegans	CHR_I (Exon)	0.173	0.458	1.20	3.41	10.2	30.8	93.9	281	761
Caenorhabditis elegans	CHR_I (Intron)	0.074	0.140	0.307	0.738	1.89	5.02	13.3	34.5	84.5
Caenorhabditis elegans	CHR_II	0.124	0.269	0.648	1.68	4.60	13.0	36.6	101	269
Caenorhabditis elegans	CHR_II (Gene)	0.130	0.291	0.705	1.84	5.03	14.2	40.3	112	296
Caenorhabditis elegans	CHR_II (Intergenic)	0.114	0.235	0.561	1.45	3.93	11.0	30.6	82.8	209
Caenorhabditis elegans	CHR_II (Exon)	0.189	0.494	1.30	3.71	11.1	33.9	104	313	844
Caenorhabditis elegans	CHR_II (Intron)	0.084	0.163	0.360	0.868	2.23	5.93	15.8	41.1	102
Caenorhabditis elegans	CHR_III	0.102	0.215	0.506	1.28	3.46	9.59	26.6	72.2	186
Caenorhabditis elegans	CHR_III (Gene)	0.104	0.225	0.531	1.35	3.62	10.0	27.8	75.5	195
Caenorhabditis elegans	CHR_III (Intergenic)	0.097	0.195	0.455	1.16	3.10	8.56	23.4	60.5	142
Caenorhabditis elegans	CHR_III (Exon)	0.173	0.456	1.20	3.42	10.2	30.9	94.3	281	—
Caenorhabditis elegans	CHR_III (Intron)	0.070	0.134	0.292	0.697	1.78	4.74	12.6	32.9	81.4
Caenorhabditis elegans	CHR_IV	0.112	0.242	0.584	1.52	4.14	11.6	32.8	91.7	248

next

		L_e (kb)								
Category	SN \ k	2	3	4	5	6	7	8	9	10
Caenorhabditis elegans	CHR_IV (Gene)	0.121	0.271	0.663	1.74	4.81	13.7	38.9	110	298
Caenorhabditis elegans	CHR_IV (Intergenic)	0.101	0.209	0.496	1.27	3.41	9.43	26.1	71.2	185
Caenorhabditis elegans	CHR_IV (Exon)	0.178	0.467	1.23	3.49	10.4	31.6	96.5	289	777
Caenorhabditis elegans	CHR_IV (Intron)	0.081	0.159	0.359	0.881	2.30	6.21	16.8	44.7	114
Caenorhabditis elegans	CHR_V	0.127	0.278	0.678	1.78	4.90	13.8	39.2	109	295
Caenorhabditis elegans	CHR_V (Gene)	0.136	0.307	0.760	2.01	5.57	15.8	45.1	126	334
Caenorhabditis elegans	CHR_V (Intergenic)	0.115	0.240	0.577	1.50	4.07	11.4	32.0	88.0	232
Caenorhabditis elegans	CHR_V (Exon)	0.195	0.500	1.33	3.79	11.3	34.7	107	325	908
Caenorhabditis elegans	CHR_V (Intron)	0.087	0.173	0.390	0.951	2.45	6.48	17.0	43.7	107
Caenorhabditis elegans	CHR_X	0.140	0.313	0.780	2.10	5.86	16.7	48.0	137	378
Caenorhabditis elegans	CHR_X (Gene)	0.158	0.371	0.947	2.59	7.36	21.3	61.9	179	499
Caenorhabditis elegans	CHR_X (Intergenic)	0.125	0.264	0.643	1.70	4.68	13.1	37.0	103	269
Caenorhabditis elegans	CHR_X (Exon)	0.200	0.527	1.40	4.04	12.2	37.5	116	349	—
Caenorhabditis elegans	CHR_X (Intron)	0.118	0.247	0.586	1.50	4.00	10.8	28.7	75.5	191
Drosophila melanogaster	CHR_2	0.338	0.883	2.47	6.98	20.0	55.5	149	384	928
Drosophila melanogaster	CHR_2 (Gene)	0.384	0.997	2.76	7.79	22.3	61.9	166	429	1031
Drosophila melanogaster	CHR_2 (Intergenic)	0.286	0.744	2.06	5.80	16.5	45.6	121	308	731
Drosophila melanogaster	CHR_2 (Exon)	0.494	1.18	2.85	7.65	21.8	61.1	173	482	1215
Drosophila melanogaster	CHR_2 (Intron)	0.306	0.791	2.19	6.05	16.8	44.6	113	273	619
Drosophila melanogaster	CHR_3	0.325	0.849	2.37	6.66	19.0	52.1	138	350	835
Drosophila melanogaster	CHR_3 (Gene)	0.365	0.949	2.62	7.35	20.9	57.5	153	391	931
Drosophila melanogaster	CHR_3 (Intergenic)	0.280	0.726	2.01	5.63	15.9	43.5	114	285	666
Drosophila melanogaster	CHR_3 (Exon)	0.484	1.16	2.73	7.23	20.3	56.2	157	426	1047
Drosophila melanogaster	CHR_3 (Intron)	0.290	0.749	2.07	5.70	15.8	41.8	105	253	576
Drosophila melanogaster	CHR_4	0.657	1.32	3.29	8.63	22.3	53.3	113	201	—
Drosophila melanogaster	CHR_4 (Gene)	0.620	1.27	3.16	8.27	21.4	51.5	110	—	—
Drosophila melanogaster	CHR_4 (Intergenic)	0.730	1.41	3.43	8.72	20.7	41.7	67.9	—	—
Drosophila melanogaster	CHR_4 (Exon)	0.497	1.26	3.42	9.61	26.8	66.7	135	—	—
Drosophila melanogaster	CHR_4 (Intron)	0.630	1.12	2.60	6.40	15.5	34.4	68.3	—	—
Drosophila melanogaster	CHR_X	0.319	0.776	2.04	5.34	13.7	31.8	68.3	138	264
Drosophila melanogaster	CHR_X (Gene)	0.362	0.886	2.31	6.00	15.3	35.6	77.2	158	306
Drosophila melanogaster	CHR_X (Intergenic)	0.274	0.653	1.71	4.45	11.4	26.6	56.8	113	212
Drosophila melanogaster	CHR_X (Exon)	0.438	1.02	2.28	5.71	15.1	38.3	95.0	223	458
Drosophila melanogaster	CHR_X (Intron)	0.287	0.677	1.76	4.45	10.9	24.2	49.4	95.9	180

next

		L_e (kb)								
Category	SN \ k	2	3	4	5	6	7	8	9	10
Tribolium castaneum	CHR_LG01=X	0.204	0.506	1.34	3.69	10.4	29.9	85.2	236	597
Tribolium castaneum	CHR_LG01=X (Gene)	0.201	0.498	1.32	3.63	10.2	29.4	83.2	225	—
Tribolium castaneum	CHR_LG01=X (Intergenic)	0.208	0.513	1.36	3.73	10.5	29.9	84.0	224	—
Tribolium castaneum	CHR_LG01=X (Exon)	0.201	0.498	1.32	3.62	10.2	28.8	77.2	—	—
Tribolium castaneum	CHR_LG01=X (Intron)	0.202	0.498	1.32	3.63	10.2	29.3	82.3	218	—
Tribolium castaneum	CHR_LG02	0.216	0.549	1.48	4.10	11.6	33.5	96.6	272	714
Tribolium castaneum	CHR_LG02 (Gene)	0.220	0.564	1.53	4.27	12.1	35.1	101	283	714
Tribolium castaneum	CHR_LG02 (Intergenic)	0.212	0.533	1.43	3.93	11.0	31.6	89.8	246	608
Tribolium castaneum	CHR_LG02 (Exon)	0.212	0.545	1.49	4.16	11.9	34.5	97.2	249	—
Tribolium castaneum	CHR_LG02 (Intron)	0.223	0.571	1.55	4.30	12.1	34.9	99.4	271	—
Tribolium castaneum	CHR_LG03	0.160	0.374	0.942	2.48	6.79	19.0	53.6	149	395
Tribolium castaneum	CHR_LG03 (Gene)	0.156	0.365	0.920	2.43	6.65	18.7	52.6	146	384
Tribolium castaneum	CHR_LG03 (Intergenic)	0.165	0.385	0.969	2.55	6.95	19.4	54.4	149	389
Tribolium castaneum	CHR_LG03 (Exon)	0.164	0.401	1.04	2.81	7.83	22.2	62.2	165	—
Tribolium castaneum	CHR_LG03 (Intron)	0.156	0.362	0.909	2.39	6.53	18.3	51.5	142	373
Tribolium castaneum	CHR_LG04	0.198	0.494	1.31	3.60	10.1	29.2	84.1	235	609
Tribolium castaneum	CHR_LG04 (Gene)	0.194	0.484	1.28	3.50	9.84	28.3	81.0	224	570
Tribolium castaneum	CHR_LG04 (Intergenic)	0.201	0.502	1.34	3.68	10.4	29.9	85.4	234	572
Tribolium castaneum	CHR_LG04 (Exon)	0.194	0.491	1.31	3.63	10.3	29.7	84.3	222	—
Tribolium castaneum	CHR_LG04 (Intron)	0.189	0.469	1.24	3.38	9.45	27.0	76.6	208	—
Tribolium castaneum	CHR_LG05	0.213	0.533	1.42	3.90	11.0	31.8	91.7	259	690
Tribolium castaneum	CHR_LG05 (Gene)	0.207	0.518	1.38	3.81	10.8	31.4	91.1	258	683
Tribolium castaneum	CHR_LG05 (Intergenic)	0.221	0.549	1.46	3.99	11.2	32.0	90.7	248	618
Tribolium castaneum	CHR_LG05 (Exon)	0.207	0.527	1.42	3.95	11.3	32.6	92.9	249	—
Tribolium castaneum	CHR_LG05 (Intron)	0.211	0.528	1.40	3.86	10.9	31.5	90.7	253	641
Tribolium castaneum	CHR_LG06	0.130	0.293	0.711	1.82	4.87	13.5	37.4	101	254
Tribolium castaneum	CHR_LG06 (Gene)	0.126	0.283	0.683	1.74	4.66	12.9	35.7	95.8	—
Tribolium castaneum	CHR_LG06 (Intergenic)	0.135	0.304	0.739	1.90	5.07	14.0	38.7	103	—
Tribolium castaneum	CHR_LG06 (Exon)	0.128	0.294	0.723	1.87	5.04	14.0	38.2	—	—
Tribolium castaneum	CHR_LG06 (Intron)	0.124	0.276	0.663	1.69	4.51	12.4	34.4	92.0	—
Tribolium castaneum	CHR_LG07	0.204	0.508	1.35	3.71	10.4	30.2	87.3	249	675
Tribolium castaneum	CHR_LG07 (Gene)	0.204	0.510	1.36	3.73	10.5	30.4	87.8	249	663
Tribolium castaneum	CHR_LG07 (Intergenic)	0.205	0.506	1.34	3.67	10.3	29.7	85.0	236	602
Tribolium castaneum	CHR_LG07 (Exon)	0.197	0.498	1.34	3.69	10.5	30.4	86.9	233	—

next

Category	SN \ k	L_e (kb) 2	3	4	5	6	7	8	9	10
Tribolium castaneum	CHR_LG07 (Intron)	0.208	0.518	1.38	3.77	10.6	30.4	87.2	244	629
Tribolium castaneum	CHR_LG08	0.151	0.356	0.898	2.37	6.46	18.1	50.8	138	350
Tribolium castaneum	CHR_LG08 (Gene)	0.150	0.353	0.892	2.35	6.45	18.1	51.0	139	349
Tribolium castaneum	CHR_LG08 (Intergenic)	0.153	0.358	0.904	2.38	6.46	18.0	49.8	133	320
Tribolium castaneum	CHR_LG08 (Exon)	0.155	0.372	0.955	2.55	7.08	20.0	55.8	146	—
Tribolium castaneum	CHR_LG08 (Intron)	0.149	0.346	0.866	2.27	6.17	17.3	48.2	130	—
Tribolium castaneum	CHR_LG09	0.171	0.413	1.07	2.87	7.97	22.7	64.7	180	467
Tribolium castaneum	CHR_LG09 (Gene)	0.173	0.419	1.09	2.93	8.17	23.4	66.7	185	475
Tribolium castaneum	CHR_LG09 (Intergenic)	0.170	0.407	1.05	2.81	7.76	21.9	61.5	166	408
Tribolium castaneum	CHR_LG09 (Exon)	0.174	0.429	1.13	3.07	8.64	24.9	70.3	184	—
Tribolium castaneum	CHR_LG09 (Intron)	0.172	0.413	1.07	2.86	7.95	22.6	64.1	175	—
Tribolium castaneum	CHR_LG10	0.140	0.311	0.750	1.91	5.08	13.8	37.1	94.6	214
Tribolium castaneum	CHR_LG10 (Gene)	0.136	0.302	0.726	1.85	4.88	13.1	34.2	82.2	—
Tribolium castaneum	CHR_LG10 (Intergenic)	0.144	0.321	0.778	1.99	5.29	14.4	39.1	101	—
Tribolium castaneum	CHR_LG10 (Exon)	0.143	0.320	0.768	1.95	5.16	13.8	35.8	—	—
Tribolium castaneum	CHR_LG10 (Intron)	0.137	0.302	0.726	1.84	4.83	12.8	32.9	75.6	—

Effective Length List 4: Plant (17).

Category	SN \ k	L_e (kb) 2	3	4	5	6	7	8	9	10
Arabidopsis thaliana	CHR_I	0.373	0.869	2.19	5.85	15.9	41.5	107	267	621
Arabidopsis thaliana	CHR_I (Gene)	0.333	0.818	2.05	5.53	15.7	44.5	128	364	985
Arabidopsis thaliana	CHR_I (Intergenic)	0.394	0.791	1.91	4.87	12.1	28.1	63.7	137	274
Arabidopsis thaliana	CHR_I (Exon)	0.290	0.720	1.77	4.77	13.7	39.5	116	337	918
Arabidopsis thaliana	CHR_I (Intron)	0.348	0.745	1.78	4.37	10.9	26.9	67.5	167	399
Arabidopsis thaliana	CHR_II	0.378	0.879	2.22	5.94	16.1	42.2	109	272	636
Arabidopsis thaliana	CHR_II (Gene)	0.338	0.831	2.09	5.63	16.0	45.3	130	366	967
Arabidopsis thaliana	CHR_II (Intergenic)	0.401	0.811	1.97	5.03	12.5	29.4	67.6	149	308
Arabidopsis thaliana	CHR_II (Exon)	0.290	0.715	1.75	4.70	13.5	38.4	111	316	819
Arabidopsis thaliana	CHR_II (Intron)	0.356	0.762	1.82	4.45	11.1	27.3	67.7	165	—
Arabidopsis thaliana	CHR_III	0.373	0.874	2.21	5.93	16.2	42.8	112	284	677

next

Category	SN \ k	L_e (kb) 2	3	4	5	6	7	8	9	10
Arabidopsis thaliana	CHR_III (Gene)	0.333	0.825	2.07	5.61	16.0	45.7	132	379	1023
Arabidopsis thaliana	CHR_III (Intergenic)	0.397	0.798	1.93	4.93	12.3	28.6	65.8	145	303
Arabidopsis thaliana	CHR_III (Exon)	0.287	0.715	1.75	4.72	13.6	39.0	114	329	877
Arabidopsis thaliana	CHR_III (Intron)	0.349	0.755	1.82	4.49	11.3	27.9	70.1	173	—
Arabidopsis thaliana	CHR_IV	0.379	0.888	2.25	6.01	16.4	42.9	112	278	648
Arabidopsis thaliana	CHR_IV (Gene)	0.339	0.836	2.10	5.66	16.1	45.5	130	366	962
Arabidopsis thaliana	CHR_IV (Intergenic)	0.401	0.817	1.99	5.10	12.7	29.7	67.7	147	297
Arabidopsis thaliana	CHR_IV (Exon)	0.293	0.723	1.77	4.78	13.7	39.2	114	324	837
Arabidopsis thaliana	CHR_IV (Intron)	0.354	0.757	1.81	4.44	11.1	27.3	67.8	165	—
Arabidopsis thaliana	CHR_V	0.363	0.848	2.14	5.73	15.6	40.9	106	266	626
Arabidopsis thaliana	CHR_V (Gene)	0.325	0.803	2.02	5.44	15.5	43.8	126	359	969
Arabidopsis thaliana	CHR_V (Intergenic)	0.381	0.776	1.89	4.84	12.2	28.6	66.0	145	299
Arabidopsis thaliana	CHR_V (Exon)	0.282	0.702	1.73	4.65	13.4	38.4	112	324	873
Arabidopsis thaliana	CHR_V (Intron)	0.347	0.745	1.78	4.37	11.0	27.0	67.5	167	400
Oryza sativa	CHR_01	0.825	1.77	3.90	9.73	25.1	63.3	159	379	795
Oryza sativa	CHR_01 (Gene)	0.506	1.33	2.92	7.52	20.6	54.9	147	380	872
Oryza sativa	CHR_01 (Intergenic)	1.02	1.74	3.81	9.14	22.2	52.9	124	269	503
Oryza sativa	CHR_01 (Exon)	0.498	1.22	2.30	5.63	15.1	38.8	100	249	539
Oryza sativa	CHR_01 (Intron)	0.223	0.659	1.79	4.94	13.5	36.4	98.3	260	633
Oryza sativa	CHR_02	0.770	1.69	3.76	9.46	24.6	62.3	157	375	784
Oryza sativa	CHR_02 (Gene)	0.464	1.23	2.75	7.14	19.6	52.5	141	363	835
Oryza sativa	CHR_02 (Intergenic)	0.985	1.72	3.80	9.20	22.5	53.8	126	275	509
Oryza sativa	CHR_02 (Exon)	0.449	1.12	2.17	5.34	14.3	36.9	95.1	234	506
Oryza sativa	CHR_02 (Intron)	0.215	0.640	1.74	4.80	13.1	35.3	94.5	246	585
Oryza sativa	CHR_03	0.780	1.71	3.78	9.50	24.6	62.4	157	378	795
Oryza sativa	CHR_03 (Gene)	0.450	1.21	2.70	7.01	19.2	51.4	138	358	832
Oryza sativa	CHR_03 (Intergenic)	1.01	1.74	3.83	9.25	22.5	53.8	127	277	517
Oryza sativa	CHR_03 (Exon)	0.458	1.13	2.16	5.27	14.1	36.0	92.2	228	494
Oryza sativa	CHR_03 (Intron)	0.204	0.613	1.68	4.64	12.7	34.4	92.9	246	596
Oryza sativa	CHR_04	0.827	1.87	4.17	10.6	27.8	71.1	181	435	915
Oryza sativa	CHR_04 (Gene)	0.543	1.41	3.11	8.09	22.3	60.3	161	404	857
Oryza sativa	CHR_04 (Intergenic)	1.05	1.92	4.29	10.5	25.9	62.2	147	324	616
Oryza sativa	CHR_04 (Exon)	0.462	1.16	2.32	5.83	15.9	42.0	110	267	543
Oryza sativa	CHR_04 (Intron)	0.279	0.806	2.16	5.89	16.0	42.7	113	288	654

next

		L_e (kb)								
Category	SN \ k	2	3	4	5	6	7	8	9	10
Oryza sativa	CHR_05	0.792	1.76	3.90	9.82	25.5	64.0	159	370	746
Oryza sativa	CHR_05 (Gene)	0.515	1.34	2.92	7.51	20.5	54.4	143	356	760
Oryza sativa	CHR_05 (Intergenic)	0.976	1.77	3.93	9.54	23.3	55.3	128	270	486
Oryza sativa	CHR_05 (Exon)	0.449	1.12	2.19	5.42	14.6	37.6	96.2	232	474
Oryza sativa	CHR_05 (Intron)	0.238	0.701	1.90	5.19	14.2	37.8	100.0	254	572
Oryza sativa	CHR_06	0.823	1.79	3.92	9.77	25.1	62.5	153	350	686
Oryza sativa	CHR_06 (Gene)	0.534	1.38	2.97	7.57	20.5	54.0	142	353	759
Oryza sativa	CHR_06 (Intergenic)	1.01	1.78	3.91	9.41	22.8	53.4	121	249	434
Oryza sativa	CHR_06 (Exon)	0.466	1.15	2.19	5.36	14.3	36.4	92.4	222	454
Oryza sativa	CHR_06 (Intron)	0.256	0.744	2.00	5.45	14.8	39.5	105	266	602
Oryza sativa	CHR_07	0.783	1.73	3.87	9.79	25.5	64.8	163	388	799
Oryza sativa	CHR_07 (Gene)	0.506	1.32	2.91	7.54	20.7	55.2	147	372	815
Oryza sativa	CHR_07 (Intergenic)	0.943	1.71	3.81	9.32	23.0	55.4	131	283	521
Oryza sativa	CHR_07 (Exon)	0.451	1.12	2.19	5.40	14.5	37.5	96.6	235	492
Oryza sativa	CHR_07 (Intron)	0.235	0.699	1.90	5.25	14.4	38.8	104	266	608
Oryza sativa	CHR_08	0.784	1.74	3.87	9.74	25.2	63.3	157	363	725
Oryza sativa	CHR_08 (Gene)	0.508	1.32	2.90	7.52	20.6	54.9	145	362	774
Oryza sativa	CHR_08 (Intergenic)	0.984	1.77	3.92	9.50	23.1	54.7	126	265	475
Oryza sativa	CHR_08 (Exon)	0.430	1.08	2.16	5.38	14.5	37.8	97.2	235	484
Oryza sativa	CHR_08 (Intron)	0.260	0.751	2.00	5.46	14.8	39.4	103	258	567
Oryza sativa	CHR_09	0.810	1.77	3.93	9.87	25.5	63.7	155	348	660
Oryza sativa	CHR_09 (Gene)	0.542	1.39	3.03	7.82	21.4	57.0	150	371	784
Oryza sativa	CHR_09 (Intergenic)	0.991	1.76	3.90	9.43	22.9	53.5	120	239	399
Oryza sativa	CHR_09 (Exon)	0.455	1.13	2.21	5.48	14.8	38.3	98.4	237	483
Oryza sativa	CHR_09 (Intron)	0.284	0.811	2.16	5.85	15.9	41.9	109	269	572
Oryza sativa	CHR_10	0.806	1.78	3.95	9.90	25.6	64.1	158	362	708
Oryza sativa	CHR_10 (Gene)	0.534	1.37	2.97	7.63	20.9	55.5	147	368	790
Oryza sativa	CHR_10 (Intergenic)	0.981	1.80	4.00	9.71	23.7	55.6	126	259	449
Oryza sativa	CHR_10 (Exon)	0.444	1.11	2.17	5.38	14.5	37.8	97.5	236	486
Oryza sativa	CHR_10 (Intron)	0.292	0.828	2.19	5.90	15.9	41.7	108	266	565
Oryza sativa	CHR_11	0.801	1.76	3.93	9.90	25.7	64.7	160	366	713
Oryza sativa	CHR_11 (Gene)	0.534	1.37	3.03	7.88	21.8	59.0	159	401	861
Oryza sativa	CHR_11 (Intergenic)	1.00	1.79	3.94	9.48	23.0	54.0	123	255	446
Oryza sativa	CHR_11 (Exon)	0.400	1.03	2.13	5.45	15.1	40.4	108	269	562

next

		L_e (kb)								
Category	SN \ k	2	3	4	5	6	7	8	9	10
Oryza sativa	CHR_11 (Intron)	0.324	0.895	2.33	6.23	16.6	43.3	111	268	553
Oryza sativa	CHR_12	0.761	1.71	3.85	9.74	25.2	63.3	155	349	664
Oryza sativa	CHR_12 (Gene)	0.498	1.29	2.89	7.55	20.8	55.8	147	358	730
Oryza sativa	CHR_12 (Intergenic)	0.944	1.73	3.88	9.39	22.9	53.9	122	250	432
Oryza sativa	CHR_12 (Exon)	0.392	1.00	2.08	5.29	14.5	38.1	98.7	236	473
Oryza sativa	CHR_12 (Intron)	0.276	0.795	2.12	5.74	15.4	40.4	104	245	487

Effective Length List 5: Vertebrates (236).

		L_e (kb)								
Category	SN \ k	2	3	4	5	6	7	8	9	10
Bos taurus	CHR_01	0.192	0.457	1.26	3.57	10.2	27.6	67.0	136	230
Bos taurus	CHR_01 (Gene)	0.193	0.459	1.27	3.63	10.5	29.3	75.0	164	298
Bos taurus	CHR_01 (Intergenic)	0.191	0.456	1.25	3.54	10.0	26.9	63.9	127	208
Bos taurus	CHR_01 (Exon)	0.195	0.471	1.25	3.61	11.0	33.2	99.0	267	—
Bos taurus	CHR_01 (Intron)	0.193	0.457	1.26	3.60	10.4	28.6	72.2	155	276
Bos taurus	CHR_02	0.186	0.449	1.25	3.56	10.2	28.2	70.3	148	257
Bos taurus	CHR_02 (Gene)	0.207	0.487	1.34	3.80	10.9	30.3	77.1	169	307
Bos taurus	CHR_02 (Intergenic)	0.198	0.471	1.29	3.65	10.3	27.7	66.5	133	221
Bos taurus	CHR_02 (Exon)	0.192	0.470	1.24	3.59	10.9	32.7	97.6	269	—
Bos taurus	CHR_02 (Intron)	0.189	0.452	1.26	3.60	10.4	29.0	74.4	163	298
Bos taurus	CHR_03	0.180	0.436	1.21	3.47	10.0	28.0	70.8	151	267
Bos taurus	CHR_03 (Gene)	0.185	0.446	1.24	3.58	10.5	29.8	78.9	180	341
Bos taurus	CHR_03 (Intergenic)	0.177	0.430	1.19	3.41	9.80	27.0	66.7	138	234
Bos taurus	CHR_03 (Exon)	0.177	0.434	1.15	3.31	10.0	30.1	90.3	255	—
Bos taurus	CHR_03 (Intron)	0.185	0.445	1.24	3.55	10.3	29.1	75.5	168	310
Bos taurus	CHR_04	0.194	0.462	1.27	3.63	10.4	28.6	71.2	149	259
Bos taurus	CHR_04 (Gene)	0.196	0.464	1.28	3.67	10.6	29.7	76.1	167	304
Bos taurus	CHR_04 (Intergenic)	0.193	0.460	1.27	3.60	10.3	28.0	68.3	140	236
Bos taurus	CHR_04 (Exon)	0.195	0.470	1.23	3.53	10.6	31.7	91.7	—	—
Bos taurus	CHR_04 (Intron)	0.195	0.462	1.28	3.65	10.5	29.1	73.5	159	284
Bos taurus	CHR_05	0.178	0.433	1.21	3.45	9.98	27.7	69.9	149	263

next

			L_e (kb)								
Category	SN	$\backslash$ k	2	3	4	5	6	7	8	9	10
Bos taurus	CHR_05 (Gene)		0.178	0.431	1.20	3.47	10.2	28.9	76.4	175	331
Bos taurus	CHR_05 (Intergenic)		0.179	0.433	1.20	3.43	9.84	26.9	66.1	136	230
Bos taurus	CHR_05 (Exon)		0.177	0.435	1.13	3.22	9.65	28.6	84.7	236	—
Bos taurus	CHR_05 (Intron)		0.177	0.428	1.20	3.44	9.99	28.1	72.9	163	301
Bos taurus	CHR_06		0.201	0.478	1.31	3.71	10.5	28.1	67.1	134	223
Bos taurus	CHR_06 (Gene)		0.209	0.493	1.36	3.85	11.0	29.9	73.9	154	268
Bos taurus	CHR_06 (Intergenic)		0.199	0.473	1.30	3.66	10.3	27.4	64.7	127	208
Bos taurus	CHR_06 (Exon)		0.221	0.523	1.38	3.99	12.0	35.6	100	—	—
Bos taurus	CHR_06 (Intron)		0.208	0.490	1.35	3.81	10.8	29.2	70.9	146	249
Bos taurus	CHR_07		0.172	0.421	1.18	3.39	9.87	27.8	71.7	158	287
Bos taurus	CHR_07 (Gene)		0.160	0.394	1.11	3.21	9.48	27.5	74.9	180	363
Bos taurus	CHR_07 (Intergenic)		0.179	0.433	1.21	3.45	9.95	27.5	68.5	144	249
Bos taurus	CHR_07 (Exon)		0.176	0.431	1.10	3.10	9.22	27.1	79.9	225	—
Bos taurus	CHR_07 (Intron)		0.158	0.388	1.09	3.15	9.26	26.5	71.0	166	324
Bos taurus	CHR_08		0.184	0.440	1.22	3.47	9.97	27.4	67.8	141	243
Bos taurus	CHR_08 (Gene)		0.181	0.434	1.21	3.47	10.1	28.5	74.0	165	305
Bos taurus	CHR_08 (Intergenic)		0.185	0.442	1.22	3.46	9.88	26.8	64.7	131	218
Bos taurus	CHR_08 (Exon)		0.187	0.450	1.19	3.42	10.3	30.8	88.9	—	—
Bos taurus	CHR_08 (Intron)		0.180	0.432	1.20	3.44	9.96	27.9	71.4	156	284
Bos taurus	CHR_09		0.198	0.470	1.29	3.65	10.4	28.0	67.6	137	231
Bos taurus	CHR_09 (Gene)		0.205	0.482	1.33	3.77	10.8	29.6	74.0	157	278
Bos taurus	CHR_09 (Intergenic)		0.195	0.464	1.27	3.60	10.2	27.2	64.9	129	213
Bos taurus	CHR_09 (Exon)		0.211	0.500	1.32	3.81	11.4	34.0	96.2	—	—
Bos taurus	CHR_09 (Intron)		0.205	0.480	1.32	3.74	10.7	29.0	71.4	149	259
Bos taurus	CHR_10		0.184	0.444	1.23	3.53	10.2	28.3	71.7	154	272
Bos taurus	CHR_10 (Gene)		0.190	0.457	1.27	3.63	10.6	29.8	77.8	176	328
Bos taurus	CHR_10 (Intergenic)		0.180	0.437	1.21	3.47	9.97	27.5	68.3	142	245
Bos taurus	CHR_10 (Exon)		0.180	0.440	1.16	3.36	10.2	30.7	91.8	253	—
Bos taurus	CHR_10 (Intron)		0.191	0.456	1.26	3.61	10.4	29.1	74.5	164	300
Bos taurus	CHR_11		0.173	0.420	1.17	3.36	9.76	27.3	69.3	150	267
Bos taurus	CHR_11 (Gene)		0.174	0.421	1.18	3.40	9.97	28.5	75.9	175	337
Bos taurus	CHR_11 (Intergenic)		0.172	0.419	1.17	3.34	9.60	26.5	65.8	137	237
Bos taurus	CHR_11 (Exon)		0.197	0.475	1.23	3.52	10.6	31.7	94.2	261	—
Bos taurus	CHR_11 (Intron)		0.172	0.416	1.16	3.35	9.78	27.7	72.6	164	308

next

			L_e (kb)								
Category	SN	\ k	2	3	4	5	6	7	8	9	10
Bos taurus	CHR_12		0.197	0.468	1.29	3.66	10.4	28.0	67.5	137	232
Bos taurus	CHR_12 (Gene)		0.205	0.479	1.32	3.78	10.9	30.4	77.3	168	304
Bos taurus	CHR_12 (Intergenic)		0.195	0.464	1.28	3.61	10.2	27.2	64.4	128	212
Bos taurus	CHR_12 (Exon)		0.253	0.588	1.51	4.32	12.9	37.5	102	—	—
Bos taurus	CHR_12 (Intron)		0.203	0.472	1.30	3.72	10.7	29.4	73.7	157	280
Bos taurus	CHR_13		0.167	0.407	1.14	3.27	9.55	27.0	70.4	157	291
Bos taurus	CHR_13 (Gene)		0.170	0.410	1.15	3.32	9.79	28.3	76.7	183	364
Bos taurus	CHR_13 (Intergenic)		0.166	0.405	1.13	3.24	9.41	26.3	67.3	146	262
Bos taurus	CHR_13 (Exon)		0.190	0.464	1.19	3.40	10.2	30.1	87.7	233	—
Bos taurus	CHR_13 (Intron)		0.168	0.405	1.13	3.27	9.59	27.5	73.3	171	333
Bos taurus	CHR_14		0.191	0.456	1.26	3.59	10.3	28.0	68.7	141	242
Bos taurus	CHR_14 (Gene)		0.201	0.474	1.31	3.76	10.9	30.4	77.7	170	310
Bos taurus	CHR_14 (Intergenic)		0.187	0.450	1.24	3.53	10.0	27.1	65.4	132	220
Bos taurus	CHR_14 (Exon)		0.218	0.517	1.33	3.81	11.4	33.3	92.7	—	—
Bos taurus	CHR_14 (Intron)		0.199	0.469	1.30	3.70	10.6	29.4	73.9	158	282
Bos taurus	CHR_15		0.177	0.429	1.19	3.42	9.90	27.6	70.0	150	266
Bos taurus	CHR_15 (Gene)		0.175	0.425	1.19	3.42	10.1	28.9	77.3	180	349
Bos taurus	CHR_15 (Intergenic)		0.177	0.429	1.19	3.40	9.77	26.8	66.2	137	234
Bos taurus	CHR_15 (Exon)		0.183	0.445	1.15	3.30	9.91	29.4	85.4	—	—
Bos taurus	CHR_15 (Intron)		0.174	0.421	1.18	3.38	9.86	28.0	73.6	167	315
Bos taurus	CHR_16		0.175	0.426	1.19	3.40	9.84	27.4	69.7	150	266
Bos taurus	CHR_16 (Gene)		0.178	0.431	1.20	3.47	10.2	29.0	76.8	176	335
Bos taurus	CHR_16 (Intergenic)		0.174	0.422	1.18	3.36	9.66	26.6	66.2	138	238
Bos taurus	CHR_16 (Exon)		0.187	0.450	1.16	3.30	9.89	29.3	85.6	—	—
Bos taurus	CHR_16 (Intron)		0.177	0.428	1.20	3.44	10.0	28.3	73.6	165	306
Bos taurus	CHR_17		0.178	0.431	1.20	3.45	9.99	27.8	70.4	151	269
Bos taurus	CHR_17 (Gene)		0.178	0.429	1.20	3.45	10.1	28.7	76.0	175	334
Bos taurus	CHR_17 (Intergenic)		0.178	0.431	1.20	3.43	9.85	27.0	66.3	137	233
Bos taurus	CHR_17 (Exon)		0.207	0.490	1.23	3.47	10.3	30.1	86.5	—	—
Bos taurus	CHR_17 (Intron)		0.176	0.422	1.18	3.40	9.87	27.8	72.3	162	304
Bos taurus	CHR_18		0.160	0.391	1.10	3.17	9.30	26.6	70.8	164	314
Bos taurus	CHR_18 (Gene)		0.156	0.382	1.07	3.11	9.19	26.7	73.3	179	369
Bos taurus	CHR_18 (Intergenic)		0.161	0.394	1.11	3.19	9.31	26.4	68.6	153	282
Bos taurus	CHR_18 (Exon)		0.182	0.441	1.11	3.14	9.28	27.0	78.5	217	—

next

Category	SN \ k	L_e (kb) 2	3	4	5	6	7	8	9	10
Bos taurus	CHR_18 (Intron)	0.153	0.374	1.05	3.04	8.92	25.6	69.0	163	325
Bos taurus	CHR_19	0.154	0.381	1.07	3.09	9.04	25.8	68.6	158	304
Bos taurus	CHR_19 (Gene)	0.155	0.381	1.07	3.10	9.18	26.8	74.7	187	396
Bos taurus	CHR_19 (Intergenic)	0.154	0.381	1.07	3.06	8.90	25.0	64.0	140	254
Bos taurus	CHR_19 (Exon)	0.169	0.415	1.06	3.01	8.96	26.5	78.8	225	—
Bos taurus	CHR_19 (Intron)	0.153	0.375	1.05	3.05	8.98	26.0	70.9	172	351
Bos taurus	CHR_20	0.193	0.461	1.27	3.61	10.3	27.7	67.2	137	230
Bos taurus	CHR_20 (Gene)	0.200	0.472	1.30	3.71	10.7	29.4	73.9	158	282
Bos taurus	CHR_20 (Intergenic)	0.191	0.456	1.26	3.56	10.1	27.0	64.4	128	211
Bos taurus	CHR_20 (Exon)	0.229	0.534	1.39	4.01	12.0	34.7	92.1	—	—
Bos taurus	CHR_20 (Intron)	0.198	0.468	1.29	3.67	10.5	28.7	71.4	151	265
Bos taurus	CHR_21	0.172	0.419	1.17	3.35	9.68	26.9	68.0	145	257
Bos taurus	CHR_21 (Gene)	0.174	0.418	1.17	3.36	9.86	28.2	74.9	173	330
Bos taurus	CHR_21 (Intergenic)	0.172	0.418	1.17	3.33	9.56	26.2	64.8	134	230
Bos taurus	CHR_21 (Exon)	0.192	0.460	1.17	3.34	9.93	29.0	82.8	—	—
Bos taurus	CHR_21 (Intron)	0.172	0.413	1.15	3.32	9.67	27.3	71.3	160	299
Bos taurus	CHR_22	0.179	0.429	1.20	3.44	10.0	28.3	73.7	165	305
Bos taurus	CHR_22 (Gene)	0.177	0.423	1.18	3.41	10.0	28.9	78.0	186	370
Bos taurus	CHR_22 (Intergenic)	0.180	0.432	1.20	3.44	9.97	27.8	70.5	151	268
Bos taurus	CHR_22 (Exon)	0.188	0.449	1.15	3.28	9.77	28.7	83.1	—	—
Bos taurus	CHR_22 (Intron)	0.176	0.419	1.17	3.36	9.85	28.1	75.0	175	342
Bos taurus	CHR_23	0.176	0.427	1.19	3.42	9.90	27.6	70.1	152	271
Bos taurus	CHR_23 (Gene)	0.174	0.422	1.18	3.40	9.99	28.6	76.3	177	340
Bos taurus	CHR_23 (Intergenic)	0.177	0.430	1.20	3.42	9.82	27.0	66.9	140	242
Bos taurus	CHR_23 (Exon)	0.170	0.416	1.07	3.05	9.06	26.6	76.8	—	—
Bos taurus	CHR_23 (Intron)	0.174	0.419	1.17	3.37	9.81	27.7	72.2	162	301
Bos taurus	CHR_24	0.191	0.457	1.26	3.60	10.3	28.2	69.4	144	249
Bos taurus	CHR_24 (Gene)	0.199	0.468	1.29	3.70	10.7	29.8	76.2	167	303
Bos taurus	CHR_24 (Intergenic)	0.189	0.452	1.25	3.55	10.1	27.5	66.7	136	229
Bos taurus	CHR_24 (Exon)	0.237	0.551	1.43	4.12	12.3	35.9	97.4	—	—
Bos taurus	CHR_24 (Intron)	0.196	0.462	1.28	3.64	10.5	29.0	73.0	157	281
Bos taurus	CHR_25	0.159	0.390	1.09	3.15	9.18	26.0	67.5	151	279
Bos taurus	CHR_25 (Gene)	0.156	0.381	1.07	3.08	9.10	26.4	72.5	176	360
Bos taurus	CHR_25 (Intergenic)	0.160	0.394	1.10	3.16	9.13	25.3	63.6	135	238

next

			L_e (kb)							
Category	SN \ k	2	3	4	5	6	7	8	9	10
Bos taurus	CHR_25 (Exon)	0.175	0.419	1.03	2.88	8.41	24.2	69.9	191	—
Bos taurus	CHR_25 (Intron)	0.153	0.373	1.05	3.03	8.89	25.5	68.5	161	318
Bos taurus	CHR_26	0.177	0.430	1.20	3.44	9.95	27.7	70.2	151	267
Bos taurus	CHR_26 (Gene)	0.176	0.428	1.19	3.44	10.1	28.7	75.3	170	318
Bos taurus	CHR_26 (Intergenic)	0.176	0.430	1.20	3.42	9.86	27.1	67.4	141	242
Bos taurus	CHR_26 (Exon)	0.190	0.461	1.23	3.54	10.7	31.5	88.0	—	—
Bos taurus	CHR_26 (Intron)	0.175	0.424	1.18	3.40	9.90	27.9	72.0	159	291
Bos taurus	CHR_27	0.197	0.466	1.28	3.65	10.4	28.2	68.9	142	244
Bos taurus	CHR_27 (Gene)	0.204	0.475	1.31	3.73	10.7	29.5	74.0	158	283
Bos taurus	CHR_27 (Intergenic)	0.195	0.462	1.27	3.61	10.3	27.6	66.8	135	228
Bos taurus	CHR_27 (Exon)	0.230	0.539	1.41	4.07	12.1	34.8	90.0	—	—
Bos taurus	CHR_27 (Intron)	0.202	0.470	1.29	3.68	10.5	28.6	70.5	148	260
Bos taurus	CHR_28	0.180	0.435	1.21	3.47	10.1	28.3	72.5	158	283
Bos taurus	CHR_28 (Gene)	0.186	0.446	1.24	3.57	10.4	29.7	78.7	181	342
Bos taurus	CHR_28 (Intergenic)	0.178	0.429	1.19	3.42	9.88	27.5	69.2	147	255
Bos taurus	CHR_28 (Exon)	0.194	0.464	1.23	3.55	10.7	31.5	87.4	—	—
Bos taurus	CHR_28 (Intron)	0.186	0.444	1.23	3.54	10.3	29.1	75.9	171	318
Bos taurus	CHR_29	0.170	0.413	1.15	3.31	9.62	27.0	69.0	150	268
Bos taurus	CHR_29 (Gene)	0.163	0.398	1.12	3.23	9.54	27.7	75.3	181	360
Bos taurus	CHR_29 (Intergenic)	0.172	0.417	1.16	3.32	9.57	26.4	65.8	138	237
Bos taurus	CHR_29 (Exon)	0.176	0.430	1.08	3.04	8.94	25.7	72.8	—	—
Bos taurus	CHR_29 (Intron)	0.161	0.392	1.10	3.18	9.34	26.8	71.5	166	321
Bos taurus	CHR_X	0.188	0.453	1.25	3.55	10.1	27.3	65.9	133	224
Bos taurus	CHR_X (Gene)	0.190	0.459	1.28	3.65	10.5	29.4	74.8	163	294
Bos taurus	CHR_X (Intergenic)	0.188	0.450	1.24	3.51	9.91	26.5	62.7	124	203
Bos taurus	CHR_X (Exon)	0.180	0.454	1.20	3.47	10.4	30.9	87.8	—	—
Bos taurus	CHR_X (Intron)	0.190	0.457	1.27	3.61	10.3	28.4	71.1	151	267
Canis familiaris	CHR_01	0.186	0.433	1.19	3.28	8.92	22.3	50.1	97.2	165
Canis familiaris	CHR_01 (Gene)	0.182	0.424	1.16	3.22	8.77	21.9	48.9	94.0	158
Canis familiaris	CHR_01 (Intergenic)	0.187	0.437	1.20	3.30	8.98	22.5	50.7	98.5	167
Canis familiaris	CHR_01 (Exon)	0.194	0.470	1.21	3.46	10.3	30.3	87.2	232	—
Canis familiaris	CHR_01 (Intron)	0.181	0.419	1.15	3.15	8.50	20.9	46.0	87.1	145
Canis familiaris	CHR_02	0.176	0.417	1.15	3.17	8.62	21.5	47.7	91.4	153
Canis familiaris	CHR_02 (Gene)	0.181	0.424	1.16	3.20	8.62	21.1	46.0	86.3	142

next

			L_e (kb)								
Category	SN	$\backslash k$	2	3	4	5	6	7	8	9	10
Canis familiaris	CHR_02 (Intergenic)		0.173	0.412	1.14	3.15	8.60	21.6	48.6	94.2	159
Canis familiaris	CHR_02 (Exon)		0.210	0.510	1.30	3.73	11.2	32.9	95.3	252	—
Canis familiaris	CHR_02 (Intron)		0.180	0.418	1.14	3.13	8.35	20.2	43.4	80.3	131
Canis familiaris	CHR_03		0.193	0.450	1.23	3.39	9.24	23.2	52.7	104	179
Canis familiaris	CHR_03 (Gene)		0.184	0.430	1.18	3.27	8.98	22.7	51.7	102	174
Canis familiaris	CHR_03 (Intergenic)		0.197	0.458	1.25	3.43	9.31	23.3	52.9	104	179
Canis familiaris	CHR_03 (Exon)		0.206	0.497	1.29	3.70	11.2	33.4	97.5	255	—
Canis familiaris	CHR_03 (Intron)		0.183	0.426	1.17	3.22	8.79	22.0	49.5	96.1	163
Canis familiaris	CHR_04		0.192	0.449	1.23	3.39	9.21	23.1	52.0	101	173
Canis familiaris	CHR_04 (Gene)		0.187	0.439	1.20	3.31	8.98	22.3	49.5	95.1	160
Canis familiaris	CHR_04 (Intergenic)		0.194	0.454	1.24	3.42	9.31	23.4	53.1	104	179
Canis familiaris	CHR_04 (Exon)		0.195	0.482	1.27	3.67	11.1	33.2	96.7	254	—
Canis familiaris	CHR_04 (Intron)		0.187	0.435	1.19	3.26	8.78	21.6	47.4	89.9	150
Canis familiaris	CHR_05		0.163	0.390	1.08	3.02	8.40	21.7	50.3	100	172
Canis familiaris	CHR_05 (Gene)		0.158	0.378	1.04	2.92	8.06	20.6	46.9	91.7	155
Canis familiaris	CHR_05 (Intergenic)		0.165	0.396	1.10	3.08	8.56	22.2	52.0	105	182
Canis familiaris	CHR_05 (Exon)		0.177	0.433	1.09	3.09	9.19	27.2	80.9	231	—
Canis familiaris	CHR_05 (Intron)		0.156	0.372	1.03	2.85	7.80	19.6	43.9	84.1	140
Canis familiaris	CHR_06		0.176	0.418	1.15	3.19	8.69	21.7	48.4	93.1	156
Canis familiaris	CHR_06 (Gene)		0.170	0.403	1.11	3.09	8.45	21.2	47.4	90.8	152
Canis familiaris	CHR_06 (Intergenic)		0.180	0.426	1.18	3.24	8.79	21.9	48.8	93.8	158
Canis familiaris	CHR_06 (Exon)		0.180	0.442	1.13	3.22	9.63	28.5	84.5	237	—
Canis familiaris	CHR_06 (Intron)		0.169	0.398	1.10	3.02	8.17	20.2	44.2	83.4	138
Canis familiaris	CHR_07		0.184	0.432	1.18	3.26	8.88	22.2	50.1	97.2	165
Canis familiaris	CHR_07 (Gene)		0.186	0.432	1.18	3.25	8.83	22.0	49.0	94.3	159
Canis familiaris	CHR_07 (Intergenic)		0.183	0.431	1.18	3.27	8.90	22.4	50.6	98.7	168
Canis familiaris	CHR_07 (Exon)		0.187	0.457	1.19	3.42	10.3	31.2	92.8	255	—
Canis familiaris	CHR_07 (Intron)		0.185	0.429	1.17	3.21	8.63	21.2	46.7	88.8	149
Canis familiaris	CHR_08		0.188	0.441	1.21	3.32	8.98	22.3	49.5	94.9	159
Canis familiaris	CHR_08 (Gene)		0.187	0.437	1.19	3.28	8.82	21.6	47.1	88.5	146
Canis familiaris	CHR_08 (Intergenic)		0.189	0.442	1.21	3.33	9.04	22.5	50.6	97.8	165
Canis familiaris	CHR_08 (Exon)		0.196	0.481	1.25	3.60	10.8	32.2	93.1	242	—
Canis familiaris	CHR_08 (Intron)		0.186	0.433	1.18	3.21	8.56	20.7	44.5	82.5	135
Canis familiaris	CHR_09		0.146	0.352	0.975	2.69	7.30	18.0	39.4	73.7	120

next

Category	SN \ k	L_e (kb) 2	3	4	5	6	7	8	9	10
Canis familiaris	CHR_09 (Gene)	0.146	0.351	0.971	2.68	7.26	17.9	39.0	72.5	118
Canis familiaris	CHR_09 (Intergenic)	0.145	0.352	0.978	2.70	7.31	18.1	39.7	74.4	122
Canis familiaris	CHR_09 (Exon)	0.170	0.423	1.06	2.98	8.86	26.0	77.3	222	—
Canis familiaris	CHR_09 (Intron)	0.144	0.344	0.949	2.60	6.94	16.8	35.7	65.2	104
Canis familiaris	CHR_10	0.178	0.421	1.16	3.21	8.73	21.8	48.7	93.6	158
Canis familiaris	CHR_10 (Gene)	0.174	0.412	1.14	3.14	8.56	21.3	47.3	90.4	151
Canis familiaris	CHR_10 (Intergenic)	0.180	0.425	1.17	3.24	8.81	22.0	49.3	95.0	160
Canis familiaris	CHR_10 (Exon)	0.205	0.506	1.29	3.68	11.0	32.5	94.7	252	—
Canis familiaris	CHR_10 (Intron)	0.172	0.406	1.12	3.08	8.31	20.5	44.8	84.4	140
Canis familiaris	CHR_11	0.190	0.442	1.21	3.30	8.91	22.0	48.9	93.6	157
Canis familiaris	CHR_11 (Gene)	0.184	0.427	1.17	3.20	8.59	21.0	45.9	86.5	143
Canis familiaris	CHR_11 (Intergenic)	0.193	0.448	1.22	3.35	9.04	22.4	50.1	96.6	163
Canis familiaris	CHR_11 (Exon)	0.193	0.475	1.25	3.63	11.0	32.8	95.4	—	—
Canis familiaris	CHR_11 (Intron)	0.183	0.423	1.15	3.15	8.38	20.3	43.7	81.4	134
Canis familiaris	CHR_12	0.202	0.463	1.25	3.40	9.07	22.1	48.3	91.3	152
Canis familiaris	CHR_12 (Gene)	0.197	0.452	1.23	3.34	8.89	21.5	46.6	87.2	144
Canis familiaris	CHR_12 (Intergenic)	0.205	0.468	1.27	3.43	9.14	22.3	48.9	93.0	156
Canis familiaris	CHR_12 (Exon)	0.178	0.448	1.19	3.43	10.4	31.2	91.6	244	—
Canis familiaris	CHR_12 (Intron)	0.197	0.450	1.22	3.28	8.65	20.7	44.1	81.6	134
Canis familiaris	CHR_13	0.203	0.471	1.29	3.53	9.58	23.9	53.9	105	180
Canis familiaris	CHR_13 (Gene)	0.205	0.473	1.29	3.53	9.54	23.6	52.3	101	170
Canis familiaris	CHR_13 (Intergenic)	0.201	0.469	1.28	3.52	9.56	24.0	54.3	106	182
Canis familiaris	CHR_13 (Exon)	0.215	0.522	1.34	3.82	11.4	33.6	95.3	—	—
Canis familiaris	CHR_13 (Intron)	0.204	0.469	1.27	3.48	9.31	22.8	50.0	95.2	160
Canis familiaris	CHR_14	0.206	0.473	1.28	3.49	9.39	23.2	51.8	100	171
Canis familiaris	CHR_14 (Gene)	0.205	0.471	1.27	3.48	9.31	22.8	50.1	96.0	162
Canis familiaris	CHR_14 (Intergenic)	0.207	0.474	1.28	3.50	9.41	23.3	52.4	102	174
Canis familiaris	CHR_14 (Exon)	0.206	0.506	1.33	3.84	11.6	34.1	95.5	—	—
Canis familiaris	CHR_14 (Intron)	0.205	0.468	1.26	3.43	9.13	22.1	48.2	91.4	154
Canis familiaris	CHR_15	0.200	0.462	1.26	3.42	9.09	22.0	47.8	90.1	150
Canis familiaris	CHR_15 (Gene)	0.202	0.465	1.26	3.41	8.99	21.5	45.7	84.6	139
Canis familiaris	CHR_15 (Intergenic)	0.198	0.460	1.25	3.41	9.12	22.2	48.7	92.4	155
Canis familiaris	CHR_15 (Exon)	0.198	0.488	1.28	3.71	11.2	33.5	96.9	—	—
Canis familiaris	CHR_15 (Intron)	0.202	0.461	1.24	3.34	8.74	20.6	43.2	79.0	128

next

		L_e (kb)								
Category	SN \ k	2	3	4	5	6	7	8	9	10
Canis familiaris	CHR_16	0.195	0.449	1.23	3.37	9.12	22.7	50.9	98.6	168
Canis familiaris	CHR_16 (Gene)	0.190	0.438	1.20	3.30	8.93	22.2	49.3	94.5	159
Canis familiaris	CHR_16 (Intergenic)	0.196	0.453	1.24	3.39	9.18	22.9	51.4	99.9	170
Canis familiaris	CHR_16 (Exon)	0.191	0.464	1.20	3.45	10.4	30.8	88.5	—	—
Canis familiaris	CHR_16 (Intron)	0.190	0.435	1.18	3.24	8.70	21.3	46.6	88.2	147
Canis familiaris	CHR_17	0.180	0.423	1.16	3.22	8.83	22.3	50.5	98.6	167
Canis familiaris	CHR_17 (Gene)	0.177	0.416	1.14	3.14	8.51	21.0	46.1	87.0	144
Canis familiaris	CHR_17 (Intergenic)	0.181	0.427	1.18	3.26	8.98	22.9	52.8	105	180
Canis familiaris	CHR_17 (Exon)	0.180	0.446	1.17	3.39	10.2	30.8	90.8	243	—
Canis familiaris	CHR_17 (Intron)	0.177	0.412	1.13	3.08	8.25	20.1	43.3	80.5	132
Canis familiaris	CHR_18	0.179	0.422	1.17	3.22	8.78	22.0	49.3	95.3	161
Canis familiaris	CHR_18 (Gene)	0.169	0.403	1.12	3.11	8.60	21.9	50.0	98.2	167
Canis familiaris	CHR_18 (Intergenic)	0.184	0.433	1.19	3.27	8.83	21.8	48.5	92.8	156
Canis familiaris	CHR_18 (Exon)	0.179	0.442	1.13	3.19	9.51	27.9	81.3	219	—
Canis familiaris	CHR_18 (Intron)	0.168	0.397	1.10	3.04	8.30	20.8	46.6	89.6	150
Canis familiaris	CHR_19	0.213	0.483	1.30	3.53	9.39	22.9	50.6	97.3	165
Canis familiaris	CHR_19 (Gene)	0.211	0.478	1.28	3.46	9.09	21.6	46.2	86.1	143
Canis familiaris	CHR_19 (Intergenic)	0.213	0.484	1.30	3.54	9.45	23.2	51.8	101	172
Canis familiaris	CHR_19 (Exon)	0.215	0.528	1.39	3.97	11.8	34.2	90.2	—	—
Canis familiaris	CHR_19 (Intron)	0.211	0.474	1.27	3.41	8.88	21.0	44.4	81.9	135
Canis familiaris	CHR_20	0.160	0.386	1.07	2.99	8.23	20.9	47.1	91.1	153
Canis familiaris	CHR_20 (Gene)	0.152	0.369	1.03	2.89	8.05	20.8	48.0	94.8	161
Canis familiaris	CHR_20 (Intergenic)	0.167	0.400	1.11	3.06	8.32	20.7	46.0	87.3	144
Canis familiaris	CHR_20 (Exon)	0.184	0.447	1.10	3.06	9.01	26.0	75.7	211	—
Canis familiaris	CHR_20 (Intron)	0.150	0.361	1.01	2.81	7.74	19.7	44.5	86.1	144
Canis familiaris	CHR_21	0.195	0.453	1.24	3.39	9.18	22.8	51.1	98.6	166
Canis familiaris	CHR_21 (Gene)	0.188	0.439	1.20	3.32	9.02	22.5	50.2	96.6	162
Canis familiaris	CHR_21 (Intergenic)	0.199	0.460	1.25	3.42	9.24	22.9	51.3	98.9	166
Canis familiaris	CHR_21 (Exon)	0.179	0.447	1.18	3.40	10.2	30.4	86.8	—	—
Canis familiaris	CHR_21 (Intron)	0.187	0.436	1.19	3.27	8.79	21.7	47.6	90.3	150
Canis familiaris	CHR_22	0.222	0.500	1.35	3.65	9.74	23.9	53.3	103	176
Canis familiaris	CHR_22 (Gene)	0.212	0.479	1.29	3.53	9.41	22.8	49.6	93.4	156
Canis familiaris	CHR_22 (Intergenic)	0.225	0.505	1.36	3.67	9.81	24.1	54.1	106	181
Canis familiaris	CHR_22 (Exon)	0.230	0.558	1.45	4.15	12.4	35.6	93.9	—	—

next

			L_e (kb)								
Category	SN	\ k	2	3	4	5	6	7	8	9	10
Canis familiaris	CHR_22 (Intron)		0.211	0.475	1.28	3.48	9.22	22.2	47.7	89.1	148
Canis familiaris	CHR_23		0.190	0.443	1.21	3.33	9.05	22.7	51.3	100	172
Canis familiaris	CHR_23 (Gene)		0.188	0.437	1.19	3.27	8.85	22.0	49.0	94.4	160
Canis familiaris	CHR_23 (Intergenic)		0.191	0.446	1.22	3.36	9.16	23.1	52.5	103	178
Canis familiaris	CHR_23 (Exon)		0.193	0.480	1.27	3.65	11.0	32.6	91.8	—	—
Canis familiaris	CHR_23 (Intron)		0.187	0.434	1.18	3.23	8.68	21.4	47.1	90.0	151
Canis familiaris	CHR_24		0.160	0.386	1.07	3.00	8.30	21.3	48.8	95.8	163
Canis familiaris	CHR_24 (Gene)		0.156	0.376	1.04	2.92	8.07	20.6	46.6	90.4	152
Canis familiaris	CHR_24 (Intergenic)		0.161	0.390	1.09	3.03	8.40	21.6	49.7	98.0	167
Canis familiaris	CHR_24 (Exon)		0.186	0.459	1.15	3.25	9.61	28.0	80.2	—	—
Canis familiaris	CHR_24 (Intron)		0.154	0.370	1.03	2.85	7.80	19.6	43.7	83.4	138
Canis familiaris	CHR_25		0.186	0.435	1.19	3.30	9.04	22.8	51.8	102	173
Canis familiaris	CHR_25 (Gene)		0.190	0.441	1.21	3.34	9.10	22.8	51.5	101	171
Canis familiaris	CHR_25 (Intergenic)		0.183	0.431	1.19	3.28	8.98	22.7	51.8	102	173
Canis familiaris	CHR_25 (Exon)		0.197	0.485	1.25	3.58	10.8	31.8	90.6	—	—
Canis familiaris	CHR_25 (Intron)		0.189	0.438	1.20	3.29	8.89	22.1	49.2	94.8	160
Canis familiaris	CHR_26		0.156	0.372	1.03	2.85	7.83	19.8	44.6	85.9	143
Canis familiaris	CHR_26 (Gene)		0.151	0.359	0.993	2.75	7.55	19.0	42.4	80.8	133
Canis familiaris	CHR_26 (Intergenic)		0.159	0.379	1.05	2.91	8.00	20.3	45.8	88.6	148
Canis familiaris	CHR_26 (Exon)		0.175	0.427	1.08	3.02	8.88	25.7	72.2	—	—
Canis familiaris	CHR_26 (Intron)		0.149	0.353	0.975	2.69	7.29	18.1	39.6	74.2	121
Canis familiaris	CHR_27		0.189	0.438	1.19	3.23	8.57	20.7	44.4	82.4	135
Canis familiaris	CHR_27 (Gene)		0.190	0.443	1.20	3.28	8.76	21.2	45.8	85.4	141
Canis familiaris	CHR_27 (Intergenic)		0.188	0.435	1.18	3.19	8.44	20.3	43.4	80.0	130
Canis familiaris	CHR_27 (Exon)		0.170	0.428	1.12	3.21	9.63	28.5	81.8	—	—
Canis familiaris	CHR_27 (Intron)		0.191	0.440	1.19	3.22	8.48	20.2	42.9	78.7	128
Canis familiaris	CHR_28		0.169	0.406	1.13	3.15	8.73	22.4	51.6	102	175
Canis familiaris	CHR_28 (Gene)		0.172	0.408	1.13	3.14	8.61	21.7	48.7	93.8	157
Canis familiaris	CHR_28 (Intergenic)		0.168	0.404	1.12	3.15	8.79	22.8	53.4	108	187
Canis familiaris	CHR_28 (Exon)		0.199	0.485	1.25	3.59	10.7	31.8	90.5	—	—
Canis familiaris	CHR_28 (Intron)		0.170	0.403	1.11	3.08	8.38	20.9	46.3	87.9	146
Canis familiaris	CHR_29		0.215	0.488	1.31	3.56	9.49	23.2	51.4	98.8	167
Canis familiaris	CHR_29 (Gene)		0.218	0.491	1.32	3.54	9.32	22.2	47.8	89.3	148
Canis familiaris	CHR_29 (Intergenic)		0.213	0.486	1.31	3.56	9.53	23.5	52.5	102	174

next

		L_e (kb)								
Category	SN \ k	2	3	4	5	6	7	8	9	10
Canis familiaris	CHR_29 (Exon)	0.216	0.538	1.43	4.12	12.3	35.3	91.6	—	—
Canis familiaris	CHR_29 (Intron)	0.218	0.488	1.30	3.50	9.12	21.6	45.9	85.0	140
Canis familiaris	CHR_30	0.179	0.421	1.16	3.16	8.50	20.8	45.1	84.5	139
Canis familiaris	CHR_30 (Gene)	0.182	0.425	1.16	3.14	8.29	19.7	41.6	75.5	122
Canis familiaris	CHR_30 (Intergenic)	0.176	0.418	1.15	3.17	8.62	21.5	47.8	91.4	153
Canis familiaris	CHR_30 (Exon)	0.182	0.448	1.17	3.37	10.2	30.5	88.7	—	—
Canis familiaris	CHR_30 (Intron)	0.182	0.420	1.14	3.07	8.02	18.8	38.9	69.7	111
Canis familiaris	CHR_31	0.213	0.486	1.32	3.59	9.61	23.5	51.9	99.4	168
Canis familiaris	CHR_31 (Gene)	0.201	0.464	1.27	3.50	9.44	23.2	51.1	97.3	164
Canis familiaris	CHR_31 (Intergenic)	0.217	0.492	1.33	3.60	9.60	23.4	51.7	99.0	167
Canis familiaris	CHR_31 (Exon)	0.231	0.549	1.36	3.81	11.1	31.1	80.3	—	—
Canis familiaris	CHR_31 (Intron)	0.199	0.458	1.25	3.44	9.20	22.4	48.7	91.8	153
Canis familiaris	CHR_32	0.222	0.496	1.32	3.53	9.24	22.0	47.4	88.4	146
Canis familiaris	CHR_32 (Gene)	0.222	0.497	1.32	3.54	9.26	22.0	47.0	87.5	144
Canis familiaris	CHR_32 (Intergenic)	0.221	0.495	1.32	3.52	9.21	22.0	47.3	88.2	145
Canis familiaris	CHR_32 (Exon)	0.199	0.495	1.31	3.79	11.3	32.9	88.3	—	—
Canis familiaris	CHR_32 (Intron)	0.223	0.495	1.31	3.50	9.07	21.3	45.1	83.2	136
Canis familiaris	CHR_33	0.207	0.473	1.28	3.46	9.16	22.1	48.1	90.7	152
Canis familiaris	CHR_33 (Gene)	0.197	0.451	1.22	3.30	8.66	20.5	43.1	78.4	127
Canis familiaris	CHR_33 (Intergenic)	0.212	0.483	1.30	3.52	9.37	22.8	50.4	96.7	164
Canis familiaris	CHR_33 (Exon)	0.189	0.465	1.23	3.54	10.6	31.0	84.0	—	—
Canis familiaris	CHR_33 (Intron)	0.197	0.448	1.21	3.24	8.43	19.7	40.9	73.6	119
Canis familiaris	CHR_34	0.199	0.461	1.26	3.49	9.55	24.2	55.5	110	191
Canis familiaris	CHR_34 (Gene)	0.197	0.458	1.25	3.46	9.43	23.6	53.3	104	178
Canis familiaris	CHR_34 (Intergenic)	0.199	0.463	1.27	3.50	9.59	24.4	56.2	112	195
Canis familiaris	CHR_34 (Exon)	0.203	0.500	1.30	3.76	11.3	32.9	89.2	—	—
Canis familiaris	CHR_34 (Intron)	0.197	0.454	1.24	3.42	9.25	22.9	51.3	99.0	168
Canis familiaris	CHR_35	0.184	0.429	1.17	3.23	8.72	21.6	48.1	92.7	157
Canis familiaris	CHR_35 (Gene)	0.186	0.432	1.18	3.22	8.62	20.9	45.3	84.7	140
Canis familiaris	CHR_35 (Intergenic)	0.183	0.428	1.17	3.22	8.72	21.7	48.7	94.7	161
Canis familiaris	CHR_35 (Exon)	0.230	0.554	1.40	3.92	11.3	30.0	69.7	—	—
Canis familiaris	CHR_35 (Intron)	0.184	0.425	1.16	3.15	8.36	20.1	43.0	79.5	130
Canis familiaris	CHR_36	0.216	0.492	1.33	3.59	9.50	22.9	49.9	94.7	159
Canis familiaris	CHR_36 (Gene)	0.224	0.505	1.36	3.67	9.71	23.4	51.0	96.9	163

next

			L_e (kb)								
Category	SN	\ k	2	3	4	5	6	7	8	9	10
Canis familiaris	CHR_36 (Intergenic)		0.212	0.484	1.31	3.54	9.37	22.6	49.1	92.9	156
Canis familiaris	CHR_36 (Exon)		0.221	0.541	1.43	4.11	12.2	35.5	95.2	—	—
Canis familiaris	CHR_36 (Intron)		0.223	0.501	1.34	3.60	9.44	22.5	48.3	90.6	151
Canis familiaris	CHR_37		0.201	0.467	1.27	3.49	9.39	23.1	51.3	98.6	167
Canis familiaris	CHR_37 (Gene)		0.204	0.472	1.28	3.51	9.37	22.8	49.6	93.7	157
Canis familiaris	CHR_37 (Intergenic)		0.199	0.464	1.27	3.47	9.38	23.2	51.9	101	172
Canis familiaris	CHR_37 (Exon)		0.202	0.500	1.31	3.77	11.3	33.2	91.2	—	—
Canis familiaris	CHR_37 (Intron)		0.204	0.468	1.27	3.44	9.10	21.8	46.9	87.6	145
Canis familiaris	CHR_38		0.194	0.452	1.24	3.39	9.11	22.4	49.4	94.3	158
Canis familiaris	CHR_38 (Gene)		0.184	0.435	1.20	3.30	8.93	22.0	48.5	92.0	153
Canis familiaris	CHR_38 (Intergenic)		0.198	0.459	1.25	3.42	9.15	22.4	49.4	94.2	158
Canis familiaris	CHR_38 (Exon)		0.189	0.464	1.17	3.29	9.69	27.5	72.7	—	—
Canis familiaris	CHR_38 (Intron)		0.183	0.430	1.18	3.23	8.66	21.1	45.6	85.3	140
Canis familiaris	CHR_X		0.193	0.447	1.22	3.33	8.99	22.3	49.6	94.4	155
Canis familiaris	CHR_X (Gene)		0.193	0.453	1.24	3.44	9.42	23.9	54.7	108	182
Canis familiaris	CHR_X (Intergenic)		0.192	0.444	1.21	3.28	8.81	21.7	47.6	89.4	145
Canis familiaris	CHR_X (Exon)		0.187	0.471	1.24	3.59	10.9	32.9	97.7	266	—
Canis familiaris	CHR_X (Intron)		0.193	0.450	1.23	3.38	9.19	23.1	52.0	101	169
Danio rerio	CHR_01		0.332	0.694	1.82	4.63	11.0	22.8	40.1	58.5	74.8
Danio rerio	CHR_01 (Gene)		0.328	0.683	1.79	4.58	11.1	23.6	42.8	64.3	83.8
Danio rerio	CHR_01 (Intergenic)		0.336	0.704	1.85	4.66	11.0	22.2	38.1	54.3	68.6
Danio rerio	CHR_01 (Exon)		0.281	0.542	1.34	3.76	11.1	33.2	98.0	268	—
Danio rerio	CHR_01 (Intron)		0.323	0.675	1.76	4.44	10.5	21.8	38.6	57.0	73.5
Danio rerio	CHR_02		0.326	0.691	1.82	4.72	11.6	25.1	46.1	69.7	91.0
Danio rerio	CHR_02 (Gene)		0.320	0.674	1.78	4.63	11.6	25.7	49.2	77.0	103
Danio rerio	CHR_02 (Intergenic)		0.331	0.702	1.86	4.78	11.6	24.4	43.6	64.2	82.3
Danio rerio	CHR_02 (Exon)		0.272	0.538	1.33	3.74	11.1	33.0	97.1	261	—
Danio rerio	CHR_02 (Intron)		0.315	0.666	1.75	4.49	11.0	23.7	44.0	67.5	89.0
Danio rerio	CHR_03		0.325	0.685	1.80	4.67	11.4	24.7	45.4	68.9	90.4
Danio rerio	CHR_03 (Gene)		0.318	0.667	1.76	4.59	11.5	25.7	49.3	78.1	106
Danio rerio	CHR_03 (Intergenic)		0.331	0.698	1.84	4.72	11.4	23.9	42.6	62.9	81.0
Danio rerio	CHR_03 (Exon)		0.266	0.524	1.30	3.66	10.7	31.2	87.5	215	—
Danio rerio	CHR_03 (Intron)		0.313	0.659	1.73	4.44	10.9	23.5	43.9	67.9	90.6
Danio rerio	CHR_04		0.335	0.702	1.85	4.75	11.7	25.4	47.6	73.3	96.9

next

		L_e (kb)								
Category	SN \ k	2	3	4	5	6	7	8	9	10
Danio rerio	CHR_04 (Gene)	0.335	0.697	1.83	4.71	11.7	26.0	50.1	79.1	106
Danio rerio	CHR_04 (Intergenic)	0.335	0.706	1.86	4.77	11.6	24.9	45.6	68.7	89.6
Danio rerio	CHR_04 (Exon)	0.284	0.538	1.32	3.68	10.8	31.3	87.4	213	—
Danio rerio	CHR_04 (Intron)	0.331	0.692	1.81	4.58	11.1	23.9	44.6	68.8	91.2
Danio rerio	CHR_05	0.322	0.677	1.78	4.61	11.3	24.4	45.0	68.2	89.4
Danio rerio	CHR_05 (Gene)	0.319	0.668	1.76	4.58	11.4	25.2	47.9	75.0	100
Danio rerio	CHR_05 (Intergenic)	0.325	0.683	1.80	4.63	11.2	23.8	42.7	63.3	81.8
Danio rerio	CHR_05 (Exon)	0.274	0.546	1.37	3.88	11.6	34.7	104	287	—
Danio rerio	CHR_05 (Intron)	0.315	0.661	1.73	4.44	10.8	23.2	43.0	66.0	87.4
Danio rerio	CHR_06	0.322	0.678	1.78	4.57	11.1	23.9	43.7	66.1	86.5
Danio rerio	CHR_06 (Gene)	0.321	0.674	1.77	4.59	11.4	25.2	47.8	74.6	99.8
Danio rerio	CHR_06 (Intergenic)	0.323	0.683	1.79	4.55	10.9	22.6	40.0	58.8	75.5
Danio rerio	CHR_06 (Exon)	0.270	0.540	1.34	3.79	11.2	33.3	96.0	246	—
Danio rerio	CHR_06 (Intron)	0.316	0.665	1.74	4.44	10.8	23.3	43.2	66.2	87.6
Danio rerio	CHR_07	0.324	0.675	1.78	4.55	11.0	23.3	42.1	62.7	81.4
Danio rerio	CHR_07 (Gene)	0.318	0.664	1.75	4.55	11.3	24.9	47.4	73.8	98.5
Danio rerio	CHR_07 (Intergenic)	0.329	0.683	1.79	4.54	10.8	22.1	38.4	55.6	70.9
Danio rerio	CHR_07 (Exon)	0.274	0.550	1.37	3.88	11.5	34.6	103	284	—
Danio rerio	CHR_07 (Intron)	0.315	0.657	1.72	4.41	10.7	23.1	42.7	65.2	86.0
Danio rerio	CHR_08	0.332	0.694	1.83	4.68	11.3	24.1	43.7	65.4	85.0
Danio rerio	CHR_08 (Gene)	0.325	0.679	1.78	4.61	11.4	24.9	46.8	72.5	96.5
Danio rerio	CHR_08 (Intergenic)	0.338	0.710	1.87	4.73	11.3	23.2	40.5	58.6	74.5
Danio rerio	CHR_08 (Exon)	0.280	0.550	1.36	3.83	11.3	33.3	95.4	242	—
Danio rerio	CHR_08 (Intron)	0.320	0.670	1.75	4.47	10.8	23.1	42.6	64.9	85.5
Danio rerio	CHR_09	0.326	0.689	1.82	4.68	11.4	24.5	44.7	67.4	66.0
Danio rerio	CHR_09 (Gene)	0.320	0.678	1.79	4.68	11.7	26.1	50.2	79.7	108
Danio rerio	CHR_09 (Intergenic)	0.332	0.698	1.84	4.68	11.2	23.1	40.5	58.8	75.0
Danio rerio	CHR_09 (Exon)	0.260	0.542	1.36	3.88	11.6	35.0	104	281	—
Danio rerio	CHR_09 (Intron)	0.317	0.671	1.76	4.52	11.0	23.9	44.8	69.7	93.3
Danio rerio	CHR_10	0.331	0.694	1.83	4.71	11.5	24.8	45.7	69.3	90.6
Danio rerio	CHR_10 (Gene)	0.330	0.691	1.82	4.71	11.6	25.5	47.8	73.7	97.6
Danio rerio	CHR_10 (Intergenic)	0.331	0.695	1.83	4.69	11.4	24.2	43.8	65.4	84.7
Danio rerio	CHR_10 (Exon)	0.291	0.575	1.42	4.02	11.9	35.2	101	255	—
Danio rerio	CHR_10 (Intron)	0.325	0.682	1.79	4.55	11.0	23.4	42.7	64.6	84.5

next

			L_e (kb)								
Category	SN	\ k	2	3	4	5	6	7	8	9	10
Danio rerio	CHR_11		0.336	0.704	1.85	4.70	11.2	23.3	41.0	60.0	76.9
Danio rerio	CHR_11 (Gene)		0.335	0.701	1.84	4.74	11.5	24.7	45.0	67.7	88.1
Danio rerio	CHR_11 (Intergenic)		0.336	0.707	1.85	4.67	11.0	22.3	38.4	55.1	69.9
Danio rerio	CHR_11 (Exon)		0.276	0.564	1.40	3.96	11.7	34.5	97.7	—	—
Danio rerio	CHR_11 (Intron)		0.331	0.694	1.81	4.60	11.0	22.9	40.8	60.4	77.8
Danio rerio	CHR_12		0.330	0.696	1.83	4.71	11.5	24.5	44.6	66.9	87.0
Danio rerio	CHR_12 (Gene)		0.320	0.676	1.79	4.68	11.8	26.5	51.6	82.4	112
Danio rerio	CHR_12 (Intergenic)		0.338	0.712	1.87	4.73	11.2	22.8	39.5	56.7	71.8
Danio rerio	CHR_12 (Exon)		0.257	0.528	1.32	3.75	11.1	33.1	95.9	246	—
Danio rerio	CHR_12 (Intron)		0.317	0.669	1.76	4.54	11.2	24.5	46.4	72.5	97.3
Danio rerio	CHR_13		0.335	0.699	1.84	4.70	11.4	24.4	44.4	66.8	87.4
Danio rerio	CHR_13 (Gene)		0.334	0.696	1.83	4.75	11.8	26.1	49.8	78.6	106
Danio rerio	CHR_13 (Intergenic)		0.335	0.701	1.84	4.66	11.1	22.9	40.2	58.5	74.8
Danio rerio	CHR_13 (Exon)		0.276	0.544	1.35	3.83	11.4	34.0	99.8	264	—
Danio rerio	CHR_13 (Intron)		0.330	0.690	1.81	4.60	11.2	24.0	44.5	68.6	91.5
Danio rerio	CHR_14		0.331	0.702	1.86	4.78	11.7	24.9	45.1	67.3	87.1
Danio rerio	CHR_14 (Gene)		0.328	0.691	1.83	4.75	11.8	26.1	49.3	76.6	102
Danio rerio	CHR_14 (Intergenic)		0.333	0.709	1.87	4.79	11.5	24.1	42.5	61.9	79.0
Danio rerio	CHR_14 (Exon)		0.307	0.590	1.48	4.18	12.4	36.6	105	268	—
Danio rerio	CHR_14 (Intron)		0.321	0.681	1.79	4.59	11.2	24.0	44.1	67.1	88.3
Danio rerio	CHR_15		0.334	0.699	1.84	4.73	11.5	24.5	44.5	66.6	86.5
Danio rerio	CHR_15 (Gene)		0.331	0.690	1.81	4.70	11.6	25.5	48.0	74.2	98.5
Danio rerio	CHR_15 (Intergenic)		0.336	0.705	1.86	4.74	11.4	23.7	42.0	61.5	78.6
Danio rerio	CHR_15 (Exon)		0.272	0.544	1.37	3.88	11.4	33.5	93.4	224	—
Danio rerio	CHR_15 (Intron)		0.328	0.685	1.79	4.55	11.0	23.3	42.6	64.3	84.3
Danio rerio	CHR_16		0.323	0.683	1.80	4.65	11.4	24.5	45.0	68.1	88.9
Danio rerio	CHR_16 (Gene)		0.318	0.672	1.77	4.63	11.6	25.9	50.0	79.3	107
Danio rerio	CHR_16 (Intergenic)		0.327	0.691	1.82	4.66	11.2	23.4	41.5	60.7	77.6
Danio rerio	CHR_16 (Exon)		0.268	0.545	1.37	3.89	11.6	34.6	101	266	—
Danio rerio	CHR_16 (Intron)		0.314	0.664	1.74	4.48	10.9	23.8	44.6	69.2	92.3
Danio rerio	CHR_17		0.326	0.684	1.80	4.63	11.2	23.8	43.0	64.0	82.7
Danio rerio	CHR_17 (Gene)		0.325	0.678	1.78	4.61	11.3	24.5	45.3	69.0	90.5
Danio rerio	CHR_17 (Intergenic)		0.326	0.688	1.81	4.63	11.1	23.2	40.9	59.6	76.0
Danio rerio	CHR_17 (Exon)		0.263	0.516	1.28	3.61	10.7	32.0	93.5	246	—

next

		L_e (kb)								
Category	SN \ k	2	3	4	5	6	7	8	9	10
Danio rerio	CHR_17 (Intron)	0.322	0.673	1.76	4.47	10.7	22.6	40.6	60.6	78.7
Danio rerio	CHR_18	0.334	0.700	1.84	4.71	11.4	24.0	42.8	63.1	81.0
Danio rerio	CHR_18 (Gene)	0.331	0.689	1.81	4.68	11.5	25.0	46.2	70.3	92.1
Danio rerio	CHR_18 (Intergenic)	0.336	0.707	1.86	4.73	11.3	23.3	40.5	58.4	74.1
Danio rerio	CHR_18 (Exon)	0.271	0.543	1.35	3.83	11.4	33.9	98.9	259	—
Danio rerio	CHR_18 (Intron)	0.327	0.683	1.79	4.54	10.9	23.1	41.6	61.9	80.2
Danio rerio	CHR_19	0.333	0.704	1.85	4.75	11.5	24.2	43.2	63.9	82.5
Danio rerio	CHR_19 (Gene)	0.333	0.703	1.85	4.75	11.6	24.9	45.9	69.7	91.4
Danio rerio	CHR_19 (Intergenic)	0.332	0.704	1.86	4.74	11.3	23.6	41.4	60.3	77.0
Danio rerio	CHR_19 (Exon)	0.269	0.547	1.36	3.84	11.4	33.8	98.7	262	—
Danio rerio	CHR_19 (Intron)	0.330	0.697	1.83	4.62	11.0	23.1	41.4	61.7	80.1
Danio rerio	CHR_20	0.331	0.695	1.83	4.72	11.5	24.3	43.6	64.5	82.8
Danio rerio	CHR_20 (Gene)	0.331	0.692	1.82	4.73	11.7	25.5	47.4	72.4	94.9
Danio rerio	CHR_20 (Intergenic)	0.330	0.696	1.84	4.70	11.3	23.4	40.9	59.1	74.9
Danio rerio	CHR_20 (Exon)	0.273	0.541	1.34	3.79	11.3	33.5	98.2	262	—
Danio rerio	CHR_20 (Intron)	0.327	0.685	1.80	4.58	11.0	23.3	42.1	62.8	81.4
Danio rerio	CHR_21	0.331	0.695	1.84	4.75	11.7	25.4	47.0	71.8	94.5
Danio rerio	CHR_21 (Gene)	0.331	0.692	1.83	4.75	11.8	26.0	49.3	76.7	102
Danio rerio	CHR_21 (Intergenic)	0.331	0.698	1.84	4.75	11.6	24.9	45.3	68.0	88.7
Danio rerio	CHR_21 (Exon)	0.266	0.520	1.29	3.61	10.6	30.8	85.8	207	—
Danio rerio	CHR_21 (Intron)	0.328	0.688	1.81	4.63	11.3	24.2	44.7	68.3	90.1
Danio rerio	CHR_22	0.341	0.718	1.89	4.88	11.9	25.3	45.7	67.8	87.4
Danio rerio	CHR_22 (Gene)	0.335	0.706	1.86	4.83	12.0	26.0	48.1	72.8	94.9
Danio rerio	CHR_22 (Intergenic)	0.345	0.726	1.91	4.90	11.8	24.8	43.8	64.0	81.7
Danio rerio	CHR_22 (Exon)	0.273	0.538	1.33	3.73	10.9	31.8	88.7	215	—
Danio rerio	CHR_22 (Intron)	0.332	0.699	1.83	4.66	11.2	23.4	41.8	61.6	79.0
Danio rerio	CHR_23	0.328	0.690	1.81	4.67	11.4	24.6	45.2	68.5	90.0
Danio rerio	CHR_23 (Gene)	0.325	0.680	1.79	4.66	11.6	25.9	49.9	79.2	107
Danio rerio	CHR_23 (Intergenic)	0.331	0.697	1.83	4.67	11.2	23.6	41.9	61.7	79.3
Danio rerio	CHR_23 (Exon)	0.273	0.539	1.34	3.78	11.2	33.3	97.7	262	—
Danio rerio	CHR_23 (Intron)	0.320	0.672	1.76	4.50	11.0	23.7	44.5	68.9	92.2
Danio rerio	CHR_24	0.332	0.697	1.83	4.65	11.1	23.2	41.1	60.4	77.6
Danio rerio	CHR_24 (Gene)	0.322	0.682	1.80	4.63	11.4	24.6	45.9	70.5	93.1
Danio rerio	CHR_24 (Intergenic)	0.339	0.709	1.86	4.64	10.9	22.0	37.6	53.7	67.6

next

		L_e (kb)								
Category	SN \ k	2	3	4	5	6	7	8	9	10
Danio rerio	CHR_24 (Exon)	0.274	0.559	1.40	3.97	11.8	34.6	97.1	—	—
Danio rerio	CHR_24 (Intron)	0.319	0.675	1.77	4.49	10.8	22.7	41.3	62.1	81.1
Danio rerio	CHR_25	0.338	0.712	1.88	4.81	11.6	24.6	44.0	65.2	84.1
Danio rerio	CHR_25 (Gene)	0.339	0.709	1.86	4.80	11.7	24.9	45.2	67.6	87.8
Danio rerio	CHR_25 (Intergenic)	0.337	0.714	1.89	4.81	11.6	24.3	43.1	63.4	81.3
Danio rerio	CHR_25 (Exon)	0.280	0.570	1.43	4.04	11.9	34.9	96.9	—	—
Danio rerio	CHR_25 (Intron)	0.336	0.702	1.84	4.65	11.1	22.9	40.5	59.4	76.3
Gallus gallus	CHR_01	0.194	0.445	1.19	3.37	9.64	27.0	72.4	178	386
Gallus gallus	CHR_01 (Gene)	0.193	0.447	1.20	3.40	9.84	28.1	78.2	203	477
Gallus gallus	CHR_01 (Intergenic)	0.194	0.443	1.18	3.33	9.47	26.0	67.9	159	329
Gallus gallus	CHR_01 (Exon)	0.194	0.456	1.17	3.36	10.1	31.2	97.2	298	834
Gallus gallus	CHR_01 (Intron)	0.193	0.445	1.19	3.37	9.71	27.5	75.6	194	447
Gallus gallus	CHR_02	0.193	0.444	1.19	3.35	9.62	27.0	72.9	181	403
Gallus gallus	CHR_02 (Gene)	0.196	0.451	1.21	3.41	9.84	27.9	77.2	199	466
Gallus gallus	CHR_02 (Intergenic)	0.191	0.438	1.17	3.31	9.44	26.2	69.6	168	359
Gallus gallus	CHR_02 (Exon)	0.198	0.466	1.20	3.45	10.4	31.9	98.7	296	—
Gallus gallus	CHR_02 (Intron)	0.195	0.448	1.20	3.38	9.71	27.4	75.0	192	444
Gallus gallus	CHR_03	0.190	0.440	1.18	3.34	9.62	27.2	74.2	188	426
Gallus gallus	CHR_03 (Gene)	0.193	0.448	1.20	3.40	9.82	27.9	77.4	200	471
Gallus gallus	CHR_03 (Intergenic)	0.187	0.432	1.16	3.28	9.42	26.4	71.3	176	387
Gallus gallus	CHR_03 (Exon)	0.190	0.449	1.15	3.27	9.86	30.2	93.4	281	—
Gallus gallus	CHR_03 (Intron)	0.192	0.446	1.19	3.37	9.68	27.3	74.6	190	439
Gallus gallus	CHR_04	0.189	0.440	1.18	3.34	9.60	27.1	73.9	187	427
Gallus gallus	CHR_04 (Gene)	0.186	0.436	1.17	3.33	9.68	27.8	78.5	209	509
Gallus gallus	CHR_04 (Intergenic)	0.191	0.441	1.18	3.33	9.51	26.4	70.4	172	374
Gallus gallus	CHR_04 (Exon)	0.193	0.456	1.16	3.31	9.94	30.3	93.0	275	—
Gallus gallus	CHR_04 (Intron)	0.185	0.433	1.16	3.29	9.52	27.2	75.8	199	478
Gallus gallus	CHR_05	0.183	0.430	1.16	3.29	9.60	27.7	78.0	207	502
Gallus gallus	CHR_05 (Gene)	0.184	0.431	1.16	3.31	9.66	28.1	80.4	219	549
Gallus gallus	CHR_05 (Intergenic)	0.183	0.428	1.15	3.28	9.51	27.1	75.3	194	451
Gallus gallus	CHR_05 (Exon)	0.186	0.437	1.11	3.17	9.54	29.0	88.7	259	—
Gallus gallus	CHR_05 (Intron)	0.183	0.428	1.15	3.27	9.51	27.4	77.4	207	510
Gallus gallus	CHR_06	0.178	0.422	1.14	3.25	9.51	27.7	79.2	215	534
Gallus gallus	CHR_06 (Gene)	0.181	0.428	1.15	3.29	9.64	28.1	80.4	219	544

next

		L_e (kb)								
Category	SN \ k	2	3	4	5	6	7	8	9	10
Gallus gallus	CHR_06 (Intergenic)	0.174	0.415	1.12	3.20	9.36	27.2	77.4	208	504
Gallus gallus	CHR_06 (Exon)	0.186	0.440	1.12	3.19	9.57	28.9	86.7	240	—
Gallus gallus	CHR_06 (Intron)	0.181	0.426	1.15	3.27	9.52	27.5	77.7	208	506
Gallus gallus	CHR_07	0.180	0.427	1.15	3.28	9.58	27.8	79.4	215	534
Gallus gallus	CHR_07 (Gene)	0.184	0.434	1.17	3.32	9.73	28.4	82.0	226	570
Gallus gallus	CHR_07 (Intergenic)	0.176	0.420	1.13	3.23	9.42	27.2	76.6	203	486
Gallus gallus	CHR_07 (Exon)	0.194	0.457	1.17	3.32	10.0	30.3	91.2	253	—
Gallus gallus	CHR_07 (Intron)	0.182	0.428	1.15	3.28	9.53	27.6	78.7	213	530
Gallus gallus	CHR_08	0.178	0.422	1.14	3.24	9.47	27.4	77.6	206	496
Gallus gallus	CHR_08 (Gene)	0.178	0.421	1.13	3.24	9.52	27.9	80.8	223	563
Gallus gallus	CHR_08 (Intergenic)	0.178	0.422	1.14	3.24	9.39	26.7	73.3	185	414
Gallus gallus	CHR_08 (Exon)	0.191	0.446	1.12	3.16	9.43	28.3	84.5	232	—
Gallus gallus	CHR_08 (Intron)	0.176	0.417	1.12	3.20	9.36	27.2	77.6	211	522
Gallus gallus	CHR_09	0.168	0.401	1.08	3.10	9.13	27.0	79.2	224	585
Gallus gallus	CHR_09 (Gene)	0.171	0.404	1.09	3.11	9.20	27.4	81.2	233	619
Gallus gallus	CHR_09 (Intergenic)	0.166	0.398	1.07	3.07	9.03	26.5	76.5	210	525
Gallus gallus	CHR_09 (Exon)	0.184	0.434	1.08	3.05	9.08	27.1	80.0	—	—
Gallus gallus	CHR_09 (Intron)	0.169	0.399	1.08	3.07	9.04	26.7	78.5	222	578
Gallus gallus	CHR_10	0.175	0.413	1.11	3.18	9.35	27.4	79.1	217	546
Gallus gallus	CHR_10 (Gene)	0.178	0.420	1.13	3.22	9.50	28.0	81.9	229	588
Gallus gallus	CHR_10 (Intergenic)	0.170	0.406	1.10	3.14	9.17	26.6	75.4	200	476
Gallus gallus	CHR_10 (Exon)	0.193	0.450	1.13	3.18	9.47	28.3	83.5	—	—
Gallus gallus	CHR_10 (Intron)	0.176	0.415	1.12	3.19	9.33	27.3	79.0	218	551
Gallus gallus	CHR_11	0.177	0.420	1.13	3.24	9.51	27.7	79.5	216	539
Gallus gallus	CHR_11 (Gene)	0.173	0.411	1.10	3.16	9.34	27.6	81.1	229	589
Gallus gallus	CHR_11 (Intergenic)	0.180	0.427	1.15	3.29	9.56	27.5	76.8	201	473
Gallus gallus	CHR_11 (Exon)	0.187	0.443	1.09	3.05	9.02	26.5	76.5	—	—
Gallus gallus	CHR_11 (Intron)	0.169	0.404	1.09	3.11	9.13	26.8	78.0	217	548
Gallus gallus	CHR_12	0.164	0.393	1.06	3.03	8.94	26.5	78.1	222	582
Gallus gallus	CHR_12 (Gene)	0.168	0.400	1.08	3.08	9.09	27.0	79.4	225	588
Gallus gallus	CHR_12 (Intergenic)	0.159	0.385	1.04	2.97	8.74	25.8	75.6	211	536
Gallus gallus	CHR_12 (Exon)	0.187	0.439	1.08	3.02	8.91	26.3	76.3	—	—
Gallus gallus	CHR_12 (Intron)	0.166	0.395	1.06	3.04	8.95	26.4	77.0	216	554
Gallus gallus	CHR_13	0.158	0.378	1.01	2.90	8.57	25.5	75.4	216	573

next

			L_e (kb)							
Category	SN \ k	2	3	4	5	6	7	8	9	10
Gallus gallus	CHR_13 (Gene)	0.160	0.380	1.02	2.92	8.64	25.8	77.0	223	595
Gallus gallus	CHR_13 (Intergenic)	0.157	0.375	1.01	2.88	8.48	25.0	73.1	204	517
Gallus gallus	CHR_13 (Exon)	0.181	0.426	1.05	2.92	8.58	25.1	72.5	—	—
Gallus gallus	CHR_13 (Intron)	0.157	0.374	1.00	2.87	8.47	25.2	74.5	212	558
Gallus gallus	CHR_14	0.157	0.376	1.01	2.90	8.56	25.4	74.9	212	557
Gallus gallus	CHR_14 (Gene)	0.158	0.380	1.02	2.92	8.62	25.7	76.2	219	580
Gallus gallus	CHR_14 (Intergenic)	0.154	0.372	1.00	2.87	8.47	24.9	72.3	199	493
Gallus gallus	CHR_14 (Exon)	0.172	0.409	1.01	2.84	8.38	24.9	74.0	205	—
Gallus gallus	CHR_14 (Intron)	0.156	0.375	1.01	2.88	8.47	25.0	73.5	207	538
Gallus gallus	CHR_15	0.153	0.368	0.986	2.82	8.35	24.9	74.5	216	584
Gallus gallus	CHR_15 (Gene)	0.160	0.382	1.02	2.92	8.66	26.0	78.3	229	621
Gallus gallus	CHR_15 (Intergenic)	0.145	0.352	0.945	2.70	7.94	23.5	69.1	193	489
Gallus gallus	CHR_15 (Exon)	0.176	0.416	1.03	2.89	8.54	25.2	73.3	—	—
Gallus gallus	CHR_15 (Intron)	0.157	0.374	1.00	2.87	8.47	25.2	75.1	216	573
Gallus gallus	CHR_16	0.128	0.311	0.804	2.23	6.20	16.5	39.3	—	—
Gallus gallus	CHR_16 (Gene)	0.118	0.284	0.715	1.93	5.21	13.1	—	—	—
Gallus gallus	CHR_16 (Intergenic)	0.138	0.340	0.900	2.52	6.98	17.9	—	—	—
Gallus gallus	CHR_16 (Exon)	0.184	0.431	0.949	2.38	5.79	12.1	—	—	—
Gallus gallus	CHR_16 (Intron)	0.095	0.229	0.588	1.58	4.16	10.1	—	—	—
Gallus gallus	CHR_17	0.136	0.329	0.874	2.49	7.32	21.8	65.3	191	522
Gallus gallus	CHR_17 (Gene)	0.140	0.337	0.894	2.55	7.52	22.5	67.8	199	544
Gallus gallus	CHR_17 (Intergenic)	0.130	0.317	0.845	2.40	7.02	20.7	60.7	170	—
Gallus gallus	CHR_17 (Exon)	0.165	0.388	0.944	2.62	7.66	22.3	64.2	—	—
Gallus gallus	CHR_17 (Intron)	0.137	0.330	0.878	2.50	7.35	21.9	65.5	190	506
Gallus gallus	CHR_18	0.143	0.348	0.932	2.67	7.89	23.7	71.4	211	582
Gallus gallus	CHR_18 (Gene)	0.150	0.362	0.968	2.77	8.21	24.6	74.2	217	577
Gallus gallus	CHR_18 (Intergenic)	0.136	0.334	0.896	2.56	7.55	22.6	67.4	194	510
Gallus gallus	CHR_18 (Exon)	0.156	0.371	0.904	2.50	7.28	20.9	57.4	—	—
Gallus gallus	CHR_18 (Intron)	0.149	0.359	0.963	2.75	8.13	24.3	72.4	208	—
Gallus gallus	CHR_19	0.147	0.355	0.943	2.69	7.95	23.8	71.8	212	591
Gallus gallus	CHR_19 (Gene)	0.148	0.357	0.947	2.70	8.00	24.1	72.9	216	596
Gallus gallus	CHR_19 (Intergenic)	0.144	0.350	0.934	2.66	7.80	23.1	68.2	192	—
Gallus gallus	CHR_19 (Exon)	0.178	0.417	1.02	2.85	8.35	24.5	70.6	—	—
Gallus gallus	CHR_19 (Intron)	0.144	0.348	0.928	2.64	7.79	23.3	70.0	205	555

next

			L_e (kb)								
Category	SN	\ k	2	3	4	5	6	7	8	9	10
Gallus gallus	CHR_20		0.146	0.351	0.938	2.68	7.92	23.7	70.6	205	556
Gallus gallus	CHR_20 (Gene)		0.148	0.354	0.944	2.70	8.01	24.0	72.1	211	571
Gallus gallus	CHR_20 (Intergenic)		0.144	0.347	0.930	2.65	7.82	23.2	68.3	193	496
Gallus gallus	CHR_20 (Exon)		0.170	0.401	0.976	2.71	7.91	23.1	66.7	—	—
Gallus gallus	CHR_20 (Intron)		0.144	0.347	0.929	2.66	7.84	23.4	69.6	201	532
Gallus gallus	CHR_21		0.140	0.345	0.921	2.63	7.74	23.2	69.5	203	549
Gallus gallus	CHR_21 (Gene)		0.143	0.350	0.933	2.66	7.88	23.7	71.3	208	—
Gallus gallus	CHR_21 (Intergenic)		0.135	0.338	0.903	2.56	7.50	22.2	65.0	181	—
Gallus gallus	CHR_21 (Exon)		0.167	0.397	0.974	2.71	7.93	23.1	65.1	—	—
Gallus gallus	CHR_21 (Intron)		0.138	0.341	0.913	2.60	7.66	22.9	68.1	195	—
Gallus gallus	CHR_22		0.148	0.379	1.03	2.95	8.66	25.3	72.1	191	—
Gallus gallus	CHR_22 (Gene)		0.147	0.376	1.02	2.92	8.55	24.9	70.1	180	—
Gallus gallus	CHR_22 (Intergenic)		0.150	0.384	1.04	2.99	8.72	25.2	69.0	—	—
Gallus gallus	CHR_22 (Exon)		0.169	0.416	1.03	2.83	7.90	20.2	—	—	—
Gallus gallus	CHR_22 (Intron)		0.145	0.371	1.01	2.89	8.42	24.4	68.1	173	—
Gallus gallus	CHR_23		0.129	0.312	0.812	2.29	6.66	19.7	59.0	173	476
Gallus gallus	CHR_23 (Gene)		0.135	0.321	0.832	2.35	6.85	20.3	60.6	175	—
Gallus gallus	CHR_23 (Intergenic)		0.123	0.304	0.792	2.22	6.44	19.0	55.9	159	—
Gallus gallus	CHR_23 (Exon)		0.171	0.400	0.950	2.59	7.41	20.9	57.3	—	—
Gallus gallus	CHR_23 (Intron)		0.127	0.304	0.796	2.25	6.54	19.3	57.2	162	—
Gallus gallus	CHR_24		0.122	0.304	0.800	2.26	6.60	19.6	58.6	172	472
Gallus gallus	CHR_24 (Gene)		0.128	0.316	0.832	2.36	6.91	20.6	61.6	179	—
Gallus gallus	CHR_24 (Intergenic)		0.116	0.292	0.766	2.15	6.26	18.4	54.3	155	—
Gallus gallus	CHR_24 (Exon)		0.163	0.384	0.915	2.50	7.20	20.5	55.6	—	—
Gallus gallus	CHR_24 (Intron)		0.123	0.307	0.812	2.30	6.72	20.0	59.4	170	—
Gallus gallus	CHR_26		0.120	0.292	0.751	2.09	6.02	17.7	52.3	152	—
Gallus gallus	CHR_26 (Gene)		0.123	0.298	0.766	2.14	6.20	18.3	54.1	156	—
Gallus gallus	CHR_26 (Intergenic)		0.117	0.285	0.729	2.02	5.76	16.7	48.2	134	—
Gallus gallus	CHR_26 (Exon)		0.169	0.399	0.934	2.54	7.26	20.4	55.1	—	—
Gallus gallus	CHR_26 (Intron)		0.115	0.281	0.729	2.04	5.88	17.2	50.5	143	—
Gallus gallus	CHR_27		0.132	0.321	0.840	2.38	6.96	20.7	61.7	178	—
Gallus gallus	CHR_27 (Gene)		0.134	0.326	0.848	2.40	7.00	20.7	61.5	174	—
Gallus gallus	CHR_27 (Intergenic)		0.128	0.314	0.826	2.34	6.82	20.1	58.4	158	—
Gallus gallus	CHR_27 (Exon)		0.174	0.412	0.948	2.56	7.25	20.1	52.9	—	—

next

		L_e (kb)								
Category	SN \ k	2	3	4	5	6	7	8	9	10
Gallus gallus	CHR_27 (Intron)	0.125	0.306	0.808	2.29	6.66	19.6	57.5	160	—
Gallus gallus	CHR_28	0.142	0.340	0.901	2.56	7.47	21.9	63.3	173	—
Gallus gallus	CHR_28 (Gene)	0.136	0.325	0.854	2.42	7.11	21.2	63.0	180	—
Gallus gallus	CHR_28 (Intergenic)	0.151	0.362	0.968	2.74	7.84	21.9	57.7	134	—
Gallus gallus	CHR_28 (Exon)	0.174	0.409	0.967	2.63	7.55	21.3	57.7	—	—
Gallus gallus	CHR_28 (Intron)	0.129	0.310	0.822	2.33	6.83	20.2	59.6	167	—
Gallus gallus	CHR_32	0.116	0.277	0.709	1.97	5.66	16.5	48.1	—	—
Gallus gallus	CHR_32 (Gene)	0.113	0.270	0.686	1.89	5.41	15.7	45.3	—	—
Gallus gallus	CHR_32 (Intergenic)	0.120	0.292	0.760	2.12	6.08	17.2	45.6	—	—
Gallus gallus	CHR_32 (Exon)	0.152	0.353	0.800	2.11	5.81	15.2	—	—	—
Gallus gallus	CHR_32 (Intron)	0.105	0.253	0.650	1.80	5.13	14.9	42.4	—	—
Gallus gallus	CHR_W	0.196	0.441	1.17	3.27	9.24	25.1	64.4	147	292
Gallus gallus	CHR_W (Gene)	0.209	0.465	1.23	3.43	9.67	26.3	67.2	152	—
Gallus gallus	CHR_W (Intergenic)	0.187	0.423	1.13	3.16	8.88	23.9	60.5	135	—
Gallus gallus	CHR_W (Exon)	0.202	0.465	1.19	3.30	9.30	24.2	—	—	—
Gallus gallus	CHR_W (Intron)	0.208	0.462	1.22	3.39	9.50	25.5	64.4	143	—
Gallus gallus	CHR_Z	0.191	0.431	1.15	3.24	9.29	26.0	70.1	172	374
Gallus gallus	CHR_Z (Gene)	0.194	0.437	1.16	3.27	9.38	26.4	71.7	179	400
Gallus gallus	CHR_Z (Intergenic)	0.188	0.426	1.13	3.21	9.19	25.7	68.6	165	350
Gallus gallus	CHR_Z (Exon)	0.187	0.434	1.12	3.20	9.61	29.2	87.8	242	—
Gallus gallus	CHR_Z (Intron)	0.193	0.435	1.15	3.23	9.21	25.7	68.8	169	371
Homo sapiens	CHR_01	0.182	0.439	1.21	3.35	9.21	23.2	51.2	94.3	150
Homo sapiens	CHR_01 (Gene)	0.180	0.433	1.18	3.28	8.90	22.0	47.1	84.4	131
Homo sapiens	CHR_01 (Intergenic)	0.183	0.443	1.22	3.40	9.43	24.1	54.6	103	166
Homo sapiens	CHR_01 (Exon)	0.166	0.403	1.10	2.99	7.91	18.6	37.8	63.8	95.2
Homo sapiens	CHR_01 (Intron)	0.176	0.425	1.16	3.21	8.66	21.2	44.9	79.3	122
Homo sapiens	CHR_02	0.196	0.467	1.28	3.59	10.0	26.1	60.9	120	201
Homo sapiens	CHR_02 (Gene)	0.196	0.465	1.27	3.53	9.72	24.6	55.1	103	167
Homo sapiens	CHR_02 (Intergenic)	0.196	0.469	1.29	3.62	10.2	27.0	64.8	131	226
Homo sapiens	CHR_02 (Exon)	0.182	0.437	1.19	3.32	9.07	22.6	49.1	88.8	139
Homo sapiens	CHR_02 (Intron)	0.191	0.456	1.25	3.49	9.65	24.6	55.6	105	171
Homo sapiens	CHR_03	0.200	0.477	1.30	3.65	10.2	26.6	62.2	122	206
Homo sapiens	CHR_03 (Gene)	0.194	0.463	1.26	3.53	9.74	24.8	55.8	106	172
Homo sapiens	CHR_03 (Intergenic)	0.205	0.486	1.33	3.74	10.5	27.8	66.8	136	235

next

			L_e (kb)							
Category	SN $\backslash$ k	2	3	4	5	6	7	8	9	10
Homo sapiens	CHR_03 (Exon)	0.179	0.431	1.18	3.27	8.92	22.2	48.1	86.7	—
Homo sapiens	CHR_03 (Intron)	0.189	0.454	1.24	3.48	9.64	24.7	56.1	107	174
Homo sapiens	CHR_04	0.223	0.520	1.41	3.94	11.0	29.1	69.9	143	251
Homo sapiens	CHR_04 (Gene)	0.219	0.510	1.39	3.86	10.7	27.5	63.7	125	210
Homo sapiens	CHR_04 (Intergenic)	0.225	0.524	1.43	3.98	11.2	29.8	72.8	153	273
Homo sapiens	CHR_04 (Exon)	0.203	0.482	1.32	3.67	10.2	26.0	58.6	110	—
Homo sapiens	CHR_04 (Intron)	0.213	0.501	1.36	3.81	10.6	27.4	63.9	126	212
Homo sapiens	CHR_05	0.204	0.483	1.32	3.71	10.4	27.2	64.4	129	219
Homo sapiens	CHR_05 (Gene)	0.199	0.473	1.29	3.60	9.93	25.2	56.7	107	174
Homo sapiens	CHR_05 (Intergenic)	0.207	0.489	1.34	3.75	10.6	28.2	68.5	141	247
Homo sapiens	CHR_05 (Exon)	0.182	0.441	1.21	3.37	9.31	23.7	52.8	98.5	—
Homo sapiens	CHR_05 (Intron)	0.194	0.463	1.27	3.54	9.82	25.2	57.1	109	177
Homo sapiens	CHR_06	0.204	0.482	1.32	3.68	10.3	26.7	62.2	122	205
Homo sapiens	CHR_06 (Gene)	0.203	0.479	1.30	3.63	10.0	25.5	57.7	109	179
Homo sapiens	CHR_06 (Intergenic)	0.204	0.483	1.32	3.70	10.4	27.3	64.8	130	223
Homo sapiens	CHR_06 (Exon)	0.186	0.446	1.22	3.39	9.28	23.2	50.3	90.6	—
Homo sapiens	CHR_06 (Intron)	0.197	0.469	1.28	3.57	9.85	25.1	56.5	107	173
Homo sapiens	CHR_07	0.194	0.459	1.25	3.47	9.49	23.8	52.4	96.4	153
Homo sapiens	CHR_07 (Gene)	0.195	0.460	1.25	3.46	9.43	23.4	50.9	92.6	146
Homo sapiens	CHR_07 (Intergenic)	0.193	0.458	1.25	3.46	9.51	24.0	53.4	99.3	159
Homo sapiens	CHR_07 (Exon)	0.176	0.420	1.14	3.09	8.15	19.3	39.1	66.3	—
Homo sapiens	CHR_07 (Intron)	0.192	0.456	1.24	3.44	9.35	23.2	50.3	91.3	144
Homo sapiens	CHR_08	0.200	0.474	1.30	3.64	10.2	26.8	63.2	126	213
Homo sapiens	CHR_08 (Gene)	0.202	0.477	1.30	3.63	10.0	25.7	58.2	111	182
Homo sapiens	CHR_08 (Intergenic)	0.198	0.471	1.29	3.64	10.3	27.3	65.8	134	233
Homo sapiens	CHR_08 (Exon)	0.176	0.429	1.18	3.29	9.04	22.7	49.7	90.4	—
Homo sapiens	CHR_08 (Intron)	0.198	0.469	1.28	3.58	9.91	25.4	57.6	110	179
Homo sapiens	CHR_09	0.187	0.448	1.23	3.44	9.51	24.3	54.7	103	166
Homo sapiens	CHR_09 (Gene)	0.179	0.431	1.18	3.29	9.04	22.7	50.1	91.9	145
Homo sapiens	CHR_09 (Intergenic)	0.193	0.460	1.26	3.52	9.80	25.2	57.7	110	180
Homo sapiens	CHR_09 (Exon)	0.162	0.395	1.08	3.01	8.22	20.5	44.3	79.0	—
Homo sapiens	CHR_09 (Intron)	0.176	0.425	1.17	3.23	8.84	22.0	47.9	86.7	136
Homo sapiens	CHR_10	0.183	0.440	1.21	3.37	9.32	23.8	53.5	101	163
Homo sapiens	CHR_10 (Gene)	0.185	0.441	1.21	3.35	9.15	22.8	49.8	90.8	143

next

		L_e (kb)								
Category	SN \ k	2	3	4	5	6	7	8	9	10
Homo sapiens	CHR_10 (Intergenic)	0.182	0.439	1.21	3.38	9.42	24.4	56.4	109	180
Homo sapiens	CHR_10 (Exon)	0.171	0.412	1.13	3.09	8.30	20.1	42.1	73.5	—
Homo sapiens	CHR_10 (Intron)	0.182	0.436	1.19	3.30	8.99	22.3	48.4	87.4	137
Homo sapiens	CHR_11	0.184	0.445	1.23	3.45	9.71	25.5	59.8	117	196
Homo sapiens	CHR_11 (Gene)	0.178	0.432	1.19	3.34	9.28	23.8	53.8	102	164
Homo sapiens	CHR_11 (Intergenic)	0.189	0.453	1.25	3.52	9.95	26.5	63.6	129	220
Homo sapiens	CHR_11 (Exon)	0.163	0.399	1.10	3.04	8.26	20.5	43.9	78.3	—
Homo sapiens	CHR_11 (Intron)	0.176	0.428	1.18	3.30	9.16	23.4	52.7	98.9	159
Homo sapiens	CHR_12	0.192	0.459	1.25	3.46	9.44	23.5	51.5	94.2	149
Homo sapiens	CHR_12 (Gene)	0.191	0.455	1.24	3.39	9.12	22.1	46.6	82.3	127
Homo sapiens	CHR_12 (Intergenic)	0.193	0.460	1.26	3.49	9.63	24.4	55.0	103	167
Homo sapiens	CHR_12 (Exon)	0.174	0.419	1.13	3.05	7.94	18.3	35.9	59.4	—
Homo sapiens	CHR_12 (Intron)	0.188	0.450	1.22	3.35	8.96	21.6	45.1	78.9	121
Homo sapiens	CHR_13	0.222	0.517	1.41	3.92	11.0	28.8	68.7	139	241
Homo sapiens	CHR_13 (Gene)	0.217	0.504	1.37	3.81	10.6	27.2	62.5	122	203
Homo sapiens	CHR_13 (Intergenic)	0.225	0.522	1.42	3.97	11.1	29.5	71.4	148	261
Homo sapiens	CHR_13 (Exon)	0.199	0.474	1.29	3.61	9.94	24.9	54.4	98.1	—
Homo sapiens	CHR_13 (Intron)	0.207	0.486	1.32	3.70	10.3	26.4	60.8	118	195
Homo sapiens	CHR_14	0.191	0.457	1.25	3.49	9.63	24.5	54.9	103	166
Homo sapiens	CHR_14 (Gene)	0.187	0.446	1.21	3.35	9.04	22.1	46.9	83.3	129
Homo sapiens	CHR_14 (Intergenic)	0.193	0.464	1.27	3.57	9.98	26.0	60.6	118	198
Homo sapiens	CHR_14 (Exon)	0.170	0.414	1.13	3.10	8.29	19.8	40.5	69.1	—
Homo sapiens	CHR_14 (Intron)	0.181	0.436	1.19	3.31	8.99	22.2	47.8	85.7	133
Homo sapiens	CHR_15	0.177	0.426	1.16	3.23	8.83	22.0	47.9	86.9	136
Homo sapiens	CHR_15 (Gene)	0.176	0.421	1.15	3.16	8.53	20.8	44.0	77.7	119
Homo sapiens	CHR_15 (Intergenic)	0.178	0.429	1.18	3.29	9.08	23.1	51.5	95.9	153
Homo sapiens	CHR_15 (Exon)	0.163	0.393	1.07	2.93	7.83	18.8	38.9	67.3	—
Homo sapiens	CHR_15 (Intron)	0.174	0.417	1.14	3.12	8.38	20.3	42.3	73.8	112
Homo sapiens	CHR_16	0.162	0.394	1.07	2.91	7.69	18.1	36.6	61.9	92.3
Homo sapiens	CHR_16 (Gene)	0.156	0.379	1.03	2.77	7.24	16.8	33.4	55.5	81.8
Homo sapiens	CHR_16 (Intergenic)	0.167	0.405	1.11	3.02	8.02	19.1	39.3	67.4	102
Homo sapiens	CHR_16 (Exon)	0.144	0.348	0.926	2.43	6.10	13.4	25.2	39.9	—
Homo sapiens	CHR_16 (Intron)	0.157	0.382	1.03	2.77	7.15	16.3	31.8	52.0	76.0
Homo sapiens	CHR_17	0.155	0.376	1.01	2.71	6.96	15.7	30.1	48.5	70.0

next

		L_e (kb)								
Category	SN \ k	2	3	4	5	6	7	8	9	10
Homo sapiens	CHR_17 (Gene)	0.153	0.369	0.989	2.63	6.67	14.8	28.0	44.7	64.1
Homo sapiens	CHR_17 (Intergenic)	0.158	0.383	1.04	2.80	7.24	16.6	32.2	52.6	76.5
Homo sapiens	CHR_17 (Exon)	0.141	0.345	0.924	2.44	6.14	13.5	25.3	39.9	—
Homo sapiens	CHR_17 (Intron)	0.153	0.370	0.985	2.59	6.45	14.0	25.7	40.3	57.1
Homo sapiens	CHR_18	0.205	0.483	1.32	3.72	10.4	27.5	65.4	132	226
Homo sapiens	CHR_18 (Gene)	0.201	0.473	1.29	3.60	9.98	25.5	57.9	110	180
Homo sapiens	CHR_18 (Intergenic)	0.207	0.489	1.34	3.77	10.6	28.4	69.4	144	255
Homo sapiens	CHR_18 (Exon)	0.193	0.457	1.24	3.44	9.39	23.2	49.8	—	—
Homo sapiens	CHR_18 (Intron)	0.194	0.461	1.26	3.53	9.80	25.1	57.2	109	178
Homo sapiens	CHR_19	0.144	0.341	0.891	2.28	5.49	11.4	20.2	30.7	42.7
Homo sapiens	CHR_19 (Gene)	0.135	0.322	0.842	2.16	5.23	11.0	19.7	30.2	42.2
Homo sapiens	CHR_19 (Intergenic)	0.152	0.356	0.931	2.38	5.69	11.7	20.5	30.9	43.0
Homo sapiens	CHR_19 (Exon)	0.139	0.331	0.856	2.16	5.09	10.4	18.0	26.8	—
Homo sapiens	CHR_19 (Intron)	0.139	0.329	0.849	2.12	4.95	9.95	17.0	25.3	34.7
Homo sapiens	CHR_20	0.160	0.393	1.08	3.02	8.34	21.2	46.9	86.5	137
Homo sapiens	CHR_20 (Gene)	0.160	0.390	1.07	2.98	8.15	20.3	44.0	79.2	123
Homo sapiens	CHR_20 (Intergenic)	0.160	0.394	1.09	3.05	8.47	21.7	49.1	92.3	148
Homo sapiens	CHR_20 (Exon)	0.150	0.367	0.998	2.72	7.20	17.1	34.9	58.8	—
Homo sapiens	CHR_20 (Intron)	0.160	0.391	1.08	2.98	8.09	19.9	42.6	75.5	116
Homo sapiens	CHR_21	0.198	0.469	1.29	3.61	10.1	26.0	60.2	117	195
Homo sapiens	CHR_21 (Gene)	0.184	0.436	1.19	3.33	9.18	23.1	51.0	94.2	149
Homo sapiens	CHR_21 (Intergenic)	0.207	0.488	1.34	3.75	10.5	27.6	65.8	133	228
Homo sapiens	CHR_21 (Exon)	0.164	0.394	1.08	3.04	8.41	21.5	47.5	—	—
Homo sapiens	CHR_21 (Intron)	0.178	0.426	1.17	3.27	9.05	23.0	51.4	95.8	153
Homo sapiens	CHR_22	0.141	0.343	0.927	2.51	6.62	15.6	31.5	52.9	78.0
Homo sapiens	CHR_22 (Gene)	0.140	0.341	0.921	2.50	6.61	15.7	32.1	54.2	80.3
Homo sapiens	CHR_22 (Intergenic)	0.141	0.344	0.932	2.52	6.59	15.4	30.8	51.2	74.9
Homo sapiens	CHR_22 (Exon)	0.137	0.335	0.894	2.39	6.11	13.9	26.6	42.5	—
Homo sapiens	CHR_22 (Intron)	0.141	0.343	0.920	2.46	6.32	14.4	27.8	45.0	64.9
Homo sapiens	CHR_X	0.207	0.488	1.33	3.72	10.4	27.2	64.1	127	215
Homo sapiens	CHR_X (Gene)	0.206	0.490	1.34	3.73	10.3	26.2	58.9	111	180
Homo sapiens	CHR_X (Intergenic)	0.207	0.486	1.33	3.70	10.4	27.4	65.6	133	228
Homo sapiens	CHR_X (Exon)	0.190	0.455	1.24	3.44	9.37	23.3	50.1	89.1	—
Homo sapiens	CHR_X (Intron)	0.203	0.481	1.31	3.63	9.93	24.9	55.1	102	162

next

		L_e (kb)								
Category	SN \ k	2	3	4	5	6	7	8	9	10
Homo sapiens	CHR_Y	0.203	0.471	1.28	3.55	9.84	25.6	60.2	119	200
Homo sapiens	CHR_Y (Gene)	0.210	0.480	1.30	3.57	9.81	24.8	56.1	106	172
Homo sapiens	CHR_Y (Intergenic)	0.201	0.467	1.27	3.50	9.64	24.7	57.3	112	186
Homo sapiens	CHR_Y (Exon)	0.201	0.472	1.27	3.38	8.72	19.7	—	—	
Homo sapiens	CHR_Y (Intron)	0.213	0.481	1.29	3.50	9.36	22.7	48.0	85.0	—
Macaca mulatta	CHR_01	0.185	0.441	1.21	3.34	9.05	22.2	47.3	83.6	128
Macaca mulatta	CHR_01 (Gene)	0.183	0.436	1.18	3.25	8.66	20.7	42.4	72.4	108
Macaca mulatta	CHR_01 (Intergenic)	0.187	0.445	1.22	3.39	9.32	23.4	51.3	93.1	146
Macaca mulatta	CHR_01 (Exon)	0.166	0.401	1.09	2.98	7.88	18.5	37.1	61.8	90.9
Macaca mulatta	CHR_01 (Intron)	0.184	0.439	1.19	3.27	8.72	20.9	42.9	73.4	110
Macaca mulatta	CHR_02	0.206	0.483	1.32	3.67	10.1	25.8	58.1	109	177
Macaca mulatta	CHR_02 (Gene)	0.196	0.463	1.26	3.51	9.60	24.0	52.4	95.3	150
Macaca mulatta	CHR_02 (Intergenic)	0.212	0.496	1.35	3.76	10.4	26.9	61.7	119	195
Macaca mulatta	CHR_02 (Exon)	0.183	0.439	1.20	3.31	8.98	21.9	46.2	81.2	—
Macaca mulatta	CHR_02 (Intron)	0.196	0.464	1.27	3.52	9.63	24.1	52.7	96.1	152
Macaca mulatta	CHR_03	0.199	0.465	1.27	3.51	9.61	24.0	52.4	95.5	150
Macaca mulatta	CHR_03 (Gene)	0.195	0.458	1.25	3.44	9.35	23.0	49.1	87.5	135
Macaca mulatta	CHR_03 (Intergenic)	0.202	0.470	1.28	3.55	9.75	24.6	54.4	100	160
Macaca mulatta	CHR_03 (Exon)	0.180	0.429	1.17	3.23	8.77	21.6	45.8	80.6	—
Macaca mulatta	CHR_03 (Intron)	0.196	0.459	1.25	3.46	9.38	23.1	49.3	87.8	136
Macaca mulatta	CHR_04	0.208	0.485	1.32	3.67	10.1	25.6	57.4	107	173
Macaca mulatta	CHR_04 (Gene)	0.203	0.474	1.29	3.56	9.70	24.0	51.9	93.7	147
Macaca mulatta	CHR_04 (Intergenic)	0.210	0.490	1.33	3.72	10.3	26.5	60.4	115	189
Macaca mulatta	CHR_04 (Exon)	0.186	0.444	1.21	3.34	9.05	22.1	46.8	82.0	—
Macaca mulatta	CHR_04 (Intron)	0.204	0.476	1.29	3.58	9.74	24.1	52.3	94.4	148
Macaca mulatta	CHR_05	0.229	0.526	1.42	3.94	10.9	27.7	63.2	121	200
Macaca mulatta	CHR_05 (Gene)	0.221	0.511	1.38	3.81	10.4	25.9	57.3	106	171
Macaca mulatta	CHR_05 (Intergenic)	0.233	0.533	1.44	3.99	11.0	28.4	65.7	128	214
Macaca mulatta	CHR_05 (Exon)	0.216	0.503	1.37	3.79	10.4	25.9	57.1	105	—
Macaca mulatta	CHR_05 (Intron)	0.222	0.511	1.38	3.81	10.4	25.9	57.3	106	171
Macaca mulatta	CHR_06	0.207	0.485	1.32	3.69	10.2	26.2	59.7	113	185
Macaca mulatta	CHR_06 (Gene)	0.200	0.471	1.28	3.56	9.73	24.2	52.7	95.6	150
Macaca mulatta	CHR_06 (Intergenic)	0.211	0.492	1.34	3.75	10.5	27.2	63.2	123	205
Macaca mulatta	CHR_06 (Exon)	0.192	0.455	1.24	3.42	9.26	22.7	48.3	85.3	—

next

			L_e (kb)								
Category	SN	\ k	2	3	4	5	6	7	8	9	10
Macaca mulatta	CHR_06 (Intron)		0.201	0.473	1.29	3.58	9.77	24.4	53.1	96.6	152
Macaca mulatta	CHR_07		0.188	0.445	1.21	3.36	9.14	22.5	48.2	85.6	132
Macaca mulatta	CHR_07 (Gene)		0.184	0.436	1.18	3.23	8.57	20.3	41.5	70.5	106
Macaca mulatta	CHR_07 (Intergenic)		0.190	0.451	1.24	3.45	9.51	24.0	53.3	98.0	155
Macaca mulatta	CHR_07 (Exon)		0.170	0.409	1.11	3.03	8.03	18.9	38.0	63.4	—
Macaca mulatta	CHR_07 (Intron)		0.185	0.438	1.19	3.24	8.61	20.5	41.8	71.1	107
Macaca mulatta	CHR_08		0.204	0.477	1.30	3.64	10.1	26.0	59.2	113	184
Macaca mulatta	CHR_08 (Gene)		0.202	0.472	1.28	3.56	9.75	24.4	53.5	97.9	155
Macaca mulatta	CHR_08 (Intergenic)		0.205	0.480	1.31	3.67	10.3	26.7	62.0	121	200
Macaca mulatta	CHR_08 (Exon)		0.183	0.439	1.20	3.34	9.13	22.7	48.9	87.0	—
Macaca mulatta	CHR_08 (Intron)		0.203	0.473	1.29	3.57	9.78	24.5	53.7	98.5	156
Macaca mulatta	CHR_09		0.186	0.444	1.21	3.38	9.25	23.1	50.6	91.9	144
Macaca mulatta	CHR_09 (Gene)		0.185	0.439	1.20	3.30	8.91	21.7	45.7	80.1	122
Macaca mulatta	CHR_09 (Intergenic)		0.187	0.446	1.22	3.42	9.46	24.1	53.9	101	161
Macaca mulatta	CHR_09 (Exon)		0.174	0.416	1.13	3.09	8.20	19.4	39.3	66.3	—
Macaca mulatta	CHR_09 (Intron)		0.185	0.441	1.20	3.31	8.95	21.8	46.1	81.0	124
Macaca mulatta	CHR_10		0.156	0.377	1.03	2.83	7.59	18.3	37.7	64.5	96.2
Macaca mulatta	CHR_10 (Gene)		0.155	0.373	1.01	2.74	7.16	16.6	32.6	53.5	77.6
Macaca mulatta	CHR_10 (Intergenic)		0.156	0.380	1.04	2.89	7.87	19.5	41.8	73.9	113
Macaca mulatta	CHR_10 (Exon)		0.142	0.348	0.942	2.56	6.73	15.9	31.9	53.2	—
Macaca mulatta	CHR_10 (Intron)		0.156	0.376	1.02	2.76	7.20	16.7	32.8	53.8	78.1
Macaca mulatta	CHR_11		0.195	0.457	1.24	3.42	9.25	22.6	47.9	84.4	129
Macaca mulatta	CHR_11 (Gene)		0.192	0.453	1.22	3.34	8.85	20.9	42.5	72.0	108
Macaca mulatta	CHR_11 (Intergenic)		0.196	0.459	1.25	3.46	9.47	23.7	51.5	93.2	146
Macaca mulatta	CHR_11 (Exon)		0.173	0.417	1.13	3.07	8.05	18.8	37.4	62.2	—
Macaca mulatta	CHR_11 (Intron)		0.193	0.455	1.23	3.36	8.89	21.0	42.8	72.7	109
Macaca mulatta	CHR_12		0.210	0.493	1.34	3.74	10.3	26.4	59.6	113	183
Macaca mulatta	CHR_12 (Gene)		0.208	0.487	1.32	3.66	9.99	24.8	53.9	97.5	153
Macaca mulatta	CHR_12 (Intergenic)		0.212	0.496	1.35	3.78	10.5	27.4	63.5	124	206
Macaca mulatta	CHR_12 (Exon)		0.194	0.462	1.25	3.46	9.33	22.8	47.9	83.1	—
Macaca mulatta	CHR_12 (Intron)		0.209	0.489	1.33	3.68	10.0	24.9	54.2	98.3	155
Macaca mulatta	CHR_13		0.193	0.454	1.24	3.47	9.63	24.6	55.2	103	165
Macaca mulatta	CHR_13 (Gene)		0.187	0.442	1.20	3.32	8.95	21.8	45.8	80.1	122
Macaca mulatta	CHR_13 (Intergenic)		0.196	0.460	1.26	3.54	9.94	26.0	60.4	118	194

next

		L_e (kb)								
Category	SN \ k	2	3	4	5	6	7	8	9	10
Macaca mulatta	CHR_13 (Exon)	0.174	0.417	1.13	3.13	8.37	20.0	40.9	69.1	—
Macaca mulatta	CHR_13 (Intron)	0.188	0.444	1.21	3.33	8.99	21.9	46.2	81.0	124
Macaca mulatta	CHR_14	0.188	0.450	1.24	3.46	9.61	24.6	55.1	103	164
Macaca mulatta	CHR_14 (Gene)	0.182	0.437	1.20	3.32	9.05	22.4	48.0	85.4	132
Macaca mulatta	CHR_14 (Intergenic)	0.193	0.459	1.26	3.55	9.97	26.0	60.2	116	190
Macaca mulatta	CHR_14 (Exon)	0.165	0.402	1.10	3.05	8.27	20.2	42.4	73.4	—
Macaca mulatta	CHR_14 (Intron)	0.183	0.440	1.20	3.34	9.11	22.6	48.5	86.4	134
Macaca mulatta	CHR_15	0.190	0.451	1.23	3.43	9.38	23.4	50.8	91.6	143
Macaca mulatta	CHR_15 (Gene)	0.179	0.427	1.17	3.22	8.71	21.2	44.6	77.7	118
Macaca mulatta	CHR_15 (Intergenic)	0.197	0.465	1.27	3.54	9.75	24.6	54.6	101	160
Macaca mulatta	CHR_15 (Exon)	0.166	0.402	1.10	3.03	8.14	19.6	40.4	68.8	—
Macaca mulatta	CHR_15 (Intron)	0.180	0.429	1.17	3.23	8.74	21.3	44.9	78.4	119
Macaca mulatta	CHR_16	0.159	0.381	1.02	2.72	6.88	15.2	28.4	44.8	63.5
Macaca mulatta	CHR_16 (Gene)	0.158	0.376	0.997	2.62	6.48	13.9	25.2	38.9	54.4
Macaca mulatta	CHR_16 (Intergenic)	0.160	0.385	1.04	2.80	7.24	16.5	31.7	51.1	73.5
Macaca mulatta	CHR_16 (Exon)	0.145	0.353	0.944	2.51	6.33	14.0	26.1	41.1	—
Macaca mulatta	CHR_16 (Intron)	0.159	0.378	1.00	2.63	6.50	13.9	25.2	38.9	54.4
Macaca mulatta	CHR_17	0.229	0.523	1.42	3.94	10.9	27.9	63.6	122	202
Macaca mulatta	CHR_17 (Gene)	0.214	0.493	1.33	3.69	10.0	24.8	53.7	97.2	152
Macaca mulatta	CHR_17 (Intergenic)	0.235	0.536	1.45	4.03	11.2	29.0	67.6	133	224
Macaca mulatta	CHR_17 (Exon)	0.204	0.477	1.30	3.62	9.91	24.8	53.5	95.3	—
Macaca mulatta	CHR_17 (Intron)	0.214	0.494	1.34	3.69	10.0	24.8	53.7	97.0	152
Macaca mulatta	CHR_18	0.210	0.490	1.34	3.73	10.4	26.7	61.0	117	191
Macaca mulatta	CHR_18 (Gene)	0.204	0.474	1.29	3.58	9.81	24.5	53.6	97.8	154
Macaca mulatta	CHR_18 (Intergenic)	0.213	0.497	1.36	3.80	10.6	27.7	64.7	127	212
Macaca mulatta	CHR_18 (Exon)	0.198	0.465	1.26	3.47	9.34	22.6	46.7	—	—
Macaca mulatta	CHR_18 (Intron)	0.204	0.475	1.29	3.59	9.84	24.6	54.0	98.6	156
Macaca mulatta	CHR_19	0.149	0.347	0.902	2.30	5.48	11.2	19.4	28.9	39.6
Macaca mulatta	CHR_19 (Gene)	0.144	0.338	0.872	2.20	5.15	10.4	17.8	26.3	35.9
Macaca mulatta	CHR_19 (Intergenic)	0.153	0.352	0.920	2.36	5.70	11.8	20.6	30.8	42.4
Macaca mulatta	CHR_19 (Exon)	0.143	0.342	0.895	2.33	5.74	12.4	22.7	35.4	—
Macaca mulatta	CHR_19 (Intron)	0.144	0.338	0.870	2.18	5.09	10.2	17.3	25.5	34.7
Macaca mulatta	CHR_20	0.166	0.398	1.08	2.93	7.67	17.8	35.4	58.6	86.2
Macaca mulatta	CHR_20 (Gene)	0.157	0.376	1.01	2.69	6.84	15.3	28.9	46.1	66.0

next

		L_e (kb)								
Category	SN \ k	2	3	4	5	6	7	8	9	10
Macaca mulatta	CHR_20 (Intergenic)	0.172	0.412	1.12	3.08	8.24	19.7	40.5	69.4	104
Macaca mulatta	CHR_20 (Exon)	0.145	0.351	0.937	2.49	6.37	14.4	27.5	44.2	—
Macaca mulatta	CHR_20 (Intron)	0.158	0.378	1.01	2.71	6.89	15.4	29.1	46.5	66.6
Macaca mulatta	CHR_X	0.208	0.487	1.32	3.68	10.2	26.0	58.6	110	176
Macaca mulatta	CHR_X (Gene)	0.205	0.483	1.31	3.63	9.87	24.3	52.2	92.8	143
Macaca mulatta	CHR_X (Intergenic)	0.208	0.488	1.33	3.69	10.3	26.5	61.0	117	191
Macaca mulatta	CHR_X (Exon)	0.187	0.451	1.24	3.46	9.51	23.9	52.2	94.1	
Macaca mulatta	CHR_X (Intron)	0.206	0.485	1.32	3.64	9.89	24.4	52.2	92.6	143
Mus musculus	CHR_01	0.209	0.468	1.28	3.51	9.71	24.6	55.2	101	152
Mus musculus	CHR_01 (Gene)	0.206	0.459	1.26	3.48	9.68	24.9	56.9	107	165
Mus musculus	CHR_01 (Intergenic)	0.211	0.471	1.28	3.52	9.67	24.3	53.8	96.4	143
Mus musculus	CHR_01 (Exon)	0.183	0.438	1.18	3.40	10.3	31.5	95.6	276	—
Mus musculus	CHR_01 (Intron)	0.207	0.460	1.26	3.47	9.57	24.3	54.8	101	154
Mus musculus	CHR_02	0.198	0.446	1.23	3.39	9.42	24.1	54.8	101	155
Mus musculus	CHR_02 (Gene)	0.191	0.431	1.19	3.31	9.25	24.0	55.7	106	168
Mus musculus	CHR_02 (Intergenic)	0.202	0.454	1.24	3.42	9.45	23.9	53.4	96.6	145
Mus musculus	CHR_02 (Exon)	0.182	0.434	1.17	3.36	10.2	31.1	95.1	278	710
Mus musculus	CHR_02 (Intron)	0.191	0.428	1.18	3.26	9.02	23.1	52.4	97.8	152
Mus musculus	CHR_03	0.217	0.485	1.32	3.65	10.1	25.7	58.5	108	165
Mus musculus	CHR_03 (Gene)	0.215	0.478	1.31	3.62	10.1	25.9	59.6	113	178
Mus musculus	CHR_03 (Intergenic)	0.217	0.486	1.32	3.63	10.0	25.5	57.4	105	158
Mus musculus	CHR_03 (Exon)	0.197	0.469	1.26	3.67	11.1	33.8	102	286	—
Mus musculus	CHR_03 (Intron)	0.216	0.480	1.31	3.61	9.96	25.3	57.3	106	165
Mus musculus	CHR_04	0.196	0.443	1.22	3.36	9.34	23.9	54.1	99.5	152
Mus musculus	CHR_04 (Gene)	0.185	0.418	1.16	3.21	8.97	23.3	54.1	103	164
Mus musculus	CHR_04 (Intergenic)	0.201	0.453	1.24	3.41	9.43	23.8	53.0	95.3	142
Mus musculus	CHR_04 (Exon)	0.177	0.424	1.14	3.28	9.91	30.1	91.5	264	—
Mus musculus	CHR_04 (Intron)	0.182	0.409	1.13	3.11	8.58	21.8	49.2	90.8	140
Mus musculus	CHR_05	0.200	0.446	1.22	3.36	9.26	23.4	52.3	95.2	145
Mus musculus	CHR_05 (Gene)	0.191	0.425	1.17	3.22	8.92	22.8	51.9	97.3	152
Mus musculus	CHR_05 (Intergenic)	0.205	0.458	1.25	3.42	9.39	23.4	51.5	91.8	137
Mus musculus	CHR_05 (Exon)	0.187	0.442	1.17	3.38	10.2	30.8	93.2	267	—
Mus musculus	CHR_05 (Intron)	0.193	0.429	1.18	3.24	8.91	22.6	50.7	93.6	145
Mus musculus	CHR_06	0.206	0.462	1.26	3.48	9.65	24.6	55.2	101	152

next

Category	SN \ k	L_e (kb) 2	3	4	5	6	7	8	9	10
Mus musculus	CHR_06 (Gene)	0.202	0.453	1.24	3.45	9.62	24.9	57.4	108	168
Mus musculus	CHR_06 (Intergenic)	0.208	0.465	1.27	3.49	9.60	24.1	53.4	95.3	141
Mus musculus	CHR_06 (Exon)	0.183	0.435	1.17	3.38	10.2	31.1	94.3	269	—
Mus musculus	CHR_06 (Intron)	0.209	0.467	1.28	3.52	9.74	24.8	56.1	103	158
Mus musculus	CHR_07	0.185	0.423	1.17	3.25	9.15	23.9	55.4	105	162
Mus musculus	CHR_07 (Gene)	0.175	0.402	1.11	3.12	8.82	23.4	55.6	109	174
Mus musculus	CHR_07 (Intergenic)	0.191	0.435	1.20	3.32	9.27	23.9	54.4	100	152
Mus musculus	CHR_07 (Exon)	0.168	0.403	1.08	3.12	9.44	28.7	87.3	255	654
Mus musculus	CHR_07 (Intron)	0.174	0.398	1.10	3.06	8.57	22.2	51.5	97.4	152
Mus musculus	CHR_08	0.202	0.450	1.23	3.39	9.33	23.5	52.3	94.6	142
Mus musculus	CHR_08 (Gene)	0.191	0.426	1.17	3.23	8.96	22.9	52.0	96.5	149
Mus musculus	CHR_08 (Intergenic)	0.208	0.462	1.26	3.45	9.46	23.7	51.9	92.2	137
Mus musculus	CHR_08 (Exon)	0.187	0.443	1.18	3.38	10.2	30.8	92.9	264	—
Mus musculus	CHR_08 (Intron)	0.193	0.428	1.17	3.22	8.82	22.1	49.0	88.7	134
Mus musculus	CHR_09	0.193	0.434	1.19	3.28	9.07	23.1	51.8	94.7	144
Mus musculus	CHR_09 (Gene)	0.188	0.421	1.16	3.20	8.86	22.6	51.4	96.0	149
Mus musculus	CHR_09 (Intergenic)	0.195	0.441	1.21	3.32	9.15	23.1	51.3	92.1	137
Mus musculus	CHR_09 (Exon)	0.184	0.437	1.17	3.38	10.2	31.2	94.9	275	—
Mus musculus	CHR_09 (Intron)	0.186	0.413	1.14	3.10	8.47	21.1	46.3	83.2	125
Mus musculus	CHR_10	0.214	0.475	1.30	3.56	9.77	24.5	54.5	98.8	150
Mus musculus	CHR_10 (Gene)	0.209	0.461	1.26	3.47	9.56	24.1	54.1	99.7	154
Mus musculus	CHR_10 (Intergenic)	0.215	0.479	1.31	3.57	9.78	24.4	54.0	97.0	146
Mus musculus	CHR_10 (Exon)	0.199	0.469	1.26	3.63	11.0	33.2	99.5	279	—
Mus musculus	CHR_10 (Intron)	0.209	0.458	1.25	3.42	9.32	23.2	51.0	92.0	140
Mus musculus	CHR_11	0.180	0.408	1.12	3.09	8.52	21.5	47.6	86.3	131
Mus musculus	CHR_11 (Gene)	0.172	0.392	1.08	2.99	8.29	21.2	48.4	90.9	142
Mus musculus	CHR_11 (Intergenic)	0.186	0.419	1.15	3.15	8.60	21.3	46.3	81.4	120
Mus musculus	CHR_11 (Exon)	0.167	0.405	1.08	3.08	9.29	28.1	85.3	251	653
Mus musculus	CHR_11 (Intron)	0.178	0.402	1.11	3.06	8.45	21.5	48.2	88.7	136
Mus musculus	CHR_12	0.205	0.460	1.26	3.48	9.69	24.8	56.5	105	160
Mus musculus	CHR_12 (Gene)	0.199	0.447	1.23	3.42	9.57	24.8	57.6	110	174
Mus musculus	CHR_12 (Intergenic)	0.206	0.464	1.27	3.50	9.68	24.6	55.3	101	152
Mus musculus	CHR_12 (Exon)	0.197	0.463	1.23	3.54	10.7	32.1	94.5	253	—
Mus musculus	CHR_12 (Intron)	0.198	0.443	1.22	3.36	9.33	23.9	54.4	102	159

next

Category	SN	\ k	L_e (kb) 2	3	4	5	6	7	8	9	10
Mus musculus	CHR_13		0.207	0.462	1.27	3.49	9.70	24.8	56.5	105	161
Mus musculus	CHR_13 (Gene)		0.208	0.463	1.27	3.52	9.79	25.3	58.3	110	173
Mus musculus	CHR_13 (Intergenic)		0.205	0.461	1.26	3.47	9.61	24.5	55.2	101	153
Mus musculus	CHR_13 (Exon)		0.201	0.469	1.26	3.64	11.0	33.1	97.7	262	—
Mus musculus	CHR_13 (Intron)		0.207	0.459	1.26	3.46	9.55	24.3	54.7	101	155
Mus musculus	CHR_14		0.210	0.471	1.29	3.56	9.89	25.3	57.5	106	163
Mus musculus	CHR_14 (Gene)		0.199	0.449	1.24	3.43	9.59	24.9	57.7	109	171
Mus musculus	CHR_14 (Intergenic)		0.216	0.482	1.31	3.61	9.97	25.3	56.8	103	156
Mus musculus	CHR_14 (Exon)		0.182	0.434	1.16	3.36	10.1	30.7	91.3	250	—
Mus musculus	CHR_14 (Intron)		0.200	0.447	1.23	3.40	9.43	24.2	55.2	103	159
Mus musculus	CHR_15		0.204	0.460	1.26	3.49	9.71	24.9	56.7	105	161
Mus musculus	CHR_15 (Gene)		0.195	0.439	1.21	3.35	9.37	24.3	56.3	107	169
Mus musculus	CHR_15 (Intergenic)		0.209	0.470	1.29	3.54	9.81	24.9	56.0	102	153
Mus musculus	CHR_15 (Exon)		0.176	0.425	1.13	3.26	9.81	29.6	88.8	251	—
Mus musculus	CHR_15 (Intron)		0.196	0.437	1.20	3.32	9.19	23.5	53.4	99.6	155
Mus musculus	CHR_16		0.217	0.481	1.31	3.60	9.93	25.1	56.3	103	157
Mus musculus	CHR_16 (Gene)		0.203	0.452	1.24	3.43	9.53	24.5	56.4	107	168
Mus musculus	CHR_16 (Intergenic)		0.223	0.494	1.34	3.68	10.1	25.4	56.6	103	155
Mus musculus	CHR_16 (Exon)		0.190	0.447	1.20	3.46	10.5	31.9	96.3	272	—
Mus musculus	CHR_16 (Intron)		0.205	0.452	1.24	3.40	9.38	23.8	53.9	100	156
Mus musculus	CHR_17		0.197	0.442	1.21	3.35	9.30	23.8	54.0	100	154
Mus musculus	CHR_17 (Gene)		0.185	0.414	1.14	3.16	8.84	22.9	53.2	102	161
Mus musculus	CHR_17 (Intergenic)		0.201	0.451	1.24	3.40	9.39	23.8	53.3	97.2	147
Mus musculus	CHR_17 (Exon)		0.173	0.412	1.10	3.15	9.48	28.6	85.7	240	—
Mus musculus	CHR_17 (Intron)		0.186	0.411	1.13	3.11	8.56	21.7	48.9	90.7	141
Mus musculus	CHR_18		0.208	0.465	1.27	3.50	9.66	24.5	55.1	101	153
Mus musculus	CHR_18 (Gene)		0.206	0.456	1.25	3.45	9.56	24.4	55.6	104	161
Mus musculus	CHR_18 (Intergenic)		0.209	0.468	1.28	3.50	9.65	24.3	54.2	97.8	146
Mus musculus	CHR_18 (Exon)		0.208	0.485	1.29	3.74	11.3	33.7	97.9	256	—
Mus musculus	CHR_18 (Intron)		0.205	0.453	1.24	3.40	9.35	23.6	52.7	96.7	148
Mus musculus	CHR_19		0.195	0.440	1.21	3.34	9.21	23.3	52.1	95.3	145
Mus musculus	CHR_19 (Gene)		0.189	0.426	1.18	3.25	9.04	23.1	52.7	98.6	154
Mus musculus	CHR_19 (Intergenic)		0.198	0.447	1.22	3.36	9.21	23.0	50.7	90.8	136
Mus musculus	CHR_19 (Exon)		0.180	0.437	1.17	3.36	10.1	30.5	90.6	249	—

next

		L_e (kb)								
Category	SN \ k	2	3	4	5	6	7	8	9	10
Mus musculus	CHR_19 (Intron)	0.189	0.423	1.17	3.20	8.79	22.1	49.1	89.6	137
Mus musculus	CHR_X	0.217	0.495	1.35	3.73	10.4	26.7	61.6	116	180
Mus musculus	CHR_X (Gene)	0.219	0.501	1.37	3.81	10.6	27.7	65.5	128	204
Mus musculus	CHR_X (Intergenic)	0.214	0.490	1.33	3.68	10.2	26.1	59.8	111	170
Mus musculus	CHR_X (Exon)	0.181	0.451	1.23	3.56	10.8	32.8	97.3	265	—
Mus musculus	CHR_X (Intron)	0.223	0.505	1.38	3.79	10.5	26.9	62.3	119	187
Mus musculus	CHR_Y	0.208	0.457	1.23	3.35	9.01	22.0	47.8	84.9	128
Mus musculus	CHR_Y (Gene)	0.192	0.428	1.16	3.18	8.69	21.7	48.5	89.7	—
Mus musculus	CHR_Y (Intergenic)	0.210	0.461	1.24	3.37	9.03	21.9	47.0	82.5	122
Mus musculus	CHR_Y (Exon)	0.160	0.371	0.975	2.69	6.88	14.3	22.9	—	—
Mus musculus	CHR_Y (Intron)	0.197	0.438	1.18	3.21	8.60	21.0	45.9	83.2	—
Pan troglodytes	CHR_01	0.185	0.446	1.23	3.43	9.48	24.2	54.6	103	167
Pan troglodytes	CHR_01 (Gene)	0.180	0.436	1.19	3.31	9.02	22.4	48.5	87.4	137
Pan troglodytes	CHR_01 (Intergenic)	0.188	0.454	1.25	3.51	9.80	25.5	59.4	116	194
Pan troglodytes	CHR_01 (Exon)	0.160	0.399	1.05	3.03	9.19	28.0	85.7	256	—
Pan troglodytes	CHR_01 (Intron)	0.180	0.433	1.18	3.27	8.80	21.5	45.6	80.9	125
Pan troglodytes	CHR_02A	0.189	0.453	1.24	3.49	9.76	25.4	59.2	116	194
Pan troglodytes	CHR_02A (Gene)	0.180	0.432	1.18	3.27	8.91	22.1	47.9	86.5	136
Pan troglodytes	CHR_02A (Intergenic)	0.194	0.464	1.28	3.60	10.2	27.2	66.0	136	237
Pan troglodytes	CHR_02A (Exon)	0.170	0.423	1.12	3.25	9.86	29.9	89.2	243	—
Pan troglodytes	CHR_02A (Intron)	0.179	0.427	1.16	3.20	8.64	21.1	44.8	79.5	123
Pan troglodytes	CHR_02B	0.204	0.484	1.33	3.73	10.5	27.7	66.3	135	233
Pan troglodytes	CHR_02B (Gene)	0.200	0.477	1.30	3.64	10.1	25.8	59.0	113	187
Pan troglodytes	CHR_02B (Intergenic)	0.205	0.488	1.34	3.77	10.7	28.6	70.3	147	263
Pan troglodytes	CHR_02B (Exon)	0.177	0.440	1.17	3.40	10.3	31.5	94.8	261	—
Pan troglodytes	CHR_02B (Intron)	0.202	0.479	1.30	3.62	9.96	25.2	56.9	108	177
Pan troglodytes	CHR_03	0.201	0.478	1.31	3.68	10.3	27.0	64.0	128	218
Pan troglodytes	CHR_03 (Gene)	0.192	0.460	1.26	3.52	9.75	24.9	56.6	108	176
Pan troglodytes	CHR_03 (Intergenic)	0.206	0.489	1.34	3.76	10.6	28.2	68.6	142	250
Pan troglodytes	CHR_03 (Exon)	0.169	0.419	1.11	3.21	9.76	29.8	91.2	264	—
Pan troglodytes	CHR_03 (Intron)	0.193	0.459	1.25	3.49	9.59	24.2	54.2	102	164
Pan troglodytes	CHR_04	0.224	0.522	1.42	3.97	11.1	29.5	71.7	149	265
Pan troglodytes	CHR_04 (Gene)	0.215	0.505	1.37	3.83	10.6	27.5	63.9	126	214
Pan troglodytes	CHR_04 (Intergenic)	0.227	0.529	1.44	4.03	11.3	30.3	74.8	159	289

next

Category	SN	\ k	L_e (kb) 2	3	4	5	6	7	8	9	10
Pan troglodytes	CHR_04 (Exon)		0.189	0.465	1.24	3.61	11.0	33.3	98.7	263	—
Pan troglodytes	CHR_04 (Intron)		0.216	0.503	1.37	3.79	10.4	26.8	61.5	120	201
Pan troglodytes	CHR_05		0.204	0.485	1.33	3.73	10.5	27.8	66.7	136	235
Pan troglodytes	CHR_05 (Gene)		0.198	0.472	1.29	3.61	10.0	25.9	59.3	114	189
Pan troglodytes	CHR_05 (Intergenic)		0.207	0.491	1.35	3.78	10.7	28.7	70.3	147	261
Pan troglodytes	CHR_05 (Exon)		0.188	0.461	1.22	3.52	10.7	32.4	97.1	269	—
Pan troglodytes	CHR_05 (Intron)		0.197	0.468	1.28	3.56	9.83	25.0	56.5	107	176
Pan troglodytes	CHR_06		0.204	0.484	1.32	3.71	10.4	27.1	64.0	128	218
Pan troglodytes	CHR_06 (Gene)		0.198	0.471	1.28	3.57	9.82	24.8	55.4	104	168
Pan troglodytes	CHR_06 (Intergenic)		0.208	0.490	1.34	3.77	10.6	28.3	68.6	142	249
Pan troglodytes	CHR_06 (Exon)		0.176	0.435	1.16	3.35	10.2	31.1	94.4	269	—
Pan troglodytes	CHR_06 (Intron)		0.198	0.469	1.28	3.53	9.65	24.1	53.1	98.3	158
Pan troglodytes	CHR_07		0.196	0.464	1.27	3.53	9.74	24.8	56.0	106	172
Pan troglodytes	CHR_07 (Gene)		0.193	0.459	1.25	3.48	9.52	23.9	52.6	96.9	154
Pan troglodytes	CHR_07 (Intergenic)		0.197	0.466	1.27	3.56	9.86	25.3	57.9	111	183
Pan troglodytes	CHR_07 (Exon)		0.173	0.424	1.11	3.19	9.62	28.9	86.3	240	—
Pan troglodytes	CHR_07 (Intron)		0.194	0.459	1.25	3.45	9.37	23.2	50.5	91.9	145
Pan troglodytes	CHR_08		0.200	0.476	1.31	3.67	10.3	27.2	64.8	131	224
Pan troglodytes	CHR_08 (Gene)		0.198	0.471	1.29	3.59	9.97	25.6	58.4	112	185
Pan troglodytes	CHR_08 (Intergenic)		0.201	0.479	1.31	3.70	10.5	27.9	67.7	140	245
Pan troglodytes	CHR_08 (Exon)		0.182	0.446	1.17	3.35	10.1	30.2	88.8	235	—
Pan troglodytes	CHR_08 (Intron)		0.198	0.470	1.28	3.56	9.78	24.8	55.6	105	171
Pan troglodytes	CHR_09		0.189	0.452	1.24	3.48	9.71	25.1	57.6	111	182
Pan troglodytes	CHR_09 (Gene)		0.178	0.429	1.18	3.29	9.05	22.8	50.4	92.8	147
Pan troglodytes	CHR_09 (Intergenic)		0.195	0.465	1.28	3.59	10.1	26.3	61.7	122	205
Pan troglodytes	CHR_09 (Exon)		0.165	0.407	1.07	3.06	9.20	27.7	82.6	230	—
Pan troglodytes	CHR_09 (Intron)		0.177	0.427	1.17	3.25	8.86	22.0	47.9	86.8	136
Pan troglodytes	CHR_10		0.183	0.442	1.22	3.40	9.44	24.3	55.4	106	173
Pan troglodytes	CHR_10 (Gene)		0.183	0.440	1.21	3.35	9.19	23.0	50.6	93.1	148
Pan troglodytes	CHR_10 (Intergenic)		0.183	0.442	1.22	3.42	9.58	25.0	58.4	114	191
Pan troglodytes	CHR_10 (Exon)		0.177	0.435	1.15	3.33	10.1	30.6	91.2	250	—
Pan troglodytes	CHR_10 (Intron)		0.182	0.437	1.19	3.31	8.99	22.3	48.2	87.3	137
Pan troglodytes	CHR_11		0.185	0.448	1.24	3.48	9.82	25.9	61.3	122	206
Pan troglodytes	CHR_11 (Gene)		0.178	0.433	1.19	3.34	9.27	23.7	53.5	100	162

next

			L_e (kb)								
Category	SN	\ k	2	3	4	5	6	7	8	9	10
Pan troglodytes	CHR_11 (Intergenic)		0.190	0.457	1.26	3.57	10.1	27.3	66.6	138	241
Pan troglodytes	CHR_11 (Exon)		0.158	0.394	1.03	2.95	8.86	26.6	80.0	231	—
Pan troglodytes	CHR_11 (Intron)		0.176	0.428	1.18	3.28	8.99	22.6	49.9	91.7	145
Pan troglodytes	CHR_12		0.189	0.454	1.24	3.44	9.42	23.6	52.0	95.8	152
Pan troglodytes	CHR_12 (Gene)		0.180	0.434	1.18	3.25	8.71	21.1	44.0	77.0	118
Pan troglodytes	CHR_12 (Intergenic)		0.195	0.466	1.28	3.56	9.86	25.3	57.7	111	182
Pan troglodytes	CHR_12 (Exon)		0.162	0.406	1.07	3.08	9.28	27.9	83.6	234	—
Pan troglodytes	CHR_12 (Intron)		0.180	0.433	1.18	3.21	8.51	20.3	41.8	72.2	110
Pan troglodytes	CHR_13		0.223	0.519	1.41	3.95	11.1	29.4	70.9	147	259
Pan troglodytes	CHR_13 (Gene)		0.210	0.491	1.34	3.71	10.2	26.1	59.1	113	186
Pan troglodytes	CHR_13 (Intergenic)		0.228	0.529	1.44	4.03	11.4	30.4	75.2	160	292
Pan troglodytes	CHR_13 (Exon)		0.196	0.478	1.27	3.66	11.1	33.0	93.2	—	—
Pan troglodytes	CHR_13 (Intron)		0.209	0.487	1.32	3.66	9.99	25.1	55.9	105	170
Pan troglodytes	CHR_14		0.191	0.459	1.26	3.52	9.76	25.0	56.8	108	177
Pan troglodytes	CHR_14 (Gene)		0.184	0.442	1.21	3.34	9.07	22.4	48.1	86.5	135
Pan troglodytes	CHR_14 (Intergenic)		0.196	0.470	1.29	3.63	10.2	26.6	62.6	124	210
Pan troglodytes	CHR_14 (Exon)		0.168	0.416	1.09	3.12	9.35	27.8	81.0	213	—
Pan troglodytes	CHR_14 (Intron)		0.184	0.441	1.20	3.30	8.87	21.5	45.5	80.5	125
Pan troglodytes	CHR_15		0.178	0.429	1.18	3.28	9.00	22.7	50.0	92.0	146
Pan troglodytes	CHR_15 (Gene)		0.176	0.423	1.15	3.17	8.55	20.8	43.8	77.1	118
Pan troglodytes	CHR_15 (Intergenic)		0.179	0.433	1.19	3.35	9.32	24.1	55.1	105	172
Pan troglodytes	CHR_15 (Exon)		0.163	0.401	1.06	3.06	9.25	28.0	83.8	231	—
Pan troglodytes	CHR_15 (Intron)		0.177	0.423	1.15	3.15	8.38	20.1	41.7	72.4	110
Pan troglodytes	CHR_16		0.163	0.395	1.08	2.94	7.81	18.6	38.0	64.9	97.6
Pan troglodytes	CHR_16 (Gene)		0.152	0.371	1.00	2.70	7.01	16.1	31.7	52.2	76.6
Pan troglodytes	CHR_16 (Intergenic)		0.169	0.409	1.12	3.08	8.28	20.1	42.2	74.0	113
Pan troglodytes	CHR_16 (Exon)		0.154	0.379	0.963	2.70	7.94	23.2	68.1	189	—
Pan troglodytes	CHR_16 (Intron)		0.152	0.369	0.994	2.65	6.77	15.2	29.2	47.3	68.6
Pan troglodytes	CHR_17		0.155	0.378	1.02	2.75	7.11	16.2	31.4	51.3	74.5
Pan troglodytes	CHR_17 (Gene)		0.153	0.372	0.999	2.67	6.82	15.3	29.3	47.1	67.9
Pan troglodytes	CHR_17 (Intergenic)		0.158	0.383	1.04	2.82	7.36	17.0	33.6	55.5	81.4
Pan troglodytes	CHR_17 (Exon)		0.153	0.380	0.972	2.74	8.13	24.0	71.4	205	—
Pan troglodytes	CHR_17 (Intron)		0.151	0.365	0.974	2.57	6.44	14.1	26.2	41.3	58.9
Pan troglodytes	CHR_18		0.205	0.485	1.33	3.74	10.5	27.9	67.3	138	240

next

			L_e (kb)								
Category	SN	\ k	2	3	4	5	6	7	8	9	10
Pan troglodytes	CHR_18 (Gene)		0.196	0.465	1.27	3.56	9.88	25.4	57.9	111	183
Pan troglodytes	CHR_18 (Intergenic)		0.210	0.496	1.36	3.83	10.9	29.3	72.4	154	278
Pan troglodytes	CHR_18 (Exon)		0.191	0.460	1.22	3.53	10.6	31.7	88.9	—	—
Pan troglodytes	CHR_18 (Intron)		0.197	0.465	1.27	3.53	9.71	24.6	55.2	104	169
Pan troglodytes	CHR_19		0.145	0.344	0.902	2.32	5.62	11.8	21.0	32.1	44.9
Pan troglodytes	CHR_19 (Gene)		0.140	0.336	0.878	2.25	5.44	11.4	20.3	31.0	43.3
Pan troglodytes	CHR_19 (Intergenic)		0.149	0.352	0.925	2.38	5.79	12.1	21.7	33.2	46.5
Pan troglodytes	CHR_19 (Exon)		0.173	0.416	1.06	2.94	8.47	23.6	63.2	154	—
Pan troglodytes	CHR_19 (Intron)		0.136	0.324	0.839	2.11	4.93	9.97	17.2	25.7	35.5
Pan troglodytes	CHR_20		0.161	0.396	1.09	3.06	8.51	21.8	49.0	91.8	147
Pan troglodytes	CHR_20 (Gene)		0.157	0.385	1.06	2.96	8.14	20.5	44.9	81.6	128
Pan troglodytes	CHR_20 (Intergenic)		0.164	0.403	1.11	3.13	8.72	22.6	51.6	98.4	160
Pan troglodytes	CHR_20 (Exon)		0.159	0.394	1.01	2.86	8.47	24.9	71.8	—	—
Pan troglodytes	CHR_20 (Intron)		0.154	0.377	1.03	2.86	7.76	19.1	40.8	72.3	111
Pan troglodytes	CHR_21		0.199	0.471	1.29	3.63	10.2	26.5	62.0	122	205
Pan troglodytes	CHR_21 (Gene)		0.187	0.446	1.22	3.42	9.47	24.1	53.8	100	161
Pan troglodytes	CHR_21 (Intergenic)		0.204	0.483	1.33	3.73	10.5	27.6	65.8	133	228
Pan troglodytes	CHR_21 (Exon)		0.176	0.422	1.09	3.08	9.07	26.0	68.6	—	—
Pan troglodytes	CHR_21 (Intron)		0.185	0.438	1.20	3.34	9.11	22.7	49.3	89.5	141
Pan troglodytes	CHR_22		0.140	0.343	0.930	2.53	6.67	15.8	32.1	54.3	80.3
Pan troglodytes	CHR_22 (Gene)		0.139	0.340	0.918	2.48	6.51	15.3	30.5	50.7	74.3
Pan troglodytes	CHR_22 (Intergenic)		0.141	0.346	0.941	2.57	6.83	16.4	33.9	58.1	86.7
Pan troglodytes	CHR_22 (Exon)		0.142	0.351	0.885	2.46	7.20	20.8	59.9	—	—
Pan troglodytes	CHR_22 (Intron)		0.136	0.331	0.893	2.40	6.18	14.2	27.6	45.1	65.4
Pan troglodytes	CHR_X		0.207	0.491	1.35	3.78	10.6	28.3	68.7	142	250
Pan troglodytes	CHR_X (Gene)		0.203	0.487	1.34	3.75	10.5	27.2	63.5	125	209
Pan troglodytes	CHR_X (Intergenic)		0.208	0.492	1.35	3.78	10.7	28.6	70.3	148	264
Pan troglodytes	CHR_X (Exon)		0.169	0.429	1.15	3.32	10.1	30.5	90.3	242	—
Pan troglodytes	CHR_X (Intron)		0.205	0.489	1.34	3.73	10.3	26.6	61.1	118	196
Pan troglodytes	CHR_Y		0.189	0.440	1.20	3.37	9.53	25.5	62.7	130	231
Pan troglodytes	CHR_Y (Gene)		0.196	0.459	1.25	3.46	9.52	24.1	53.9	100	—
Pan troglodytes	CHR_Y (Intergenic)		0.187	0.437	1.19	3.34	9.48	25.5	63.1	132	234
Pan troglodytes	CHR_Y (Exon)		0.168	0.433	1.14	3.16	8.45	—	—	—	—
Pan troglodytes	CHR_Y (Intron)		0.199	0.459	1.24	3.40	9.22	22.8	49.7	—	—

next

Category	SN \ k	L_e (kb) 2	3	4	5	6	7	8	9	10
Rattus norvegicus	CHR_01	0.200	0.449	1.23	3.38	9.33	23.6	51.9	91.7	133
Rattus norvegicus	CHR_01 (Gene)	0.193	0.432	1.19	3.28	9.08	23.1	51.4	92.5	137
Rattus norvegicus	CHR_01 (Intergenic)	0.205	0.459	1.26	3.44	9.44	23.7	51.6	89.9	129
Rattus norvegicus	CHR_01 (Exon)	0.177	0.422	1.12	3.20	9.69	29.6	91.4	276	751
Rattus norvegicus	CHR_01 (Intron)	0.193	0.428	1.17	3.21	8.78	21.9	47.4	83.2	121
Rattus norvegicus	CHR_02	0.226	0.502	1.36	3.73	10.3	25.9	57.5	102	149
Rattus norvegicus	CHR_02 (Gene)	0.226	0.497	1.36	3.71	10.2	25.8	57.1	102	151
Rattus norvegicus	CHR_02 (Intergenic)	0.226	0.502	1.36	3.72	10.2	25.8	57.2	101	146
Rattus norvegicus	CHR_02 (Exon)	0.203	0.476	1.26	3.65	11.1	33.9	103	295	—
Rattus norvegicus	CHR_02 (Intron)	0.227	0.495	1.35	3.67	9.99	24.8	53.9	94.5	138
Rattus norvegicus	CHR_03	0.206	0.459	1.26	3.46	9.53	24.0	53.0	94.0	138
Rattus norvegicus	CHR_03 (Gene)	0.197	0.441	1.21	3.35	9.26	23.5	52.2	94.1	140
Rattus norvegicus	CHR_03 (Intergenic)	0.211	0.470	1.28	3.51	9.62	24.1	52.7	92.3	134
Rattus norvegicus	CHR_03 (Exon)	0.185	0.436	1.15	3.30	9.99	30.6	94.0	279	—
Rattus norvegicus	CHR_03 (Intron)	0.196	0.436	1.20	3.29	8.99	22.4	48.5	85.3	125
Rattus norvegicus	CHR_04	0.215	0.476	1.30	3.56	9.77	24.5	53.7	94.0	136
Rattus norvegicus	CHR_04 (Gene)	0.211	0.466	1.27	3.50	9.64	24.3	53.4	94.5	138
Rattus norvegicus	CHR_04 (Intergenic)	0.217	0.482	1.31	3.58	9.79	24.5	53.3	92.4	132
Rattus norvegicus	CHR_04 (Exon)	0.187	0.440	1.16	3.34	10.1	30.9	93.9	270	—
Rattus norvegicus	CHR_04 (Intron)	0.212	0.464	1.27	3.46	9.46	23.5	50.8	88.6	128
Rattus norvegicus	CHR_05	0.205	0.460	1.26	3.46	9.52	24.0	52.8	93.5	137
Rattus norvegicus	CHR_05 (Gene)	0.194	0.433	1.19	3.27	8.98	22.5	49.4	87.9	130
Rattus norvegicus	CHR_05 (Intergenic)	0.211	0.473	1.29	3.53	9.71	24.4	53.7	94.3	136
Rattus norvegicus	CHR_05 (Exon)	0.181	0.430	1.14	3.27	9.87	30.1	91.9	268	—
Rattus norvegicus	CHR_05 (Intron)	0.194	0.429	1.18	3.21	8.72	21.5	46.1	80.2	117
Rattus norvegicus	CHR_06	0.213	0.473	1.29	3.54	9.72	24.4	53.6	94.3	137
Rattus norvegicus	CHR_06 (Gene)	0.208	0.459	1.26	3.46	9.53	24.1	53.3	95.2	141
Rattus norvegicus	CHR_06 (Intergenic)	0.215	0.479	1.31	3.57	9.77	24.4	53.2	92.5	133
Rattus norvegicus	CHR_06 (Exon)	0.198	0.466	1.22	3.50	10.6	31.9	95.1	261	—
Rattus norvegicus	CHR_06 (Intron)	0.207	0.455	1.25	3.41	9.32	23.3	50.7	89.3	131
Rattus norvegicus	CHR_07	0.210	0.468	1.28	3.52	9.67	24.3	53.5	94.7	139
Rattus norvegicus	CHR_07 (Gene)	0.204	0.452	1.24	3.42	9.42	23.8	52.6	94.6	141
Rattus norvegicus	CHR_07 (Intergenic)	0.213	0.475	1.30	3.55	9.73	24.4	53.2	93.1	135
Rattus norvegicus	CHR_07 (Exon)	0.186	0.444	1.16	3.34	10.1	30.5	92.5	269	—

next

Category	SN \ k	L_e (kb) 2	3	4	5	6	7	8	9	10
Rattus norvegicus	CHR_07 (Intron)	0.204	0.448	1.23	3.35	9.12	22.6	48.8	85.6	126
Rattus norvegicus	CHR_08	0.202	0.447	1.22	3.33	9.07	22.4	48.0	82.9	119
Rattus norvegicus	CHR_08 (Gene)	0.195	0.430	1.18	3.22	8.75	21.6	46.4	80.9	118
Rattus norvegicus	CHR_08 (Intergenic)	0.206	0.457	1.25	3.39	9.22	22.7	48.5	82.8	118
Rattus norvegicus	CHR_08 (Exon)	0.190	0.447	1.17	3.37	10.2	31.1	94.9	276	—
Rattus norvegicus	CHR_08 (Intron)	0.194	0.425	1.16	3.15	8.47	20.6	43.3	74.1	107
Rattus norvegicus	CHR_09	0.218	0.481	1.31	3.57	9.72	24.1	52.0	90.2	130
Rattus norvegicus	CHR_09 (Gene)	0.214	0.469	1.28	3.51	9.58	23.9	51.8	91.0	133
Rattus norvegicus	CHR_09 (Intergenic)	0.220	0.486	1.32	3.59	9.76	24.1	51.6	88.6	127
Rattus norvegicus	CHR_09 (Exon)	0.189	0.449	1.18	3.40	10.3	31.4	94.9	267	—
Rattus norvegicus	CHR_09 (Intron)	0.215	0.467	1.27	3.46	9.36	22.9	48.9	84.3	122
Rattus norvegicus	CHR_10	0.182	0.406	1.11	3.01	8.07	19.4	40.3	67.9	97.1
Rattus norvegicus	CHR_10 (Gene)	0.175	0.393	1.08	2.95	8.00	19.7	42.0	73.1	107
Rattus norvegicus	CHR_10 (Intergenic)	0.189	0.417	1.14	3.05	8.06	19.0	38.3	62.8	87.8
Rattus norvegicus	CHR_10 (Exon)	0.168	0.402	1.04	2.97	8.93	27.0	82.0	242	—
Rattus norvegicus	CHR_10 (Intron)	0.175	0.389	1.07	2.89	7.70	18.5	38.4	65.1	93.9
Rattus norvegicus	CHR_11	0.229	0.505	1.37	3.75	10.3	25.8	56.6	100	146
Rattus norvegicus	CHR_11 (Gene)	0.224	0.491	1.34	3.67	10.1	25.2	55.2	98.2	145
Rattus norvegicus	CHR_11 (Intergenic)	0.232	0.511	1.39	3.78	10.3	25.9	56.7	99.3	144
Rattus norvegicus	CHR_11 (Exon)	0.197	0.462	1.22	3.50	10.5	31.9	94.4	250	—
Rattus norvegicus	CHR_11 (Intron)	0.220	0.480	1.31	3.57	9.69	23.9	51.5	90.0	132
Rattus norvegicus	CHR_12	0.176	0.379	1.02	2.69	6.92	15.8	31.2	51.1	72.5
Rattus norvegicus	CHR_12 (Gene)	0.173	0.375	1.02	2.71	7.12	16.7	34.1	57.2	82.3
Rattus norvegicus	CHR_12 (Intergenic)	0.179	0.382	1.03	2.66	6.70	14.9	28.5	45.5	63.8
Rattus norvegicus	CHR_12 (Exon)	0.180	0.420	1.07	3.04	9.04	26.8	78.8	213	—
Rattus norvegicus	CHR_12 (Intron)	0.169	0.362	0.979	2.57	6.60	15.1	29.9	49.0	69.7
Rattus norvegicus	CHR_13	0.219	0.480	1.30	3.53	9.53	23.3	49.2	83.2	118
Rattus norvegicus	CHR_13 (Gene)	0.215	0.467	1.27	3.45	9.31	22.7	47.9	81.1	115
Rattus norvegicus	CHR_13 (Intergenic)	0.221	0.485	1.31	3.55	9.58	23.4	49.3	83.0	117
Rattus norvegicus	CHR_13 (Exon)	0.190	0.446	1.18	3.40	10.3	31.2	93.3	254	—
Rattus norvegicus	CHR_13 (Intron)	0.215	0.464	1.26	3.40	9.09	21.8	45.2	75.5	106
Rattus norvegicus	CHR_14	0.222	0.492	1.34	3.68	10.1	25.6	56.8	101	150
Rattus norvegicus	CHR_14 (Gene)	0.217	0.477	1.30	3.59	9.89	25.0	55.8	101	151
Rattus norvegicus	CHR_14 (Intergenic)	0.224	0.498	1.36	3.71	10.2	25.7	56.7	100	146

next

		L_e (kb)								
Category	SN \ k	2	3	4	5	6	7	8	9	10
Rattus norvegicus	CHR_14 (Exon)	0.201	0.470	1.23	3.54	10.7	32.3	96.3	261	—
Rattus norvegicus	CHR_14 (Intron)	0.216	0.471	1.29	3.52	9.60	23.9	52.1	92.2	136
Rattus norvegicus	CHR_15	0.223	0.492	1.34	3.67	10.1	25.4	55.8	98.7	144
Rattus norvegicus	CHR_15 (Gene)	0.214	0.469	1.28	3.52	9.65	24.2	53.1	94.1	138
Rattus norvegicus	CHR_15 (Intergenic)	0.226	0.502	1.36	3.72	10.2	25.6	56.4	99.2	144
Rattus norvegicus	CHR_15 (Exon)	0.188	0.440	1.15	3.30	9.91	29.8	87.2	227	—
Rattus norvegicus	CHR_15 (Intron)	0.214	0.465	1.27	3.46	9.40	23.2	50.0	87.1	127
Rattus norvegicus	CHR_16	0.220	0.485	1.32	3.61	9.88	24.7	53.9	94.3	137
Rattus norvegicus	CHR_16 (Gene)	0.213	0.466	1.27	3.49	9.58	24.1	52.9	93.9	139
Rattus norvegicus	CHR_16 (Intergenic)	0.224	0.494	1.34	3.66	9.98	24.8	53.9	93.4	134
Rattus norvegicus	CHR_16 (Exon)	0.203	0.470	1.23	3.52	10.6	31.8	93.1	244	—
Rattus norvegicus	CHR_16 (Intron)	0.210	0.455	1.24	3.38	9.19	22.7	48.8	85.1	124
Rattus norvegicus	CHR_17	0.210	0.461	1.25	3.42	9.28	22.9	49.0	84.1	120
Rattus norvegicus	CHR_17 (Gene)	0.211	0.460	1.25	3.43	9.35	23.2	50.2	87.3	126
Rattus norvegicus	CHR_17 (Intergenic)	0.209	0.460	1.25	3.40	9.21	22.6	48.1	81.8	116
Rattus norvegicus	CHR_17 (Exon)	0.199	0.465	1.22	3.52	10.6	32.1	94.4	246	—
Rattus norvegicus	CHR_17 (Intron)	0.211	0.458	1.25	3.39	9.18	22.5	47.8	81.9	117
Rattus norvegicus	CHR_18	0.219	0.482	1.31	3.58	9.76	24.3	52.6	91.4	132
Rattus norvegicus	CHR_18 (Gene)	0.218	0.476	1.30	3.55	9.68	24.1	52.3	91.8	135
Rattus norvegicus	CHR_18 (Intergenic)	0.219	0.484	1.32	3.58	9.75	24.2	52.2	89.9	129
Rattus norvegicus	CHR_18 (Exon)	0.214	0.489	1.28	3.64	10.9	31.8	88.3	208	—
Rattus norvegicus	CHR_18 (Intron)	0.218	0.473	1.29	3.50	9.48	23.3	49.8	86.3	126
Rattus norvegicus	CHR_19	0.195	0.429	1.17	3.16	8.49	20.5	42.9	72.9	105
Rattus norvegicus	CHR_19 (Gene)	0.185	0.407	1.11	3.02	8.13	19.8	41.8	72.0	105
Rattus norvegicus	CHR_19 (Intergenic)	0.201	0.442	1.20	3.24	8.67	20.8	43.2	72.3	102
Rattus norvegicus	CHR_19 (Exon)	0.185	0.433	1.11	3.17	9.45	28.1	82.0	217	—
Rattus norvegicus	CHR_19 (Intron)	0.183	0.400	1.09	2.94	7.82	18.7	38.6	65.4	94.0
Rattus norvegicus	CHR_20	0.209	0.457	1.24	3.37	9.03	21.9	46.3	79.9	117
Rattus norvegicus	CHR_20 (Gene)	0.194	0.420	1.14	3.09	8.27	20.0	42.2	73.6	109
Rattus norvegicus	CHR_20 (Intergenic)	0.218	0.475	1.29	3.49	9.35	22.6	47.7	81.7	118
Rattus norvegicus	CHR_20 (Exon)	0.181	0.430	1.11	3.14	9.30	27.0	73.8	169	—
Rattus norvegicus	CHR_20 (Intron)	0.194	0.416	1.13	3.02	7.94	18.8	38.6	65.9	96.8
Rattus norvegicus	CHR_X	0.223	0.508	1.38	3.79	10.5	26.8	60.4	107	154
Rattus norvegicus	CHR_X (Gene)	0.227	0.518	1.41	3.91	10.9	28.1	64.2	118	174

next

Category	SN	\ k	L_e (kb)								
			2	3	4	5	6	7	8	9	10
Rattus norvegicus	CHR_X (Intergenic)		0.222	0.504	1.37	3.74	10.3	26.2	58.4	102	145
Rattus norvegicus	CHR_X (Exon)		0.181	0.450	1.21	3.51	10.6	32.3	95.8	258	—
Rattus norvegicus	CHR_X (Intron)		0.229	0.520	1.42	3.89	10.7	27.2	61.1	110	160
