# Supplementary material for: Universal Global Imprints of Genome Growth and Evolution – Equivalent Length and Cumulative Mutation Density
Source: PLoS One. 2010 Apr 14;5(4):e9844. doi: 10.1371/journal.pone.0009844 (PMC2854691; doi:10.1371/journal.pone.0009844)
Supplement: Table S3 — Le(k), k = 2 to 10, averaged over categories of organisms. (0.06 MB PDF) [file pone.0009844.s006.pdf]

Table S3. $L_e(k)$, k=2 to 10, averaged over categories of organisms

For each complete chromosome or concatenate $L_e(k)$ is computed if the sequence length is greater than 4^{k+1}. The L_e's given are mean±SD average over categories of sequences: archaea , bacteria, insects, unicells, plants, vertebrates, and "all". Total sequence length is 2.2×10[10] bp. See Table S1 for list of complete sequences and S2 for $L_e(k)$'s of all chromosomes.

	L_e (kb)[d]								
Category \ k	2	3	4	5	6	7	8	9	10
Aechaea (32)[a]	.362±.228	.759±.401	1.75±.83	4.51±2.09	12.3±5.8	35.3±16.2	99.0±45.1	270±113	971±32
Aechaea (g; 85.4%)[b]	.335±.208	.700±.360	1.61±.75	4.14±1.89	11.5±5.1	32.5±14.6	92.3±41.8	252±105	879±0
Aechaea (ig; 14.5%)[b]	.519±.389	.964±.535	2.21±1.22	5.47±2.92	13.4±6.7	30.5±14.3	83.0±21.9	226±19	—
Bacteria (435)	.356±.251	.729±.459	1.68±.94	4.29±2.29	11.6±6.1	32.3±17.1	90.7±48.1	253±126	669±349
Bacteria (g; 87.6%)[b]	.332±.228	.674±.405	1.55±.83	3.96±2.05	10.7±5.5	29.7±15.6	83.6±43.9	231±116	573±349
Bacteria (ig; 12.3%)[b]	.451±.355	.912±.623	2.04±1.21	5.02±2.76	12.8±6.7	32.0±16.3	84.8±34.0	240±80	—
Insects (39)[a]	.222±.098	.513±.218	1.30±.58	3.39±1.61	8.88±4.43	22.5±11.9	55.5±32.0	132±87	298±231
Insects (g; 40.8%)[b]	.235±.099	.545±.227	1.38±.61	3.60±1.66	9.42±4.61	23.9±12.5	58.9±33.7	138±92	399±253
Insects (ig; 59.1%)[b]	.216±.104	.492±.219	1.24±.57	3.22±1.53	8.35±4.02	20.9±10.3	50.7±27.7	119±77	261±196
Insects (e; 13%)[b,c]	.364±.161	.884±.380	2.14±.83	5.77±2.20	16.0±5.9	43.0±14.6	109±33	261±92	761±268
Insects (i; 27.9%)[b,c]	.210±.101	.475±.215	1.19±.57	3.05±1.52	7.83±4.14	19.5±11.2	47.0±30.1	108±81	312±217
Plants (17)[a]	.672±.193	1.50±.41	3.40±.78	8.66±1.80	22.7±4.3	57.6±10.2	145±24	343±49	718±77
Plants (g; 47.5%)[b]	.458±.084	1.18±.24	2.67±.40	6.98±.94	19.3±2.3	52.3±5.2	142±10	369±14	857±89
Plants (ig; 52.5%)[b]	.815±.272	1.48±.44	3.33±.91	8.15±2.08	20.0±5.0	47.2±12.0	109±28	232±59	428±97
Plants (e; 31%)[b,c]	.399±.075	.996±.187	2.06±.20	5.23±.35	14.3±.6	38.4±1.4	103±8	265±41	607±170
Plants (i; 24.3%)[b,c]	.284±.051	.747±.068	1.94±.18	5.14±.61	13.7±2.0	35.8±6.0	93.1±16.9	233±44	557±74
Unicells (106)[a]	.716±.340	1.26±.57	2.95±1.38	7.84±3.85	22.1±11.3	61.0±32.5	169±91	435±252	1436±459
Unicells (g; 64.4%)[b]	.514±.159	1.12±.40	2.57±.99	6.78±2.88	19.1±8.8	52.4±26.0	146±74	421±193	1130±0
Unicells (ig; 35.5%)[b]	1.09±1.28	1.51±1.04	3.38±2.27	8.18±5.43	20.1±13.9	49.9±35.7	118±93	427±261	—
Unicells (e; 52.1%)[b,c]	.529±.129	1.17±.36	2.59±.95	6.77±2.87	19.0±8.9	52.4±26.6	138±74	414±179	—
Unicells (i; 3.7%)[b,c]	.884±.775	1.28±.97	2.26±1.79	4.63±4.30	11.0±12.4	26.0±29.7	117±58	—	—
Vertebrates (236)[a]	.202±.049	.465±.090	1.26±.23	3.48±.55	9.49±1.23	24.2±3.2	55.9±11.7	113±41	200±120
Vertebrates (g; 38%)[b]	.199±.048	.458±.089	1.24±.23	3.42±.56	9.36±1.31	23.8±3.6	55.4±13.3	113±47	200±133
Vertebrates (ig; 63.5%)[b]	.204±.049	.469±.091	1.27±.23	3.49±.55	9.52±1.20	24.2±3.1	55.7±11.3	111±38	189±98
Vertebrates (e; 2%)[b,c]	.193±.033	.452±.055	1.18±.13	3.33±.41	9.76±1.45	28.0±5.6	78.7±21.3	203±81	491±301
Vertebrates (i; 34.8%)[b,c]	.197±.049	.454±.089	1.23±.23	3.37±.54	9.13±1.28	23.0±3.7	52.7±13.2	106±45	184±121
All (865)[a]	.359±.273	.729±.477	1.74±1.02	4.56±2.63	12.4±7.3	33.7±20.8	89.6±58.5	223±154	388±339
All (g; 41.8%)[b]	.317±.204	.676±.395	1.61±.83	4.21±2.16	11.5±6.1	31.2±17.5	82.8±49.8	202±130	337±292
All (ig; 59.6%)[b]	.462±.588	.859±.676	1.99±1.39	4.99±3.20	12.7±7.8	31.6±19.0	76.5±45.0	137±104	213±130
All (e; 3.3%)[b,c]	.292±.163	.673±.372	1.62±.80	4.40±2.12	12.6±6.1	35.3±17.2	95.8±46.7	243±125	620±253
All (i; 31.8%)[b,c]	.348±.455	.644±.568	1.47±.96	3.65±2.04	9.46±5.06	23.5±11.4	55.0±21.1	114±59	213±155

[a] Number in parentheses indicates total number of complete chromosomes in category.

[b] Abbreviations: g, gene; ig, intergenic; e, exon; i, intron. A "g" sequence, for instance, is the concatenation of all genes in a complete genome or chromosome. Genes are from a single strand and are included in both positive and negative orientation. Percentage given indicates portion of complete sequence. "N-runs" or gaps in sequences are not counted.

[c] exon and intron segments selected as given by Genbank. There may be incomplete or include duplicates, hence sum of percentages for exon and intron may be less or exceed that of gene.

[d] $L_e(k)$ computed only if category has more than one sequence whose length exceeds 4^{k+1}.
