# Supplementary material for: Universal Global Imprints of Genome Growth and Evolution – Equivalent Length and Cumulative Mutation Density
Source: PLoS One. 2010 Apr 14;5(4):e9844. doi: 10.1371/journal.pone.0009844 (PMC2854691; doi:10.1371/journal.pone.0009844)
Supplement: Table S4 — Le of sequences with highly biased compositions. (0.06 MB PDF) [file pone.0009844.s007.pdf]

Table S4. L_e of sequences with highly biased compositions

Notation for parts are: *gn*, genes; *ig*, inter-genic; *ex*, exon; *in*, intron; *p*, fractional AT-content; q=1-p. $L_e(k)$ computed only if sequence length is greater than 4^{k+1}. The universality class is defined in the main text.

Organism	Sequence ID	Length (Mb)	max(p,q*)	$L_e(k)$ (kb) k=2	3	5	7	9
All genes		0.2–230	0.5–0.8	.317±.204	.676±.395	4.21±2.16	31.2±17.5	202±130
Universality class			0.50	.31±.21	.78±.50	4.9±3.2	31±20	194±125
D. hansenii	NC_006049 (*ig*)	0.45	0.68	6.97	3.43	17.4	81.3	—
A. mellifera	Chr. LG7 (*in*)	2.53	0.68	0.214	0.471	2.79	14.9	59.9
C. elegans	Chr. 4 (*in*)	5.35	0.69	0.081	0.159	0.881	6.21	44.7
C. jejuni	NC_002163 (*gn*)	1.56	0.69	0.131	0.253	1.56	11.0	80.4
Universality class			0.70	.31±.21	.69±.44	3.4±2.2	17±11	82±53
M. stadtmanae	NC_007681 (*gn*)	1.50	0.71	0.732	1.64	5.72	29.9	176
N. farcinica	NC_006361 (*gn*)	5.45	0.71*	0.206	0.380	1.69	9.95	66.8
S. coelicolor	NC_003888 (*gn*)	7.76	0.71*	0.421	0.710	2.58	14.3	95.2
F. alni	NC_008278 (*gn*)	6.47	0.73*	0.253	0.461	1.83	10.8	72.3
F. nucleatum	NC_003454 (*gn*)	1.95	0.73	0.240	0.508	2.68	15.6	98.6
B. aphidicola	NC_004545 (*gn*)	0.51	0.73	0.646	0.952	3.88	21.3	—
M. penetrans	NC_004432 (*gn*)	1.21	0.74	0.224	0.558	3.16	17.9	106
Universality class			0.75	.31±.21	.65±.42	2.8±1.8	12±7.7	54±39
M. mycoides	NC_005364 (*gn*)	0.99	0.76	0.210	0.470	2.57	13.8	—
M. capricolum	NC_007633 (*gn*)	0.91	0.76	0.204	0.455	2.50	13.6	—
P. falciparum	Chr. 2 (*ex*)	0.46	0.76	0.523	0.670	2.16	9.24	—
P. falciparum	Chr. 14 (*ex*)	1.78	0.78	0.561	0.757	2.16	8.47	30.6
F. nucleatum	NC_003454 (*ig*)	0.21	0.78	0.253	0.467	2.05	10.3	—
B. aphidicola	NC_008513 (*gn*)	0.36	0.79	0.237	0.313	0.925	3.65	—
B. afzelii PKo	NC_008277 (*ig*)	0.05	0.79	0.232	0.490	2.42	—	—
Universality class			0.80	.31±.21	.60±.39	2.3±1.5	8.7±5.6	33±21
C. jejuni	NC_002163 (*ig*)	0.07	0.81	0.093	0.182	0.911	4.70	—
M. penetrans	NC_004432 (*ig*)	0.14	0.82	0.206	0.432	2.05	8.71	—
M. capricolum	NC_007633 (*ig*)	0.09	0.82	0.241	0.433	1.78	7.84	—
M. stadtmanae	NC_007681(*ig*)	0.26	0.83	0.838	0.856	1.97	7.60	—
C. C. ruddii	NC_008512 (*gn*)	0.15	0.84	0.119	0.206	0.672	2.41	—
B. aphidicola	NC_004545 (*ig*)	0.10	0.85	0.736	1.02	3.26	12.1	—
Universality class			0.85	.31±.21	.55±.35	1.8±1.2	5.7±3.7	18±12
B. aphidicola Sg	NC_004061 (*ig*)	0.05	0.86	0.161	0.215	0.633	—	—
P. falciparum	Chr. 2 (*ig*)	0.42	0.85	0.819	0.143	0.205	0.361	—
P. falciparum	Chr. 2 (*in*)	0.05	0.87	0.148	0.056	0.070	—	—
P. falciparum	Chr. 14 (*ig*)	1.32	0.87	0.781	0.100	0.151	0.260	0.430
P. falciparum	Chr. 14 (*in*)	0.18	0.87	0.148	0.053	0.076	0.116	—
Universality class			0.90	.31±.21	.50±.32	1.3±0.8	3.2±2.1	8.5±5.5
B. aphidicola	NC_008513 (*ig*)	0.05	0.92	0.756	0.470	1.14	—	—
