# Supplementary material for: Universal Global Imprints of Genome Growth and Evolution – Equivalent Length and Cumulative Mutation Density
Source: PLoS One. 2010 Apr 14;5(4):e9844. doi: 10.1371/journal.pone.0009844 (PMC2854691; doi:10.1371/journal.pone.0009844)
Supplement: Table S5 — Effect of replication and segmental duplication on le. (0.04 MB PDF) [file pone.0009844.s008.pdf]

Table S5. Effect of replication and segmental duplication on l_e

Equivalent length (l_e) of a sequence after an n-fold increase in length via three basic modes of growth. Initial sequence length is $l_0 >> 1$, final sequence length is $L = nl_0$.

Sequence type	Initial l_e	Mode of growth	Final l_e
Random	l_0	Random base-by-base growth	$L(=nl_0)$
		Whole-sequence replication (n-1 times)	$\approx l_0$
		Segmental duplication	$l_0 < l_e << nl_0$
Non-random	l_{e0} ($<< l_0$)	Random base-by-base growth	$\approx \min(n^2 l_{e0}, L)$
		Whole-sequence replication (n-1 times)	$\approx l_{e0}$
		Segmental duplication	$l_{e0} < l_e << \min(n^2 l_{e0}, L)$
