# Supplementary material for: Universal Global Imprints of Genome Growth and Evolution – Equivalent Length and Cumulative Mutation Density
Source: PLoS One. 2010 Apr 14;5(4):e9844. doi: 10.1371/journal.pone.0009844 (PMC2854691; doi:10.1371/journal.pone.0009844)
Supplement: Text S1 — (0.07 MB PDF) [file pone.0009844.s009.pdf]

Section S1. L_e for coding and non-codong parts

Our study suggests that a general rule is that the L_e's for coding and noncoding parts essentially do not differ. Fig. S1 shows the p dependence of L_e for coding and noncoding concatenates averaged over categories of genomes, for k=7 (situations for other k's are similar). For eukaryotes coding and noncoding are the exon (*ex*) and intron (*in*) parts, respectively, and for prokaryotes they are the genic (*gn*) and intergenic (*ig*) parts, respectively. The curve in the figure represents $L_e^{\{uc\}}(k;p)$, or the universality class. After the expected p dependence is accounted for, over all there is little difference in the coding and noncoding L_e's. Note that in most cases $L_e^{code}>L_e^{ncode}$, which seems counterintuitive because it implies coding regions are more random then non-coding regions. Part of this may be attributed to the fact that coding regions are relatively CG-rich, which is confirmed by the relation $p^{code}<p^{ncode}$ holding in all cases (p^{code} is the p value for the coding region). According to $L_e^{\{uc\}}(k;p)$ and with everything other than the p-value being equal (statistically), we expect $L_e^{code}>L_e^{ncode}$ if $p^{code}>0.5$, and $L_e^{code}<L_e^{ncode}$ if $p^{ncode}<0.5$. Only data for prokaryotes may seems to go against this trend. For unicellulars and insects the difference between L_e^{code} and L_e^{ncode} is seen to be significantly larger than that given by $L_e^{\{uc\}}(k;p)$.

Section S2. χ^2 for genomic $L_e(k)$ versus universality class

The χ^2_σ for a set σ $L_e(k)$'s versus $L_e^{\{uc\}}(k;p)$, the empirical representation of the data, or the universality class (see main text), is defined as,

$$\chi^2_\sigma = \frac{1}{N_\sigma}\sum_{i\in\{\sigma\}} \ln^2\left(\frac{L_{e,i}}{L_e^{\{uc\}}(k;p)}\right) \qquad (E1)$$

where N_σ is the total number of pieces of data in the data set $\{\sigma\}$. To leading order χ^2 is just the coefficient of variation squared, while the logarithm gives equal weights to L_e's that are greater and less than $L_e^{\{uc\}}$. Fig. S2 shows that χ^2 for a large majority of data is less than 0.5 (data and $L_e^{\{uc\}}$ differ by less than a factor of 2) and for a sizable fraction it is less than 0.1; not shown are about 3% of the data whose χ^2 is less than 10^{-3}. The χ^2 for the k=2, 4, 6, 8, 10 data sets (Fig. 2 in text), respectively, are 0.44 0.34 0.34 0.49 0.73. The relatively large χ^2 for k=10 is due to a smaller sample size, and short sequence length relative to ($4\times4^{10}\approx$) 4 Mb of a significant portion of the samples (see Methods). The χ^2 for the "All" data is 0.43. This implies that on average the genomic L_e is within a factor of 1.9 of $L_e^{\{uc\}}$, which in turn specifies the error on the quantity L_{e2} in the universality class. The universal formula works moderately better for prokaryotes (χ^2=0.39) than for eukaryotes (0.48), significantly better for *gn* (0.34) than for *ig* (0.48) parts, and much better for *ex* (0.23) than for *in* (0.57) parts. The most prominent outlying data are the $L_e^{ig}(k=2)$ of the seven chromosomes of the marine yeast *D. hansenii*, and the fourteen chromosomes of the malaria causing parasite *P. falciparum*, whose values are much greater than the universal lengths, and the $L_e^{ig}(k\neq2)$ of *P. falciparum* chromosomes, whose values are much less than the universal lengths. These cases are responsible for the data with $\chi^2\geq50$ in the bottom panels in Fig. S2.

The l_e is defined such that the bulk of its p-dependence is removed. However, genomic data indicate a residual p-dependence. The p-dependence in $a(p)$ is meant to represent this; it is symmetric with respect to p=0.5 and decreases with increasing $|p-0.5|$. Table S4

illustrates how well the formula represent the general trend of p-dependence in the data. Among the examples included are the endosymbiotic Gamma Proteobacteria *Candidatus Carsonella ruddii*, which at 150 kb and p=0.84 is the smallest and most AT-rich genome of any characterised organism, and the inter-genic parts of the two species of *B. aphidicola* (NC_004061 and NC_008513) that are only 50 kb long and have p=0.86 and 0.92, respectively.

Section S3. Replication and segmental duplication shortens equivalent length

We show that segmental duplication will cause a sequence to have an equivalent length l_e much shorter than its true length. Consider the effect on the l_e of a sequence of even base composition (for simplicity) and initial length l_0 having its length doubled via three routes: random base-by-base growth, whole-sequence replication, and random segmental duplication. For simplicity we assume for the moment that the original sequence is random so that l_e is, by definition, equal to l_0. Doubling by random base-by-base growth is the same as concatenating the original with another random sequence of equal length; the result is still a random sequence, hence l_e will also be doubled to $2l_0$. Doubling the original sequence by replication will double the frequency of every k-mer, which will in turn double both the standard deviation and the mean frequency, so that the coefficient of variation will remain unchanged. So will l_e since it is defined in terms of the coefficient variation (Eq. (13) in main text). That is, for the new sequence of length $2l_0$ we still have l_e=l_0. If the original sequence is a genomic sequence such that $l_e << l$, doubling its length by replication will again double the frequency of every k-mer and leave l_e unchanged. However, doubling the sequence by concatenating it with a random sequence of equal length will add to each k-mer frequency an amount approximately equal to the mean frequency of the original sequence (because a random sequence has a frequency distribution that is close to being uniform), this will cause the standard deviation (in the doubled sequence) to remain unchanged, the mean frequency doubled, the coefficient of variation halved, and l_e increased by a factor of 4. Similar reasoning applies when the length of a sequence is increased n-fold. Segmental duplication bridges the two extreme modes of growth: it approaches random base-by-base growth when the duplicated segments are very short, and approaches whole-sequence replication when the duplicated segment is comparable to the original sequence length. Results are summarized in Table S6.

Section S4. Additional results on minimum RSD model

Here we present additional discussion and results on the minimum RSD model. The model has three explicit parameters: L_0, the initial sequence length; $\bar{d}$, the average length of duplicated segments; r, the cumulative point mutation density (replacement only), or number of mutations per site. The "best" set of parameters, the set giving the smallest χ^2_σ (Eq. (E1), Section S2), are r=0.73, $\bar{d}$=1000, and L_0=64. For every set of model parameters r, $\bar{d}$, and L_0, 200 model sequences of length 2 Mb were generated. Results are shown in Fig. S3; the top panels are included in the main text and are repeated here for better integrity of presentation. The top-left panel is an equi-χ^2 plot in the r-$\bar{d}$ plane. The top-right panel shows $L_e(k)$'s for 200 model sequences generated in the RSD model whose distribution in p match that of the genomic sequences studied. Length on model sequences is 2 Mb. Each piece of data shown gives the $L_e(k)$ from one model sequnece. The lines are $L_e^{\{uc\}}(k;p)$ (Eq. (10), main text).

The bottom-left panel shows that with r and $\bar{d}$ fixed at their respective "best" values, χ^2 (for k=2 and for model sequences with p=0.5) grows rapidly with increasing L_0 with an approximate power-law relation. This rapid growth (but not the power law) can be understood as follows. As explained in Section S3, the growth of a sequence will generally cause its L_e to increase. If the initial sequence is random, then the initial L_e=L_0, and L_e will increase with subsequent sequence growth. In particular, base-by-base random growth will result in $L_e \approx L$ (sequence length), whereas whole sequence replication, the best way for preventing L_e from increasing, will result in $L_e \approx L_0$. Returning to the bottom-left panel of Fig. S3, the universal L_{e2} (L_e for k=2) about 310 b, so the lower limit of χ^2 is $\ln^2(L_0/310)$, which will grow rapidly with increasing L_0.

The bottom-right panel shows that $L_e(k)$ is insensitive to L provided that L is greater than 2 Mb. This may be interpreted as a manifest of homogeneity of the model sequences on a scale significantly shorter than 2 Mb but longer than $L_e(k)$ (for given k). Here homogeneity is used in the sense of having similar k-mer content. In a RSD model, when much of the length of the grown sequence is acquired by SD and if the length is much greater then $\bar{d}$, then, because the process is random, the sequence is expected to have reached a state of homogeneity. That genomes are essentially in such a state is evident in Fig. 2 (l_e leveling off as a function of sequence length) and Fig. 5 (high intra-sequence similarity).
